# Stereoselective construction of sterically hindered oxaspirocycles *via* chiral bidentate directing group-mediated C(sp^3^)–O bond formation†
†Electronic supplementary information (ESI) available: Experimental procedure, characterization of new compounds (^1^H and ^13^C NMR spectra), computational details, Cartesian coordinates of all of the calculated structures, additional discussions, and X-ray crystallographic data of **2a** (CCDC 1581871). For the ESI and crystallographic data in CIF or other electronic format see DOI: 10.1039/c7sc04691j


**DOI:** 10.1039/c7sc04691j

**Published:** 2017-11-27

**Authors:** Yechan Kim, Seoung-Tae Kim, Dahye Kang, Te-ik Sohn, Eunyoung Jang, Mu-Hyun Baik, Sungwoo Hong

**Affiliations:** a Department of Chemistry , Korea Advanced Institute of Science and Technology (KAIST) , Daejeon , 34141 , Korea . Email: hongorg@kaist.ac.kr ; Email: mbaik2805@kaist.ac.kr; b Center for Catalytic Hydrocarbon Functionalizations , Institute for Basic Science (IBS) , Daejeon 34141 , Korea

## Abstract

The systematic investigation of chiral bidentate auxiliaries has resulted in the discovery of a chiral 2,2-dimethyl-1-(pyridin-2-yl)propan-1-amine-derived directing group that enables stereoselective palladium(ii)-catalyzed intramolecular C(sp^3^)–O bond formation. This new chiral directing group exhibited high reactivity in the activation of methylene C(sp^3^)–H bonds with excellent levels of stereoselectivity (a diastereomeric ratio of up to 39 : 1), which allowed the construction of a wide range of oxaspirocycles. Mechanistic investigations were also conducted to elucidate the reaction mechanism and understand the origin of the diastereoselectivity. DFT calculations suggest that only modest levels of diastereoselectivity are accomplished at the rate-determining C–H metalation–deprotonation step and the d.r. is further enriched at the reductive elimination step.

## Abstract 2

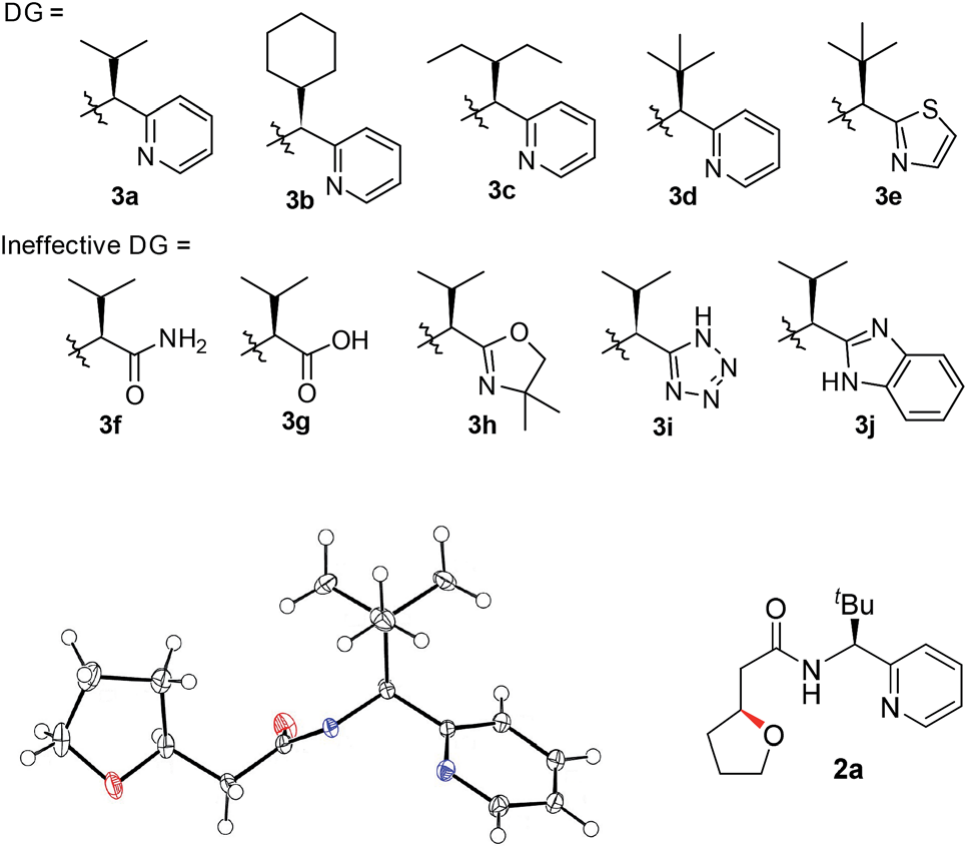
A new, bidentate, chiral directing group derived from 2,2-dimethyl-1-(pyridin-2-yl)propan-1-amine was discovered, which enables stereoselective palladium(ii)-catalyzed intramolecular C(sp^3^)–O bond formation.

## Introduction

Oxaspirocycles are important constituents of many biologically active molecules and natural products.^1^ They feature structural complexity and serve as privileged motifs that provide an opportunity to explore the three-dimensional space of structures, which allows for the fine tuning of physicochemical properties in medicinal applications,^2^ for example. Accordingly, extensive research efforts have been made to develop synthetic methods for accessing spiroether moieties.^3^ Retrosynthetic disconnections for the asymmetric synthesis of chiral cyclic ethers generally rely on intramolecular oxa-Michael reactions to tethered α,β-unsaturated carbonyl groups mediated by chiral catalysts.^4^,^5^ However, the stereoselective construction of sterically hindered oxygenated centers such as oxaspirocycles continues to be challenging owing to steric crowding and the resulting reduced nucleophilicity of the pendant alcohol.

Palladium-catalyzed direct C–O bond formation *via* the activation of a C(sp^3^)–H bond enabled by directing groups has emerged recently as a promising strategy.^6^,^7^ The intermolecular alkoxylation of methyl C–H bonds using a picolinamide-derived bidentate directing group (DG)^8^ was first demonstrated by Chen *et al.*^9^ The Shi^10^ and Rao^11^ groups reported elegant methods for the alkoxylation of unactivated methylene C(sp^3^)–H bonds by employing 2-pyridinylisopropyl amine- and 8-aminoquinoline-derived DGs,^8^,^12^ respectively. In addition, important advances have been made by Dong *et al.* in the intramolecular alkoxylation of methyl C–H bonds.^13^

Recently, examples of enantioselective benzylic C–H arylation using bidentate DGs and BINOL-based ligands were reported by Duan^14^ and Chen.^15^ Bidentate auxiliary directed C(sp^3^)–O bond formation using chiral ligands is attractive for the asymmetric construction of cyclic ethers and oxaspirocycles. But this approach has not yet been successful, partly because strongly coordinating bidentate DGs may prevent potentially powerful chiral bidentate ligands from binding^16^ and promote competing C–H alkoxylation without involving the ligand. We imagined that a properly constructed stereogenic unit in the bidentate DG may enable C–H functionalization in a stereoselective fashion without the need for external chiral ligands. If successful, these chiral DGs may be valuable additions to the synthetic chemistry toolbox and offer a new retrosynthetic disconnection strategy constructing sterically-hindered cyclic ethers and oxaspirocyclic structural motifs in a stereoselective fashion.

## Results and discussion

**Scheme 1:**
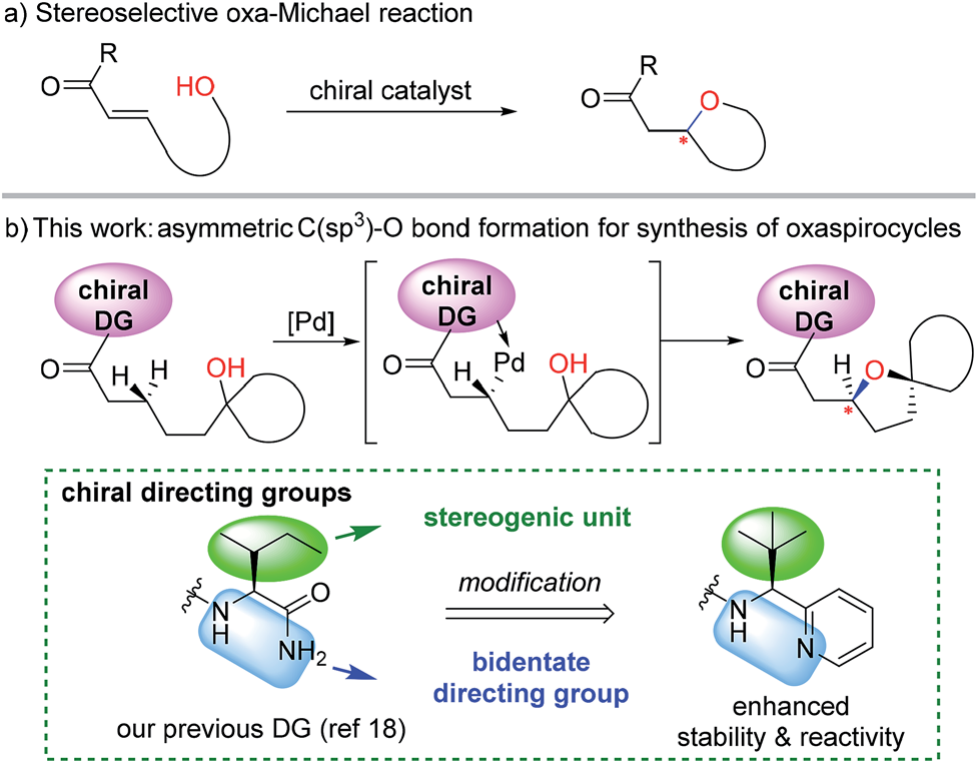
Different disconnections (conjugate addition *vs.* β-C–H functionalization) for the stereoselective synthesis of oxacycles.

Previously, we reported a highly stereoselective C–H arylation of cyclopropanes mediated by a chiral auxiliary that mainly utilized steric demands to impose stereocontrol of the reaction.^17^ A chiral substituent and two nitrogen atoms worked in concert to assemble the reactant complex and to enable the C–H activation in a stereoselective fashion. To apply the same strategy for direct C(sp^3^)–O bond formation, one challenge must be addressed. Initial attempts to carry out these reactions with previously developed DGs showed low stability of the amino acid amide moieties under the reaction conditions that are required for oxidation of the palladium to a high valent Pd(iv) state. Herein, we present the discovery of a new chiral bidentate DG that enables the stereoselective β-methylene C(sp^3^)–H bond functionalization/alkoxylation process to afford a series of oxaspirocycle scaffolds with diastereomeric ratios reaching 39 : 1 (Scheme 1).

**Fig. 1:**
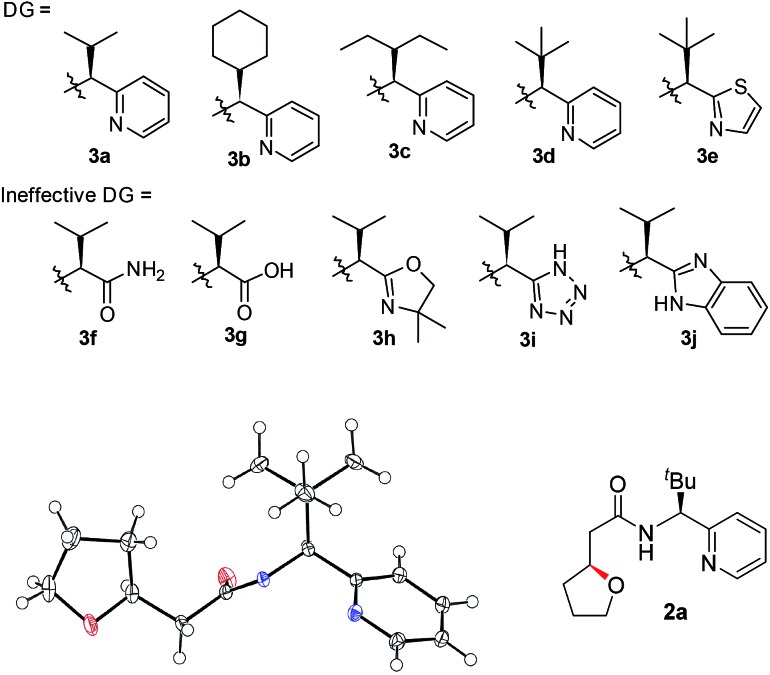
Chemical structures of effective and ineffective DGs (**3a**–**3j**). The X-ray crystal structure of **2a**.

A number of chiral bidentate DGs were tested for their ability to promote the stereoselective assembly of oxacycles while differentiating the β-methylene C–H bonds and using the pendant alcohol as an internal nucleophile. Since the amino acid amide DG (**3f**) did not give any reactivity, we refined the ligand design and evaluated various DGs to form chiral auxiliaries. As summarized in Table 1, the amino acid (**3g**), dihydrooxazole (**3h**), tetrazole (**3i**), and benzimidazole (**3j**) moieties did not give any reaction. The pyridyl or thiazolyl methanamine-type functionalities were found to be the most effective for C(sp^3^)–O bond formation. For example, a pyridyl methanamine auxiliary^18^ containing the isopropyl substituent (**3a**) led to the desired product with a 65% yield, while displaying meaningful levels of diastereoselectivity (entry 1, d.r. = 4.5 : 1), thus highlighting that our conceptual design is plausible. The moderate diastereoselectivity observed with an isobutyl substituent (**3a**) prompted us to scrutinize the effect of sterically demanding substituents on the stereochemical outcome. In particular, the alkyl substituents of the coordinating fragment were varied systematically. To prepare a series of these modified DGs, we used a highly efficient asymmetric imine addition with Ellman’s auxiliary^19^ from picolinaldehyde and optically pure sulfinamide. Intriguingly, a sterically bulky *t*-butyl group (**3d**) present on the directing group displayed drastically improved diastereoselectivity (entry 4, d.r. = 26 : 1) compared to those with isopropyl (**3a**), cyclohexyl (**3b**), or 3-pentyl (**3c**) substituents. Thus, **3d** was employed as an optimal bidentate DG for further reaction optimization; representative catalytic systems are listed in Table 1 (entries 6–12). The choice of additive was critical for both the reaction efficiency and diastereoselectivity, and AcOH was found to be the most effective. Under the optimized reaction conditions, the desired product (**2a**) was formed in 71% yield with excellent diastereoselectivity (entry 12, d.r. = 30 : 1). The absolute configuration of the product **2a** was unambiguously confirmed to be (*S*) by X-ray diffraction (Fig. 1). The DG could be removed under mild conditions^17^ to afford the corresponding carboxylic acids with conservation of the stereogenic center (93% ee).

**Table 1:** Screening of potential bidentate chiral auxiliaries and optimization of the reaction conditions^*a*^

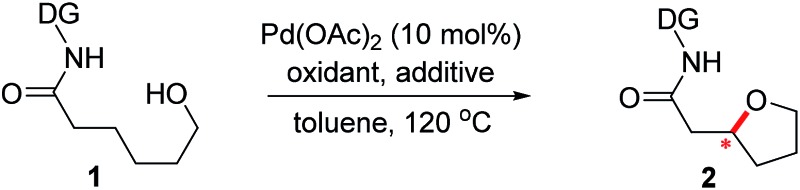				
Entry	DG	Oxidant (equiv.)	Additive (equiv.)	Yield^*c*^ (d.r.)^*d*^
1	**3a**	PhI(OAc)_2_ (2)	AcOH (4)	65% (4.5 : 1)
2	**3b**	PhI(OAc)_2_ (2)	AcOH (4)	64% (3.3 : 1)
3	**3c**	PhI(OAc)_2_ (2)	AcOH (4)	64% (6.2 : 1)
4	**3d**	PhI(OAc)_2_ (2)	AcOH (4)	63% (26 : 1)
5	**3e**	PhI(OAc)_2_ (2)	AcOH (4)	43% (19 : 1)
6	**3d**	K_2_S_2_O_8_ (2)	AcOH (4)	NR
7	**3d**	DMP (2)	AcOH (4)	NR
8	**3d**	PhI(OAc)_2_ (2)	—	46% (8.3 : 1)
9	**3d**	PhI(OAc)_2_ (2)	AgOAc (2)	39% (6.7 : 1)
10	**3d**	PhI(OAc)_2_ (2)	PivOH (4)	58% (23 : 1)
11	**3d**	PhI(OAc)_2_ (3)	AcOH (4)	66% (26 : 1)
12^*b*^	**3d**	PhI(OAc)_2_ (3)	AcOH (4)	71% (30 : 1)

^*a*^Substrate (1.0 equiv.), Pd(OAc)_2_ (10 mol%), oxidant, and additive in toluene (0.1 M) at 120 °C for 10 h.

^*b*^The reaction was carried out in a co-solvent system (toluene : EtOH = 10 : 1).

^*c*^The isolated yields of products.

^*d*^The d.r. was determined by HPLC analysis. DMP = Dess–Martin periodinane. NR = no reaction.

Having established a highly diastereoselective Pd(ii)-catalyzed C(sp^3^)–O bond forming reaction with the optimal DG, we turned our attention to the construction of valuable oxaspirocyclic motifs. We were delighted to observe that a wide range of sterically hindered tertiary alcohols can be employed to efficiently afford a variety of corresponding spiroethers with excellent levels of asymmetric induction, summarized in Table 2. The size of the spiro rings did not show much change in reactivity and selectivity to afford 5,4- (**2c**), 5,5- (**2j**), 5,6- (**2k**, **2l**, and **2g**), 5,7- (**2q**), and 5,8-ring (**2r**) systems. In addition, the secondary (**2d** and **2e**) and acyclic tertiary alcohol (**2b**) substrates gave their corresponding products in good yields. Further exploration demonstrated that spirocyclic ethers containing key structural motifs that are highly sought after in medicinal chemistry, such as azetidine (**2f**, **2g**, **2h**, **2i** and **2d**), tetrahydropyran (**2n**) and piperidine (**2o** and **2p**) can be efficiently accessed under these reaction conditions. In addition, oxaspirocycles bearing sterically bulky systems, as exemplified by the bicyclo[3.3.1]nonyl (**2s**) and adamantyl (**2t**) groups, were efficiently synthesized. Importantly, the scope could be expanded to the tri-spiroether ring structures (**2u** and **2v**) with excellent levels of diastereoselectivity. Thus, the asymmetric C(sp^3^)–O bond formation method provides a versatile strategy for the synthesis of a variety of spirocyclic ether scaffolds.

**Table 2:** Substrate scope^*a*^

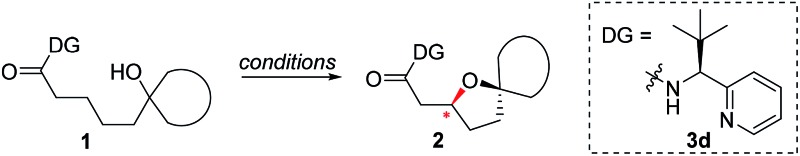		
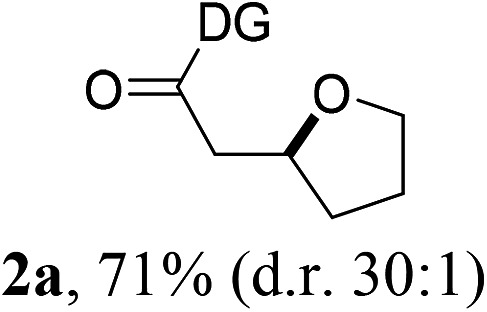	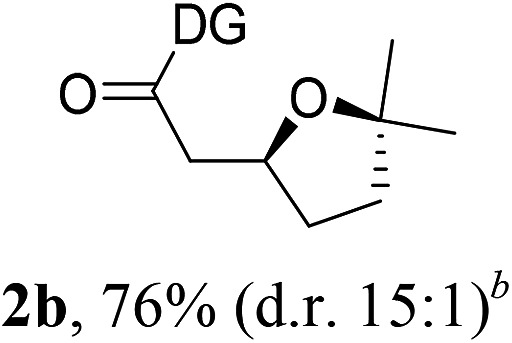	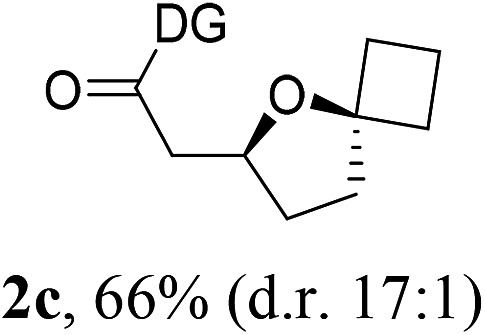
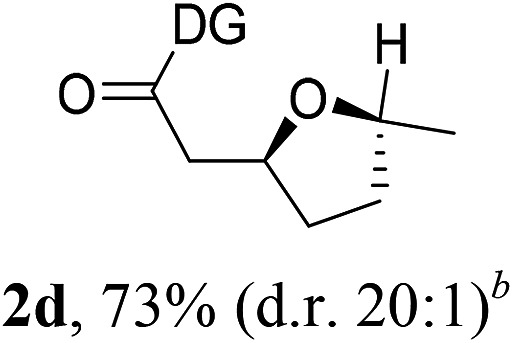	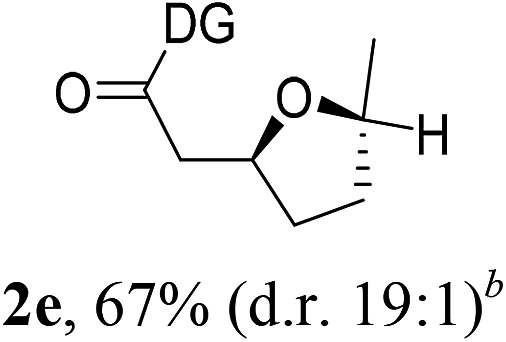	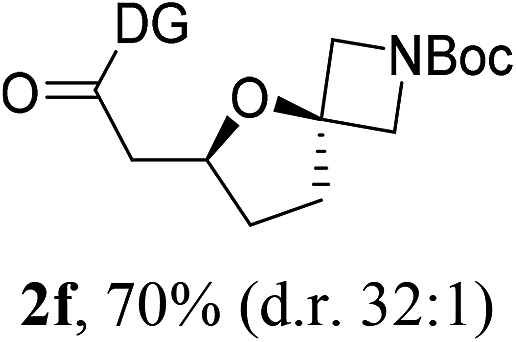
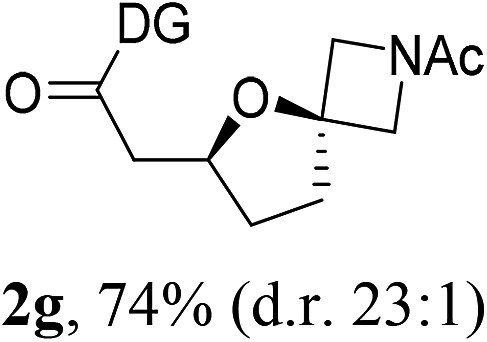	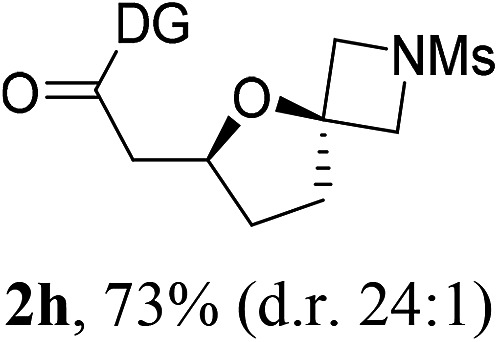	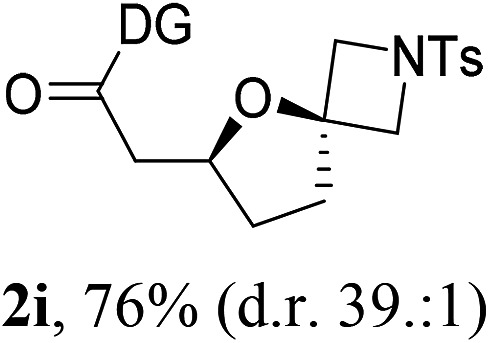
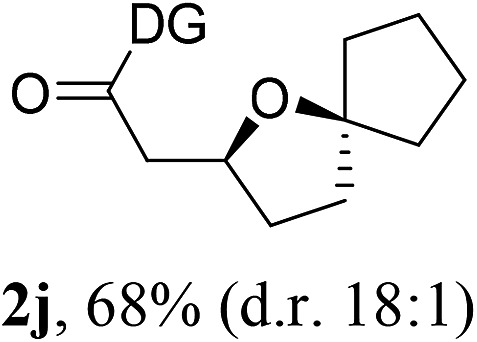	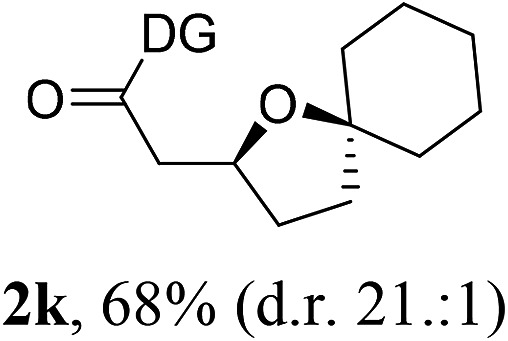	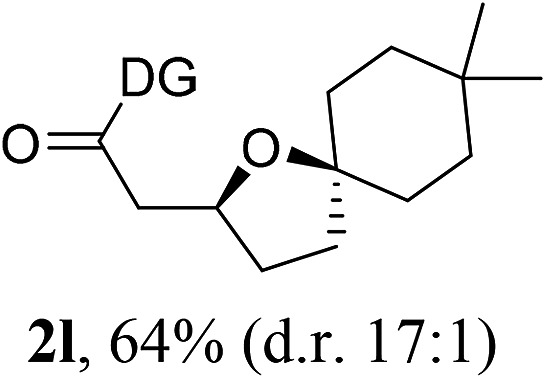
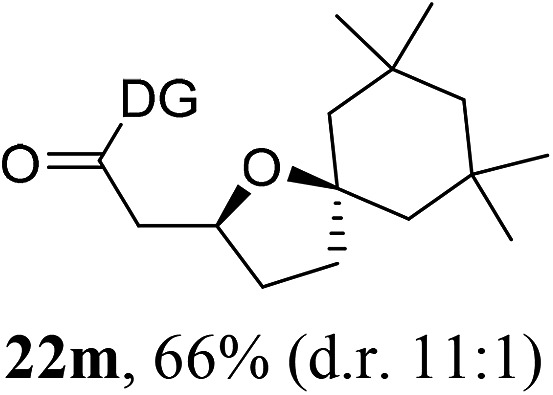	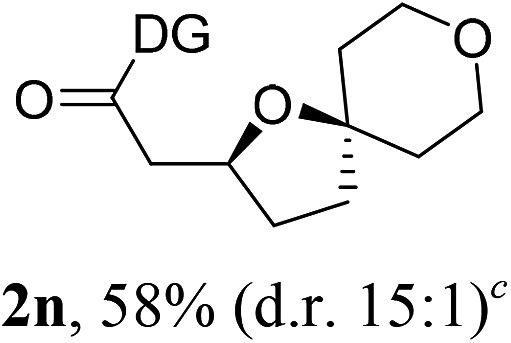	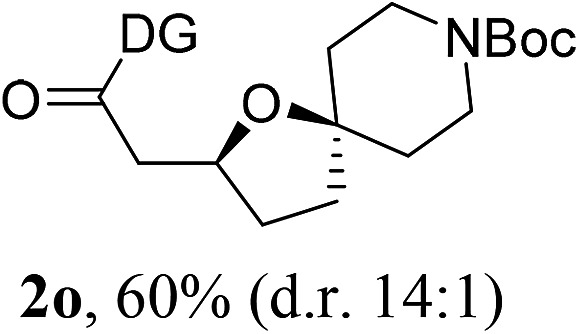
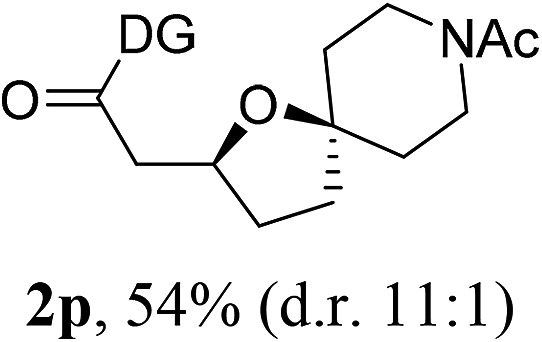	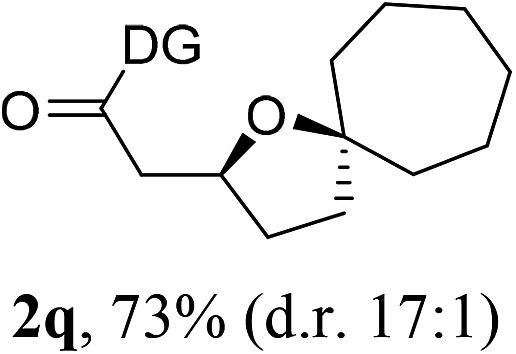	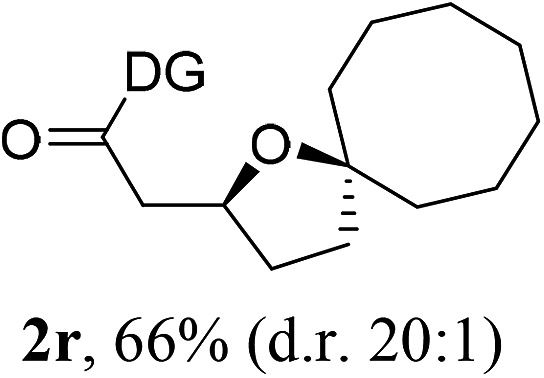
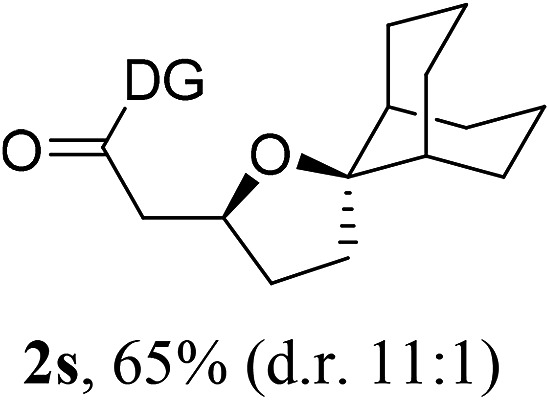		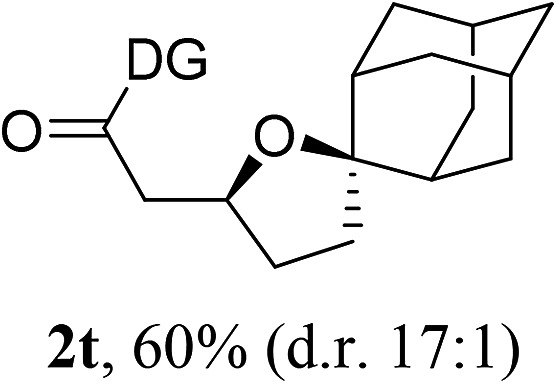
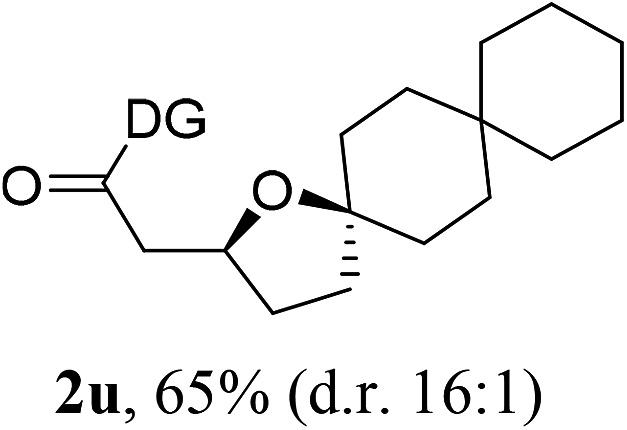		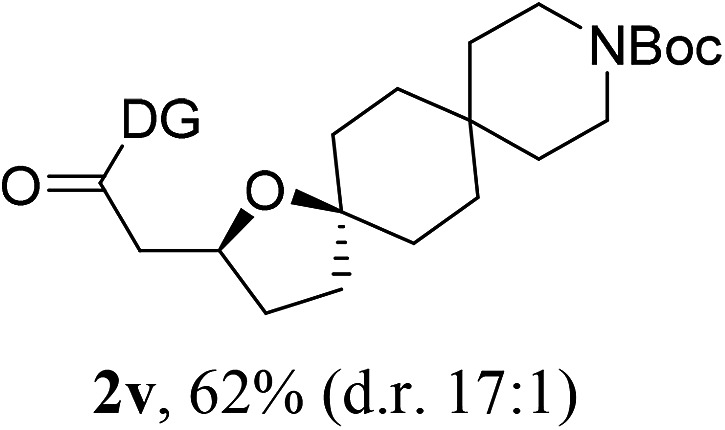

^*a*^Substrate (1.0 equiv.), Pd(OAc)_2_ (10 mol%), PhI(OAc)_2_ (3.0 equiv.), and AcOH (4.0 equiv.) in PhMe + EtOH (10 : 1) at 120 °C for 6–18 h. Isolated yields of products. The diastereoisomeric ratio (d.r.) was determined by HPLC analysis.

^*b*^The d.r. was determined by ^1^H NMR analysis.

^*c*^AcOH (8.0 equiv.) was used.

**Scheme 2:**
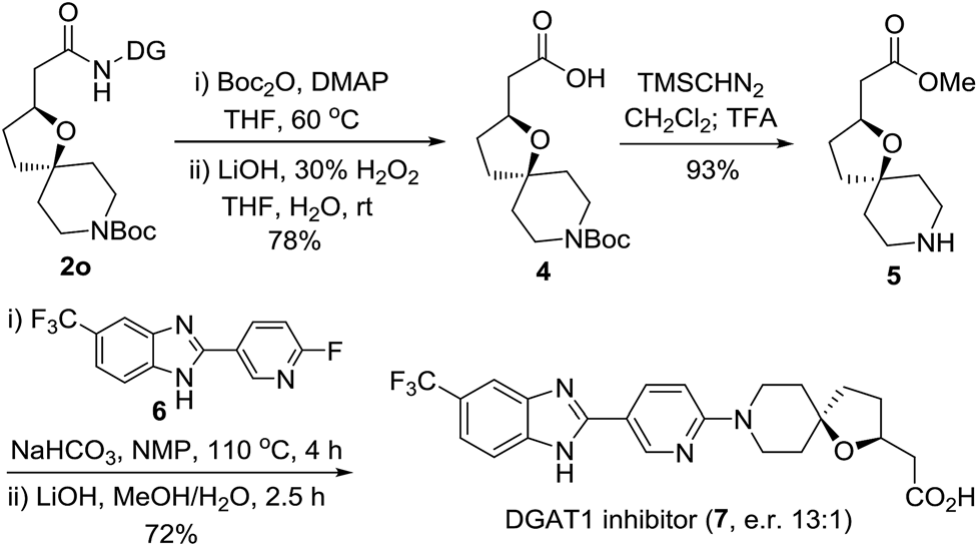
Application of the asymmetric synthesis of the DGAT1 inhibitor.

In order to demonstrate the synthetic utility of the current method, for the first asymmetric synthesis of the potent diacylglycerol acyltransferase (DGAT1) inhibitor (**7**), we treated **2o** as outlined in Scheme 2.^20^ Removal of the DG from **2o** gave the carboxylic acid **4**, which was readily converted to the intermediate **5** by esterification followed by *N*-Boc deprotection with TFA. S_*N*_Ar displacement of the pyridyl fluoride of **6** was subsequently executed with oxa-azaspirocyclic amine **5** by heating to 110 °C using NaHCO_3_ as the base in the solvent *N*-methyl-2-pyrrolidone (NMP). Finally, the corresponding DGAT1 inhibitor **7** (e.r. 13 : 1) was obtained by hydrolysis. This simple and efficient synthesis provides an excellent opportunity for exploring the derivatization strategies of this potent inhibitor bearing hindered oxaspirocyclic moieties.

**Scheme 3:**
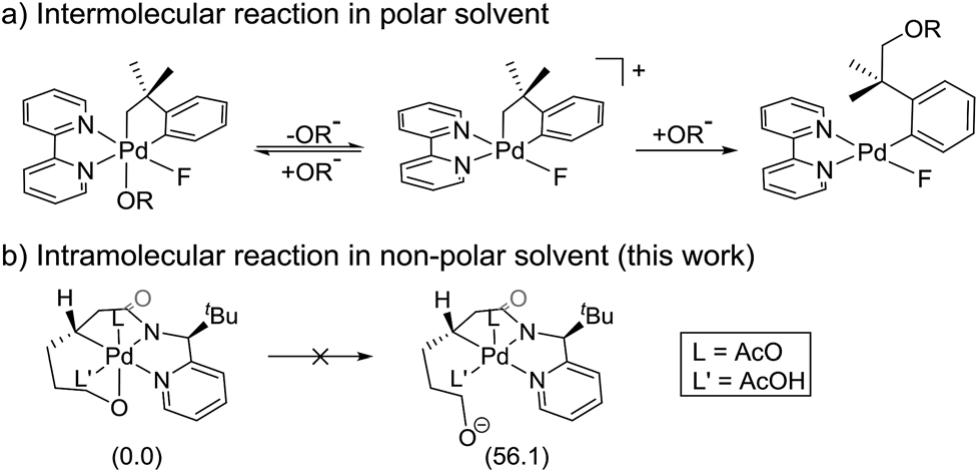
The proposed S_*N*_2-type reductive elimination mechanism.

Scheme 3a summarizes the mechanism of a Pd(iv) mediated C(sp^3^)–O coupling previously proposed by Sanford,^21^,^22^ which involves an S_*N*_2-type reductive elimination to form a 5-coordinate cationic Pd(iv) intermediate. In that case, invoking such an intermediate was reasonable, since (i) the relatively polar solvent acetonitrile effectively stabilizes the cationic intermediate, (ii) the release of the alkoxide is energetically favored due to the high solvation energy of the anionic leaving group and (iii) the increase in translational entropy due to the liberation of the alkoxide provides an additional driving force. The current system employs toluene, a non-polar solvent, which should substantially disfavor the elimination and there is no gain in translational entropy since the alkoxide is tethered, rendering the reaction unimolecular. Our computational models confirm that the S_*N*_2-type reductive elimination analogous to the Sanford proposal requires 56.1 kcal mol^–1^ in solution phase free energy (Scheme 3b).

**Fig. 2:**
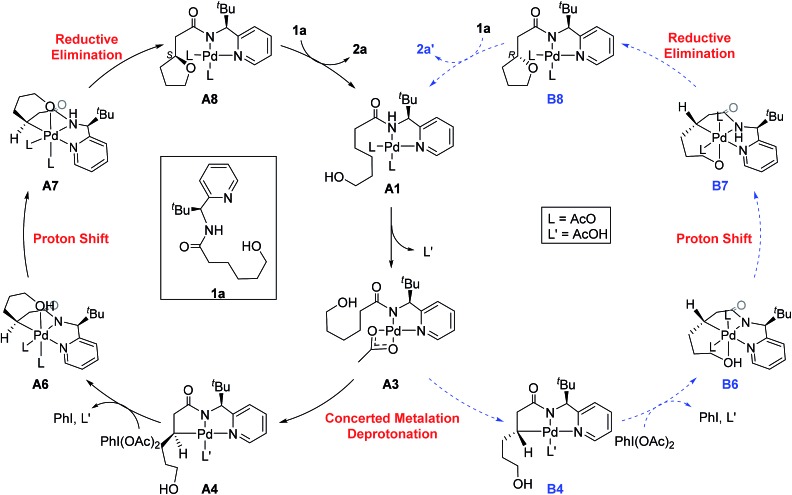
The proposed catalytic cycle of chiral bidentate directing group-mediated C(sp^3^)–O bond formation.

**Fig. 3:**
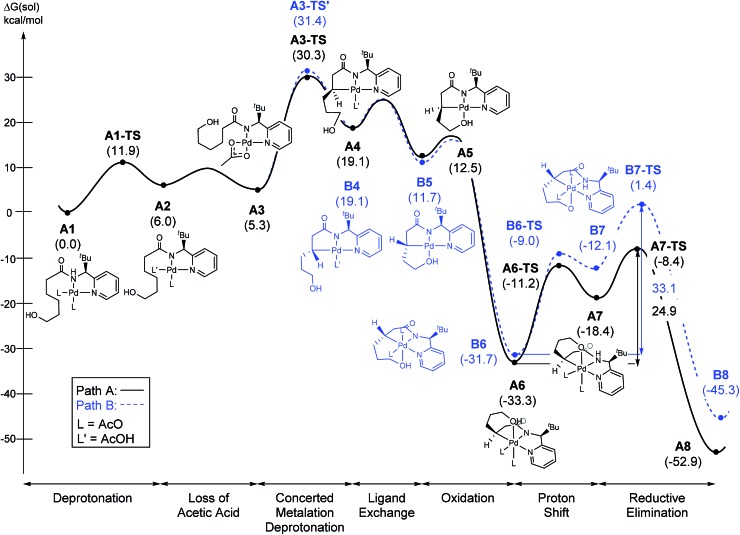
The energy profile of the proposed mechanism.

**Fig. 4:**
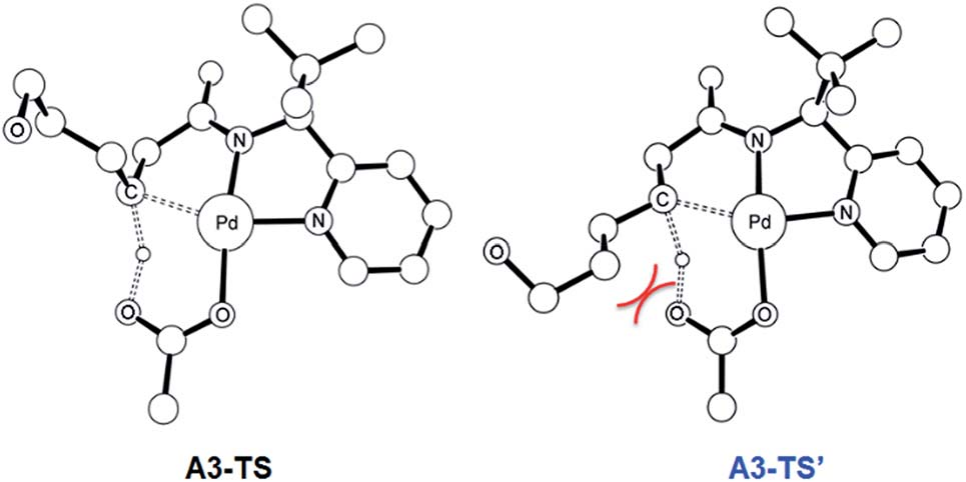
The DFT-optimized geometry of **A3-TS** (left) and **A3-TS′** (right). Nonessential hydrogen atoms are omitted for clarity.

The most probable catalytic mechanism according to our DFT calculations is shown in Fig. 2 and the reaction energy profile is given in Fig. 3 (optimized structures and vibrational frequencies were calculated using the B3LYP-D3/6-31G**/LACVP level of theory and triple-ζ basis sets, cc-pVTZ(-f)/LACV3P, were employed to get precise electronic energies. See the ESI† for full computational details). The catalytic cycle begins with the deprotonation of the DG in **A1** facilitated by one of the two acetate ligands bound to the Pd. After losing the acetic acid, the remaining acetate becomes bidentate to afford the Pd(ii)-intermediate **A3** at a relative energy of 5.3 kcal mol^–1^. This step is associated with a barrier of only 11.9 kcal mol^–1^ and is therefore expected to be easy. The Pd-center in the intermediate **A3** has the proper geometry to undergo a concerted metalation–deprotonation (CMD) reaction, where C–H bond activation takes place. Depending on the orientation of the pendant alcohol moiety in this CMD step, the two diastereomeric products **A4** and **B4** may be obtained. Pathway A gives the experimentally observed (*S*,*S*)-product traversing the transition state **A3-TS** at 30.3 kcal mol^–1^, whereas Pathway B affords the (*S*,*R*)-product and is associated with the transition state **A3-TS′**, which we located at 31.4 kcal mol^–1^. Fig. 4 illustrates the computed structures of these two transition states and a more detailed energy decomposition analysis indicated that the difference of 1.1 kcal mol^–1^ in the CMD barrier is due to the greater steric demand caused by the orientation of the alcohol pendant in **A3-TS′**.

At 120 °C a barrier difference of 1.1 kcal mol^–1^ should translate into a product ratio of roughly 4 : 1. Thus, if we assume that the observed diastereoselectivity is solely determined by this barrier difference, then the computationally predicted d.r. is notably smaller than the experimentally obtained d.r. of 26 : 1 by roughly one order of magnitude. We have carefully examined both transition states and searched for alternative saddle points on the potential energy surface that may offer better agreement with the experiment. The general observation in many unrelated but similar studies is that DFT calculations typically overestimate the barrier differences for reactions where significant d.r. are observed,^23^ which further suggests that the computed barrier difference is too small to explain the diastereoselectivity. After extensive exploration, we concluded that the barrier difference of 1.1 kcal mol^–1^ is the most reliable result for this step. This apparent disagreement between the computer model and experiment is satisfactorily resolved, however, as will be described below. In short, the predicted difference in the rate of reaction at this step is only partially responsible for the d.r. – there is a second process in the mechanism that leads to an additional enrichment of the d.r. in favor of the experimentally observed product. Irrespective of these energy considerations, one important conclusion can be drawn: our computed transition state structures illustrate that the orientation of the pendant alcohol is a plausible structural feature for determining the diastereoselectivity at the CMD step, giving rise to a meaningful energy difference between the two possible conformers.

**Fig. 5:**
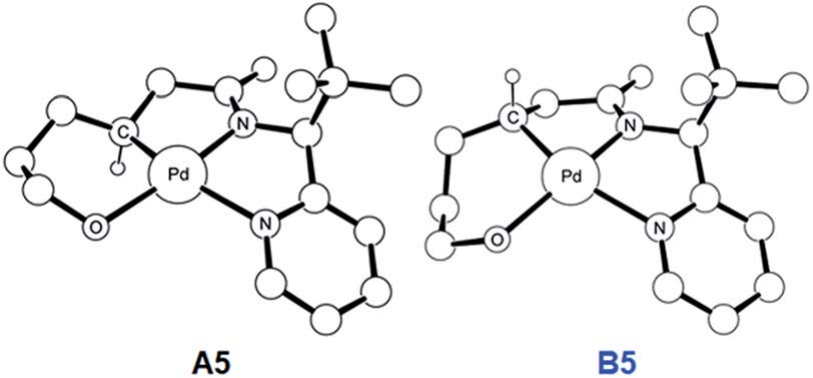
DFT-optimized geometry of **A5** (left) and **B5** (right). Nonessential hydrogen atoms are omitted for clarity.

To push the catalytic process forward, the intermediates **A4** and **B4** may lose an equivalent of acetic acid creating a vacant binding site on Pd that is utilized by the pendant alcohol moiety to complete a ligand exchange and form the transient intermediates **A5** and **B5**, which were located at 12.4 and 11.7 kcal mol^–1^, respectively. These two square planar Pd(ii) complexes can readily undergo chemical oxidation furnished by iodobenzene diacetate (PhI(OAc)_2_) to form the octahedral Pd(iv) complexes **A6** and **B6**, where two acetate ligands bind to Pd, one adopting an axial and the other an equatorial position. This oxidation step is computed to be exergonic by –33.3 and –31.7 kcal mol^–1^, respectively. At the given length of the alkyl-tether, consisting of three methylene moieties, the hydroxyl moiety prefers to bind in the axial position. Forcing it to bind in the equatorial position gives an energy penalty of ∼2 kcal mol^–1^ for both diastereomers (Table S4 and Scheme S8†). Interestingly, the stereochemistry of the alkyl-carbon bound to the Pd dictates whether the alcohol binds in a *syn* or *anti* disposition to the ^*t*^Bu-moiety: the alcohol pendant in **A5** can only form the *syn*-adduct, whereas **B5** can only form the *anti*-adduct, as highlighted in Fig. 5. This structural consequence of the stereochemical orientation of the Pd-alkyl fragment is chemically meaningful, as the energy demands for the next steps of the catalytic cycle are directly connected to the position of the hydroxyl. Specifically, the resting states **A6** and **B6** first engage in a proton shift where the proton from the hydroxyl group is moved to the Pd-bound amide ligand to give the transient intermediates **A7** and **B7**, respectively. The alkoxide-oxygen can now form a C–O bond in a reductive elimination step. Whereas the transition state for this product forming step **A7-TS** is found at –8.4 kcal mol^–1^ resulting in a barrier of 24.9 kcal mol^–1^, the analogous transition state for the other diastereomer, **B7-TS**, is located at 1.4 kcal mol^–1^ giving rise to a barrier of 33.1 kcal mol^–1^.

These dramatically different reductive elimination barriers will have a profound impact on the d.r. of the reaction. Whereas the barrier of 24.9 kcal mol^–1^ is decisively lower than the CMD barrier of 30.3 kcal mol^–1^ and we therefore do not anticipate any notable accumulation of **A6**, intermediate **B6** will accumulate and only turn over at a much slower rate, since the reductive elimination barrier of 33.1 kcal mol^–1^ is higher than the CMD barrier of 31.4 kcal mol^–1^ discussed above. Thus, in addition to the 4 : 1 selectivity anticipated in the CMD step, our calculations suggest a second kinetic resolution feature at this reductive elimination step, which we propose is the reason for the much higher d.r. value observed experimentally. The kinetic trapping of **B6** prevents the completion of the reductive elimination providing a rationale for the product yields of 60–75%.

**Fig. 6:**
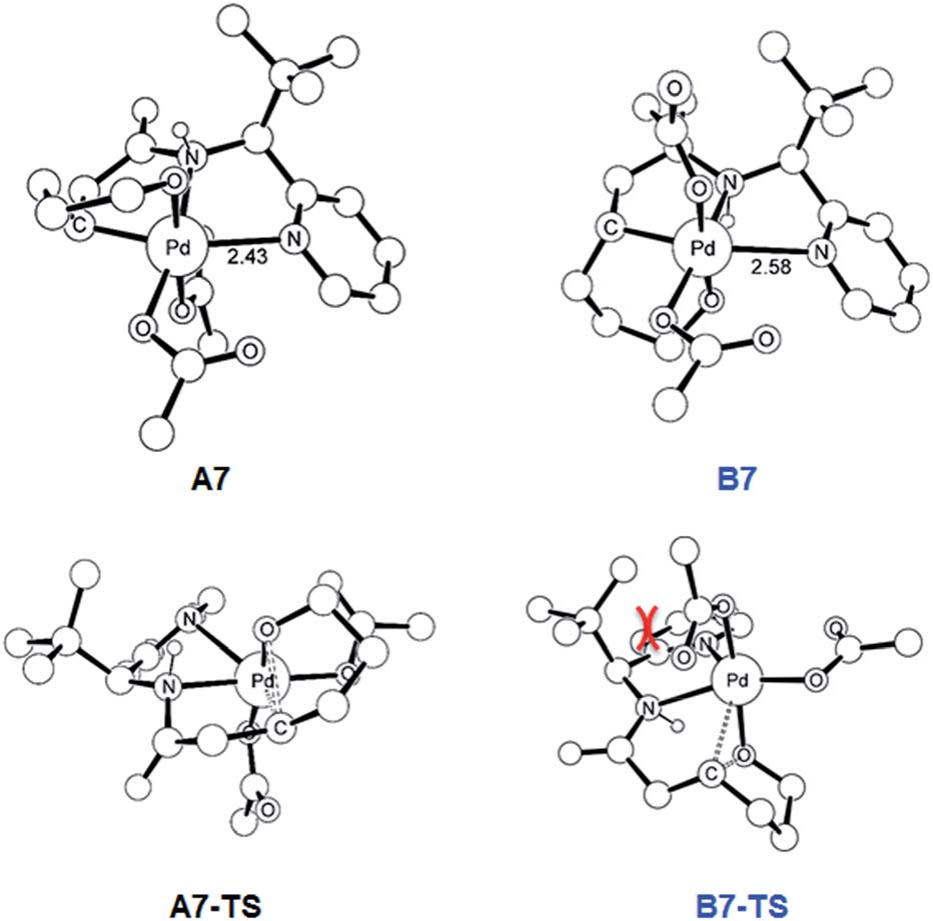
DFT-optimized geometry of **A7**/**B7** and **A7-TS**/**B7-TS** with selected distances in Å. Nonessential hydrogen atoms are omitted for clarity.

As illustrated in Fig. 3, the energetic divergence of the reductive elimination pathways for the two diastereomers culminating in an energy difference of 9.8 kcal mol^–1^ between **A7-TS** and **B7-TS** is visible already at the initial proton-shift step. The free energy of the intermediate **A7** is –18.4 kcal mol^–1^, which is more than 6 kcal mol^–1^ lower than its diastereomeric analogue **B7** at –12.1 kcal mol^–1^. A closer inspection of the molecular structures of these intermediates and transition states offers a simple explanation for this energy difference. The structures of **A7**/**B7** and **A7-TS**/**B7-TS** are compared in Fig. 6. The energy gap between **A7** and **B7** stems from structural distortions induced by the amide ligand upon protonation. Most notably, the puckering of the 5-membered palladacycle is determined by the relative arrangement of the ^*t*^Bu moiety and the (*S*/*R*)-amino-group leading to a much higher strain in the **B7** case. As a result, the Pd–N(pyridine) bond in intermediate **B7** is more extended at 2.58 Å, while the more stable intermediate **A7** shows a bond length of 2.43 Å. This structural preference for **A7** is maintained as the reductive elimination transition state is reached, but there is also an additional effect. As highlighted in Fig. 6, the reductive elimination goes hand in hand with a slight change in the bonding angle of the (*S*/*R*)-amino functionality that is needed to allow the C–O coupling to take place. In doing so, the ^*t*^Bu group can be extended away from the Pd-center in **A7-TS**, whereas the orientation of this sterically demanding group in **B7-TS** is such that an unfavorable clash between the ^*t*^Bu group and one of the acetate ligands cannot be avoided. Together, these two effects amount to the energy difference of 9.8 kcal mol^–1^.

In summary, our calculations suggest that the rate determining step should be the CMD reaction that is associated with a barrier of 30.3 kcal mol^–1^ for the major diastereomer. Interestingly, we found that the reductive elimination step enhances the diastereocontrol by only allowing the experimentally observed diastereomer to complete the reaction readily, whereas the other diastereomer is prevented from proceeding by a much higher barrier of 33.1 kcal mol^–1^. Experimentally, we found that under standard conditions a primary kinetic isotope effect (KIE) of 3.4 can be observed when the methylene-hydrogens are substituted with deuterium (eqn (1)). In accordance with this result, the predicted KIE value of the aforementioned CMD reaction is 3.6 (see the ESI† for details). When the KIE value difference of 0.2 is converted into the activation energy difference, it becomes ∼0.05 kcal mol^–1^ in the given conditions. Therefore, the KIE values prove that the cleavage of the methylene C–H bond is indeed rate limiting, as was suggested by our calculations.1
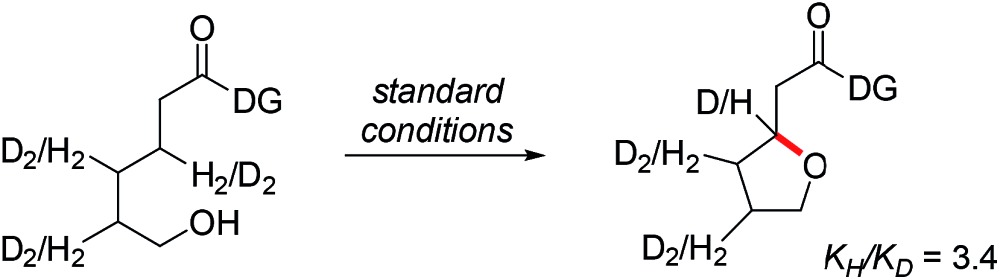



## Conclusions

We discovered a new, bidentate, chiral directing group derived from 2,2-dimethyl-1-(pyridin-2-yl)propan-1-amine, which enables the diastereoselective assembly of C(sp^3^)–O bonds using palladium(ii). Excellent selectivities were achieved for a variety of substrates, with diastereomeric ratios reaching 39 : 1. The utility of the present method was demonstrated by implementing a convenient asymmetric synthesis strategy for a wide range of oxaspirocycles, which are privileged scaffolds for biologically active molecules in medicinal chemistry. Furthermore, the new methodology was utilized to provide a concise stereoselective synthesis of a potent diacylglycerol acyltransferase (DGAT1) inhibitor. Lastly, a detailed mechanistic study based on DFT calculations revealed intriguing features of how the high stereoselectivity is achieved. Surprisingly, two different steps in the catalytic cycle were found to contribute to the kinetic resolution, namely, the concerted metalation–deprotonation step, which is proposed to be rate determining, and the reductive elimination step. This work constitutes the first example for stereoselective C–O bond formation *via* methylene C(sp^3^)–H bond activation.

## Conflicts of interest

There are no conflicts to declare.

## Supplementary Material

Supplementary information

Crystal structure data
