# Supplementary material for: Stereoselective construction of sterically hindered oxaspirocycles via chiral bidentate directing group-mediated C(sp3)–O bond formation
Source: Chem Sci. 2017 Nov 27;9(6):1473–80. doi: 10.1039/c7sc04691j (PMC5875089; doi:10.1039/c7sc04691j)
Supplement: Supplementary file 1 [file SC-009-C7SC04691J-s001.pdf]

Electronic Supplementary Material (ESI) for Chemical Science.
This journal is © The Royal Society of Chemistry 2017

Supporting Information

Stereoselective Construction of Sterically Hindered Oxaspirocycles via Chiral Bidentate Directing Group-Mediated Csp^3−O Bond Formation

Yechan Kim,[a,b,‖] Seoung-Tae Kim,[a,b,‖] Dahye Kang,[a,b] Te-ik Sohn,[a,b] Eunyoung Jang,[a,b] Mu-Hyun Baik,*[,b,a] and Sungwoo Hong*[,b,a]

[a]Department of Chemistry, Korea Advanced Institute of Science and Technology (KAIST), Daejeon, 34141, Korea

[b]Center for Catalytic Hydrocarbon Functionalizations, Institute for Basic Science (IBS), Daejeon 34141, Korea

I. General Methods and Materials S2

II. Experimental Procedure S3

III. Optimization Study S11

IV. Compound Characterizations S15

V. Computational Study S27

VI. Reference S85

Appendix I

Spectral Copies of ^{1}H- and ^{13}C-NMR Data Obtained in this Study S86

Appendix II

Crystallographic Data for 2a S114

I. General Methods and Materials.

Commercial grade reagents and solvents were used without further purification except as indicated below. Analytical thin layer chromatography (TLC) was performed on precoated silica gel 60 F^{254} plates and visualization on TLC was achieved by UV light (254 nm) and anisaldehyde solution, and heat as developing agents. Flash column chromatography was undertaken on silica gel (400-630 mesh). ^{1}H NMR was recorded on 400 MHz and chemical shifts were quoted in parts per million (ppm) referenced to the appropriate solvent peak or 2.50 ppm for DMSO-d_6. The following abbreviations were used to describe peak splitting patterns when appropriate: br = broad, s = singlet, d = doublet, t = triplet, q = quartet, m = multiplet, dd = doublet of doublet, td = triplet of doublet, ddd = doublet of doublet of doublet. Coupling constants, *J*, were reported in hertz unit (Hz). ^{13}C NMR was recorded on 151 MHz and was fully decoupled by broad band proton decoupling. Chemical shifts were reported in ppm referenced to the center line of a septet at 39.5 ppm of DMSO-d_6. Diastereomeric ratios were determined by integration of HPLC. Analytical HPLC was performed with an Agilent 1200 Series HPLC utilizing Poroschell 120 EC-C18 columns (4.6 × 50 mm or 4.6 × 150 mm) with visualization at 254 nm. The HPLC column was operated at 35 ºC. Mass spectral data were obtained from the KAIST Basic Science Institute by using ESI method.

II. Experimental Procedure

Scheme S1. General Procedure for Synthesis of Chiral Bidentate Directing Groups

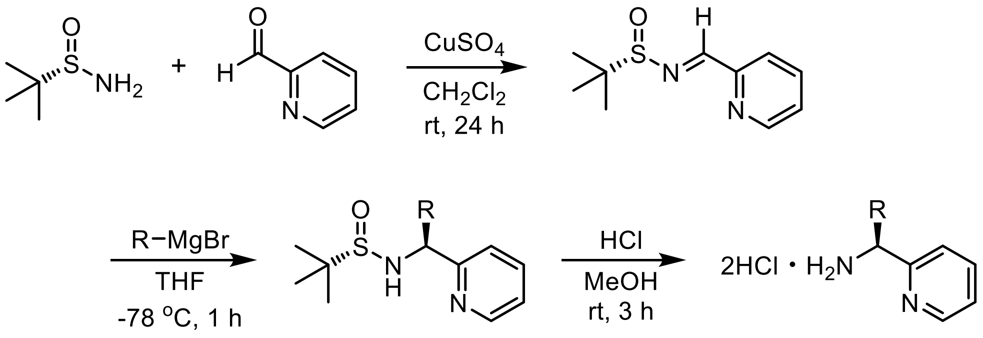

Synthesis of *N*-sulfinylimine.[1] To a solution of (*S*)-(-)-2-methyl-2-propanesulfinamide (5.0 g, 41.3 mmol) and flame-dried copper(II) sulfate (19.8 g, 123.9 mmol) in CH_2Cl_2 (80 mL) was added dropwise 2-pyridinecarboxaldehyde (4.0 g, 37.2 mmol) under argon atmosphere. The reaction was stirred at room temperature for 24 h. The reaction mixture was filtered through a pad of Celite and the filter cake washed with in CH_2Cl_2. After the removal of solvent, the residue was purified by flash column chromatography (CH_2Cl_2/EtOAc = 3:1) on silica gel to give the desired product as pale yellow oil. (4.9 g, 63% yield)

Synthesis of *N*-sulfinamide.[2] To a solution of *N*-sulfinylimine (4.9 g, 23.5 mmol) in THF (80 mL) at -78 °C was added dropwise alkylmagnesium chloride (35.3 mmol) under argon atmosphere. The reaction was stirred at -78 °C for 1 h. The reaction mixture was quenched by the addition of saturated aqueous NH_4Cl solution (50 mL) and extracted with EtOAc (3 × 100 mL). The combined organic layer was washed with brine (50 mL) and dried over $MgSO_4$. After the removal of solvent, the residue was purified by flash column chromatography (EtOAc/hexane= 1.5:1) on silica gel to give the crude product as yellow solid. The major diastereomer was separated by CombiFlash® Rf+ automated flash chromatography system (Redi*Sep*® Rf reversed phase C18 column, MeOH/H_2O).

Synthesis of amine dihydrochloride salt. To a solution of *N*-sulfinamide (15.0 mmol) in MeOH (50 mL) was added 2N HCl in diethyl ether (22.5 mL, 45.0 mmol). The reaction was stirred at room temperature for 3 h. After the removal of solvent, the residue was triturated with diethyl ether. The resulting precipitate was filtered, washed with diethyl ether, and dried under vacuum to give the desired product as white solid (quantitative yield).

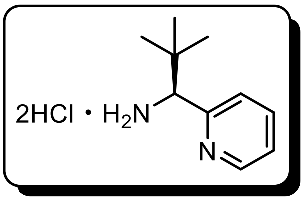

(*S*)-2,2-dimethyl-1-(pyridin-2-yl)propan-1-amine dihydrochloride. White solid. ^{1}H NMR (400 MHz, DMSO-d_6) δ 8.65 (ddd, *J* = 4.8, 1.8, 0.9 Hz, 1H), 8.33 (s, 3H), 7.88 (td, *J* = 7.8, 1.8 Hz, 1H), 7.49 (dt, *J* = 7.8, 1.1 Hz, 1H), 7.44 (ddd, *J* = 7.5, 4.8, 1.1 Hz, 1H), 4.68 (br, 1H), 4.24 (q, *J* = 5.7 Hz, 1H), 0.93 (s, 9H). ^{13}C NMR (151 MHz, DMSO-d_6) δ 153.0, 145.6, 140.6, 125.3, 125.0, 61.2, 34.4, 26.3. HRMS (ESI^{+}) m/z calcd. for $[C_{10}H_{17}N_2]^{+}$: 165.1386, found : 165.1390. e.e > 99%. The enantiomeric ratio was determined by HPLC analysis (after conversion to the acetamide by using AcCl and TEA) on Daicel Chiralcel OD-H, Et_2NH:Hexane:*i*PrOH = 0.1:95:5, flow rate 0.5 mL/min, λ = 254 nm, t_R= 10.6 min (*R*-isomer), 14.6 min (*S*-isomer).

Scheme S2. Procedure for Synthesis of 1a

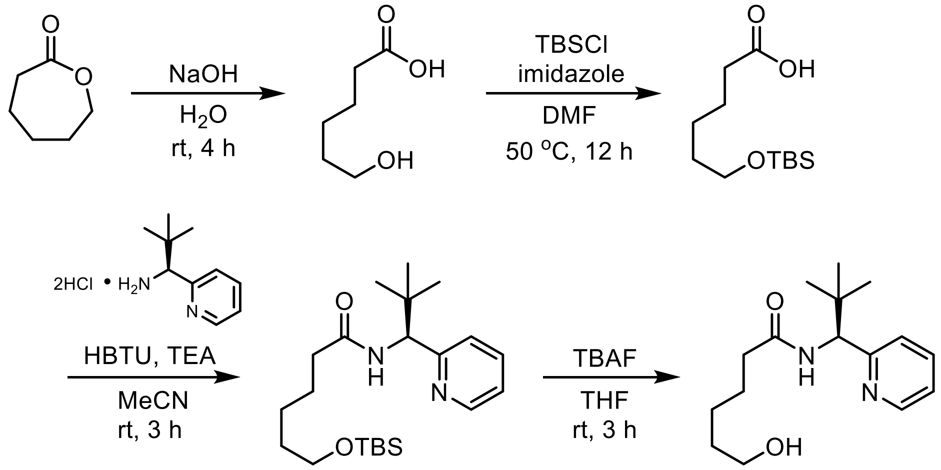

Lactone hydrolysis. To a solution of lactone (3.0 g, 26.3 mmol) in H_2O (100 mL) was added NaOH (2.1 g, 52.6 mmol). The reaction was stirred at room temperature for 4 h. The reaction mixture was acidified to pH 2.0 with 1N HCl aqueous solution and extracted with diethyl ether (3 × 50 mL). The combined organic layer was dried over $MgSO_4$ and concentrated to give the desired product as white solid (3.3 g, 95% yield).

TBS protection. To a solution of 6-hydroxyhexanoic acid (3.3 g, 25.0 mmol) and imidazole (3.7 g, 55.0 mmol) in DMF (25 mL) was added *tert*-butyldimethylsilyl chloride (4.1 g, 27.5 mmol) under argon atmosphere. The reaction was stirred at 50 °C for 12 h. The reaction mixture was diluted with diethyl ether (3 × 100 mL) and washed with brine (50 mL). The combined organic layer was dried over $MgSO_4$. After the removal of solvent, the residue was purified by flash column chromatography

(EtOAc/hexane= 1:2) on silica gel to give the desired product as colorless oil (4.9 g, 80% yield).

Amide bond formation. To a solution of 6-((*tert*-butyldimethylsilyl)oxy)hexanoic acid (2.0 g, 8.1 mmol) and amine dihydrochloride salt (1.9 g, 8.1 mmol) in acetonitrile (40 mL) was added HBTU (3.2 g, 8.5 mmol) and trimethylamine (4.0 mL, 28.4 mmol). The reaction was stirred at room temperature for 3 h. The solvent was evaporated under vacuum. The reaction mixture was diluted with diethyl ether (3 × 50 mL) and washed with brine (50 mL). The combined organic layer was washed with saturated aqueous $NaHCO_3$ solution (3 × 50 mL) and dried over $MgSO_4$. After the removal of solvent, the residue was purified by flash column chromatography (EtOAc/hexane= 1:2) on silica gel to give the desired product as pale yellow oil (2.3 g, 73% yield).

TBS deprotection. To a solution of (*S*)-6-((*tert*-butyldimethylsilyl)oxy)-*N*-(2,2-dimethyl-1-(pyridin-2-yl)propyl)hexanamide (2.3 g, 5.9 mmol) in THF (5 mL) was added tetrabutylammonium fluoride solution (1.0 M in THF, 17.6 mL, 17.6 mmol) under argon atmosphere. The reaction was stirred at room temperature for 5 h. The reaction mixture was diluted with CH_2Cl_2 (3 × 50 mL) and washed with saturated aqueous $NaHCO_3$ solution (50 mL). The combined organic layer was dried over $MgSO_4$. After the removal of solvent, the residue was purified by flash column chromatography (acetone/hexane= 1:1) on silica gel to give the desired product **1a** as white amorphous solid (1.4 g, 87% yield).

Scheme S3. Procedure for Synthesis of substrates

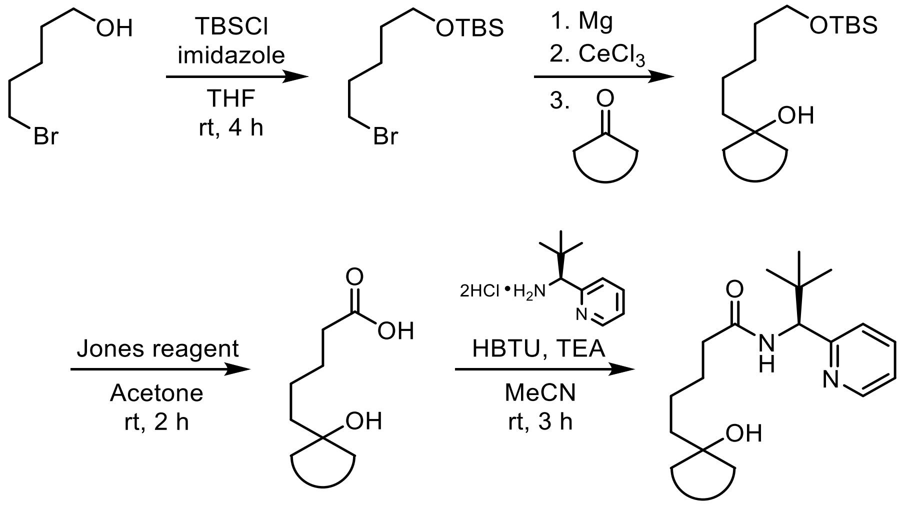

TBS protection. To a solution of imidazole (4.9 g, 71.8 mmol) in THF (60 mL) was added 5-bromopentan-1-ol (6.0 g, 35.9 mmol) and *tert*-butyldimethylsilyl chloride (6.5 g, 43.1 mmol) under argon atmosphere. The reaction was stirred at room temperature for 4 h. The reaction mixture was diluted with diethyl ether (3 × 70 mL) and washed with H_2O (70 mL). The combined organic layer was dried over $MgSO_4$. After the removal of solvent, the residue was purified by flash column chromatography (EtOAc/hexane= 1:10) on silica gel to give the desired product as colorless oil (8.1 g, 80% yield).

1,2-addition of cyclic ketone.[3] To a suspension of magnesium (0.3 g, 13.2 mmol) in diethyl ether (10 mL) was added ((5-bromopentyl)oxy)(*tert*-butyl)dimethylsilane (1.2 g, 4.4 mmol) in diethyl ether (5 mL) under argon atmosphere. The reaction was stirred at room temperature for 1 h to afford a solution of Grignard reagent. A suspension of flame-dried $CeCl_3$ (1.3 g, 5.3 mmol) in THF (10 mL) was stirred at room temperature for 3 h. To a suspension of $CeCl_3$ in THF was added dropwise a solution of Grignard reagent at 0 ºC. The reaction was stirred at 0 ºC for 1 h before a solution of cyclic ketone (1.8 mmol) in THF (3 mL) was added. After 1 h at 0 °C, the reaction mixture was quenched by the addition of H_2O (1 mL). The reaction mixture was filtered through a pad of Celite and Na_2SO_4 and the filter cake washed with in EtOAc. After the removal of solvent, the residue was purified by flash column chromatography on silica gel to give the desired product.

Oxidation.[4] To a solution of silyl ether (1.6 mmol) in acetone (20 mL) was added dropwise Jones reagent (2.0 M CrO_3 in aqueous H_2SO_4, 3.3 mmol) at -10 ºC. The reaction was stirred at room temperature for 2 h. The reaction mixture was quenched by the addition of 2-propanol (5 mL). The reaction mixture was filtered through a pad of Celite and the filter cake washed with in acetone. After the removal of solvent, the residue diluted with saturated aqueous $NaHCO_3$ solution (3 × 30 mL) and washed with diethyl ether (30 mL). The aqueous layer was acidified to pH 2.0 with 1N HCl aqueous solution at 0 ºC and extracted with EtOAc (3 × 50 mL). The combined organic layer was dried over $MgSO_4$ and concentrated to give the desired product.

Amide bond formation. To a solution of hydroxycarboxylic acid (1.5 mmol) and amine dihydrochloride salt (0.36 g, 1.5 mmol) in acetonitrile (6 mL) was added HBTU (0.6 g, 1.6 mmol) and trimethylamine (0.73 mL, 5.3 mmol). The reaction was stirred at room temperature for 3 h. The solvent was evaporated under vacuum. The reaction mixture was diluted with diethyl ether (3 × 50 mL) and washed with brine (50 mL). The combined organic layer was washed with saturated aqueous $NaHCO_3$ solution (3 × 50 mL) and dried over $MgSO_4$. After the removal of solvent, the residue was purified by flash column chromatography (acetone/hexane) on silica gel to give the desired product.

Scheme S4. Procedure for Synthesis of substrates 1d and 1e.

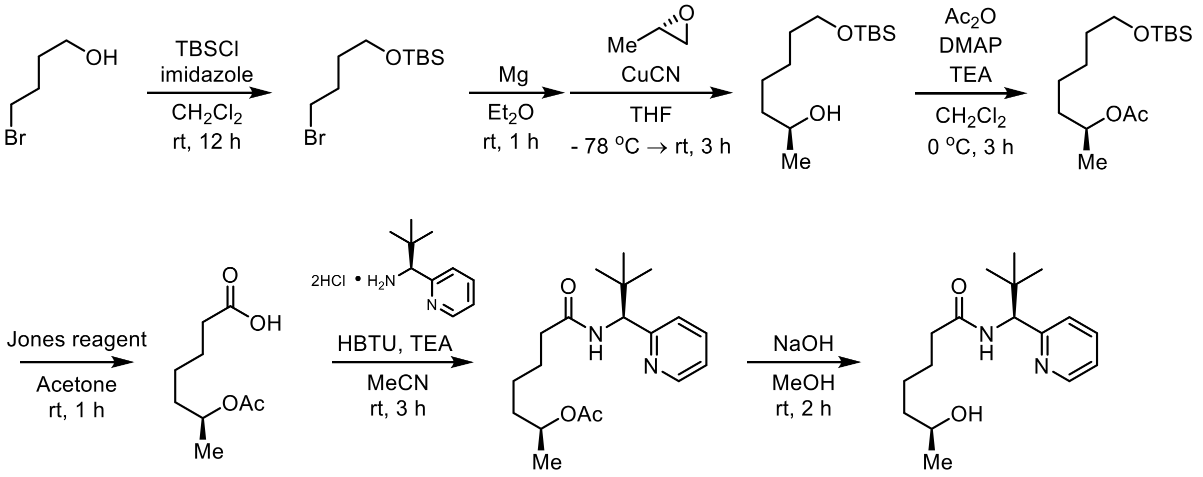

TBS protection. To a solution of imidazole (681 mg, 10.0 mmol) in CH_2Cl_2 (30 mL) was added 4-bromobutan-1-ol (1.0 g, 6.7 mmol) and *tert*-butyldimethylsilyl chloride (1.2 g, 8.0 mmol) under argon atmosphere. The reaction was stirred at room temperature for 12 h. The reaction mixture was diluted with diethyl ether (3 × 70 mL) and washed with H_2O (70 mL). The combined organic layer was dried over $MgSO_4$. After the removal of solvent, the residue was purified by flash column chromatography (EtOAc/hexane= 1:10) on silica gel to give the desired product as colorless oil (1.5 *g*, 84*%* yield).

Grignard reaction. To a suspension of magnesium (137 mg, 5.6 mmol) in diethyl ether (3 mL) was added (4-bromobutoxy)(*tert*-butyl)dimethylsilane (600 mg, 2.2 mmol) in diethyl ether (2 mL) under argon atmosphere. The reaction was stirred at room temperature for 1 h to afford a solution of Grignard reagent. To a suspension of CuCN (9.1 mg, 0.1 mmol) in THF (2 mL) was added (*S*)-(-)-propylene oxide (118 mg, 2.0 mmol, (*R*)-(+)-propylene oxide for synthesis of **1d**) at -78 °C. To a suspension of CuCN and (*S*)-(-)-propylene oxide in THF was added dropwise a solution of Grignard reagent at -78 °C. After 3 h at -78 °C, the reaction mixture was quenched by the addition of aqueous NH_4Cl solution (2 mL). The reaction mixture was diluted with diethyl ether (3 × 20 mL) and washed with H_2O (20 mL). The combined organic layer was dried over $MgSO_4$. After the removal of solvent, the residue was purified by flash column chromatography (EtOAc/hexane = 1:5) on silica gel to give the desired product (382 mg, 76*%* yield).

Acetyl protection. To a solution of alcohol (249 mg, 1.0 mmol), DMAP (24.4 mg, 0.2 mmol), and triethylamine (0.7 mL, 5.1 mmol) in CH_2Cl_2 (10 mL) was added dropwise acetic anhydride (0.2 mL, 2.0 mmol) at 0 °C. The reaction was stirred at 0 °C for 3 h. After the removal of solvent, the reaction mixture was diluted with EtOAc (3 × 20 mL) and washed with saturated aqueous $NaHCO_3$ (20 mL). The combined organic layer was dried over $MgSO_4$. After the removal of solvent, the residue was purified by flash column chromatography (EtOAc/hexane = 1:7) on silica gel to give the desired product (273 m*g,* 94*%* yield*).*

Oxidation. To a solution of silyl ether (273 mg, 0.95 mmol) in acetone (18 mL) was added dropwise

Jones reagent (2.0 M CrO_3 in aqueous H_2SO_4, 2.0 mmol) at -10 °C. The reaction was stirred at room temperature for 2 h. The reaction mixture was quenched by the addition of 2-propanol (5 mL). The reaction mixture was filtered through a pad of Celite and the filter cake washed with in acetone. After the removal of solvent, the residue diluted with saturated aqueous $NaHCO_3$ solution (3 × 20 mL) and washed with diethyl ether (20 mL). The aqueous layer was acidified to pH 2.0 with 1N HCl aqueous solution at 0 °C and extracted with EtOAc (3 × 30 mL). The combined organic layer was dried over $MgSO_4$ and concentrated to give the desired product (169 mg, 94% yield).

Amide bond formation. To a solution of carboxylic acid (97 mg, 0.51 mmol) and amine dihydrochloride salt (122 mg, 0.51 mmol) in acetonitrile (3 mL) was added HBTU (203 mg, 0.54 mmol) and triethylamine (0.25 mL, 1.8 mmol). The reaction was stirred at room temperature for 3 h. The solvent was evaporated under vacuum. The reaction mixture was diluted with diethyl ether (3 × 30 mL) and washed with brine (20 mL). The combined organic layer was washed with saturated aqueous $NaHCO_3$ solution (3 × 20 mL) and dried over $MgSO_4$. After the removal of solvent, the residue was purified by flash column chromatography (EtOAc/hexane = 1:1) on silica gel to give the desired product (142 mg, 83% yield).

Acetyl deprotection. To a solution of acetate (142 mg, 0.42 mmol) in MeOH (1 mL) was added NaOH (2.0 M in H_2O, 0.42 mL) at room temperature. The reaction was stirred at room temperature for 2 h. The reaction mixture was diluted with CH_2Cl_2 (3 × 20 mL) and washed with brine (20 mL). The combined organic layer was dried over $MgSO_4$. After the removal of solvent, the residue was purified by flash column chromatography (acetone/hexane= 1:1) on silica gel to give the desired product. (122 mg, quantitative yield).

Scheme S5. General Procedure for Stereoselective Intramolecular C(sp³)–O Bond Formation

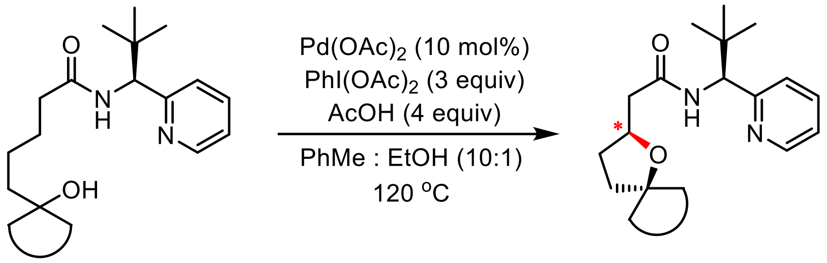

Substrate (0.1 mmol), $Pd(OAc)_2$ (2.2 mg, 10 mol%), $PhI(OAc)_2$ (96.6 mg, 3.0 equiv), and AcOH (24.0 mg, 4.0 equiv) were combined in PhMe and EtOH (10 : 1, 1.0 mL) under argon atmosphere (balloon). The reaction was stirred at 120 °C for 6 – 18 h. The mixture was monitored by TLC using acetone/hexane (1:3) as the mobile phase. The reaction mixture was diluted with CH_2Cl_2 (3 × 25 mL) and washed with saturated aqueous $NaHCO_3$ solution (30 mL).

Determining Diastereomeric Ratios: The organic layer was dried over $MgSO_4$ and filtered through a

short column chromatography on silica gel. The crude mixtures were collected and subjected for the determination of diastereomeric ratio by HPLC analysis (EC-C18 column, 4.6 × 50 mm or 4.6 × 150 mm, λ = 254 nm).

Purification: After the removal of solvent, the residue was purified by flash column chromatography (acetone/hexane or CH_2Cl_2/MeOH, indicated below) on silica gel to give the desired product.

Scheme S6. Procedure for Cleavage of Chiral Bidentate Auxiliary

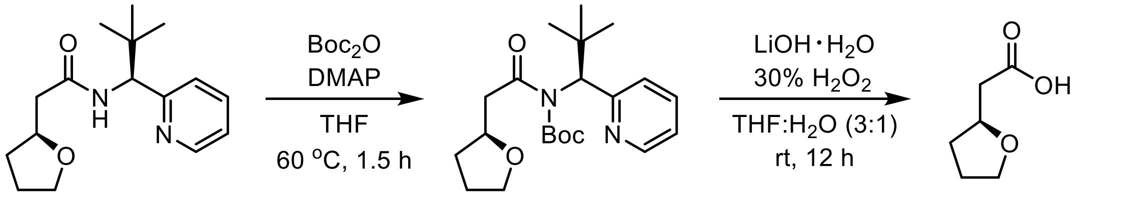

2a (d.r = 96.8 : 3.2) (e.r = 96.7 : 3.3)

To a solution of **2a** (33.2 mg, 0.12 mmol) and DMAP (22.0 mg, 0.18 mmol) in THF (1.5 mL) was added dropwise Boc_2O (0.17 mL, 0.72 mmol) under argon atmosphere. The reaction was stirred at 80 °C for 1.5 h. After the removal of solvent, the residue was purified by flash column chromatography (acetone/hexane= 1:3) on silica gel to give the desired product as yellow solid (43.9 mg, 97% yield). To a solution of N-Boc imide (43.9 mg, 0.117 mmol) and 30% H_2O_2 (0.27 mL) in THF/H_2O (3:1, 1.2 mL) was added LiOH·H_2O (48.9 mg, 1.17 mmol) at 0 °C. The reaction was stirred at room temperature for 12 h and quenched by the addition of saturated aqueous Na_2SO_3 solution. The reaction mixture was basified with 1N NaOH aqueous solution and washed with EtOAc. The aqueous layer was acidified to pH 2.0 with 1N HCl aqueous solution and extracted with EtOAc (3 × 20 mL). The combined organic layer was dried over $MgSO_4$. After the removal of solvent, the residue was purified by flash column chromatography (DCM/MeOH= 12:1) on silica gel to give the desired product as colorless oil (10.8 mg, 71% yield, e.r = 96.7:3.3). The enantiomeric ratio was determined by HPLC analysis (after conversion to the benzyl ester by using BnOH, DMAP, and DCC) on Daicel Chiralcel OJ-H, Hexane:*i*PrOH = 80:20, flow rate 1.0 mL/min, λ = 254 nm, t_R= 7.7 min (minor), 8.2 min (major).[5]

Procedure for Synthesis of DGAT inhibitor 6.

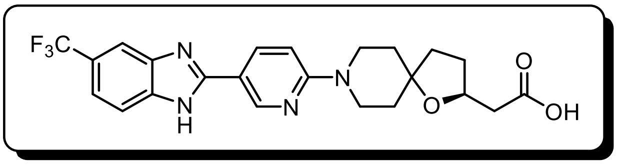

Cleavage of chiral bidentate auxiliary. To a solution of **2o** (116 mg, 0.26 mmol) and DMAP (47 mg, 0.40 mmol) in THF (4.0 mL) was added dropwise Boc_2O (0.36 mL, 1.55 mmol) under argon atmosphere. The reaction was stirred at 80 °C for 5 h. After the removal of solvent, the residue was purified by flash

column chromatography (EtOAc/hexanes = 1:4) on silica gel to give the desired product**.** To a solution of N-Boc imide and 30% H_2O_2 (0.7 mL) in THF + H_2O (3:1, 3.0 mL) was added LiOH·H_2O (107 mg, 2.6 mmol) at 0 ºC. The reaction was stirred at room temperature for 12 h and quenched by the addition of saturated aqueous Na_2SO_3 solution. The reaction mixture was basified with 1N NaOH aqueous solution and washed with EtOAc. The aqueous layer was acidified to pH 2.0 with 1N HCl aqueous solution and extracted with EtOAc (3 × 20 mL). The combined organic layer was dried over $MgSO_4$. After the removal of solvent, the residue was purified by flash column chromatography (CH_2Cl_2/MeOH = 15:1) on silica gel to give the desired product as colorless oil (61 mg, 78%, overall yield).

Synthesis of (*S*)-2-(8-(5-(5-(trifluoromethyl)-1*H*-benzo[*d*]imidazol-2-yl)pyridin-2-yl)-1-oxa-8-azaspiro[4.5]decan-2-yl)acetic acid (6). To a solution of *N*-Boc protected acid (30.3 mg, 0.10 mmol) in CH_2Cl_2 (1 mL) was added dropwise trimethylsilyldiazomethane (2.0 M in hexanes, 0.13 mL) at 0 ºC. The reaction was stirred at 0 ºC for 3 h. The reaction mixture was diluted with CH_2Cl_2 (3 × 10 mL) and washed with H_2O (6 mL). The combined organic layer was dried over $MgSO_4$. After the removal of solvent, the residue was purified by flash column chromatography (EtOAc/hexane = 1:3) on silica gel to give the desired product as yellow oil (29.1 mg, 93% yield). To a solution of *N*-Boc protected methyl ester (15.2 mg, 0.048 mmol) in CH_2Cl_2 (0.5 mL) was added trifluoroacetic acid (0.15 mL) at room temperature. The reaction was stirred at room temperature for 1 h. After the removal of solvent, the brown residue was directly used for the next step without further purification. To a solution of TFA amine salt (15.6 mg, 0.048 mmol) in NMP (0.5 mL) was added 2-(6-fluoropyridin-3-yl)-5-(trifluoromethyl)-1*H*-benzo[*d*]imidazole (13.4 mg, 0.048 mmol) and $NaHCO_3$ (40.3 mg, 0.48 mmol) at room temperature. The reaction was stirred at 110 ºC for 4 h. The reaction mixture was diluted with EtOAc (3 × 10 mL) and washed with brine (10 mL). The combined organic layer was dried over $MgSO_4$. After the removal of solvent, the residue was purified by flash column chromatography (acetone/hexane = 1:2) on silica gel to give the desired product as light yellow solid (18.7 mg, 82% yield). To a solution of methyl ester (17.5 mg, 0.037 mmol) in THF, H_2O, and MeOH (1:1:1, 0.5 mL) was added LiOH·H_2O (7.7 mg, 0.19 mmol) at room temperature. The reaction was stirred at room temperature for 2.5 h. The reaction mixture was acidified to pH 2.0 with 1N HCl aqueous solution and extracted with EtOAc (3 × 40 mL). The combined organic layer was dried over $MgSO_4$. After the removal of solvent, the residue was purified by flash column chromatography (CH_2Cl_2/MeOH = 15:1 to 5:1) on silica gel to give the desired product as white solid (14.8 mg, 88% yield). ^{1}H NMR (400 MHz, Methanol-d_4) δ 8.82 (d, J = 2.6 Hz, 1H), 8.17 (dd, J = 9.1, 2.6 Hz, 1H), 7.84 (s, 1H), 7.68 (d, J = 8.4 Hz, 1H), 7.51 (d, J = 8.4 Hz, 1H), 6.96 (d, J = 9.1 Hz, 1H), 4.40 (p, J = 6.9 Hz, 1H), 3.95 – 3.84 (m, 2H), 3.72 – 3.61 (m, 2H), 2.57 (dd, J = 15.0, 6.9 Hz, 1H), 2.50 (dd, J = 15.0, 6.9 Hz, 1H), 2.24 – 2.14 (m, 1H), 1.93 – 1.82 (m, 2H), 1.82 – 1.63 (m, 5H). HRMS (ESI$^+$) m/z calcd. for $[C_{23}H_{24}F_3N_4O_3]^+$: 461.1795, found : 461.1800. e.r = 13:1. The enantiomeric ratio was determined by HPLC analysis on Daicel Chiralpak IA, hexanes:EtOH = 65:35, flow rate 0.5 mL/min, λ = 254 nm, t_R= 10.9 min ((*S*)-isomer), 13.0 min ((*R*)-isomer).

III. Optimization Study

Table S1. Additive Screening[a]

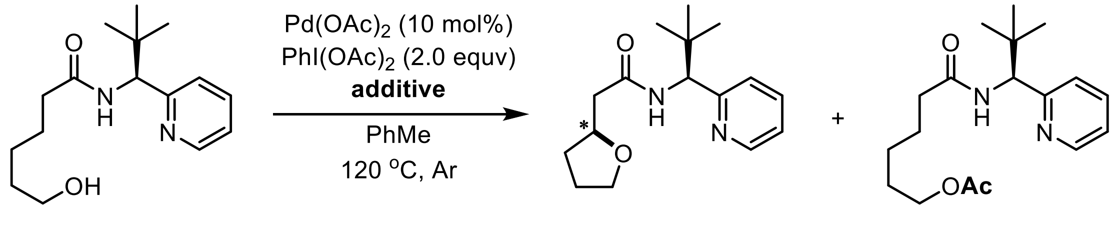

1a **2a** **2a'**

entry	additive (equiv)	**2a** % (d.r.)	**2a'** %
1	-	46 (8.3 : 1)	
2	AcOH (0.2)	41 (8.1 : 1)	12
3	AcOH (1)	47 (10.4 :1)	11
4	AcOH (2)	49 (14.3 : 1)	11
5	AcOH (4)	63 (26.1 : 1)	11
6	AcOH (8)	61 (32.2 :1)	11
7	AcOH (20)	51 (33.1 : 1)	12
8	AcOH (50)	33	23
9	PivOH (4)	58 (23 : 1)	8
10	$(BnO)_2PO_2H$ (2)	NR	
11	Ac-Gly-OH (2)	35	11
12	$TsOH \cdot H_2O$ (2)	trace	
13	Ag_2CO_3 (2)	33 (5.8 : 1)	6
14	AgOAc (2)	39 (6.7 : 1)	16
15	LiOAc (2)	35 (7.5 : 1)	25

[a] Conditions: Substrate (0.1 mmol), $Pd(OAc)_2$ (10 mol%), $PhI(OAc)_2$ (2.0 equiv), additive in PhMe (1.0 mL) at 120 °C for 12 h. The d.r. was determined by HPLC analysis. NR = no reaction

Table S2. Oxidant Screening[a]

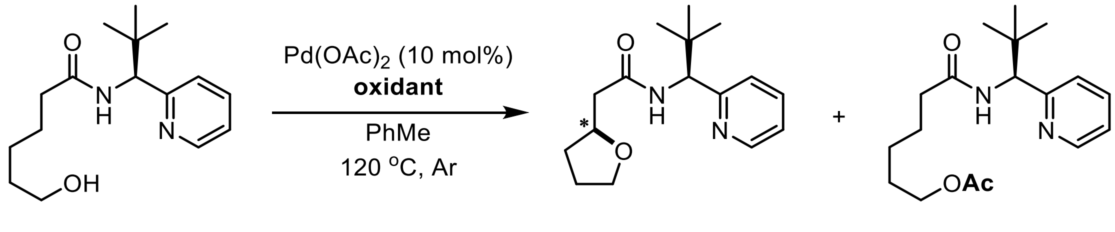

1a **2a** **2a'**

entry	oxidant (equiv)	**2a** % (d.r.)	**2a'** %

1	$PhI(OAc)_2$ (2)	46 (8.3 : 1)	16
2	$PhI(OPiv)_2$ (2)	39	10
3	$PhI(TFA)_2$ (2)	NR	
4	PhITs(OH) (2)	NR	
5	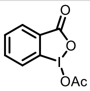 (2)	28	25
6	oxone (2)	NR	
7	$K_2S_2O_8$ (2)	NR	
8	$NaIO_4$ (2)	NR	
9	$NaIO_4$ (2)	NR	
10	$NaIO_4$ (2) / Ac_2O (10)	NR	40
11	DMP (2)	NR	
12	DDQ (2)	NR	
13	Selectfluor (2)	NR	
14	$Ce(SO_4)_2$ (2)	NR	

[a] Conditions: Substrate (0.1 mmol), $Pd(OAc)_2$ (10 mol%), oxidant in PhMe (1.0 mL) at 120 ºC for 12 h. The d.r. was determined by HPLC analysis. NR = no reaction

Table S3. Co-solvent Screening[a]

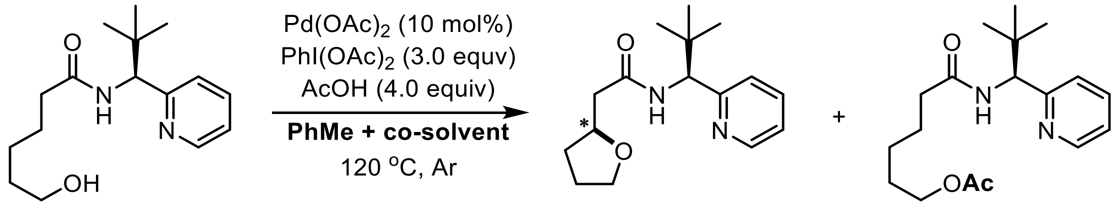

1a 2a 2a'

entry	(co)solvent (v/v)	**2a** % (d.r.)	**2a'** %
1	PhMe	63 (26 : 1)	5
2	PhMe + EtOH (5:1)	66 (27 : 1)	6
3	PhMe + EtOH (10:1)	71 (31 : 1)	4
4	PhMe + EtOH (20:1)	67 (29 : 1)	7
5	PhMe + 1,2-DCE (5:1)	49	16
6	PhMe + DMF (5:1)	44	9
7	PhMe + dioxane (5:1)	53	14

8	PhMe + HFIP (5:1)	31	1
9	PhMe + EtOAc (5:1)	54	18
10	PhMe + MeCN (5:1)	36	12
11	PhMe + DMSO (5:1)	28	8
12	PhMe + *n*PrOH (5:1)	59	10
13	PhMe + *n*BuOH (5:1)	60	9

[a] Conditions: Substrate (0.1 mmol), $Pd(OAc)_2$ (10 mol%), $PhI(OAc)_2$ (2.0 equiv), AcOH (8.0 equiv) in co-solvent (1.0 mL) at 120 °C for 6 h. The d.r. was determined by HPLC analysis.

Kinetic isotope effect (KIE) Studies

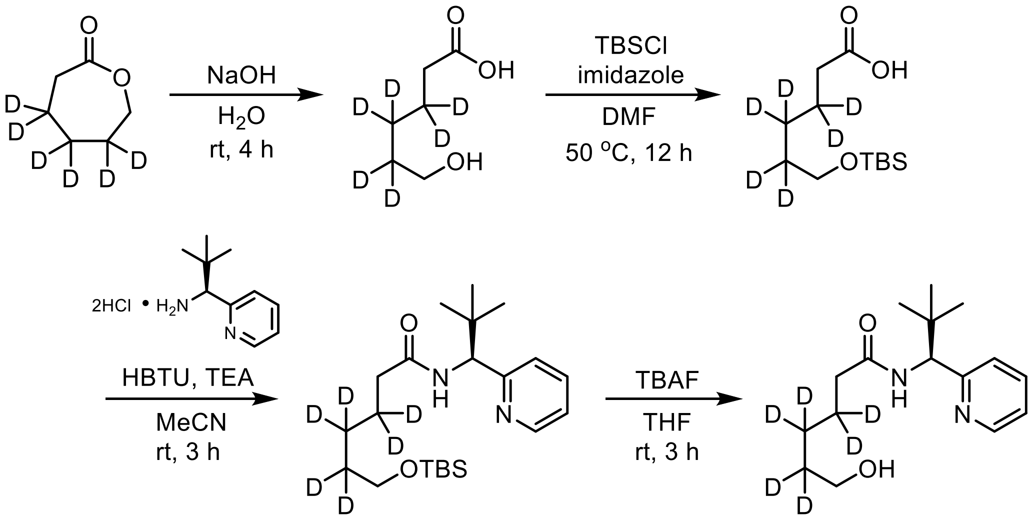

Lactone hydrolysis. To a solution of *ε*-caprolactone-3,3,4,4,5,5-*d*$_6$ (CAS No. 1219802-08-0, purchased from C/D/N Isotope Inc., 500 mg, 4.16 mmol) in H_2O (15 mL) was added NaOH (333 mg, 8.32 mmol). The reaction was stirred at room temperature for 4 h. The reaction mixture was acidified to pH 2.0 with 1N HCl aqueous solution and extracted with diethyl ether (3 × 20 mL). The combined organic layer was dried over $MgSO_4$ and concentrated to give the desired product as white solid (557 mg, 97% yield).

TBS protection. To a solution of 6-hydroxyhexanoic acid (400 mg, 2.92 mmol) and imidazole (437 mg, 6.42 mmol) in DMF (10 mL) was added *tert*-butyldimethylsilyl chloride (484 mg, 3.21 mmol) under argon atmosphere. The reaction was stirred at 50 °C for 12 h. The reaction mixture was diluted with diethyl ether (3 × 15 mL) and washed with brine (10 mL). The combined organic layer was dried over $MgSO_4$. After the removal of solvent, the residue was purified by flash column chromatography (EtOAc/hexane= 1:2) on silica gel to give the desired product as colorless oil (567 mg, 77% yield).

Amide bond formation. To a solution of 6-((*tert*-butyldimethylsilyl)oxy)hexanoic acid (567 mg, 2.25 mmol) and amine dihydrochloride salt (533 mg, 2.25 mmol) in acetonitrile (11 mL) was added HBTU (894 mg, 2.36 mmol) and trimethylamine (1.1 mL, 7.86 mmol). The reaction was stirred at room temperature for 3 h. The solvent was evaporated under vacuum. The reaction mixture was diluted with diethyl ether (3 × 15 mL) and washed with brine (10 mL). The combined organic layer was washed with saturated aqueous $NaHCO_3$ solution (3 × 10 mL) and dried over $MgSO_4$. After the removal of solvent, the residue was purified by flash column chromatography (EtOAc/hexane= 1:2) on silica gel to give the desired product as pale yellow oil (777 mg, 87% yield).

TBS deprotection. To a solution of (*S*)-6-((*tert*-butyldimethylsilyl)oxy)-*N*-(2,2-dimethyl-1-(pyridin-2-yl)propyl)hexanamide (777 mg, 1.95 mmol) in THF (2 mL) was added tetrabutylammonium fluoride solution (1.0 M in THF, 4.87 mL, 4.87 mmol) under argon atmosphere. The reaction was stirred at room temperature for 5 h. The reaction mixture was diluted with CH_2Cl_2 (3 × 15 mL) and washed with saturated aqueous $NaHCO_3$ solution (10 mL). The combined organic layer was dried over $MgSO_4$. After the removal of solvent, the residue was purified by flash column chromatography (acetone/hexane= 1:1) on silica gel to give the desired product **1a-*d*₆** as white amorphous solid (500 mg, 90% yield).

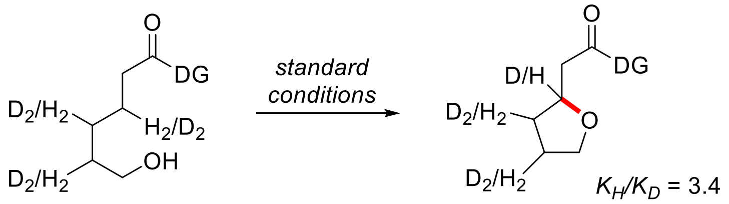

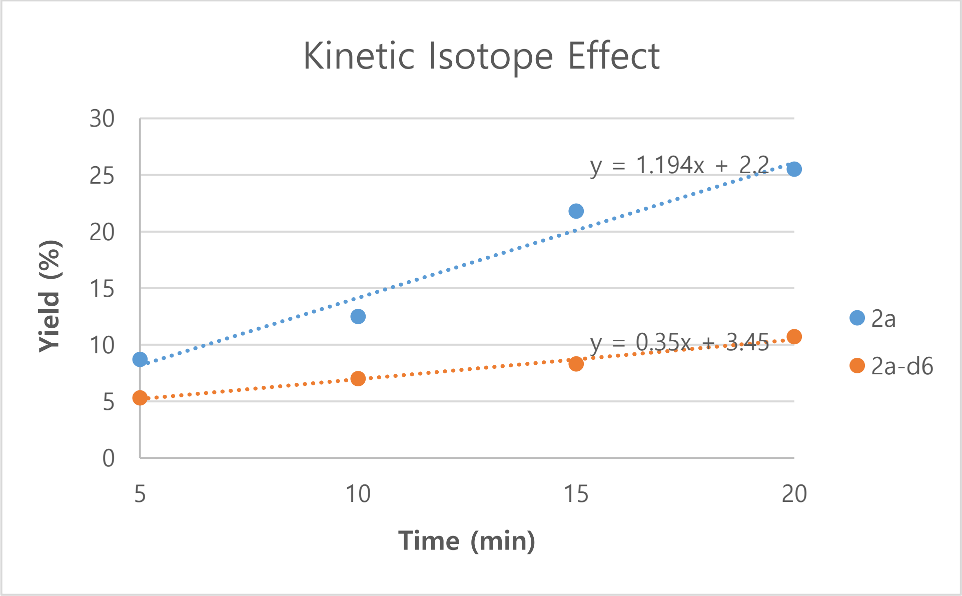

IV. Compound Characterizations

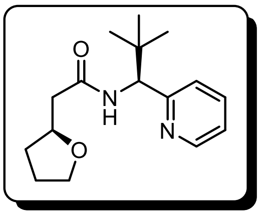

***N*-((*S*)-2,2-dimethyl-1-(pyridin-2-yl)propyl)-2-((*S*)-tetrahydrofuran-2-yl)acetamide (2a).** Monitored by TLC using acetone:hexanes = 1:2 (R_f = 0.3) as the mobile phase and purified with flash column chromatography (acetone:hexanes = 1:2). From **1a** (27.8 mg, 0.1 mmol), compound **2a** (18.4 mg, 71%) was obtained. mp 76-78 °C. White solid. ^{1}H NMR (400 MHz, DMSO-d_6) δ 8.52 (ddd, *J* = 4.8, 1.8, 0.9 Hz, 1H), 8.09 (d, *J* = 9.7 Hz, 1H), 7.73 (td, *J* = 7.9, 1.8 Hz, 1H), 7.34 (dt, *J* = 7.9, 1.1 Hz, 1H), 7.25 (ddd, *J* = 7.5, 4.8, 1.1 Hz, 1H), 4.85 (d, *J* = 9.7 Hz, 1H), 4.03 (p, *J* = 6.7 Hz, 1H), 3.76 – 3.69 (m, 1H), 3.59 – 3.52 (m, 1H), 2.44 (dd, *J* = 13.8, 6.7 Hz, 1H), 2.38 (dd, *J* = 13.8, 6.7 Hz, 1H), 1.94 – 1.73 (m, 3H), 1.55 – 1.45 (m, 1H), 0.86 (s, 9H). ^{13}C NMR (151 MHz, DMSO-d_6) δ 169.3, 159.6, 148.1, 135.8, 123.2, 122.0, 75.8, 66.8, 61.2, 41.5, 35.0, 30.6, 26.7, 25.0. HRMS (ESI$^+$) m/z calcd. for $[C_{16}H_{24}N_2NaO_2]^+$: 299.1730, found : 299.1743. d.r = 30:1. The diastereomeric ratio was determined by HPLC analysis on C18 column 4.6 × 50 mm, TFA:MeOH:H_2O = 0.1:2:98 to 0.1:50:50 gradient in 90 min, flow rate 0.5 mL/min, λ = 254 nm, t_R= 36.3 min (minor), 37.0 min (major).

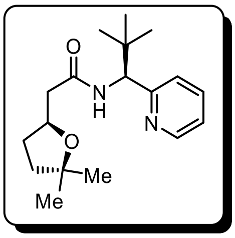

***N*-((*S*)-2,2-dimethyl-1-(pyridin-2-yl)propyl)-2-((*S*)-5,5-dimethyltetrahydrofuran-2-yl)acetamide (2b).** Monitored by TLC using acetone:hexanes = 1:3 (R_f= 0.4) as the mobile phase and purified with flash column chromatography (acetone:hexanes = 1:4). From **1b** (30.6 mg, 0.1 mmol), compound **2b** (23.2 mg, 76%) was obtained. Yellow oil. ^{1}H NMR (400 MHz, DMSO-d_6) δ 8.51 (ddd, *J* = 4.8, 1.9, 0.9 Hz, 1H), 8.09 (d, *J* = 9.6 Hz, 1H), 7.72 (td, *J* = 7.7, 1.9 Hz, 1H), 7.33 (dt, *J* = 7.7, 1.1 Hz, 1H), 7.25 (ddd, *J* = 7.5, 4.8, 1.1 Hz, 1H), 4.83 (d, *J* = 9.6 Hz, 1H), 4.14 (p, *J* = 6.4 Hz, 1H), 2.42 (dd, *J* = 13.8, 6.6 Hz, 1H), 2.36 (dd, *J* = 13.8, 6.6 Hz, 1H), 2.03 – 1.89 (m, 1H), 1.77 – 1.57 (m, 3H), 1.18 (s, 3H), 1.12 (s, 3H), 0.86 (s, 9H). ^{13}C NMR (101 MHz, DMSO-d_6) δ 169.4, 159.7, 148.1, 135.8, 123.2, 122.0, 80.1, 75.1, 61.2, 42.5, 37.8, 35.0, 31.2, 29.2, 28.0, 26.7. HRMS (ESI$^+$) m/z calcd. for $[C_{18}H_{28}N_2NaO_2]^+$: 327.2043, found : 327.2066. d.r = 15:1. The diastereomeric ratio was determined by ^{1}H NMR analysis.

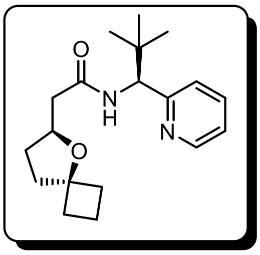

***N*-((*S*)-2,2-dimethyl-1-(pyridin-2-yl)propyl)-2-((*S*)-5-oxaspiro[3.4]octan-6-yl)acetamide (2c).** Monitored by TLC using acetone:hexanes = 1:3 (R_f = 0.3) as the mobile phase and purified with flash column chromatography (acetone:hexanes = 1:3). From **1c** (31.8 mg, 0.1 mmol), compound **2c** (20.7 mg, 66%) was obtained. Yellow oil. ^{1}H NMR (400 MHz, DMSO-d_6) δ 8.51 (ddd, *J* = 4.8, 1.9, 0.9 Hz, 1H), 8.09 (d, *J* = 9.7 Hz, 1H), 7.73 (td, *J* = 7.9, 1.9 Hz, 1H), 7.34 (dt, *J* = 7.9, 1.1 Hz, 1H), 7.26 (ddd, *J* = 7.5, 4.8, 1.1 Hz, 1H), 4.83 (d, *J* = 9.7 Hz, 1H), 4.09 (p, *J* = 6.7 Hz, 1H), 2.40 (dd, *J* = 13.8, 6.7 Hz, 1H), 2.34 (dd, *J* = 13.8, 6.7 Hz, 1H), 2.05 (dq, *J* = 19.6, 10.1 Hz, 2H), 1.98 – 1.82 (m, 5H), 1.63 – 1.51 (m, 2H), 1.50 – 1.41 (m, 1H), 0.86 (s, 9H). ^{13}C NMR (151 MHz, DMSO-d_6) δ 169.3, 159.6, 148.1, 135.8, 123.2, 122.0, 82.4, 75.3, 61.2, 42.5, 36.0, 35.9, 35.6, 35.0, 30.1, 26.7, 12.2. HRMS (ESI$^+$) m/z calcd. for $[C_{19}H_{28}N_2NaO_2]^+$: 339.2043, found : 339.2087. d.r = 17:1. The diastereomeric ratio was determined by HPLC analysis on C18 column 4.6 × 150 mm, TFA:MeOH:H_2O = 0.1:65:35, flow rate 0.5 mL/min, λ = 254 nm, t_R= 5.9 min (minor), 5.6 min (major).

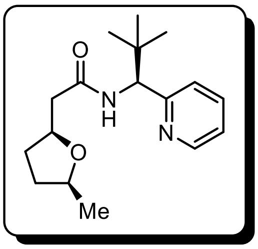

***N*-((*S*)-2,2-dimethyl-1-(pyridin-2-yl)propyl)-2-((2*S*,5*S*)-5-methyltetrahydrofuran-2-yl)acetamide (2d).** Monitored by TLC using acetone:hexanes = 1:3 (R_f= 0.3) as the mobile phase and purified with flash column chromatography (acetone:hexanes = 1:3). From **1d** (29.2 mg, 0.1 mmol), compound **2d** (21.3 mg, 73%) was obtained. Yellow oil. ^{1}H NMR (400 MHz, DMSO-d_6) δ 8.51 (ddd, *J* = 4.8, 1.9, 0.9 Hz, 1H), 8.09 (d, *J* = 9.6 Hz, 1H), 7.72 (td, *J* = 7.7, 1.9 Hz, 1H), 7.34 (dt, *J* = 7.7, 1.1 Hz, 1H), 7.25 (ddd, *J* = 7.5, 4.8, 1.1 Hz, 1H), 4.84 (d, *J* = 9.6 Hz, 1H), 4.01 (p, *J* = 6.6 Hz, 1H), 3.87 – 3.75 (m, 1H), 2.45 (dd, *J* = 13.9, 6.8 Hz, 1H), 2.39 (dd, *J* = 13.9, 6.8 Hz, 1H), 1.97 – 1.83 (m, 2H), 1.63 – 1.53 (m, 1H), 1.45 – 1.34 (m, 1H), 1.13 (d, *J* = 6.0 Hz, 3H), 0.86 (s, 9H). ^{13}C NMR (101 MHz, DMSO-d_6) δ 169.3, 159.6, 148.1, 135.8, 123.2, 122.0, 75.8, 74.6, 61.2, 42.1, 35.0, 32.3, 30.6, 26.7, 21.3. HRMS (ESI$^+$) m/z calcd. for $[C_{17}H_{26}N_2NaO_2]^+$: 313.1886, found : 313.1891. d.r = 20:1. The diastereomeric ratio was determined by HPLC analysis on C18 column 4.6 × 150 mm, TFA:MeOH:H_2O = 0.1:30:70, flow rate 0.5 mL/min, λ = 254 nm, t_R= 28.3 min (minor), 29.7 min (major).

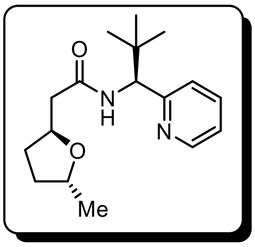

***N*-((*S*)-2,2-dimethyl-1-(pyridin-2-yl)propyl)-2-((2*S*,5*R*)-5-methyltetrahydrofuran-2-yl)acetamide (2e).** Monitored by TLC using acetone:hexanes = 1:3 (R_f = 0.3) as the mobile phase and purified with flash column chromatography (acetone:hexanes = 1:3). From **1e** (29.2 mg, 0.1 mmol), compound **2e** (19.2 mg, 67%) was obtained. Yellow oil. ^{1}H NMR (400 MHz, DMSO-d_6) δ 8.51 (ddd, *J* = 4.8, 1.9, 0.8 Hz, 1H), 8.08 (d, *J* = 9.6 Hz, 1H), 7.72 (td, *J* = 7.6, 1.9 Hz, 1H), 7.34 (dt, *J* = 7.6, 1.1 Hz, 1H), 7.25 (ddd, *J* = 7.5, 4.8, 1.1 Hz, 1H), 4.84 (d, *J* = 9.6 Hz, 1H), 4.19 (p, *J* = 6.7 Hz, 1H), 4.08 – 3.95 (m, 1H), 2.41 (dd, *J* = 13.8, 6.7 Hz, 1H), 2.35 (dd, *J* = 13.8, 6.7 Hz, 1H), 2.09 – 1.90 (m, 2H), 1.63 – 1.49 (m, 1H), 1.44 – 1.30 (m, 1H), 1.08 (d, *J* = 6.1 Hz, 3H), 0.86 (s, 9H). ^{13}C NMR (151 MHz, DMSO-d_6) δ 169.4, 159.7, 148.1, 135.8, 123.2, 122.0, 75.1, 73.7, 61.2, 41.9, 35.0, 33.1, 31.4, 26.7, 21.2. HRMS (ESI$^+$) m/z calcd. for $[C_{17}H_{26}N_2NaO_2]^+$: 313.1886, found : 313.1910. d.r = 19:1. The diastereomeric ratio was determined by HPLC analysis on C18 column 4.6 × 150 mm, TFA:MeOH:H_2O = 0.1:30:70, flow rate 0.5 mL/min, λ = 254 nm, t_R= 30.5 min (minor), 27.4 min (major).

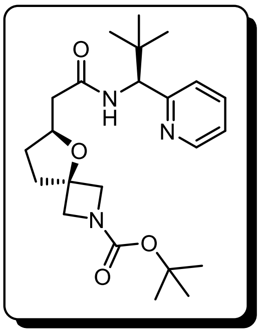

***tert*-butyl (*S*)-6-(2-(((*S*)-2,2-dimethyl-1-(pyridin-2-yl)propyl)amino)-2-oxoethyl)-5-oxa-2-azaspiro[3.4]octane-2-carboxylate (2f).** Monitored by TLC using acetone:hexanes = 1:2 (R_f = 0.3) as the mobile phase and purified with flash column chromatography (acetone:hexanes = 1:2). From **1f** (42.0 mg, 0.1 mmol), compound **2f** (28.9 mg, 70%) was obtained. Yellow oil. ^{1}H NMR (400 MHz, DMSO-d_6) δ 8.51 (ddd, *J* = 4.8, 1.8, 0.9 Hz, 1H), 8.16 (d, *J* = 9.7 Hz, 1H), 7.72 (td, *J* = 7.6, 1.8 Hz, 1H), 7.34 (dt, *J* = 7.9, 1.1 Hz, 1H), 7.26 (ddd, *J* = 7.6, 4.8, 1.1 Hz, 1H), 4.84 (d, *J* = 9.7 Hz, 1H), 4.19 (p, *J* = 6.7 Hz, 1H), 3.88 – 3.70 (m, 4H), 2.44 (dd, *J* = 13.7, 6.7 Hz, 1H), 2.38 (dd, *J* = 13.7, 6.7 Hz, 1H), 2.15 – 2.07 (m, 1H), 2.04 – 1.88 (m, 2H), 1.65 – 1.53 (m, 1H), 1.36 (s, 9H), 0.85 (s, 9H). ^{13}C NMR (151 MHz, DMSO-d_6) δ 169.1, 159.6, 155.6, 148.1, 135.7, 123.2, 122.0, 78.6, 77.6, 76.5, 61.2, 41.9,

35.0, 34.8, 30.1, 28.0, 26.7. HRMS (ESI$^+$) m/z calcd. for $[C_{23}H_{35}N_3NaO_4]^+$: 440.2520, found : 440.2548. d.r = 32:1. The diastereomeric ratio was determined by HPLC analysis on C18 column 4.6 × 150 mm, TFA:MeOH:H_2O = 0.1:50:50, flow rate 0.5 mL/min, λ = 254 nm, t_R= 19.4 min (minor), 17.1 min (major).

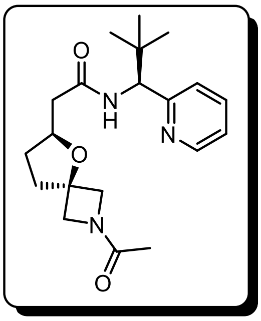

2-((*S*)-2-acetyl-5-oxa-2-azaspiro[3.4]octan-6-yl)-*N*-((S)-2,2-dimethyl-1-(pyridin-2-yl)propyl)acetamide (2g). Monitored by TLC using CH_2Cl_2:MeOH = 17:1 (R_f = 0.3) as the mobile phase and purified with flash column chromatography (CH_2Cl_2:MeOH = 17:1). From **1g** (36.1 mg, 0.1 mmol), compound **2g** (26.5 mg, 74%) was obtained. Yellow oil. ^{1}H NMR (400 MHz, DMSO-d_6) δ 8.55 – 8.48 (m, 1H), 8.13 (d, *J* = 9.7 Hz, 1H), 7.72 (tt, *J* = 7.7, 1.7 Hz, 1H), 7.34 (dt, *J* = 7.7, 1.1 Hz, 1H), 7.26 (ddt, *J* = 7.4, 4.9, 1.1 Hz, 1H), 4.84 (d, *J* = 9.7 Hz, 1H), 4.21 (pd, *J* = 6.7, 2.1 Hz, 1H), 4.14 – 3.96 (m, 2H), 3.87 – 3.68 (m, 2H), 2.46 (ddd, *J* = 13.8, 6.7, 1.9 Hz, 1H), 2.40 (ddd, *J* = 13.8, 6.7, 1.9 Hz, 1H), 2.19 – 1.89 (m, 3H), 1.74 (d, *J* = 3.5 Hz, 3H), 1.66 – 1.56 (m, 1H), 0.86 (s, 9H). ^{13}C NMR (151 MHz, DMSO-d_6) δ 169.6 (d, *J* = 8.6 Hz), 169.0 (d, *J* = 7.0 Hz), 159.6, 148.1, 135.8 (d, *J* = 2.4 Hz), 123.2 (d, *J* = 5.3 Hz), 122.0, 77.3 (d, *J* = 2.9 Hz), 76.6 (d, *J* = 10.1 Hz), 62.9 (d, *J* = 59.7 Hz), 61.2 (d, *J* = 2.3 Hz), 60.2 (d, *J* = 47.1 Hz), 41.9, 35.0 (d, *J* = 2.0 Hz), 34.8 (d, *J* = 8.3 Hz), 30.1 (d, *J* = 5.7 Hz), 26.7, 19.0 (d, *J* = 3.9 Hz). HRMS (ESI$^+$) m/z calcd. for $[C_{20}H_{29}N_3NaO_3]^+$: 382.2101, found : 382.2153. d.r = 23:1. The diastereomeric ratio was determined by HPLC analysis on C18 column 4.6 × 50 mm, TFA:MeOH:H_2O = 0.1:15:85, flow rate 0.5 mL/min, λ = 254 nm, t_R= 19.1 min (minor), 21.8 min (major).

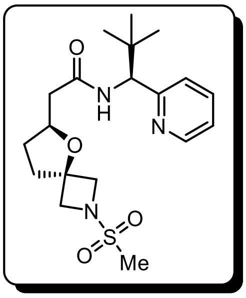

***N*-((*S*)-2,2-dimethyl-1-(pyridin-2-yl)propyl)-2-((*S*)-2-(methylsulfonyl)-5-oxa-2-azaspiro[3.4]octan-6-yl)acetamide (2h).** Monitored by TLC using CH_2Cl_2:MeOH = 15:1 (R_f= 0.3) as the mobile phase and purified with flash column chromatography (CH_2Cl_2:MeOH = 15:1). From **1h** (39.7 mg, 0.1 mmol), compound **2h** (28.8 mg, 73%) was obtained. Yellow oil. ^{1}H NMR (400 MHz, DMSO-d_6) δ 8.52 (ddd, *J* = 4.8, 1.9, 0.9 Hz, 1H), 8.13 (d, *J* = 9.7 Hz, 1H), 7.73 (td, *J* = 7.7, 1.9 Hz, 1H), 7.34 (dt, *J* = 7.7, 1.1 Hz, 1H), 7.26 (ddd, *J* = 7.5, 4.8, 1.1 Hz, 1H), 4.85 (d, *J* = 9.7 Hz, 1H), 4.21 (p, *J* = 6.7 Hz, 1H), 3.87 – 3.77 (m, 4H), 2.94 (s, 3H), 2.46 (dd, *J* = 13.8, 6.7 Hz, 1H), 2.39 (dd, *J* = 13.8, 6.7 Hz, 1H), 2.22 – 2.13 (m, 1H), 2.08 – 2.03 (m, 1H), 2.01 – 1.91 (m, 1H), 1.66 – 1.56 (m, 1H), 0.86 (s, 9H). ^{13}C NMR (101 MHz, DMSO-d_6) δ 169.0, 159.6, 148.1, 135.8, 123.2, 122.1, 76.8, 76.5, 63.1, 62.6, 61.2, 41.8, 35.0, 34.8, 32.9, 30.0, 26.7. HRMS (ESI$^+$) m/z calcd. for $[C_{19}H_{29}N_3NaO_4S]^+$: 418.1771, found : 418.1779. d.r = 24:1. The diastereomeric ratio was determined by HPLC analysis on C18 column 4.6 × 150 mm, TFA:MeOH:H_2O = 0.1:30:70, flow rate 0.5 mL/min, λ = 254 nm, t_R= 10.1 min (minor), 10.8 min (major).

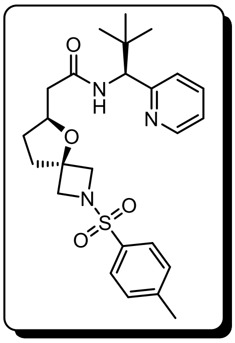

***N*-((*S*)-2,2-dimethyl-1-(pyridin-2-yl)propyl)-2-((*S*)-2-tosyl-5-oxa-2-azaspiro[3.4]octan-6-yl)acetamide (2i).** Monitored by TLC using acetone:hexanes = 1:1 (Rf = 0.5) as the mobile phase and purified with flash column chromatography (acetone:hexanes = 1:1.5). From **1i** (47.3 mg, 0.1 mmol), compound **2i** (35.8 mg, 76%) was obtained. Light yellow solid. ^{1}H NMR (400 MHz, DMSO-d_6) δ 8.50 (m, 1H), 8.01 (d, *J* = 9.6 Hz, 1H), 7.73 – 7.64 (m, 3H), 7.47 (d, *J* = 8.0 Hz, 2H), 7.30 – 7.23 (m, 2H), 4.77 (d, *J* = 9.6 Hz, 1H), 4.08 (p, *J* = 6.5 Hz, 1H), 3.70 (dd, *J* = 11.4, 8.3 Hz, 2H), 3.49 (dd, *J* = 8.2, 4.3 Hz, 2H), 2.41 (s, 3H), 2.34 (dd, *J* = 13.8, 6.6 Hz, 1H), 2.27 (dd, *J* = 13.8, 6.6 Hz, 1H), 2.03 – 1.81 (m, 3H), 1.61 – 1.49 (m, 1H), 0.81 (s, 9H). ^{13}C NMR (151 MHz, DMSO-d_6) δ 168.9, 159.5, 148.0, 144.0, 135.7, 130.3, 129.9, 128.3, 123.1, 122.0, 76.8, 76.2, 63.5, 63.2, 61.2, 41.6, 34.9, 34.7, 29.8, 26.7, 21.1. HRMS (ESI$^+$) m/z calcd. for $[C_{25}H_{34}N_3O_4S]^+$: 472.2265, found : 472.2294. d.r = 39:1. The diastereomeric ratio was determined by HPLC analysis on C18 column 4.6 × 150 mm, TFA:MeOH:H_2O = 0.1:50:50, flow rate 0.5 mL/min, λ = 254 nm, t_R= 12.3 min (minor), 11.1 min (major).

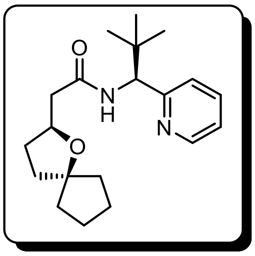

***N*-((*S*)-2,2-dimethyl-1-(pyridin-2-yl)propyl)-2-((*S*)-1-oxaspiro[4.4]nonan-2-yl)acetamide (2j).** Monitored by TLC using acetone:hexanes = 1:3 (R_f = 0.3) as the mobile phase and purified with flash column chromatography (acetone:hexanes = 1:3). From **1j** (33.2 mg, 0.1 mmol), compound **2j** (22.2 mg, 68%) was obtained. Pale yellow oil. ^{1}H NMR (400 MHz, DMSO-d_6) δ 8.50 (ddd, J = 4.8, 1.8, 0.9 Hz, 1H), 8.07 (d, J = 9.6 Hz, 1H), 7.72 (td, J = 7.9, 1.8 Hz, 1H), 7.33 (dt, J = 7.9, 1.1 Hz, 1H), 7.25 (ddd, J = 7.5, 4.8, 1.1 Hz, 1H), 4.83 (d, J = 9.6 Hz, 1H), 4.08 (p, J = 6.7 Hz, 1H), 2.42 (dd, J = 13.9, 6.7 Hz, 1H), 2.35 (dd, J = 13.9, 6.7 Hz, 1H), 1.99 – 1.89 (m, 1H), 1.88 – 1.74 (m, 2H), 1.73 – 1.42 (m, 9H), 0.86 (s, 9H). ^{13}C NMR (151 MHz, DMSO-d_6) δ 169.4, 159.6, 148.1, 135.7, 123.1, 122.0, 90.6, 74.8, 61.2, 42.5, 38.6, 37.8, 35.4, 35.0, 31.2, 26.7, 23.5, 23.4. HRMS (ESI$^+$) m/z calcd. for $[C_{20}H_{30}N_2NaO_2]^+$: 353.2199, found : 353.2241. d.r = 18:1. The diastereomeric ratio was determined by HPLC analysis on C18 column 4.6 × 50 mm, TFA:MeOH:H_2O = 0.1:60:40, flow rate 0.5 mL/min, λ = 254 nm, t_R= 3.9 min (minor), 3.4 min (major).

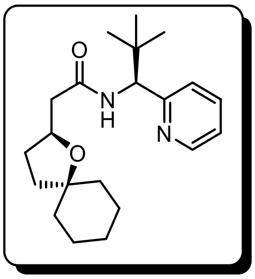

***N*-((*S*)-2,2-dimethyl-1-(pyridin-2-yl)propyl)-2-((*S*)-1-oxaspiro[4.5]decan-2-yl)acetamide (2k).** Monitored by TLC using acetone:hexanes = 1:4 (R_f = 0.3) as the mobile phase and purified with flash column chromatography (acetone:hexanes = 1:4). From **1k** (34.6 mg, 0.1 mmol), compound **2k** (23.3 mg, 68%) was obtained. Pale yellow oil. ^{1}H NMR (400 MHz, DMSO-d_6) δ 8.50 (ddd, J = 4.8, 1.9, 0.9 Hz, 1H), 8.08 (d, J = 9.6 Hz, 1H), 7.71 (td, J = 7.9, 1.9 Hz, 1H), 7.33 (dt, J = 7.9, 1.1 Hz, 1H), 7.25 (ddd, J = 7.5, 4.8, 1.1 Hz, 1H), 4.83 (d, J = 9.6 Hz, 1H), 4.10 (p, J = 6.6 Hz, 1H), 2.43 (dd, J = 13.8, 6.6 Hz, 1H), 2.34 (dd, J = 13.8, 6.6 Hz, 1H), 1.98 – 1.87 (m, 1H), 1.71 – 1.28 (m, 13H), 0.86 (s, 9H). ^{13}C NMR (151 MHz, DMSO-d_6) δ 169.5, 159.6, 148.1, 135.7, 123.1, 122.0, 81.9, 74.6, 61.1, 42.7, 38.1, 36.8, 35.7, 35.0, 30.8, 26.7, 25.2, 23.3, 23.0. HRMS (ESI$^+$) m/z calcd. for $[C_{21}H_{32}N_2NaO_2]^+$: 367.2356, found : 367.2370. d.r = 21:1. The diastereomeric ratio was determined by HPLC analysis on C18

column 4.6 × 50 mm, TFA:MeOH:H_2O = 0.1:50:50, flow rate 0.5 mL/min, λ = 254 nm, t_R= 15.9 min (minor), 12.4 min (major).

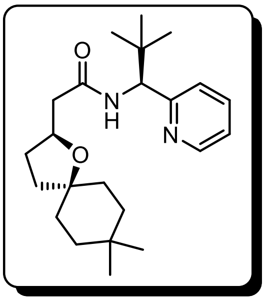

***N*-((*S*)-2,2-dimethyl-1-(pyridin-2-yl)propyl)-2-((*S*)-8,8-dimethyl-1-oxaspiro[4.5]decan-2-yl)acetamide (2l).** Monitored by TLC using acetone:hexanes = 1:4 (R_f = 0.3) as the mobile phase and purified with flash column chromatography (acetone:hexanes = 1:4). From **1l** (37.4 mg, 0.1 mmol), compound **2l** (23.3 mg, 64%) was obtained. Pale yellow oil. ^{1}H NMR (400 MHz, DMSO-d_6) δ 8.50 (ddd, *J* = 4.8, 1.9, 0.9 Hz, 1H), 8.08 (d, *J* = 9.6 Hz, 1H), 7.71 (td, *J* = 7.7, 1.9 Hz, 1H), 7.33 (dt, *J* = 7.7, 1.1 Hz, 1H), 7.25 (ddd, *J* = 7.5, 4.8, 1.1 Hz, 1H), 4.84 (d, *J* = 9.6 Hz, 1H), 4.10 (p, *J* = 6.6 Hz, 1H), 2.43 (dd, *J* = 14.0, 6.6 Hz, 1H), 2.33 (dd, *J* = 14.0, 6.6 Hz, 1H), 1.97 – 1.88 (m, 1H), 1.75 – 1.35 (m, 9H), 1.19 – 1.10 (m, 2H), 0.91 – 0.80 (m, 15H). ^{13}C NMR (151 MHz, DMSO-d_6) δ 169.4, 159.5, 148.1, 135.7, 123.2, 122.0, 81.8, 74.6, 61.1, 42.8, 35.9, 35.6, 35.0, 34.0, 32.7, 30.7, 29.2, 26.7. HRMS (ESI$^+$) m/z calcd. for $[C_{23}H_{36}N_2NaO_2]^+$: 395.2669, found : 395.2680. d.r = 17:1. The diastereomeric ratio was determined by HPLC analysis on C18 column 4.6 × 50 mm, TFA:MeOH:H_2O = 0.1:70:30, flow rate 0.5 mL/min, λ = 254 nm, t_R= 5.7 min (minor), 5.0 min (major).

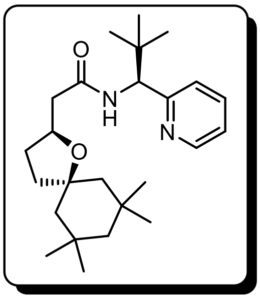

***N*-((*S*)-2,2-dimethyl-1-(pyridin-2-yl)propyl)-2-((*S*)-7,7,9,9-tetramethyl-1-oxaspiro[4.5]decan-2-yl)acetamide (2m).** Monitored by TLC using acetone:hexanes = 1:4 (R_f = 0.3) as the mobile phase and purified with flash column chromatography (acetone:hexanes = 1:4). From **1m** (40.3 mg, 0.1 mmol), compound **2m** (26.3 mg, 66%) was obtained. Pale yellow oil. ^{1}H NMR (400 MHz, DMSO-d_6) δ ^{1}H NMR (400 MHz, DMSO-d_6) δ 8.48 (ddd, *J* = 4.8, 1.9, 0.9 Hz, 1H), 8.04 (d, *J* = 9.6 Hz, 1H), 7.69 (td, *J* = 7.6, 1.9 Hz, 1H), 7.30 (dt, *J* = 7.6, 1.1 Hz, 1H), 7.23 (ddd, *J* = 7.5, 4.8, 1.1 Hz, 1H), 4.83 (d, *J* = 9.6 Hz, 1H), 4.13 (p, *J* = 6.7 Hz, 1H), 2.43 (dd, *J* = 13.4 Hz, 6.7 Hz, 1H), 2.38 (dd, 13.4 Hz, 6.7 Hz, 1H),

1.92 – 1.82 (m, 1H), 1.70 – 1.51 (m, 3H), 1.49 – 1.41 (m, 2H), 1.21 – 1.14 (m, 2H), 1.08 – 0.92 (m, 8H), 0.90 – 0.76 (m, 15H). ^{13}C NMR (151 MHz, DMSO-*d*$_6$) δ 169.4, 159.6, 148.1, 135.7, 123.1, 121.9, 83.0, 75.3, 61.1, 51.7, 48.9, 46.9, 43.0, 35.3, 35.3, 35.0, 31.4, 29.7, 28.6, 28.3, 26.8. HRMS (ESI$^+$) m/z calcd. for $[C_{25}H_{40}N_2NaO_2]^+$: 423.2982, found : 423.2980. d.r= 11:1. The diastereomeric ratio was determined by HPLC analysis on C18 column 4.6 × 50 mm, TFA:MeOH:H_2O = 0.1:70:30, flow rate 0.5 mL/min, λ = 254 nm, t_R= 14.6 min (minor), 11.1 min (major).

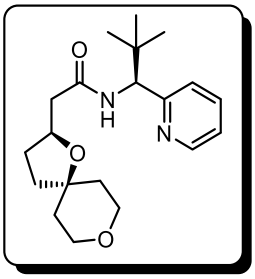

***N*-((*S*)-2,2-dimethyl-1-(pyridin-2-yl)propyl)-2-((*S*)-1,8-dioxaspiro[4.5]decan-2-yl)acetamide (2n).** Monitored by TLC using acetone:hexanes = 1:2 (R_f = 0.4) as the mobile phase and purified with flash column chromatography (acetone:hexanes = 1:3). From **1n** (34.8 mg, 0.1 mmol), compound **2n** (19.8 mg, 58%) was obtained. mp 118-121 °C. White solid. ^{1}H NMR (400 MHz, DMSO-*d*$_6$) δ 8.50 (ddd, *J* = 4.8, 1.9, 0.9 Hz, 1H), 8.08 (d, *J* = 9.6 Hz, 1H), 7.72 (td, *J* = 7.7, 1.9 Hz, 1H), 7.33 (dt, *J* = 7.7, 1.1 Hz, 1H), 7.26 (ddd, *J* = 7.5, 4.8, 1.1 Hz, 1H), 4.84 (d, *J* = 9.6 Hz, 1H), 4.15 (p, *J* = 6.9 Hz, 1H), 3.64 – 3.56 (m, 2H), 3.54 – 3.44 (m, 2H), 2.46 (dd, *J* = 13.8, 6.9 Hz, 1H), 2.36 (dd, *J* = 13.8, 6.9 Hz, 1H), 2.00 – 1.91 (m, 1H), 1.80 – 1.44 (m, 7H), 0.86 (s, 9H). ^{13}C NMR (151 MHz, DMSO-*d*$_6$) δ 169.4, 159.5, 148.1, 135.7, 123.2, 122.0, 79.2, 74.9, 64.5, 64.3, 61.1, 42.6, 38.4, 37.1, 35.8, 35.0, 30.4, 26.7. HRMS (ESI$^+$) m/z calcd. for $[C_{20}H_{30}N_2NaO_3]^+$: 369.2149, found : 369.2177. d.r = 15:1. The diastereomeric ratio was determined by HPLC analysis on C18 column 4.6 × 50 mm, TFA:MeOH:H_2O = 0.1:20:80, flow rate 0.5 mL/min, λ = 254 nm, t_R= 29.4 min (minor), 32.0 min (major).

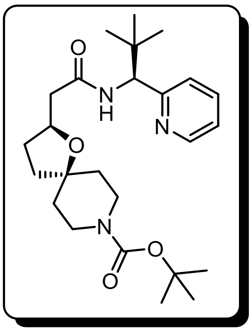

***tert*-butyl (*S*)-2-(2-(((*S*)-2,2-dimethyl-1-(pyridin-2-yl)propyl)amino)-2-oxoethyl)-1-oxa-8-azaspiro[4.5]decane-8-carboxylate (2o).** Monitored by TLC using acetone:hexanes = 1:3 (R_f = 0.3) as the mobile phase and purified with flash column chromatography (acetone:hexanes = 1:3). From **1o**

(44.8 mg, 0.1 mmol), compound **2o** (26.7 mg, 60%) was obtained. Yellow oil. ^{1}H NMR (400 MHz, DMSO-d_6) δ 8.49 (ddd, J = 4.8, 1.9, 0.9 Hz, 1H), 8.07 (d, J = 9.6 Hz, 1H), 7.70 (td, J = 7.7, 1.9 Hz, 1H), 7.33 (dt, J = 7.7, 1.1 Hz, 1H), 7.24 (ddd, J = 7.5, 4.8, 1.1 Hz, 1H), 4.84 (d, J = 9.6 Hz, 1H), 4.14 (p, J = 6.6 Hz, 1H), 3.49 – 3.37 (m, 2H), 3.21 – 3.02 (m, 2H), 2.45 (dd, J = 13.7, 6.6 Hz, 1H), 2.35 (dd, J = 13.7, 6.6 Hz, 1H), 2.02 – 1.92 (m, 1H), 1.79 – 1.58 (m, 3H), 1.52 – 1.35 (m, 13H), 0.86 (s, 9H). ^{13}C NMR (151 MHz, DMSO-d_6) δ 169.4, 159.5, 153.9, 148.1, 135.7, 123.2, 122.0, 79.8, 78.4, 75.1, 61.1, 42.7, 35.7, 35.0, 30.5, 28.1, 26.7. HRMS (ESI$^+$) m/z calcd. for $[C_{25}H_{39}N_3NaO_4]^+$: 468.2833, found : 468.2883. d.r = 14:1. The diastereomeric ratio was determined by HPLC analysis on C18 column 4.6 × 50 mm, TFA:MeOH:H$_2$O = 0.1:52:48, flow rate 0.5 mL/min, λ = 254 nm, t_R= 12.8 min (minor), 10.5 min (major).

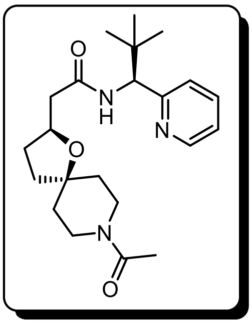

2-((*S*)-8-acetyl-1-oxa-8-azaspiro[4.5]decan-2-yl)-*N*-((*S*)-2,2-dimethyl-1-(pyridin-2-yl)propyl)acetamide (2p). Monitored by TLC using acetone:hexanes = 3:1 (R_f = 0.3) as the mobile phase and purified with flash column chromatography (acetone:hexanes = 3:1). From **1p** (39.0 mg, 0.1 mmol), compound **2p** (20.8 mg, 54%) was obtained. Pale yellow oil. ^{1}H NMR (400 MHz, DMSO-d_6) δ 8.54 – 8.46 (m, 1H), 8.08 (dd, J = 9.6, 3.1 Hz, 1H), 7.72 (tdd, J = 7.7, 3.2, 1.9 Hz, 1H), 7.34 (dt, J = 7.7, 1.1 Hz, 1H), 7.25 (ddd, J = 7.4, 4.8, 1.1 Hz, 1H), 4.85 (d, J = 9.6 Hz, 1H), 4.16 (p, J = 6.7 Hz, 1H), 3.80 – 3.68 (m, 1H), 3.48 – 3.37 (m, 1H), 3.30 – 3.02 (m, 2H), 2.47 (dd, J = 13.7, 6.7 Hz, 1H), 2.37 (dd, J = 13.7, 6.7 Hz, 1H), 2.03 – 1.94 (m, 4H), 1.83 – 1.30 (m, 7H), 0.87 (s, 9H). ^{13}C NMR (151 MHz, DMSO-d_6) δ 169.4 (d, J = 4.9 Hz), 167.8 (d, J = 5.9 Hz), 159.5, 148.1, 135.7 (d, J = 3.8 Hz), 123.2 (d, J = 4.4 Hz), 122.0 (d, J = 2.7 Hz), 79.9, 75.2 (d, J = 6.1 Hz), 61.1 (d, J = 2.3 Hz), 43.1 (d, J = 21.6 Hz), 42.7 (d, J = 3.9 Hz), 38.2 (d, J = 21.9 Hz), 37.8, 37.1, 36.5, 35.8, 35.8 (d, J = 2.9 Hz), 35.1 (d, J = 2.2 Hz), 30.5 (d, J = 5.6 Hz), 29.6, 26.7, 21.3. HRMS (ESI$^+$) m/z calcd. for $[C_{22}H_{33}N_3NaO_3]^+$: 410.2414, found : 410.2448. d.r= 11:1. The diastereomeric ratio was determined by HPLC analysis on C18 column 4.6 × 50 mm, TFA:MeOH:H$_2$O = 0.1:33:67, flow rate 0.5 mL/min, λ = 254 nm, t_R= 4.5 min (minor), 4.8 min (major).

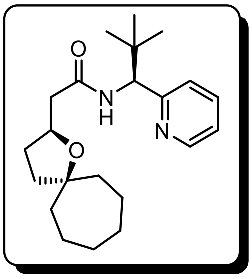

***N*-((*S*)-2,2-dimethyl-1-(pyridin-2-yl)propyl)-2-((*S*)-1-oxaspiro[4.6]undecan-2-yl)acetamide (2q).** Monitored by TLC using acetone:hexanes = 1:5 (R_f = 0.2) as the mobile phase and purified with flash column chromatography (acetone:hexanes = 1:4). From **1q** (36.1 mg, 0.1 mmol), compound **2q** (26.1 mg, 73%) was obtained. Yellow oil. ^{1}H NMR (400 MHz, DMSO-d_6) δ 8.50 (ddd, *J* = 4.8, 1.9, 0.9 Hz, 1H), 8.07 (d, *J* = 9.6 Hz, 1H), 7.72 (td, *J* = 7.7, 1.9 Hz, 1H), 7.33 (dt, *J* = 7.7, 1.1 Hz, 1H), 7.25 (ddd, *J* = 7.5, 4.8, 1.1 Hz, 1H), 4.82 (d, *J* = 9.6 Hz, 1H), 4.06 (p, *J* = 6.7 Hz, 1H), 2.42 (dd, *J* = 13.8, 6.7 Hz, 1H), 2.33 (dd, *J* = 13.8, 6.7 Hz, 1H), 1.95 – 1.86 (m, 1H), 1.74 – 1.26 (m, 15H), 0.86 (s, 9H). ^{13}C NMR (151 MHz, DMSO-d_6) δ 169.5, 159.6, 148.1, 135.7, 123.1, 122.0, 85.5, 74.7, 61.2, 42.6, 41.3, 37.7, 35.0, 30.7, 28.7, 28.7, 26.7, 22.6, 22.3. HRMS (ESI$^+$) m/z calcd. for $[C_{22}H_{34}N_2NaO_2]^+$: 381.2512, found : 381.2565. d.r = 17:1. The diastereomeric ratio was determined by HPLC analysis on C18 column 4.6 × 50 mm, TFA:MeOH:H_2O = 0.1:65:35, flow rate 0.5 mL/min, λ = 254 nm, t_R= 5.9 min (minor), 4.9 min (major).

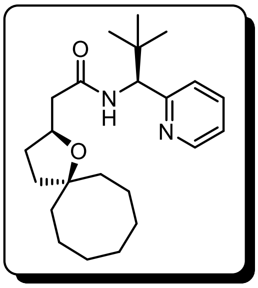

***N*-((*S*)-2,2-dimethyl-1-(pyridin-2-yl)propyl)-2-((*S*)-1-oxaspiro[4.7]dodecan-2-yl)acetamide (2r).** Monitored by TLC using acetone:hexanes = 1:5 (R_f = 0.2) as the mobile phase and purified with flash column chromatography (acetone:hexanes = 1:4). From **1r** (37.5 mg, 0.1 mmol), compound **2r** (24.3 mg, 66%) was obtained. Yellow oil. ^{1}H NMR (400 MHz, DMSO-d_6) δ 8.50 (ddd, *J* = 4.8, 1.9, 0.9 Hz, 1H), 8.06 (d, *J* = 9.6 Hz, 1H), 7.72 (td, *J* = 7.7, 1.9 Hz, 1H), 7.33 (dt, *J* = 7.7, 1.1 Hz, 1H), 7.25 (ddd, *J* = 7.5, 4.8, 1.1 Hz, 1H), 4.82 (d, *J* = 9.6 Hz, 1H), 4.07 (p, *J* = 6.7 Hz, 1H), 2.41 (dd, *J* = 13.8, 6.7 Hz, 1H), 2.33 (dd, *J* = 13.8, 6.7 Hz, 1H), 1.97 – 1.87 (m, 1H), 1.77 – 1.33 (m, 17H), 0.86 (s, 9H). ^{13}C NMR (151 MHz, DMSO-d_6) δ 169.5, 159.6, 148.1, 135.7, 123.1, 122.0, 85.4, 74.6, 61.2, 42.7, 36.6, 36.4, 35. 1, 35.0, 30.9, 27.9, 27.7, 26.7, 24.2, 22.4, 22.1. HRMS (ESI$^+$) m/z calcd. for $[C_{23}H_{36}N_2NaO_2]^+$: 395.2669, found : 395.2682. d.r = 20:1. The diastereomeric ratio was determined by HPLC analysis on

C18 column 4.6 × 50 mm, TFA:MeOH:H_2O = 0.1:70:30, flow rate 0.5 mL/min, λ = 254 nm, t_R= 5.7 min (minor), 4.7 min (major).

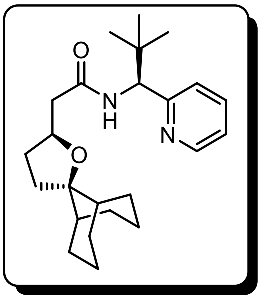

2-((1*R*,5*S*,5'*S*)-dihydro-3'H-spiro[bicyclo[3.3.1]nonane-9,2'-furan]-5'-yl)-*N*-((*S*)-2,2-dimethyl-1-(pyridin-2-yl)propyl)acetamide (2s). Monitored by TLC using acetone:hexanes = 1:4 (R_f = 0.3) as the mobile phase and purified with flash column chromatography (acetone:hexanes = 1:4). From **1s** (38.7 mg, 0.1 mmol), compound **2s** (20.3 mg, 53%) was obtained. Yellow oil. ^{1}H NMR (400 MHz, DMSO-*d*$_6$) δ 8.49 (ddd, *J* = 4.8, 1.9, 0.9 Hz, 1H), 8.06 (d, *J* = 9.6 Hz, 1H), 7.71 (td, *J* = 7.7, 1.9 Hz, 1H), 7.33 (dt, *J* = 7.7, 1.1 Hz, 1H), 7.25 (ddd, *J* = 7.5, 4.8, 1.1 Hz, 1H), 4.85 (d, *J* = 9.6 Hz, 1H), 4.11 (p, *J* = 6.8 Hz, 1H), 2.44 (dd, *J* = 13.8, 6.8 Hz, 1H), 2.34 (dd, *J* = 13.8, 6.8 Hz, 1H), 2.07 (tq, *J* = 12.8, 6.4 Hz, 2H), 1.97 – 1.53 (m, 12H), 1.44 – 1.29 (m, 4H), 0.86 (s, 9H). ^{13}C NMR (151 MHz, DMSO-*d*$_6$) δ 169.5, 159.5, 148.1, 135.7, 123. 1, 122.0, 84. 4, 74.1, 61.1, 43.2, 37.6, 36.2, 35.0, 33.7, 30.9, 30.1, 29.6, 27.1, 27.0, 26.7, 20.7, 20.5. HRMS (ESI$^+$) m/z calcd. for $[C_{24}H_{36}N_2NaO_2]^+$: 407.2669, found : 407.2678. d.r = 11:1. The diastereomeric ratio was determined by HPLC analysis on C18 column 4.6 × 50 mm, TFA:MeOH:H_2O = 0.1:65:35, flow rate 0.5 mL/min, λ = 254 nm, t_R= 15.4 min (minor), 12.0 min (major).

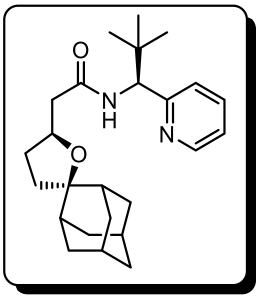

2-((1*S*,3*S*,5*S*,5'*S*,7*S*)-dihydro-3'H-spiro[adamantane-2,2'-furan]-5'-yl)-*N*-((*S*)-2,2-dimethyl-1-(pyridin-2-yl)propyl)acetamide (2t). Monitored by TLC using acetone:hexanes = 1:4 (R_f = 0.3) as the mobile phase and purified with flash column chromatography (acetone:hexanes = 1:4). From **1t** (39.9 mg, 0.1 mmol), compound **2t** (23.6 mg, 60%) was obtained. Yellow oil. ^{1}H NMR (400 MHz, DMSO-*d*$_6$) δ 8.49 (ddd, *J* = 4.8, 1.8, 0.9 Hz, 1H), 8.05 (d, *J* = 9.5 Hz, 1H), 7.70 (td, *J* = 7.7, 1.8 Hz, 1H), 7.33

(dt, J = 7.7, 1.1 Hz, 1H), 7.24 (ddd, J = 7.5, 4.8, 1.1 Hz, 1H), 4.84 (d, J = 9.6 Hz, 1H), 4.10 (p, J = 6.7 Hz, 1H), 2.44 (dd, J = 13.7, 6.7 Hz, 1H), 2.33 (dd, J = 13.7, 6.7 Hz, 1H), 2.11 – 1.99 (m, 2H), 1.96 – 1.83 (m, 2H), 1.77 – 1.52 (m, 12H), 1.46 – 1.35 (m, 2H), 0.86 (s, 9H). ^{13}C NMR (151 MHz, DMSO-d_6) δ 169.6, 159.5, 148.1, 135.7, 123. 1, 122.0, 85.8, 74.5, 61.1, 43.0, 37.9, 37.3, 36.6, 35.6, 35.0, 34.8, 34.2, 33.0, 33.0, 31.2, 26.7, 26.6, 26.5. HRMS (ESI$^+$) m/z calcd. for $[C_{25}H_{36}N_2NaO_2]^+$: 419.2669, found : 419.2667. d.r = 17:1. The diastereomeric ratio was determined by HPLC analysis on C18 column 4.6 × 50 mm, TFA:MeOH:H_2O = 0.1:65:35, flow rate 0.5 mL/min, λ = 254 nm, t_R= 15.4 min (minor), 11.1 min (major).

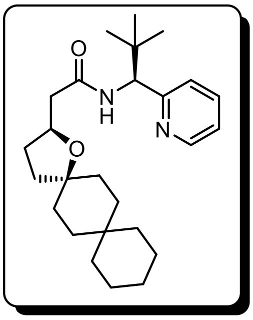

***N*-((*S*)-2,2-dimethyl-1-(pyridin-2-yl)propyl)-2-((*S*)-1-oxadispiro[4.2.5^8.2^5]pentadecan-2-yl)acetamide (2u).** Monitored by TLC using acetone:hexanes = 1:3 (R_f = 0.3) as the mobile phase and purified with flash column chromatography (acetone:hexanes = 1:3). From **1u** (41.5 mg, 0.1 mmol), compound **2u** (26.6 mg, 65%) was obtained. Yellow oil. ^{1}H NMR (400 MHz, DMSO-d_6) δ 8.50 (ddd, J = 4.8, 1.9, 0.9 Hz, 1H), 8.07 (d, J = 9.6 Hz, 1H), 7.71 (td, J = 7.7, 1.9 Hz, 1H), 7.33 (dt, J = 7.7, 1.1 Hz, 1H), 7.25 (ddd, J = 7.6, 4.8, 1.1 Hz, 1H), 4.83 (d, J = 9.6 Hz, 1H), 4.09 (p, J = 6.6 Hz, 1H), 2.42 (dd, J = 14.0, 6.6 Hz, 1H), 2.33 (dd, J = 14.0, 6.6 Hz, 1H), 1.97 – 1.87 (m, 1H), 1.74 – 1.53 (m, 3H), 1.50 – 1.32 (m, 12H), 1.30 – 1.18 (m, 6H), 0.86 (s, 9H). ^{13}C NMR (151 MHz, DMSO-d_6) δ 169.5, 159.5, 148.1, 135.7, 123.3, 122.0, 82.3, 74.5, 61.1, 42.8, 35.1, 33.2, 31.8, 31.3, 30.7, 26.7, 26.5, 21.4, 21.2. HRMS (ESI$^+$) m/z calcd. for $[C_{26}H_{40}N_2NaO_2]^+$: 435.2982, found : 435.2991. d.r = 16:1. The diastereomeric ratio was determined by HPLC analysis on C18 column 4.6 × 50 mm, TFA:MeOH:H_2O = 0.1:65:35, flow rate 0.5 mL/min, λ = 254 nm, t_R= 27.7 min (minor), 23.1 min (major).

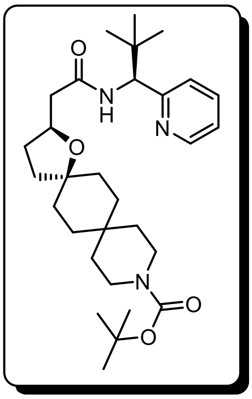

***tert*-butyl (*S*)-2-(2-(((*S*)-2,2-dimethyl-1-(pyridin-2-yl)propyl)amino)-2-oxoethyl)-1-oxa-11-azadispiro[4.2.5^8.2^5]pentadecane-11-carboxylate (2v).** Monitored by TLC using acetone:hexanes = 1:3 (R_f = 0.3) as the mobile phase and purified with flash column chromatography (acetone:hexanes = 1:3). From **1v** (51.6 mg, 0.1 mmol), compound **2v** (29.8 mg, 62%) was obtained. Yellow oil. ^{1}H NMR (400 MHz, DMSO-d_6) δ 8.50 (ddd, J = 4.8, 1.9, 0.8 Hz, 1H), 8.07 (d, J = 9.6 Hz, 1H), 7.72 (td, J = 7.8, 1.9 Hz, 1H), 7.33 (dt, J = 7.8, 1.1 Hz, 1H), 7.25 (ddd, J = 7.5, 4.8, 1.1 Hz, 1H), 4.84 (d, J = 9.6 Hz, 1H), 4.10 (p, J = 6.6 Hz, 1H), 3.30 – 3.21 (m, 4H), 2.43 (dd, J = 13.9, 6.6 Hz, 1H), 2.33 (dd, J = 13.9, 6.6 Hz, 1H), 1.97 – 1.89 (m, 1H), 1.74 – 1.55 (m, 3H), 1.38 (s, 21H), 0.86 (s, 9H). ^{13}C NMR (151 MHz, DMSO-d_6) δ 169.4, 159.5, 153.9, 148.1, 135.8, 123.2, 122.0, 82.0, 78.3, 74.6, 61.1, 54.9, 42.8, 35.1, 33.0, 32.2, 31.9, 31.7, 30.7, 30.1, 28.1, 26.7. HRMS (ESI$^+$) m/z calcd. for $[C_{30}H_{47}N_3NaO_4]^+$: 536.3459, found : 536.3456. d.r = 17:1. The diastereomeric ratio was determined by HPLC analysis on C18 column 4.6 × 150 mm, TFA:MeOH:H_2O = 0.1:70:30, flow rate 0.5 mL/min, λ = 254 nm, t_R= 17.9 min (minor), 16.4 min (major).

VI. Computational Study

All calculations were carried out using density functional theory (DFT)[6] as implemented in the Jaguar 9.1 suite of ab initio quantum chemistry programs.[7] Geometry optimizations were performed with B3LYP[8] functional including Grimme's D3 dispersion corrections[9] at 6-31G** as a basis set level.[10] Pd was described by the LACVP basis set[11] which includes relativistic effective core potentials (ECP). The electronic energies were reevaluated by single point calculations using Dunning's correlation-consistent triple-ζ basis set, cc-pVTZ(-f)[12] which includes a double set of polarization functions. For Pd, a modified version of LACVP, designated as LACV3P: containing the decontracted exponents to match the effective core potential with triple-ζ quality, was used. Vibrational frequencies were computed at the B3LYP-D3/6-31G**/LACVP level of theory. We used zero point energy (ZPE) and vibrational entropy corrections from unscaled frequencies. Solvation corrections were evaluated by a self-consistent reaction field (SCRF) approach[13] with the dielectric constant ε = 2.379 (toluene) on the gas phase optimized structures. All values in the energy profile are relative free energies (kcal/mol) at 393.15 K.

VI.1. Fragment analysis of A7-TS and B7-TS.

Here, we discuss fragment analysis of the two transition states, **A7-TS** and **B7-TS** to highlight the origin of the energetic difference. Fig. S1 illustrates the relative electronic energy of the transition states have contributions from distortion and interaction energies. **A7** loses 24.0 kcal/mol of interaction energy between the directing group (DG) and metal fragment (MF) when it transforms to **A7-TS**. The

relaxation of DG and metal fragments stabilize the transition state energy -2.1 and -6.3 kcal/mol, respectively. So, the overall electronic energy change from **A7** to **A7-TS** is 15.7 kcal/mol. In comparison, this change in energy from **B7** to **B7-TS** is 2.5 kcal/mol higher. The main component which induces this difference is the distortion energy of DG fragment, which stabilizes the **A7-TS** by 2.1 kcal/mol and destabilizes the **B7-TS** by 3.9 kcal/mol. Hence, the energy difference in the reductive elimination transition states appears to be dictated by the distortion energy of the DG fragments. The configuration of the amine ligand may have caused the difference in distortion energies, as discussed in the main text.

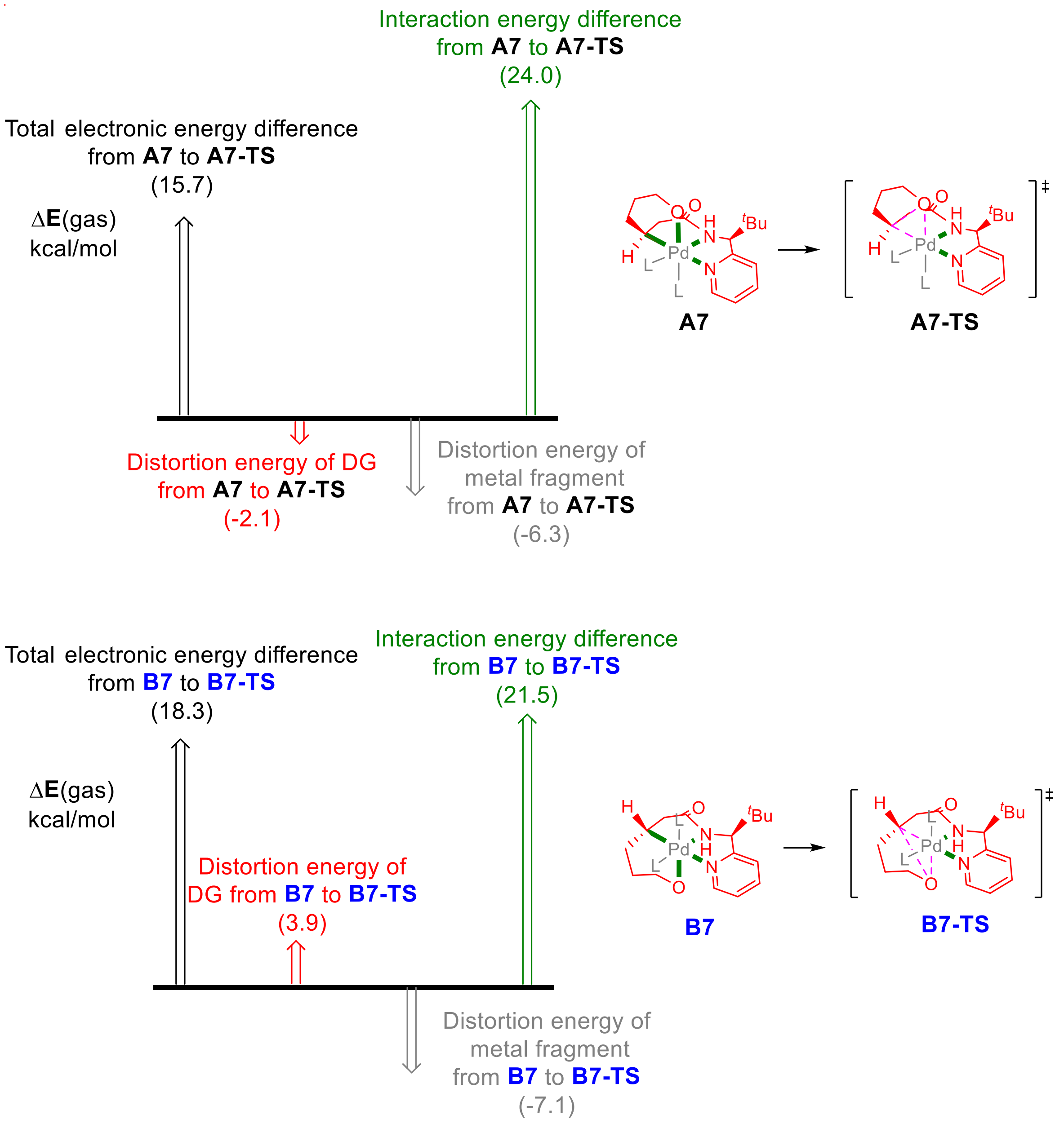

Fig. S1 Distortion/interaction model of reductive elimination step.

VI.2. Competitive Reductive Elimination Mechanism for C–O bond forming.

Scheme S7. Comparison of two possible reductive elimination mechanisms. a) Path A, b) Path B.

a)

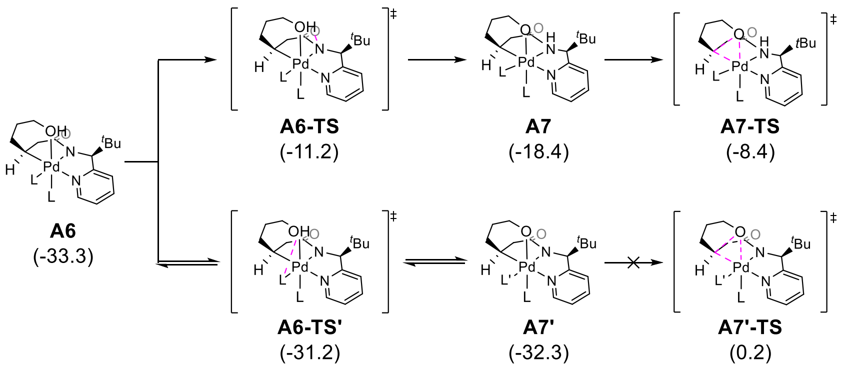

b)

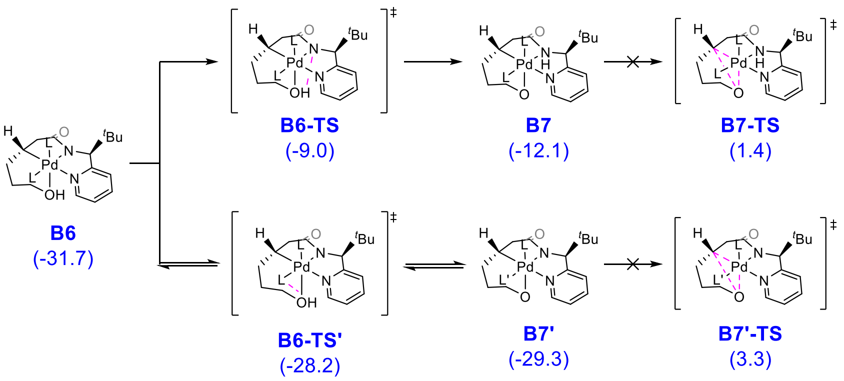

Scheme S7a describes the two possible reductive elimination steps in path A. The hydroxyl moiety of **A6** must be deprotonated to enable the reductive elimination affording the product. There are two possible pathways to mediate the deprotonation: one is the proton shift from hydroxyl to amido ligand (**A6-TS**), and the other is the acetate mediated deprotonation (**A6-TS'**). As expected, **A6-TS'** is more feasible since the acetate is more basic than the amido ligand. The barrier of the acetate mediated deprotonation step is only 2.1 kcal/mol and it gives **A7',** which is isoenergetic to **A6.** But this pathway cannot produce the product because the reductive elimination step, the following **A7'-TS**, has a relatively high barrier (33.5 kcal/mol). So, the formation of the target oxaspirocycle product(S,S) proceeds via **A6-TS**. The proton shift is 14.9 kcal/mol uphill traversing the transition state **A6-TS** associated with a barrier of 22.1 kcal/mol. Although this reaction is a significantly more uphill process compared to the acetate mediated deprotonation, the relatively low reductive elimination barrier (24.9 kcal/mol) allows the product formation. In contrast to Path A, Path B barely forms the oxaspirocycle product(S,R) in both reductive elimination pathways as illustrated in Scheme S7b. The kinetic barriers of the two reductive elimination steps **B7-TS** and **B7'-TS** are both more than 33 kcal/mol which could be difficult to overcome in the given experimental condition.

VI.3. Comparison of axial hydroxyl moiety and equatorial hydroxyl moiety complexes.

Tables S4 represents the relative free energies of octahedral Pd(IV) reactant complexes which hold hydroxyl moiety as a ligand either in axial or equatorial position. In both reductive elimination pathways discussed in Section VI.2, the reactant complex with axial hydroxyl moiety is more stable than the complex with equatorial hydroxyl moiety. For example, the axial hydroxyl moiety containing **B6** is found at -31.7 kcal/mol and this complex is 1.4 kcal/mol more stable than **B6'** which accommodates equatorial hydroxyl moiety.

Table S4. Relative free energy of reactant complexes.

	Relative free energy (kcal/mol)
**A6** (axial hydroxyl moiety)	-33.3
**A6'** (equatorial hydroxyl moiety)	-31.1
**B6** (axial hydroxyl moiety)	-31.7
**B6'** (equatorial hydroxyl moiety)	-30.3

Even though **B6** is more stable than **B6'**, the small free energy gap suggests a possibility of reductive elimination from **B6'**. As illustrated in Scheme S8, reactant complex **B6'** also has two possible reductive elimination mechanisms as discussed in Section VI.2. In both cases, the reductive elimination barriers are more than ~40 kcal/mol, which is in good agreement with the initial proposal of path B incapable of affording the product.

Scheme S8. Possible two reductive elimination steps with **B6'** which involves equatorial positioned hydroxyl moiety.

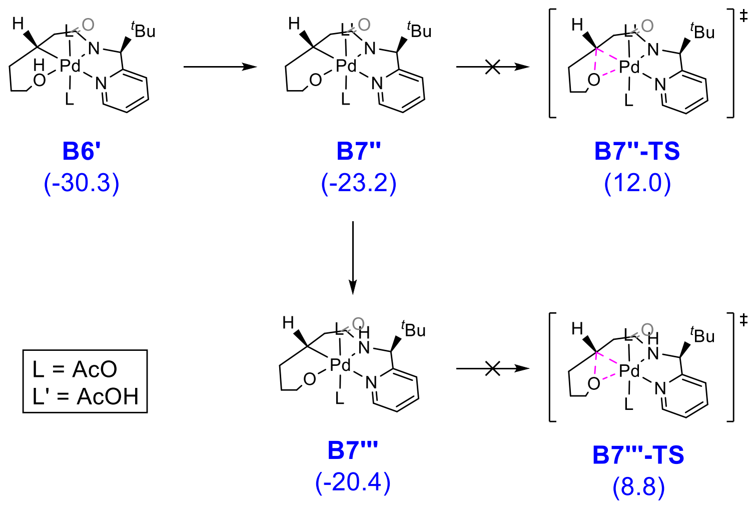

VI.4. Comparison of experimental and predicted KIE value.

The experimental KIE value (3.4) is in good agreement with the predicted KIE value (3.6) of **A3-TS** which is treated as a rate limiting step in the proposed mechanism. The predicted KIE value is gotten from below equations. (The pre-exponential factors A_H and A_D are assumed to be identical)

$$k_H = A_H \cdot e^{-Ea(H)/RT} \qquad (1)$$

$$k_D = A_D \cdot e^{-Ea(D)/RT} \qquad (2)$$

$$\frac{k_H}{k_D} = \exp\left[\frac{-(24.9-25.9)\times 10^3 \ \text{cal mol}^{-1}}{(393.15 \ \text{K})(1.98 \ \text{cal mol}^{-1} \ \text{K}^{-1})}\right] \approx 3.6 \qquad (3)$$

Table S5. Computed energy components for DFT-optimized structures.

	**E(SCF)/(eV)**	**ZPE/(kcal/mol)**	**S(gas)/(cal/mol)**	**G(solv)/(kcal/mol)**
	cc-pVTZ(-f)/LACV3P**	6-31G**/LACVP**	6-31G**/LACVP**	6-31G**/LACVP**
$PhI(OAc)_2$	-19052.771	121.12	137.237	-6.74
Acetate	-6220.611	30.18	68.855	-43.12
Acetic acid	-6236.252	38.90	68.452	-4.28
Iodobenzene	-6615.418	56.61	79.773	-1.24
**A1**	-39991.673	321.83	205.725	-9.73
**A1-TS**	-39991.218	319.12	195.059	-8.78
**A2**	-39991.376	321.09	207.215	-9.36

**A3**	-33754.408	281.26	183.111	-8.15
**A3 (D)**[a]	-33754.408	268.70	185.813	-8.15
**A3-TS**	-33753.121	277.30	180.327	-9.77
**A3-TS (D)**[a]	-33753.121	265.69	182.795	-9.77
**A3-TS'**	-33753.082	277.29	179.649	-9.68
**A4**	-33753.821	280.57	177.063	-9.00
**A5**	-27517.109	240.72	149.895	-8.74
**A6**	-39956.867	306.37	195.759	-9.13
**A6’**	-39956.850	306.96	192.436	-8.92
**B4**	-33753.786	280.67	178.447	-9.49
**B5**	-27517.089	240.39	153.342	-8.58
**B6**	-39956.883	306.70	192.723	-8.61
**B6’**	-39956.840	306.62	192.185	-8.25
**A6-TS**	-39955.857	303.97	193.316	-8.64
**A7**	-39956.286	306.94	195.399	-8.35
**A7-TS**	-39955.605	305.63	202.079	-10.66
**A8**	-39957.539	307.86	202.792	-12.59
**A6-TS'**	-39956.784	304.01	188.068	-8.89
**A7'**	-39956.740	304.69	197.424	-8.86
**A7'-TS**	-39955.358	304.40	190.520	-10.00
**B6-TS**	-39955.811	304.21	192.024	-8.13
**B7**	-39956.06	307.25	192.745	-8.29
**B7-TS**	-39955.267	306.01	199.218	-9.98
**B8**	-39957.305	308.19	201.854	-11.7
**B6-TS'**	-39956.668	304.51	190.806	-8.28
**B7'**	-39956.726	305.45	194.032	-7.96
**B7'-TS**	-39955.315	303.19	185.417	-8.18
**B7”**	-39956.458	306.08	196.017	-8.24
**B7”-TS**	-39954.725	304.54	198.891	-10.7
**B7”’**	-39956.348	306.72	197.388	-8.24
**B7”’-TS**	-39954.998	305.52	196.772	-9.17
**DG of A7**[b]	-24060.651	-	-	-
**MF of A7**[c]	-15862.286	-	-	-
**DG of A7-TS**[b]	-24060.741	-	-	-
**MF of A7-TS**[c]	-15862.558	-	-	-

**DG of B7**[b]	-24060.571	-	-	-
**MF of B7**[c]	-15862.330	-	-	-
**DG of B7-TS**[b]	-24060.403	-	-	-
**MF of B7-TS**[c]	-15862.638	-	-	-

[a] Deuterium labeled structure
[b] DG represents directing group fragment on fragment analysis
[c] MF represents metal fragment on fragment analysis

Table S6. Cartesian Coordinates of the Optimized Geometries.

===============================

$PhI(OAc)_2$

===============================

I	0.729892313	-0.125034913	-4.710765362
C	0.391378850	-0.062632956	-6.825250626
C	-0.192873135	1.079063892	-7.365354538
C	0.769336820	-1.164299130	-7.586305618
C	-0.409921736	1.105225205	-8.745039940
C	0.544644713	-1.107859254	-8.963902473
C	-0.042677466	0.019144034	-9.541406631
H	-0.495460987	1.903762341	-6.731035233
H	1.247502327	-2.021296978	-7.127019882
H	-0.866966963	1.983580112	-9.190862656
H	0.832901835	-1.953957796	-9.580510139
H	-0.213790134	0.051213261	-10.613320351
O	-1.432685494	-0.246302128	-4.452308178
C	-2.109215260	0.894827127	-4.490499020
O	-1.634315133	1.991600752	-4.750315189
C	-3.580447197	0.677237749	-4.176667213
H	-3.684920549	0.214041039	-3.191470861
H	-4.012209892	-0.011573897	-4.908491611
H	-4.106085777	1.632067561	-4.202708721
O	2.866925001	0.001486539	-5.126682281
C	3.486599445	-1.128510833	-5.442664623
O	2.941495657	-2.208377838	-5.623656750
C	4.987535000	-0.922109246	-5.566299438
H	5.385747910	-0.525916934	-4.627955914
H	5.192044735	-0.182444051	-6.345757961
H	5.468802929	-1.869301915	-5.810998440

===============================

Acetate

===============================

O	-1.109082937	-0.363739520	-4.025866508
C	-1.722265363	0.544827044	-3.409200430
O	-1.514200449	1.783529162	-3.380192280
C	-2.935598135	0.030390412	-2.544684410
H	-2.582779169	-0.704740286	-1.807836175
H	-3.654690742	-0.496092856	-3.187753677
H	-3.451093912	0.844761670	-2.020951986

===============================

Acetic Acid

===============================

O	-1.189250112	-0.451623589	-4.007086277
C	-1.782359481	0.586471319	-3.364225388
O	-1.423819780	1.734156728	-3.508269310
C	-2.909862995	0.110216707	-2.482542992
H	-2.537628889	-0.630090535	-1.768487573
H	-3.674078703	-0.380424351	-3.092958689

H	-3.341027737	0.959585071	-1.954370499
H	-0.481844962	-0.064693049	-4.549826622

================================

Iodobenzene

================================

I	0.585881054	0.000001274	-5.323780060
C	0.585963070	-0.000003732	-7.461621761
C	1.800370455	-0.000004731	-8.149033546
C	-0.628472805	-0.000001400	-8.149024963
C	1.792553902	0.000000191	-9.545749664
C	-0.620681524	-0.000001637	-9.545743942
C	0.585932672	-0.000001421	-10.246473312
H	2.740722656	-0.000005596	-7.608182430
H	-1.568820357	0.000000094	-7.608172894
H	2.737249613	0.000002832	-10.081936836
H	-1.565378189	-0.000003707	-10.081929207
H	0.585952818	-0.000002416	-11.332369804

================================

A1

================================

Pd	-11.124900818	9.334115028	8.138036728
N	-11.858442307	8.336491585	6.509900093
C	-11.356338501	7.191663742	6.012373447
C	-13.005720139	8.863156319	6.018048286
C	-11.976983070	6.528129101	4.961808205
C	-13.673941612	8.230893135	4.972525597
C	-13.151133537	7.059062958	4.431394577
H	-10.473015785	6.808273315	6.504083633
H	-11.553784370	5.600877285	4.592780590
H	-14.605452538	8.648068428	4.608512878
H	-13.668351173	6.554502964	3.620894432
C	-13.540340424	10.111306190	6.700473309
H	-14.632036209	10.037281990	6.663321495
C	-13.170553207	11.477717400	6.026973724
C	-11.660639763	11.775493622	6.025510311
H	-11.087696075	10.976212502	5.544841766
H	-11.474635124	12.700580597	5.468431950
H	-11.276094437	11.920030594	7.038527012
C	-13.676252365	11.443565369	4.570090771
H	-13.526807785	12.423899651	4.107697010
H	-13.137516975	10.707669258	3.966128588
H	-14.747251511	11.213717461	4.517947674
C	-13.917750359	12.604193687	6.774267197
H	-13.549708366	12.741209984	7.793830872
H	-13.770239830	13.552929878	6.247626781
H	-14.996090889	12.408987999	6.811233997
N	-13.141176224	10.030556679	8.138467789
C	-13.978361130	9.161072731	8.922183990
O	-14.800006866	8.458147049	8.370102882
C	-13.777375221	9.217638969	10.426143646
H	-12.722473145	9.393379211	10.657051086
H	-14.319093704	10.111317635	10.766581535
C	-14.301967621	7.952832222	11.125789642
H	-15.245543480	7.661493301	10.652444839
H	-14.530614853	8.203256607	12.168151855
C	-13.312141418	6.773200512	11.098454475
H	-12.447785378	7.015580177	11.733226776
H	-13.801437378	5.908774853	11.568814278
C	-12.804297447	6.384001732	9.704098701
H	-13.639425278	6.279878616	9.002589226
H	-12.156597137	7.177752495	9.310110092
C	-11.980782509	5.093330860	9.692945480
H	-12.624189377	4.238750935	9.942380905
H	-11.201969147	5.145403862	10.472314835

O	-11.411437035	4.837849617	8.419452667
H	-10.654844284	5.444299221	8.347323418
O	-10.517429352	10.374478340	9.788789749
C	-11.054716110	11.505644798	10.106002808
O	-12.079739571	12.004362106	9.591137886
C	-10.330456734	12.236675262	11.222650528
H	-9.317094803	12.484747887	10.892881393
H	-10.236926079	11.579018593	12.091545105
H	-10.868770599	13.145758629	11.492501259
O	-9.166621208	8.904283524	7.840445518
C	-8.725522041	7.727986813	8.166059494
O	-9.423654556	6.774852276	8.535697937
C	-7.213573456	7.602643490	8.051383972
H	-6.746695518	8.186957359	8.850557327
H	-6.868558884	8.015636444	7.099661350
H	-6.918562889	6.556614876	8.143955231
H	-12.906431198	10.934012413	8.634584427

================================

A1-TS

================================

Pd	-11.099734306	9.236200333	8.099966049
N	-11.816747665	8.294427872	6.449278355
C	-11.298861504	7.178389072	5.900973797
C	-12.964993477	8.839022636	5.976000786
C	-11.909317017	6.560654640	4.817319870
C	-13.625730515	8.240732193	4.903393269
C	-13.091008186	7.100679398	4.311110497
H	-10.416741371	6.777767181	6.385521412
H	-11.470575333	5.661465168	4.399290562
H	-14.559967041	8.665472031	4.556241989
H	-13.601397514	6.628153801	3.477082253
C	-13.496157646	10.051637650	6.716608524
H	-14.587095261	9.968997955	6.687845230
C	-13.158031464	11.441102028	6.068440914
C	-11.657498360	11.782939911	6.083649635
H	-11.058621407	11.012246132	5.587849140
H	-11.490777016	12.727564812	5.553144932
H	-11.277272224	11.907984734	7.100525856
C	-13.646767616	11.438411713	4.604896545
H	-13.530044556	12.438385963	4.175006866
H	-13.076704979	10.743220329	3.981471062
H	-14.708127975	11.172043800	4.535033226
C	-13.946781158	12.527437210	6.829072952
H	-13.631047249	12.620078087	7.869438648
H	-13.792185783	13.500745773	6.350675106
H	-15.022111893	12.315612793	6.814794540
N	-13.081026077	9.924983025	8.141544342
C	-13.974979401	9.120692253	8.898537636
O	-14.971014023	8.620772362	8.393921852
C	-13.643351555	8.994672775	10.380129814
H	-12.582236290	8.748808861	10.485913277
H	-13.770713806	10.002435684	10.797379494
C	-14.517123222	7.973511696	11.138242722
H	-15.411538124	7.758765697	10.543229103
H	-14.866587639	8.444109917	12.064723015
C	-13.807592392	6.660508156	11.520750046
H	-12.853137970	6.892955780	12.007238388
H	-14.424968719	6.153831005	12.274388313
C	-13.540452957	5.648402214	10.392055511
H	-13.135892868	4.738366604	10.853585243
H	-14.486264229	5.369618893	9.907109261
C	-12.563735008	6.110233784	9.306492805
H	-13.068763733	6.813063145	8.627203941
H	-12.273412704	5.242335320	8.692914009
O	-11.428833008	6.710025311	9.910943985

H	-10.670656204	6.658707619	9.291468620
O	-10.485818863	10.344867706	9.761965752
C	-11.107208252	11.373687744	10.125220299
O	-12.231717110	11.767813683	9.628263474
C	-10.502202034	12.250664711	11.189567566
H	-9.987560272	13.136795044	10.655062675
H	-9.721372604	11.658057213	11.773100853
H	-11.329149246	12.655622482	11.854762077
O	-9.118387222	8.835805893	7.805195808
C	-8.623135567	7.652081966	7.958981991
O	-9.269863129	6.617400646	8.206568718
C	-7.111183643	7.599266052	7.804234028
H	-6.651886940	8.032720566	8.698776245
H	-6.792529106	8.198200226	6.947394848
H	-6.781032085	6.565279007	7.696671009
H	-12.660794258	11.005341530	8.898422241

===============================

A2

===============================

Pd	-11.012580872	9.239967346	7.970491409
N	-11.868062973	8.309853554	6.410300255
C	-11.369425774	7.204175472	5.831809521
C	-13.060308456	8.837680817	6.035214424
C	-12.056353569	6.562722206	4.809545517
C	-13.794730186	8.215544701	5.026484966
C	-13.287626266	7.076006889	4.405132771
H	-10.424377441	6.850295067	6.220545769
H	-11.637450218	5.668773651	4.361854076
H	-14.760470390	8.620501518	4.748693943
H	-13.857452393	6.584724426	3.621967793
C	-13.540038109	10.050102234	6.814363003
H	-14.624070168	9.945724487	6.928134441
C	-13.313531876	11.433545113	6.110986710
C	-11.824951172	11.786508560	5.943912983
H	-11.283759117	11.015223503	5.385897636
H	-11.726196289	12.729153633	5.393375874
H	-11.327380180	11.917515755	6.908355713
C	-13.975354195	11.409628868	4.718292713
H	-13.930062294	12.406562805	4.267876148
H	-13.472700119	10.717245102	4.036222458
H	-15.032240868	11.124392509	4.781333923
C	-14.016034126	12.515973091	6.956055164
H	-13.577805519	12.613837242	7.950974941
H	-13.930229187	13.489668846	6.460988998
H	-15.080964088	12.290956497	7.078808784
N	-12.951786995	9.951748848	8.177051544
C	-13.827850342	9.371475220	9.117435455
O	-15.040693283	9.325507164	8.947225571
C	-13.187373161	8.731005669	10.333152771
H	-12.181827545	9.117132187	10.522663116
H	-13.813340187	8.933317184	11.207763672
C	-13.120354652	7.212862968	10.049508095
H	-12.732576370	7.046181679	9.040457726
H	-14.142579079	6.815396309	10.057128906
C	-12.237597466	6.423687935	11.023742676
H	-11.246129036	6.893891335	11.076518059
H	-12.667693138	6.475046158	12.032740593
C	-12.086217880	4.953576088	10.581343651
H	-11.898069382	4.308786869	11.450744629
H	-13.031538963	4.610130310	10.139517784
C	-10.939340591	4.726215363	9.582448959
H	-10.990382195	3.698923111	9.197173119
H	-9.979932785	4.831629753	10.110674858
O	-10.965151787	5.611726761	8.466762543
H	-10.432437897	6.382790565	8.748589516

O	-10.167270660	10.375287056	9.534190178
C	-10.689760208	11.428086281	9.953547478
O	-11.867156982	11.856976509	9.599838257
C	-9.934918404	12.304474831	10.913825035
H	-9.245205879	12.932444572	10.339155197
H	-9.342763901	11.682239532	11.586652756
H	-10.617399216	12.946800232	11.470979691
O	-9.120162010	8.609143257	7.493238926
C	-8.523677826	7.902511120	8.407685280
O	-9.087153435	7.370431900	9.376768112
C	-7.023168564	7.775155067	8.205159187
H	-6.547033787	8.715664864	8.502515793
H	-6.791626453	7.608893871	7.150043488
H	-6.628936291	6.963539600	8.817935944
H	-12.348375320	11.176298141	8.961027145

==============================

A3

==============================

C	-3.913718224	1.454022288	1.232185841
C	-2.849029779	0.578483701	0.537572384
C	-2.176861286	-0.407004446	1.490347505
O	-2.439046144	-1.615240335	1.434572577
N	-1.307078719	0.102690689	2.419190884
Pd	-0.892997861	2.016496658	2.856301785
C	-0.664755285	-0.833206058	3.353765726
H	-1.281064272	-1.738680363	3.396658182
C	0.778524935	-1.298646808	2.899320841
C	1.802174091	-0.151524723	2.956271410
H	2.782216787	-0.513170004	2.626105070
H	1.512199879	0.673734546	2.298590183
H	1.919508219	0.247489154	3.969848871
C	1.254484653	-2.444850445	3.813496113
H	2.201098680	-2.850970984	3.441314697
H	1.429424405	-2.108729362	4.840576649
H	0.528320849	-3.266005993	3.836265326
C	0.689351499	-1.821871161	1.453263402
H	-0.064905614	-2.606232882	1.356857419
H	0.419757187	-1.017063618	0.764384985
H	1.662447333	-2.220501423	1.144352317
C	-0.666055143	-0.167311981	4.708097935
C	-0.670237958	-0.836212873	5.931582928
N	-0.686103284	1.184709907	4.687633038
C	-0.677653551	-0.104675949	7.115978241
H	-0.689124823	-1.919244051	5.942255497
C	-0.696578383	1.905814767	5.820694923
C	-0.682187140	1.290628076	7.064930439
H	-0.690469921	-0.618618846	8.072467804
H	-0.720616460	2.983646870	5.697377682
H	-0.692760706	1.893169045	7.966126442
H	-3.311588526	-0.034808829	-0.240845039
H	-2.098869562	1.226786256	0.073850542
H	-4.386686802	2.098967791	0.481888652
C	-4.984862328	0.639411569	1.988301158
H	-4.571930408	-0.330040634	2.290277958
H	-5.810520649	0.395361543	1.309218407
C	-5.544400692	1.335763812	3.241088390
H	-6.019096375	2.290215731	2.971385717
H	-6.326592922	0.701759398	3.676565170
C	-4.482124329	1.614291072	4.317820072
H	-3.807594776	2.423266411	3.992965698
H	-4.969062805	1.959247470	5.236767292
O	-3.733579874	0.459229797	4.680379868
H	-3.197201490	0.216868490	3.912031174
O	-0.495983541	4.102625370	3.045401812
C	-0.695035040	4.238914967	1.790154934

O	-1.000387073	3.201938868	1.106482387
H	-3.397805929	2.135796547	1.915635467
C	-0.596642733	5.583114147	1.130582571
H	-1.606455326	5.972912312	0.963536084
H	-0.047059558	6.277641296	1.767599702
H	-0.110714182	5.483003139	0.157341376

==============================

A3-TS

==============================

C	-2.228770018	1.806027055	5.692164421
C	-2.224452734	0.689907789	6.761240959
C	-1.161732435	-0.388577640	6.554758072
O	-1.244640112	-1.526276708	7.022696495
N	-0.126601875	0.065765508	5.803149223
Pd	0.032338198	2.000046730	5.446656227
C	1.056925893	-0.747009635	5.553360462
H	1.114545465	-1.522975206	6.328382492
C	0.996350467	-1.498811126	4.159935474
C	1.011006713	-0.503927231	2.985873699
H	0.921531737	-1.045348763	2.037380457
H	0.177824914	0.201336026	3.052185297
H	1.940237284	0.075365894	2.950602055
C	2.190104246	-2.463414907	4.024176121
H	2.076701403	-3.071204185	3.120086432
H	3.144050598	-1.933858633	3.938933134
H	2.249629021	-3.149729967	4.877583981
C	-0.304857910	-2.322431087	4.115213871
H	-0.388171107	-2.986874819	4.978904247
H	-1.182935119	-1.673035383	4.121523380
H	-0.331136793	-2.921772242	3.198060989
C	2.264164686	0.160293236	5.655278683
C	3.554721832	-0.282276154	5.952863216
N	2.044242620	1.478012443	5.421692371
C	4.611793995	0.624360025	5.961943626
H	3.716233015	-1.329116106	6.180482388
C	3.056414127	2.360633850	5.425350189
C	4.364636421	1.967898846	5.678030014
H	5.617493153	0.286535025	6.194250584
H	2.775378704	3.390826225	5.233410835
H	5.161496162	2.703536034	5.671247959
H	-2.052124739	1.129798412	7.751819611
H	-3.199148893	0.191684157	6.816333294
H	-1.612384200	3.093165636	5.755334854
C	-2.668050766	1.366360545	4.284121990
H	-2.045628548	0.530448377	3.946181059
H	-2.501420259	2.184921503	3.576129198
C	-4.145247936	0.949297011	4.194027901
H	-4.353748322	0.116719387	4.877148628
H	-4.789311409	1.785518289	4.496652126
C	-4.538387299	0.513085723	2.787428141
H	-3.907776117	-0.339664549	2.481407881
H	-5.585094452	0.166092664	2.782610178
O	-4.361187935	1.624208093	1.911786318
H	-4.535773754	1.329085231	1.010123014
O	0.468255550	4.026094913	5.054861546
C	-0.486114204	4.821246147	5.317986488
O	-1.616488099	4.430103779	5.739424229
H	-2.985643148	2.525337696	6.043661118
C	-0.277863622	6.301229477	5.102748871
H	-0.604475439	6.849171638	5.990128040
H	0.766627610	6.521032333	4.881259918
H	-0.905777097	6.626123428	4.267128944

==============================

A3-TS'

===============================

C	-2.294480562	1.774702072	5.901708603
C	-2.192077637	0.734800220	7.030408382
C	-1.060107589	-0.276955903	6.821435452
O	-0.991666436	-1.345821142	7.432817459
N	-0.168192327	0.125196695	5.874979973
Pd	-0.119043283	2.034645081	5.379831314
C	1.016822577	-0.662046075	5.560715199
H	1.196274281	-1.361146688	6.388646603
C	0.818226039	-1.537186265	4.256388664
C	0.655212164	-0.647928834	3.011305571
H	0.464325428	-1.269851446	2.129665375
H	-0.186398223	0.041087154	3.128001690
H	1.553677320	-0.053310253	2.811163902
C	2.018167496	-2.482506037	4.056225777
H	1.816529393	-3.169437885	3.227278948
H	2.936765671	-1.941082001	3.808971643
H	2.203731775	-3.090050220	4.950231075
C	-0.453617215	-2.385167360	4.447702885
H	-0.412277371	-2.965131521	5.373436928
H	-1.340964913	-1.749954939	4.499141693
H	-0.573383033	-3.070699692	3.601341248
C	2.192303419	0.284459144	5.436831474
C	3.524753809	-0.100332081	5.604790211
N	1.899763703	1.573089600	5.132060528
C	4.543071747	0.830219090	5.415201664
H	3.749413729	-1.121039867	5.890036583
C	2.874589682	2.478618145	4.949247837
C	4.216996670	2.141658068	5.066778660
H	5.580703259	0.537036955	5.545610428
H	2.538310289	3.485382795	4.722829819
H	4.981429577	2.894725800	4.909023762
H	-2.028103113	1.227313042	7.998000622
H	-3.124596119	0.164538339	7.132944107
H	-2.341165304	1.247861266	4.940935135
C	-3.596113443	2.607645273	5.952387333
H	-3.631302118	3.239623308	6.847813129
H	-4.435617447	1.905150294	6.033290386
C	-3.803386211	3.468854189	4.701943398
H	-2.944733620	4.133403778	4.552793503
H	-3.863436460	2.822824955	3.816418409
C	-5.061985016	4.323242664	4.769668579
H	-5.000439167	4.992312908	5.645268917
H	-5.127652645	4.961097717	3.871969461
O	-6.184326172	3.450463533	4.863864422
H	-6.976503372	3.986773252	4.988120079
O	0.137242109	4.112335682	5.008307934
C	-0.551251352	4.794904709	5.821567059
O	-1.425010920	4.282859325	6.599896908
H	-1.580299020	3.058137655	6.241549969
C	-0.371921897	6.292220592	5.861631870
H	-0.426319063	6.647723675	6.892600536
H	0.574027538	6.579358578	5.401685715
H	-1.193676829	6.755974293	5.305395126

===============================

A4

===============================

C	-1.914594293	1.773741841	5.732183456
C	-2.073543072	0.584761798	6.701739311
C	-1.022916794	-0.511973977	6.484173298
O	-1.110962629	-1.651260018	6.945301056
N	0.020481899	-0.046549264	5.741889000
Pd	0.111967482	1.891654134	5.373106480
C	1.206549287	-0.854808748	5.519300938
H	1.235193729	-1.637594104	6.288995266

C	1.192556143	-1.607640743	4.125977516
C	1.266263604	-0.622321367	2.946710825
H	1.199265480	-1.166146874	1.997655869
H	0.436340302	0.088431805	2.988193989
H	2.205451965	-0.057589777	2.940436602
C	2.375983715	-2.591202736	4.043855667
H	2.311288595	-3.181419611	3.123058558
H	3.342384577	-2.076812029	4.032917976
H	2.369591951	-3.292852640	4.886499882
C	-0.121841051	-2.403847218	4.032119274
H	-0.257326275	-3.052026272	4.901432991
H	-0.980864584	-1.729306817	3.993941069
H	-0.123855457	-3.015556097	3.122625589
C	2.434863329	0.025649067	5.680323601
C	3.644097805	-0.481822312	6.165432453
N	2.329223156	1.334780812	5.338739872
C	4.754908562	0.349607080	6.275599957
H	3.698404789	-1.523974061	6.459043026
C	3.403046370	2.134418249	5.447963238
C	4.638319969	1.688024163	5.901893616
H	5.695331573	-0.039438996	6.654992104
H	3.251406431	3.170344353	5.158499241
H	5.475430012	2.374430180	5.970637798
H	-1.955685854	0.921663761	7.738990784
H	-3.069432735	0.125643224	6.637936115
H	1.408846974	2.679756641	3.508771181
C	-2.766772985	1.615199447	4.469376564
H	-3.818169832	1.559558034	4.792365551
H	-2.552452803	0.641739011	4.004980564
C	-2.623141289	2.745423079	3.428684711
H	-3.582971811	2.953966856	2.942222357
H	-2.313836813	3.669449329	3.929933786
C	-1.588261962	2.418979645	2.359316587

H	-0.707281232	1.966583371	2.838304996
H	-1.993180752	1.677369952	1.652116776
O	-1.229024291	3.628829956	1.681104541
H	-0.493841171	3.425986528	1.088273883
O	0.089487500	4.033299923	4.832010269
C	0.680756927	4.395792007	3.814363241
O	1.460890889	3.576555014	3.104933262
H	-2.154574394	2.727833033	6.218419075
C	0.573232532	5.777763844	3.250775814
H	-0.226488635	5.740866184	2.504142761
H	0.299120486	6.478243828	4.039678574
H	1.502339005	6.078006744	2.762627363

==============================

A5

==============================

C	-1.966840625	1.626308203	5.765863419
C	-2.298043728	0.168956280	6.122776508
C	-1.053411365	-0.691768885	6.367093563
O	-1.059756398	-1.737627864	7.020220280
N	0.020839918	-0.190054446	5.697404385
Pd	-0.031931445	1.695827365	5.102100849
C	1.289875865	-0.898997188	5.674114227
H	1.350730181	-1.538847208	6.564852715
C	1.430010319	-1.863115311	4.422016144
C	1.472514272	-1.069625854	3.104655027
H	1.483190060	-1.759575129	2.252974033
H	0.593330324	-0.424768746	3.013774872
H	2.364387512	-0.437891841	3.033942938
C	2.710547209	-2.710295916	4.553176880
H	2.764874935	-3.437916517	3.735800266
H	3.618273497	-2.100091219	4.505974770
H	2.722405910	-3.271120071	5.495707989

C	0.215307847	-2.810268641	4.397023201
H	0.088094831	-3.324644804	5.352398396
H	-0.705646098	-2.256273508	4.199231148
H	0.341846883	-3.553778648	3.601330519
C	2.419653654	0.112971403	5.720253468
C	3.648659468	-0.179486409	6.320356369
N	2.204066515	1.321169496	5.143074989
C	4.673698902	0.761714458	6.301268101
H	3.785239220	-1.141388416	6.801602364
C	3.197037697	2.223295450	5.126442909
C	4.450302601	1.990901947	5.681262970
H	5.629291058	0.542434335	6.768856525
H	2.965013742	3.176615238	4.657508373
H	5.217447758	2.756993532	5.641052246
H	-2.962376833	0.092594326	6.991977692
H	-2.826421738	-0.310196251	5.286154270
C	-2.973581076	2.279763460	4.826140881
H	-3.977852821	2.206905127	5.276272297
H	-3.027764559	1.716419220	3.883354187
C	-2.711143255	3.763581038	4.519279003
H	-3.577255249	4.186038971	3.994065523
H	-2.600645542	4.330218792	5.453264713
C	-1.497961879	4.041575432	3.643670797
H	-1.515169621	3.408416271	2.746360540
H	-1.483084679	5.095187664	3.337891102
O	-0.278579980	3.782798290	4.392467022
H	0.474526465	3.994327545	3.825383902
H	-1.885065794	2.223167419	6.686535358

================================

A6

================================

C	-2.031259060	1.645229936	5.674683571
C	-2.144646645	0.455549926	6.620721340
C	-1.071709514	-0.612631679	6.362344265
O	-1.143978715	-1.750585794	6.812221050
N	-0.056743406	-0.164001971	5.559625626
Pd	0.027945537	1.806883574	5.375318527
C	1.184846997	-0.936951816	5.450002193
H	1.199283242	-1.613607645	6.311275005
C	1.239237547	-1.835191131	4.139153957
C	1.704815626	-1.037444353	2.909013271
H	1.639817595	-1.666776180	2.013876915
H	1.088651776	-0.149573669	2.758087158
H	2.745654583	-0.710830271	3.007654428
C	2.207964420	-3.010724783	4.375588417
H	2.196539879	-3.681190491	3.509149790
H	3.240914822	-2.674841404	4.505693913
H	1.919185042	-3.596550941	5.255591393
C	-0.161074802	-2.414155245	3.862424850
H	-0.554802358	-2.937681913	4.736578941
H	-0.864923894	-1.619178772	3.600030899
H	-0.110560149	-3.114671946	3.021146059
C	2.391902447	-0.022064211	5.552433014
C	3.639297962	-0.496453166	5.971669197
N	2.244115114	1.265864372	5.184710979
C	4.734002113	0.359396905	5.968864441
H	3.732365131	-1.516230702	6.324200153
C	3.298320055	2.094331026	5.175510406
C	4.571274281	1.680212140	5.545155048
H	5.705144405	0.002585707	6.300196171
H	3.093813896	3.107556343	4.844039440
H	5.403014660	2.376007080	5.517099380
H	-2.032117128	0.776965141	7.658734322
H	-3.125365257	-0.027503610	6.529421329
C	-2.759307384	1.455009222	4.360078335

H	-3.825932264	1.600128889	4.592620850
H	-2.656883478	0.412562698	4.031973362
C	-2.364569426	2.399224758	3.212935925
H	-3.196983576	2.490862608	2.504982710
H	-2.152716398	3.402435541	3.601395845
C	-1.160635352	1.878740788	2.433273792
H	-1.379394650	0.888765335	2.016773701
H	-0.915879369	2.555903196	1.606210589
O	0.022487476	1.735374808	3.233670235
H	0.585813165	2.605228901	3.156620502
H	-2.225019217	2.614506483	6.137033463
O	0.059504531	2.201665163	7.346446991
C	0.755405366	1.515677691	8.234077454
O	1.396534204	0.494681090	8.048140526
C	0.651423037	2.180001259	9.605876923
H	-0.394927025	2.215612173	9.923281670
H	1.013855815	3.210288048	9.550463676
H	1.240202904	1.609409928	10.324938774
O	-0.015871903	3.925786257	5.208706856
C	0.650156260	4.517470360	4.287005901
O	1.173142791	3.950007677	3.290987730
C	0.795123041	6.017695427	4.452440262
H	1.295405746	6.230676174	5.401684284
H	-0.197431490	6.475679398	4.499592781
H	1.362683773	6.440534115	3.622829437

===============================

A6'

===============================

C	-2.165241480	1.602782845	5.717176437
C	-2.416851759	0.112982862	5.878585339
C	-1.147355914	-0.664269984	6.241081715
O	-1.131656647	-1.634843230	6.990458012
N	-0.069912061	-0.224534258	5.517429829
Pd	-0.175450534	1.722521544	5.101514816
C	1.252000093	-0.826936245	5.709445477
H	1.275721312	-1.295175076	6.701531887
C	1.503329396	-1.972407103	4.640330315
C	1.538662314	-1.387240171	3.218379974
H	1.630549669	-2.196954489	2.485411644
H	0.619164348	-0.832977593	3.016123772
H	2.385324001	-0.707986712	3.075093269
C	2.829522133	-2.695111752	4.943591118
H	2.945374966	-3.551186085	4.270222664
H	3.698676109	-2.047078609	4.800437927
H	2.850931883	-3.078975916	5.970799446
C	0.363299578	-3.006403446	4.725320339
H	0.244295776	-3.392607927	5.740277290
H	-0.590863883	-2.569073677	4.422008038
H	0.577524841	-3.842635393	4.050111771
C	2.336566448	0.232383996	5.637636662
C	3.563910961	0.082157932	6.286304474
N	2.091496468	1.314632177	4.872698784
C	4.563024998	1.031973839	6.079936028
H	3.728911400	-0.771464229	6.933856964
C	3.047873735	2.230469465	4.674635410
C	4.314681053	2.113514185	5.237171650
H	5.524412632	0.927402496	6.574970722
H	2.767246723	3.072539568	4.053004742
H	5.065989017	2.872955799	5.050373077
H	-3.174105883	-0.068687558	6.648842335
H	-2.786275148	-0.312449425	4.936760902
C	-3.081541538	2.354045391	4.783057690
H	-4.106331348	2.171624899	5.146550179
H	-3.029416084	1.923252106	3.775429010
C	-2.841073751	3.870568752	4.737906456

H	-3.703794241	4.363228798	4.273345470
H	-2.757535934	4.269121170	5.756958961
C	-1.611770749	4.275393009	3.938863993
H	-1.666875005	3.856435299	2.926788330
H	-1.554210782	5.367588043	3.857403040
O	-0.393348724	3.835179806	4.573294640
H	0.271064192	3.733694315	3.828473091
H	-2.054503679	2.099344969	6.682466984
O	-0.580038190	1.291481495	3.115525961
C	0.143869266	1.929834604	2.245331764
O	0.813756049	2.958961248	2.467183590
C	0.129139289	1.308874488	0.862645090
H	-0.884461522	1.001432657	0.593908191
H	0.751419783	0.408232540	0.880638897
H	0.526941419	2.012236118	0.130281523
O	0.163954496	2.007643700	7.077712536
C	1.005840659	2.969519138	7.418725014
O	1.621571302	3.696254730	6.653417110
C	1.140445113	3.060982943	8.934432983
H	1.543145895	2.121923447	9.325785637
H	0.158693254	3.211903572	9.392133713
H	1.805999517	3.887191534	9.188350677

================================

B4

================================

C	-2.359509230	1.411844492	5.694569588
C	-2.300159216	0.347402751	6.804121017
C	-1.099184871	-0.586934745	6.672983646
O	-0.988576174	-1.658263683	7.273155689
N	-0.170827135	-0.064257294	5.826719761
Pd	-0.370534956	1.790723920	5.187343597
C	1.105024934	-0.727656305	5.597686768
H	1.295966744	-1.395841002	6.447930813
C	1.099643946	-1.641344547	4.302779675
C	0.955740392	-0.804942369	3.019164562
H	0.889726937	-1.467217803	2.148518324
H	0.048606496	-0.193724483	3.048326731
H	1.808843851	-0.135585487	2.865679741
C	2.402668238	-2.460628033	4.234791756
H	2.358044863	-3.166036129	3.397808552
H	3.282250881	-1.827703834	4.079284191
H	2.555642843	-3.043950081	5.150904179
C	-0.089832902	-2.614445925	4.406594276
H	-0.075324379	-3.165202856	5.350342751
H	-1.038702965	-2.074666262	4.359177113
H	-0.060412373	-3.326366425	3.573795795
C	2.198685646	0.323734462	5.538228989
C	3.516566515	0.062742770	5.925427914
N	1.852962017	1.546053410	5.070380688
C	4.484040737	1.056059122	5.797331333
H	3.768908024	-0.912099183	6.327039719
C	2.785067081	2.500731945	4.941405773
C	4.117959023	2.300388575	5.283099174
H	5.509556293	0.863169909	6.099051476
H	2.429176807	3.452554464	4.556002617
H	4.840449810	3.100671053	5.162248611
H	-2.211983681	0.835742533	7.785329342
H	-3.216402531	-0.256697655	6.849403381
H	-2.754976034	0.967191517	4.771628857
C	-3.220621824	2.604971647	6.109655380
H	-2.727462769	3.133378744	6.937875271
H	-4.161167622	2.221084833	6.540747643
C	-3.560036898	3.600313187	4.990094662
H	-2.692250013	3.759027481	4.345932007
H	-4.346285820	3.194635391	4.343023777

C	-3.996095419	4.954648495	5.540205956
H	-4.998849392	4.885143280	5.989603996
H	-3.302433968	5.265186787	6.339963436
O	-3.976671457	5.906124592	4.469816685
H	-4.399000645	6.717042446	4.780456066
O	-0.370089650	3.892275333	4.486911297
C	-0.460413814	4.785823822	5.335414886
O	-0.479767263	4.525933743	6.637171268
H	-0.425963312	3.538893223	6.731186867
C	-0.624907911	6.227628708	4.972330570
H	-0.349934727	6.882033348	5.799984455
H	-0.032382011	6.447280407	4.082768917
H	-1.683777928	6.376035213	4.716979980

==============================

B5

==============================

C	-2.387285948	1.283724546	5.522317410
C	-2.261515856	0.441681594	6.804227829
C	-1.044322491	-0.491135448	6.754588127
O	-0.899181545	-1.470741153	7.490955830
N	-0.153781146	-0.075785354	5.813864708
Pd	-0.448740661	1.710753798	5.016365528
C	1.141020775	-0.711171567	5.657983780
H	1.352926016	-1.279951215	6.573844433
C	1.174560189	-1.755837560	4.468732357
C	1.008373260	-1.061807036	3.106018782
H	0.980857968	-1.809391141	2.304753780
H	0.076378331	-0.489804387	3.072391033
H	1.835250258	-0.374791086	2.894922495
C	2.501388311	-2.537984133	4.485099792
H	2.480106354	-3.329019070	3.727148771
H	3.361543894	-1.898490667	4.262641430
H	2.670374632	-3.016986370	5.457251072
C	0.014879411	-2.746987343	4.677387714
H	0.038945973	-3.183063507	5.679619789
H	-0.948424816	-2.244104385	4.563929081
H	0.072455399	-3.551162958	3.934797764
C	2.195935726	0.374162912	5.505563259
C	3.521436691	0.182128102	5.909945488
N	1.808584213	1.553220153	4.963232040
C	4.456685543	1.196621656	5.727016926
H	3.803202629	-0.756580114	6.373095989
C	2.711143017	2.529797792	4.789521217
C	4.048862457	2.397302151	5.145712376
H	5.486629486	1.055863976	6.042553425
H	2.327655315	3.453276634	4.363636494
H	4.741271019	3.217478275	4.987236500
H	-2.125514507	1.093790770	7.678502560
H	-3.161586285	-0.156258583	7.003087997
H	-2.745168447	0.642522156	4.707918644
C	-3.288970232	2.517844200	5.652599335
H	-4.099308491	2.294589996	6.363257408
H	-3.784202099	2.722555399	4.692512989
C	-2.585178852	3.812016726	6.115627766
H	-3.313632488	4.506093979	6.556166649
H	-1.852022171	3.586362600	6.900056839
C	-1.902705908	4.570933819	4.983911991
H	-2.639492750	4.826294899	4.211978912
H	-1.447265267	5.498416424	5.350007534
O	-0.840550423	3.785583973	4.368063450
H	-0.920826912	3.838727951	3.408773899

==============================

B6

==============================

C	-2.356601715	1.799936771	5.326676846
C	-2.646235466	0.302476257	5.274417877
C	-1.487948775	-0.579253554	5.768611431
O	-1.657188892	-1.661039352	6.327171803
N	-0.279570311	-0.054802082	5.436064243
Pd	-0.281777442	1.922226787	5.179493904
C	0.977719247	-0.767133117	5.658132553
H	0.847191215	-1.418095827	6.532536983
C	1.359704375	-1.701035976	4.432468414
C	1.590042472	-0.872648001	3.156979561
H	1.821141005	-1.541123629	2.319750547
H	0.692703843	-0.302656263	2.908132792
H	2.419561148	-0.167064399	3.262951374
C	2.628208399	-2.510809422	4.765402794
H	2.821574450	-3.236290693	3.968122959
H	3.515060902	-1.876952410	4.852487564
H	2.513207674	-3.072955132	5.700283527
C	0.212040320	-2.696721315	4.171594620
H	-0.049175892	-3.259636402	5.070816517
H	-0.690269530	-2.183996439	3.830879450
H	0.511919141	-3.400266171	3.386492968
C	2.085127115	0.231248975	5.946404934
C	3.202900887	-0.088020571	6.720482826
N	1.979043961	1.440320969	5.361896992
C	4.236083031	0.839581609	6.837703228
H	3.258020163	-1.051620245	7.214357376
C	2.975560188	2.328631163	5.454907894
C	4.136229038	2.062534094	6.174903393
H	5.112289429	0.606744647	7.436264515
H	2.809319973	3.266602039	4.940225124
H	4.925519466	2.804507017	6.230724335
H	-3.538465500	0.052349206	5.858332634
H	-2.829022884	0.001720313	4.237252712
H	-2.629434586	2.335783482	4.418416500
C	-2.824834585	2.516543865	6.575221539
H	-3.922096014	2.418809891	6.573432446
H	-2.608440876	3.587075949	6.486968994
C	-2.286086798	1.971076965	7.909704685
H	-2.963411808	2.253768921	8.724236488
H	-2.249965429	0.875868082	7.899635792
C	-0.913244069	2.529806137	8.258231163
H	-0.966759205	3.622581482	8.351726532
H	-0.566349387	2.115790606	9.212375641
O	0.088382185	2.212087154	7.275234699
H	0.608258247	3.090209246	7.086471558
O	-0.561725736	1.723174930	3.209856033
C	0.326672077	2.373552561	2.469655752
O	1.292243600	3.001714468	2.877019882
C	0.007999081	2.211181879	0.989381433
H	-1.035152316	2.471676588	0.791978836
H	0.151224405	1.165353775	0.700180769
H	0.674701452	2.848412991	0.406861126
O	-0.468089134	3.997953176	4.950524330
C	0.363161862	4.758226871	5.567692757
O	1.040948868	4.425964355	6.574031353
C	0.524260938	6.141506672	4.977503777
H	-0.450886428	6.558030605	4.714310646
H	1.099346638	6.038591385	4.050975323
H	1.052574754	6.798437595	5.669438362

===============================

B6'

===============================

C	-2.272240639	1.454467773	5.418814659
C	-2.243474245	0.505643129	6.606663227
C	-1.068630576	-0.478465289	6.518434525

O	-1.007615566	-1.512508988	7.174537182
N	-0.125509828	-0.095218465	5.599198341
Pd	-0.242527857	1.828763127	5.135082722
C	1.190184712	-0.743555844	5.560227871
H	1.302108049	-1.261946321	6.519642353
C	1.292768717	-1.833512783	4.413643360
C	1.504977703	-1.190005660	3.030312538
H	1.517235756	-1.968710303	2.258557081
H	0.697494864	-0.489151418	2.806013346
H	2.459313631	-0.648653746	2.973604918
C	2.465714931	-2.791226625	4.715948105
H	2.459802151	-3.616756678	3.995099306
H	3.440650463	-2.296666384	4.628728867
H	2.383377314	-3.226464510	5.721738338
C	-0.012656930	-2.657098293	4.391666412
H	-0.236128032	-3.076627970	5.377501965
H	-0.860081136	-2.032670736	4.090129375
H	0.080199324	-3.475814342	3.669243574
C	2.296332598	0.296705097	5.472599030
C	3.604717016	0.016383642	5.883056164
N	1.992089152	1.511724710	4.977675438
C	4.589626789	0.986617565	5.745748520
H	3.828387260	-0.939512730	6.339775562
C	2.934270859	2.457452774	4.857455730
C	4.257894516	2.233860016	5.210680485
H	5.605817318	0.779368699	6.069087505
H	2.592148066	3.415491581	4.475958824
H	4.997719288	3.018365145	5.092904091
H	-2.149943113	1.056591272	7.547858238
H	-3.174777269	-0.073824920	6.657781124
H	-2.451035023	0.904810071	4.494337082
C	-3.133313417	2.711043358	5.502361298
H	-3.956136942	2.489076853	6.197929859
H	-3.595016956	2.884835243	4.521659374
C	-2.471387863	4.029592991	5.958327293
H	-3.266288996	4.755181789	6.172086239
H	-1.903908253	3.877362490	6.878948212
C	-1.567299604	4.666236401	4.903590202
H	-2.108855009	4.761629581	3.950652838
H	-1.272763014	5.671364784	5.231154919
O	-0.353546888	3.925405979	4.670666218
H	-0.198340267	3.820857763	3.658689737
O	-0.565745115	1.288415313	3.148246288
C	-0.335282564	2.099553823	2.168730736
O	-0.062599443	3.318068266	2.265182018
C	-0.384701550	1.446674109	0.800981164
H	-1.158501983	0.674908698	0.770510018
H	0.581782877	0.960083723	0.620063841
H	-0.552457511	2.201642036	0.029109634
O	-0.124708332	2.456278801	7.057133198
C	0.661292017	1.969030619	7.997863770
O	1.383549571	0.987828732	7.928098679
C	0.557349622	2.813735962	9.266546249
H	-0.477870345	2.829443216	9.620710373
H	0.848610163	3.846235752	9.051859856
H	1.206647873	2.392342567	10.034802437

===============================

A6-TS

===============================

C	-1.995310426	1.753706455	5.750665665
C	-2.052937269	0.511091948	6.628648758
C	-1.130549431	-0.616453469	6.169442177
O	-1.245704889	-1.763274193	6.540742397
N	-0.094045214	-0.187256992	5.287419796
Pd	0.038812149	1.897179246	5.276574612

C	1.152251959	-1.001625180	5.319977760
H	1.032524109	-1.696202397	6.154488087
C	1.355103612	-1.872294545	4.016698360
C	1.922901392	-1.045515537	2.847558022
H	1.969024301	-1.666926861	1.945957303
H	1.310315013	-0.168000758	2.631945610
H	2.937765360	-0.696351707	3.063353539
C	2.336713552	-3.018725395	4.338266373
H	2.443147898	-3.669799566	3.464117765
H	3.334417582	-2.646356344	4.586578369
H	1.977871299	-3.633183718	5.171920300
C	0.007093234	-2.503598928	3.612179518
H	-0.463240445	-3.009356260	4.459915638
H	-0.697024345	-1.756809950	3.231712341
H	0.166910574	-3.233512163	2.811371803
C	2.346260786	-0.113014691	5.620234489
C	3.472445726	-0.617415488	6.276993275
N	2.295015097	1.168549538	5.214818954
C	4.571331501	0.212377504	6.475155830
H	3.468043804	-1.635354877	6.648561001
C	3.351392746	1.973170638	5.406442165
C	4.519531250	1.529262543	6.018439293
H	5.452130795	-0.161906525	6.989293575
H	3.216854811	2.989833117	5.053913116
H	5.355358124	2.208389997	6.149652004
H	-1.725425243	0.731247425	7.647983551
H	-3.074944019	0.116973221	6.687103748
C	-2.895180941	1.657656908	4.525076389
H	-3.883906364	2.003676891	4.860296249
H	-3.022940397	0.602572918	4.245244026
C	-2.461628199	2.454317093	3.288825035
H	-3.314557076	2.610436201	2.617849588
H	-2.083692551	3.434230328	3.591425896

C	-1.378005028	1.687028766	2.525954485
H	-1.826008677	0.824817479	2.011521339
H	-0.903805196	2.327077389	1.774134874
O	-0.349297613	1.179232359	3.373729944
H	-0.440766454	0.206103131	4.097860336
H	-2.097955704	2.694583416	6.289108276
O	0.433480591	2.467580557	7.177385330
C	0.800809026	1.603223801	8.095412254
O	0.779131770	0.379187435	7.999604225
C	1.297803283	2.306622982	9.352244377
H	0.660694063	3.158547163	9.600456238
H	2.306218147	2.688769579	9.161624908
H	1.330535412	1.594475627	10.178286552
O	-0.269943297	3.908581257	5.007918358
C	0.694505751	4.593015194	4.440998554
O	1.750398636	4.131714821	4.017255783
C	0.368856549	6.079960346	4.360596180
H	0.349840969	6.500616074	5.371343613
H	-0.621717095	6.231977940	3.922607660
H	1.128259182	6.589087963	3.764800072

================================

A7

================================

C	-2.003298044	1.733590007	5.658583641
C	-2.082743168	0.537379503	6.603325844
C	-1.132419348	-0.605732203	6.287368298
O	-1.217699528	-1.718702674	6.729433060
N	-0.067795075	-0.231654331	5.328826904
Pd	0.017698042	1.873292089	5.180344105
C	1.187108397	-1.046050310	5.409080505
H	1.057275772	-1.705595374	6.268937588
C	1.379370332	-1.948555589	4.136713028

C	1.741281748	-1.111371160	2.895477057
H	1.776550531	-1.760641098	2.013725758
H	1.020103097	-0.310499549	2.707242966
H	2.723052740	-0.642273486	3.007597208
C	2.506985426	-2.963103771	4.415449619
H	2.613593340	-3.637798786	3.559735298
H	3.470988512	-2.470277309	4.566015720
H	2.289839506	-3.575375557	5.298816204
C	0.080760323	-2.743698359	3.879588842
H	-0.259439796	-3.256942987	4.783556938
H	-0.738032997	-2.106566191	3.526029110
H	0.254844755	-3.489200592	3.097326756
C	2.369470835	-0.136092395	5.684048176
C	3.465821266	-0.594228566	6.419640541
N	2.329123497	1.108847618	5.181966782
C	4.556160450	0.250821829	6.603229046
H	3.447472334	-1.587321520	6.854370594
C	3.376320124	1.927985072	5.363536358
C	4.518718243	1.532675505	6.054747105
H	5.416659832	-0.083173610	7.176235676
H	3.252838373	2.921575069	4.947531700
H	5.346424580	2.223972082	6.175170898
H	-1.817813873	0.829068720	7.622876644
H	-3.099176645	0.125537053	6.631376266
C	-2.870291233	1.614654064	4.418304443
H	-3.903799295	1.769015670	4.767639160
H	-2.829074860	0.592023849	4.021880627
C	-2.549407005	2.626377583	3.313450098
H	-3.383454561	2.682637691	2.604788065
H	-2.430223942	3.615048409	3.764827967
C	-1.247769475	2.233288765	2.565388918
H	-1.507869244	1.670562267	1.654581547
H	-0.708724916	3.138456106	2.251086950
O	-0.411910772	1.372675180	3.297996759
H	-0.468513906	-0.279834867	4.379312992
H	-2.121820688	2.691059113	6.165179253
O	0.510912180	2.437598705	7.108030319
C	0.812105238	1.570464373	8.029578209
O	0.623475432	0.350105375	7.971038342
C	1.459309936	2.210163832	9.251514435
H	0.848811567	3.043146372	9.611198425
H	2.433451891	2.618214846	8.964273453
H	1.587067246	1.465916634	10.038647652
O	-0.308241785	3.859951496	4.928243160
C	0.688645065	4.570507050	4.455729961
O	1.769475460	4.131087303	4.079079151
C	0.366747051	6.059205055	4.437094212
H	0.474637181	6.454195976	5.452873230
H	-0.663416088	6.234983444	4.118486881
H	1.065936804	6.574484825	3.776837111

==============================

A7-TS

==============================

C	-2.340305567	1.348458767	5.317164898
C	-2.290280104	-0.146181583	5.388811111
C	-0.973711014	-0.703738153	5.943057060
O	-0.895380318	-1.369969249	6.947628975
N	0.165532663	-0.337191522	5.167659760
Pd	0.169411793	1.892289639	5.303278923
C	1.434020758	-1.080704331	5.406471729
H	1.332446337	-1.526552200	6.398359299
C	1.600737929	-2.252139091	4.372354031
C	1.790992260	-1.723391771	2.937767267
H	1.886886597	-2.566583157	2.245053053
H	0.943200052	-1.118222952	2.595396280

H	2.685639620	-1.103106618	2.851083755
C	2.831774950	-3.091442347	4.770133495
H	2.922643423	-3.956716299	4.105384827
H	3.757805824	-2.515359640	4.697599411
H	2.743061543	-3.468090534	5.795874119
C	0.356747091	-3.162407875	4.425655365
H	0.149969980	-3.498614788	5.446154594
H	-0.539485276	-2.655048609	4.051615715
H	0.510473371	-4.043806076	3.794773102
C	2.604966402	-0.114842154	5.415998459
C	3.699116230	-0.306665480	6.261374474
N	2.514740705	0.923491240	4.576952457
C	4.741527557	0.617491663	6.218870163
H	3.728618622	-1.155382156	6.937580109
C	3.506185293	1.823529601	4.555791855
C	4.644735813	1.705740929	5.352827549
H	5.606891155	0.497793317	6.864958763
H	3.345690966	2.675855637	3.902729273
H	5.425705910	2.457991123	5.304360867
H	-3.063148260	-0.474668324	6.096073627
H	-2.500846148	-0.583820999	4.407246590
C	-3.351322651	2.065809727	4.494914055
H	-4.100543022	2.476241112	5.188184261
H	-3.853742123	1.383310795	3.799547911
C	-2.680699110	3.226228714	3.736930847
H	-3.409472942	3.771929741	3.128543615
H	-2.230509758	3.920340300	4.451608658
C	-1.579634428	2.565206528	2.901648521
H	-2.016229153	2.109035492	1.999670386
H	-0.818427801	3.294510126	2.597060442
O	-0.974321425	1.534590602	3.651282787
H	-0.056689292	-0.245235920	4.175175190
H	-2.010679722	1.846936822	6.231816292
O	1.156883717	1.948812127	7.066642761
C	0.369044095	1.809748888	8.098751068
O	-0.857961595	1.669922829	8.054452896
C	1.125179887	1.838995218	9.418681145
H	1.450142622	2.864558220	9.622675896
H	2.021668673	1.216655612	9.354005814
H	0.477921963	1.495635629	10.226613045
O	-0.032202262	3.911193371	5.379256248
C	0.740564585	4.564840794	4.545978069
O	1.464103818	4.060463905	3.688109636
C	0.662408471	6.073802948	4.744875908
H	1.180289268	6.338318348	5.672589779
H	-0.376224279	6.399799347	4.844866753
H	1.142194748	6.580231190	3.905877590

==============================

A8

==============================

C	-2.450061083	1.524518847	4.256477833
C	-2.290323973	0.106994085	3.700260639
C	-1.171807170	-0.602575064	4.460147858
O	-1.327304244	-0.998684764	5.605005741
N	0.026678182	-0.687787890	3.784920931
Pd	0.234910324	1.949391484	5.794970036
C	1.304860115	-0.813359082	4.513366699
H	1.080348372	-0.591124773	5.563376427
C	1.875802517	-2.270084381	4.504888535
C	1.987242460	-2.880670071	3.092854977
H	2.220168114	-3.947926283	3.169986963
H	1.036350608	-2.785255194	2.559137106
H	2.768746614	-2.432964802	2.473394394
C	3.227651119	-2.303281307	5.248476505
H	3.560496569	-3.339196920	5.373747826

H	4.021414757	-1.763860106	4.726115704
H	3.128763199	-1.862883925	6.247586250
C	0.881338418	-3.145988464	5.302234650
H	0.727008879	-2.758611679	6.312954426
H	-0.097297840	-3.183132887	4.819032669
H	1.268521190	-4.168339252	5.374785900
C	2.182271719	0.344214618	4.028465748
C	3.255057812	0.223520890	3.141950607
N	1.822477221	1.576560259	4.467783451
C	3.962260485	1.351812601	2.732210159
H	3.543151617	-0.750359833	2.777899027
C	2.509290934	2.669708490	4.089750290
C	3.587238550	2.600042105	3.215097666
H	4.798877716	1.248702645	2.047192335
H	2.196567059	3.602504730	4.540441036
H	4.116681576	3.504966021	2.938699245
H	-3.221359491	-0.441091031	3.872028828
H	-2.103620052	0.151573539	2.620787382
C	-3.464580059	2.410155296	3.530004978
H	-4.464358807	2.306356192	3.959984541
H	-3.520077229	2.137232304	2.468631744
C	-2.873306274	3.818202257	3.697213411
H	-3.248879433	4.532482624	2.959224463
H	-3.062828302	4.209683418	4.699772835
C	-1.377682447	3.564579964	3.549021959
H	-1.057963133	3.535287857	2.499224186
H	-0.771187723	4.261515617	4.125319481
O	-1.166295886	2.228309631	4.104207516
H	0.070190884	-0.188390210	2.907859564
H	-2.654033184	1.474182725	5.330368042
O	1.451771855	1.405537486	7.304455757
C	2.466691494	2.200256586	7.550251007
O	2.746471405	3.228737593	6.938714504
C	3.313468218	1.689278722	8.711894035
H	3.726392984	0.706097603	8.463576317
H	2.690922022	1.566054463	9.602393150
H	4.124084473	2.391006947	8.913028717
O	-1.234960556	2.309367418	7.131985664
C	-1.640485048	3.550902843	7.194442749
O	-1.301565051	4.458050728	6.428940296
C	-2.608959675	3.793277264	8.344106674
H	-2.053018332	3.765928745	9.286876678
H	-3.361358881	3.001166344	8.386982918
H	-3.083670855	4.769397259	8.232733727

===============================

A6-TS'

===============================

C	-2.003526926	1.713183522	5.655016899
C	-2.134063005	0.526210010	6.607536316
C	-1.098252177	-0.569312871	6.317789555
O	-1.190955162	-1.710390687	6.752776623
N	-0.082981683	-0.144973993	5.496855736
Pd	0.066592343	1.822193384	5.374264717
C	1.151609659	-0.936215937	5.407641411
H	1.132054210	-1.617628932	6.264158726
C	1.236570358	-1.829564214	4.102453709
C	1.728655577	-1.022962570	2.884922743
H	1.586949348	-1.607158899	1.968153358
H	1.180854201	-0.084360585	2.784593344
H	2.794326305	-0.778726280	2.964510679
C	2.198818207	-3.004736662	4.361660480
H	2.169840336	-3.700515747	3.515800238
H	3.236149549	-2.674479008	4.479427338
H	1.910580158	-3.566109896	5.258057594
C	-0.160167277	-2.411134243	3.805748701

H	-0.548421681	-2.968259096	4.662376404
H	-0.873657107	-1.617779016	3.570030689
H	-0.101956494	-3.085038185	2.943199396
C	2.375103235	-0.053415742	5.557818413
C	3.601527452	-0.574514806	5.984123707
N	2.263777256	1.250204682	5.241600990
C	4.716724873	0.253315002	6.032886028
H	3.661886215	-1.608670115	6.302704334
C	3.332378149	2.056247950	5.301438332
C	4.590701103	1.597744703	5.670884132
H	5.674235821	-0.141223714	6.362443924
H	3.155267715	3.096791267	5.043323040
H	5.437306881	2.276259422	5.695774078
H	-1.983775616	0.838432372	7.644084454
H	-3.130857944	0.072772868	6.542361259
C	-2.796840906	1.518063307	4.374282837
H	-3.827992678	1.817780375	4.619816780
H	-2.837401867	0.446635962	4.139365196
C	-2.342610598	2.269831419	3.116026163
H	-3.144654036	2.233303547	2.368170261
H	-2.146209240	3.327298164	3.333079576
C	-1.094769239	1.632140279	2.505280018
H	-1.251881003	0.554769576	2.368309259
H	-0.897742033	2.068346500	1.515156746
O	0.076723509	1.818400979	3.282046080
H	0.495837897	2.906664610	3.161792755
H	-2.161803961	2.687860012	6.120895863
O	0.125184298	2.176189661	7.365038395
C	0.768684864	1.446220636	8.253628731
O	1.381157875	0.407528341	8.056900024
C	0.653915524	2.071543694	9.641538620
H	-0.386454374	2.029989719	9.979168892
H	0.952236891	3.123183489	9.610254288
H	1.285820723	1.517440319	10.336924553
O	0.135564834	3.975627661	5.296300411
C	0.518226087	4.614267349	4.272816658
O	0.783619463	4.085479736	3.141154528
C	0.696613610	6.108173370	4.404383183
H	1.730509281	6.307761669	4.708267212
H	0.032334436	6.501501083	5.175508022
H	0.521671116	6.598427773	3.445125103

================================

A7'

================================

C	-1.992259741	1.713868260	5.647250652
C	-2.115458012	0.544905841	6.620986462
C	-1.091968417	-0.559167266	6.326161861
O	-1.189737797	-1.699372172	6.762456894
N	-0.076269984	-0.143678203	5.498857975
Pd	0.079122394	1.822122931	5.357459068
C	1.154086590	-0.944283307	5.407551765
H	1.123920321	-1.631709218	6.259897709
C	1.218991995	-1.824742079	4.090476513
C	1.712211967	-1.005560994	2.884927750
H	1.598620653	-1.589124799	1.964087009
H	1.153976440	-0.072764620	2.784420252
H	2.772478342	-0.746274590	2.983872175
C	2.169407606	-3.018239975	4.317738056
H	2.140071869	-3.685401440	3.449093103
H	3.209307909	-2.702762604	4.442494869
H	1.875164390	-3.603044033	5.197037697
C	-0.185800403	-2.387145042	3.797679901
H	-0.589207768	-2.914365530	4.665954590
H	-0.883406341	-1.585646868	3.541621685
H	-0.137374505	-3.080976963	2.950998306

C	2.384114265	-0.068755023	5.574522495
C	3.602894783	-0.598142326	6.015437126
N	2.284779310	1.238973260	5.270833492
C	4.718114376	0.225514978	6.099957943
H	3.656441450	-1.635973930	6.319694042
C	3.353662252	2.040113449	5.367941380
C	4.602005005	1.574957132	5.758343697
H	5.666959763	-0.175702557	6.444563389
H	3.182900190	3.085464716	5.126049995
H	5.447906971	2.251657248	5.814907074
H	-1.944304585	0.872569323	7.648861885
H	-3.115997076	0.097727828	6.578953743
C	-2.788266420	1.488158226	4.373768806
H	-3.820588589	1.787131667	4.613173962
H	-2.822096348	0.411542743	4.162856579
C	-2.340754986	2.208801746	3.096290827
H	-3.136838913	2.132324934	2.345273495
H	-2.171153307	3.276990891	3.281659126
C	-1.070786595	1.583861470	2.518588781
H	-1.206427932	0.498881936	2.407676697
H	-0.880736053	1.992802262	1.514523387
O	0.088733293	1.833477736	3.289569616
H	0.447418600	3.060285091	3.127552271
H	-2.153643370	2.695964575	6.095254898
O	0.150342643	2.181842566	7.353515625
C	0.771596432	1.439447284	8.244832993
O	1.336826324	0.372019470	8.055827141
C	0.681460917	2.073305607	9.631268501
H	-0.342662573	1.973462343	10.005747795
H	0.914862692	3.139853716	9.584758759
H	1.364654779	1.558676958	10.308049202
O	0.172330722	4.004478931	5.289094925
C	0.476247787	4.668419361	4.266547680
O	0.666781306	4.163151741	3.097060919
C	0.670539796	6.158576488	4.387997150
H	1.744659305	6.363767624	4.457123756
H	0.182792470	6.533411503	5.287952900
H	0.290541738	6.660714626	3.495864868

================================

A7'-TS

================================

C	-2.629647732	1.182486534	5.241883755
C	-2.401315689	0.188402817	6.330795288
C	-1.209463000	-0.733388603	5.978951454
O	-1.224135876	-1.934141517	6.222540379
N	-0.188993841	-0.072341874	5.346312523
Pd	-0.231253490	1.940008998	5.514327049
C	1.088547707	-0.804578900	5.223116875
H	1.029765248	-1.611876130	5.960898876
C	1.324355841	-1.511904836	3.825253725
C	1.884907246	-0.542538822	2.766933918
H	1.874043107	-1.020952344	1.780935884
H	1.304569483	0.380329460	2.711698294
H	2.918450832	-0.257972836	2.986693859
C	2.340475321	-2.655444145	4.031182766
H	2.524473667	-3.170344114	3.081565380
H	3.304812193	-2.286015034	4.394706249
H	1.964274526	-3.396836758	4.745469093
C	0.007426529	-2.130862474	3.322760820
H	-0.434109181	-2.782720089	4.079296589
H	-0.725102127	-1.357893109	3.078823090
H	0.195481673	-2.714242935	2.414215565
C	2.275541306	0.064491674	5.596061230
C	3.322321653	-0.455732554	6.365891933
N	2.301332474	1.328083396	5.136943817

C	4.432487488	0.342163205	6.637560368
H	3.258235931	-1.469340563	6.747472286
C	3.359400272	2.094877005	5.417862892
C	4.456940174	1.649157166	6.153781891
H	5.257383823	-0.047406252	7.228024006
H	3.323207855	3.113629341	5.038709641
H	5.296429157	2.309854984	6.345225334
H	-2.196379900	0.636955500	7.303908348
H	-3.284657001	-0.461670190	6.409979820
C	-3.092421055	0.590641320	3.948015451
H	-4.191666126	0.574189961	4.058351517
H	-2.769969225	-0.454421490	3.901868820
C	-2.735385656	1.304896712	2.635508776
H	-2.997790337	0.664461911	1.785148740
H	-3.295338869	2.242540121	2.546920061
C	-1.242353916	1.633602858	2.661842346
H	-0.645495176	0.714056492	2.664665937
H	-0.967923403	2.194502354	1.752410889
O	-0.992766321	2.455392122	3.752755642
H	1.303932309	3.224509001	7.315206051
H	-2.937383413	2.197583199	5.474693298
O	0.680223286	1.910678148	7.405344486
C	0.585281134	1.075751185	8.429047585
O	-0.330758244	0.290097624	8.601439476
C	1.765001059	1.191934586	9.382842064
H	1.890841246	2.229551315	9.709222794
H	2.678169727	0.900965214	8.855362892
H	1.610755324	0.545824111	10.247584343
O	-0.261454672	4.108260155	5.754958153
C	0.621536434	4.750179291	6.353231907
O	1.530113697	4.225027084	7.130986691
C	0.722109854	6.243998051	6.196786404
H	0.646202981	6.720849514	7.178349495
H	-0.059727225	6.609215260	5.532378197
H	1.707431674	6.492159843	5.789516926

================================

B6-TS

================================

C	-2.350430727	1.862686753	5.416160583
C	-2.633189440	0.363277406	5.521720409
C	-1.494198680	-0.543751001	5.952641964
O	-1.640533328	-1.679018617	6.351966381
N	-0.216769904	0.077626429	5.856378555
Pd	-0.256606102	2.076916218	5.235819340
C	1.002090096	-0.762315929	5.727170944
H	0.897036791	-1.560247898	6.470293999
C	1.234419942	-1.470355511	4.325016975
C	1.934642196	-0.548894405	3.305127144
H	1.980100989	-1.058787942	2.336244822
H	1.409542918	0.397083640	3.174200535
H	2.962034225	-0.319285482	3.603247643
C	2.145033360	-2.689265728	4.584807873
H	2.350852251	-3.206634998	3.641848087
H	3.109223843	-2.394340277	5.011392117
H	1.670544028	-3.407166243	5.263717175
C	-0.087189078	-1.972511888	3.716967344
H	-0.606781840	-2.659938097	4.387119770
H	-0.759376347	-1.148129582	3.464833021
H	0.130994111	-2.506000519	2.785321236
C	2.179809332	0.124006063	6.087519169
C	3.275892496	-0.351794451	6.807238102
N	2.130453587	1.387473345	5.624080181
C	4.363324165	0.496022552	7.017458439
H	3.272746086	-1.366312027	7.191737175
C	3.168506145	2.204758883	5.831969738

C	4.315184593	1.793129563	6.512919426
H	5.231217384	0.146833271	7.569937706
H	3.046346188	3.207676411	5.437695980
H	5.140681744	2.483875513	6.651154041
H	-3.487260103	0.163774893	6.178376198
H	-2.876219511	0.011572827	4.513971329
H	-2.626191139	2.262334347	4.444182873
C	-2.855540752	2.715786219	6.557288170
H	-3.953677654	2.628454924	6.511075497
H	-2.615718842	3.765013695	6.359935760
C	-2.384069920	2.318164587	7.963039875
H	-2.974029541	2.858373880	8.713062286
H	-2.552910805	1.248033166	8.143169403
C	-0.909296513	2.647615433	8.183050156
H	-0.743395925	3.725652695	8.059317589
H	-0.604252696	2.370852709	9.201730728
O	-0.032463264	1.973652959	7.294264793
H	-0.158700481	0.781542897	6.906581879
O	-0.146226510	2.074250460	3.222419024
C	-0.952790260	1.472844481	2.384582520
O	-1.945856690	0.805850387	2.664734125
C	-0.488700807	1.660002708	0.945440233
H	0.419778317	1.068726301	0.790375113
H	-0.237227276	2.705854654	0.754825592
H	-1.268162131	1.318440676	0.263257563
O	-0.593928993	4.079476357	5.104438782
C	0.421394289	4.825230122	4.729782581
O	1.566459894	4.431775093	4.543202400
C	0.017212097	6.280891418	4.535055161
H	-0.526916623	6.646389961	5.410475731
H	-0.652933300	6.358051777	3.673042536
H	0.907449961	6.887372017	4.362952709

================================

B7

================================

C	-2.359063148	1.893539429	5.524950504
C	-2.644064188	0.430946469	5.859982014
C	-1.549705863	-0.590525925	5.596333504
O	-1.731233716	-1.770971060	5.463703156
N	-0.215327337	-0.008259121	5.710235119
Pd	-0.274936020	2.082752228	5.276018620
C	1.024040699	-0.852984965	5.521387577
H	0.865802348	-1.739779830	6.145720005
C	1.408965945	-1.387033820	4.084675789
C	1.994619370	-0.292918056	3.168680191
H	2.145102262	-0.714377463	2.168548107
H	1.345797181	0.576916218	3.083458185
H	2.966329098	0.059335846	3.525334597
C	2.506012440	-2.456581116	4.311582088
H	2.805799961	-2.874432325	3.345292568
H	3.402956247	-2.041671991	4.780584812
H	2.141981602	-3.285182476	4.930972099
C	0.235625699	-2.085224152	3.371746778
H	-0.170155451	-2.900745153	3.973577738
H	-0.577653289	-1.398990989	3.138691664
H	0.603875339	-2.506201506	2.428906441
C	2.143018246	-0.000967022	6.102022171
C	3.118151188	-0.522531569	6.952069283
N	2.141193867	1.288661599	5.722744465
C	4.140880108	0.317767620	7.392975330
H	3.076455355	-1.563957334	7.254367352
C	3.116565228	2.095971584	6.150791645
C	4.146627426	1.647502780	6.979259968
H	4.916510105	-0.062250905	8.052008629
H	3.041829586	3.123425484	5.811354637

H	4.924361706	2.333846092	7.298436165
H	-2.894485474	0.319433033	6.922387600
H	-3.508051395	0.091764227	5.281999588
H	-2.641595125	2.130021811	4.503919601
C	-2.874444485	2.911640167	6.518133640
H	-3.967856884	2.765378475	6.540647984
H	-2.699059963	3.913437128	6.118251801
C	-2.328644276	2.821677685	7.951228619
H	-2.833987236	3.571220160	8.573091507
H	-2.552029371	1.842599988	8.394639015
C	-0.814411223	3.049517393	8.000218391
H	-0.581188202	4.064434052	7.644437313
H	-0.468626112	2.977747679	9.043572426
O	-0.093204275	2.085918665	7.278419971
H	-0.181670099	0.325896144	6.696766376
O	-0.323448867	2.070064545	3.224555731
C	-1.102117777	1.260589242	2.564043283
O	-1.876623392	0.433943063	3.059050560
C	-0.945621908	1.400430441	1.057056189
H	0.044982649	1.032797694	0.769965589
H	-1.001942158	2.452237368	0.764785945
H	-1.714298129	0.818235397	0.547374785
O	-0.560334682	4.077784061	5.135666847
C	0.509410560	4.757473469	4.782209396
O	1.632804990	4.288756847	4.643684864
C	0.200759023	6.225615501	4.527922153
H	-0.581698000	6.590027332	5.197021008
H	-0.155042902	6.332395077	3.497440100
H	1.113030553	6.813016891	4.645432949

================================

B7-TS

================================

C	-2.495129824	1.537709117	6.022094250
C	-2.475475550	0.142717540	6.571902275
C	-1.340334892	-0.755996883	6.059984207
O	-1.500615835	-1.942925096	5.882402420
N	-0.093329683	-0.095194489	5.862343311
Pd	-0.213476315	2.144819736	5.121251106
C	1.116200805	-0.938762307	5.617834091
H	1.008647680	-1.829729438	6.246973038
C	1.351521850	-1.449708462	4.130642414
C	2.220788240	-0.474799752	3.307559013
H	2.339383602	-0.878292680	2.295407534
H	1.770081520	0.514596879	3.231297493
H	3.223437548	-0.365504116	3.732293129
C	2.108363628	-2.790377140	4.245768070
H	2.310326338	-3.190317869	3.246697426
H	3.074436903	-2.670003176	4.748140335
H	1.522429109	-3.536411762	4.794616699
C	0.032959763	-1.702316880	3.375856400
H	-0.543175638	-2.507992506	3.831789494
H	-0.600142360	-0.813463688	3.331662178
H	0.267296880	-1.991253853	2.345178843
C	2.295596123	-0.113263667	6.110670567
C	3.421684027	-0.693842888	6.698422909
N	2.202379465	1.206882000	5.891832352
C	4.498410225	0.130466357	7.028573513
H	3.453270197	-1.762826800	6.885307312
C	3.228991747	1.998651743	6.217844009
C	4.407057762	1.499538302	6.778241634
H	5.393650532	-0.292211622	7.477094650
H	3.076712370	3.054288387	6.009272099
H	5.225429058	2.170877695	7.019309044
H	-2.447088480	0.141389847	7.666501999
H	-3.399851561	-0.360214591	6.259396076

H	-2.435674906	1.563938856	4.928394318
C	-3.328781605	2.626964331	6.618707657
H	-4.365313053	2.260200024	6.665346622
H	-3.307420731	3.482360840	5.935625076
C	-2.811955214	3.041551590	7.997155666
H	-3.302336693	3.953860521	8.350811005
H	-3.000100851	2.252284288	8.734720230
C	-1.307035089	3.237365723	7.795547009
H	-1.106010318	4.163510799	7.245894432
H	-0.776387751	3.268772125	8.756896973
O	-0.826154172	2.117141724	7.078318119
H	0.064612404	0.461385906	6.710793972
O	0.193802476	2.031796455	3.156537533
C	-0.813412011	1.612374663	2.439526558
O	-1.919989347	1.278721571	2.881204367
C	-0.475443512	1.498846769	0.962172747
H	0.166477919	0.622281253	0.821051657
H	0.083714053	2.374905348	0.625427723
H	-1.388276100	1.374199986	0.378320575
O	-0.670648515	4.095853806	4.858191013
C	0.386415362	4.880723000	4.878352165
O	1.532387495	4.534795284	5.146939754
C	0.044755235	6.317509174	4.506731033
H	-0.934230387	6.606396198	4.896340847
H	0.005482496	6.393528938	3.414833307
H	0.822336376	6.986299992	4.879335403

================================

B8

================================

C	-2.344784021	1.370346546	6.643861294
C	-2.392231464	0.029804155	7.393805027
C	-1.454759836	-1.043789983	6.812042236
O	-1.833298683	-2.191137552	6.610671997
N	-0.212739259	-0.563583553	6.534565926
Pd	0.235345542	2.265303850	5.019360542
C	0.869496942	-1.191158414	5.785817623
H	1.182072759	-2.091739655	6.327002525
C	0.512967885	-1.683070064	4.303904533
C	1.725586534	-1.496615529	3.367341757
H	1.495090485	-1.943096995	2.394814968
H	1.945468664	-0.442016900	3.188188553
H	2.621595860	-1.996577382	3.753468037
C	0.184699491	-3.189816475	4.376944542
H	-0.106794208	-3.554308414	3.385727644
H	1.061780691	-3.768217564	4.695637226
H	-0.631986201	-3.378018379	5.075642586
C	-0.687128067	-0.923734069	3.714572668
H	-1.604778647	-1.133776665	4.267671585
H	-0.509693801	0.152277559	3.665798903
H	-0.839950860	-1.253358126	2.681699753
C	2.061774492	-0.240620673	5.856561661
C	3.317762852	-0.789060473	6.142957687
N	1.934510946	1.095378518	5.650947094
C	4.447046280	0.015981637	6.205914974
H	3.391989470	-1.858183265	6.307981491
C	3.036041021	1.879207850	5.714375019
C	4.302057743	1.384552360	5.988674164
H	5.419291496	-0.415725768	6.424564838
H	2.879606485	2.930474281	5.507116795
H	5.146870136	2.063441277	6.027197361
H	-2.155084372	0.192800075	8.452628136
H	-3.401922703	-0.384534657	7.339471340
H	-2.467441082	1.216171861	5.570049763
C	-3.290487289	2.444917679	7.179969311
H	-4.248119354	2.030409336	7.505429268

H	-3.476732254	3.177687168	6.388949394
C	-2.486193895	3.087252617	8.323004723
H	-2.830258131	4.096163750	8.563673973
H	-2.551941156	2.480547428	9.232276917
C	-1.062916636	3.101565361	7.765069962
H	-0.839803159	4.005990028	7.196053505
H	-0.287650526	2.924591541	8.515722275
O	-1.010327816	1.972606182	6.820287228
H	-0.054479148	0.385587782	6.838360786
O	1.427155137	2.998107195	3.582172155
C	1.419653654	2.427970648	2.396874905
O	0.803644240	1.416999340	2.085906744
C	2.311936855	3.177445173	1.411746264
H	3.350548506	3.150578499	1.757387877
H	2.012337446	4.227737904	1.357445240
H	2.239300489	2.712497473	0.427825600
O	-1.319869161	3.269370556	4.249617577
C	-1.388616800	4.549098492	4.526308537
O	-0.696523786	5.143444538	5.352571964
C	-2.443808794	5.263134956	3.692679644
H	-3.353757620	4.661684990	3.618939638
H	-2.057730198	5.396357536	2.676732063
H	-2.661786079	6.240229607	4.126223564

================================

B6-TS'

================================

C	-2.338901281	1.739687443	5.422528267
C	-2.612073898	0.237879932	5.464420319
C	-1.412032127	-0.626473248	5.865865707
O	-1.523792386	-1.771497011	6.301932812
N	-0.226363212	-0.027462551	5.592640400
Pd	-0.272895068	1.903912902	5.198668957
C	1.041945577	-0.744713724	5.644869328
H	0.953165174	-1.469762206	6.461579800
C	1.390671134	-1.577405453	4.317050457
C	2.290386438	-0.786281526	3.341183901
H	2.411688328	-1.360406995	2.415626287
H	1.864628553	0.187503129	3.085987806
H	3.292033434	-0.609651864	3.752462864
C	2.133867264	-2.853838682	4.760111332
H	2.426559448	-3.446430683	3.885992527
H	3.052075624	-2.616194248	5.313512802
H	1.498116970	-3.480335712	5.398378372
C	0.103412770	-1.971082687	3.565871716
H	-0.573691905	-2.546708822	4.201716423
H	-0.417359084	-1.077363014	3.205980778
H	0.366285026	-2.587897301	2.698148251
C	2.156825781	0.229936048	5.946521759
C	3.300535917	-0.129571274	6.661283493
N	2.041229010	1.449523330	5.386940479
C	4.352461338	0.780028343	6.755855083
H	3.359227657	-1.106036663	7.130138874
C	3.052555561	2.318642378	5.463827610
C	4.238719463	2.021684647	6.130842686
H	5.252193928	0.519783616	7.306486607
H	2.890176773	3.271902800	4.973577499
H	5.041648388	2.750394106	6.167498589
H	-3.441606522	0.004281651	6.140468121
H	-2.891621113	-0.112727344	4.464947224
H	-2.644112349	2.213442564	4.489963055
C	-2.785482883	2.513454676	6.642681122
H	-3.874524593	2.360335588	6.706895828
H	-2.632071972	3.587060690	6.481677055
C	-2.142139196	2.081439972	7.970997334
H	-2.779004335	2.388116837	8.809424400

H	-2.053384304	0.990104079	8.021858215
C	-0.770888209	2.721941710	8.176292419
H	-0.903042853	3.815770149	8.245953560
H	-0.351815253	2.379415751	9.133275986
O	0.179945007	2.426693678	7.164853096
H	0.694017351	3.542254448	6.784900665
O	-0.617643058	1.472645521	3.252517462
C	0.223368987	2.068231821	2.425787210
O	1.181435585	2.761746883	2.749947071
C	-0.111593068	1.754564643	0.974035144
H	-1.164609551	1.966507435	0.769814789
H	0.053202778	0.687868536	0.790417373
H	0.529170454	2.344109774	0.316706508
O	-0.488749981	3.992938280	4.764662266
C	0.330484957	4.795314312	5.284657478
O	1.003878713	4.548957348	6.354998589
C	0.578920960	6.110506535	4.599545002
H	-0.365789860	6.530215740	4.246655464
H	1.198500633	5.904069901	3.719153643
H	1.095341802	6.808898449	5.259478569

================================

B7'

================================

C	-2.281167746	1.876134634	5.290477276
C	-2.632124424	0.392430395	5.234425068
C	-1.504245281	-0.514198422	5.743125439
O	-1.696045876	-1.562317967	6.353481770
N	-0.282903939	-0.061726697	5.339725018
Pd	-0.206118241	1.914144397	5.111198425
C	0.943429530	-0.804972529	5.633676052
H	0.774780035	-1.400992751	6.539905071
C	1.292569518	-1.815683126	4.459591866
C	1.577138662	-1.054706335	3.152842045
H	1.751510620	-1.768423796	2.339984179
H	0.725641191	-0.424424797	2.886581421
H	2.460168600	-0.414321810	3.231664419
C	2.514426947	-2.671070337	4.846199989
H	2.685549736	-3.435837746	4.081468105
H	3.429691792	-2.078293562	4.925191402
H	2.356095314	-3.189379692	5.799777031
C	0.099573508	-2.763965607	4.230695724
H	-0.198339015	-3.269455194	5.152359486
H	-0.770733237	-2.223179102	3.851952076
H	0.373194158	-3.518637180	3.484514952
C	2.092347860	0.154235259	5.878476143
C	3.180265665	-0.175665781	6.689845562
N	2.057972670	1.325425506	5.215730190
C	4.260412693	0.700841725	6.766845226
H	3.175094128	-1.107476711	7.244385242
C	3.100719690	2.161487341	5.270593643
C	4.235698223	1.882620573	6.025907993
H	5.114246368	0.460402638	7.394237518
H	2.994895458	3.066989660	4.684447289
H	5.064352512	2.582516432	6.046607494
H	-3.535676241	0.177074000	5.814054489
H	-2.814714909	0.096071444	4.196218967
H	-2.540256023	2.424991369	4.385193825
C	-2.707952499	2.593753815	6.551829338
H	-3.805950165	2.510220289	6.583635330
H	-2.480154991	3.662732363	6.467599392
C	-2.127657890	2.034304380	7.861775875
H	-2.750021219	2.355215311	8.705739021
H	-2.139797926	0.938800275	7.860125065
C	-0.709575355	2.539401054	8.116425514
H	-0.745125353	3.638018608	8.221555710

H	-0.341956615	2.129428148	9.068039894
O	0.237715319	2.189988613	7.118518353
H	0.896336555	3.340096951	6.823754311
O	-0.482607782	1.708637714	3.116197348
C	0.412030369	2.350862741	2.389170885
O	1.347211838	3.023490429	2.812011957
C	0.181095153	2.128919363	0.900856614
H	-0.880155325	2.206004858	0.654511213
H	0.511642158	1.118095040	0.640690804
H	0.761045694	2.855122089	0.330185205
O	-0.295796394	4.049734116	4.900244236
C	0.596831918	4.726150513	5.468004704
O	1.266483188	4.323198318	6.497405529
C	0.952823520	6.071660519	4.904026508
H	0.044520430	6.608083248	4.621768951
H	1.527152419	5.889627457	3.988933325
H	1.547967196	6.652905941	5.608586788

==============================

B7'-TS

==============================

C	-2.454296589	1.567793250	5.734217644
C	-2.532495499	0.148412496	6.176336765
C	-1.235158443	-0.683961511	6.056503773
O	-1.246899128	-1.795355082	6.588230133
N	-0.183911845	-0.172443852	5.351094246
Pd	-0.134588227	1.837946057	5.007246971
C	1.080578566	-0.932446480	5.501165867
H	0.923416495	-1.604989171	6.350471497
C	1.400899887	-1.873613238	4.281126499
C	1.650901914	-1.081956267	2.991903782
H	1.871038437	-1.771601796	2.167657614
H	0.768663943	-0.494964510	2.732922316
H	2.495200157	-0.393897206	3.094672918
C	2.644851685	-2.718886852	4.619942188
H	2.832880259	-3.443607092	3.820327282
H	3.542810440	-2.102499962	4.726963520
H	2.506143808	-3.281397104	5.551311016
C	0.206957430	-2.821831226	4.058047295
H	-0.028947555	-3.390534639	4.960950851
H	-0.688025415	-2.259069204	3.778223038
H	0.435418308	-3.521713734	3.245699167
C	2.234723330	-0.017808396	5.869871140
C	3.151600122	-0.371544123	6.866823196
N	2.345787048	1.136677504	5.196043968
C	4.209878445	0.489767015	7.149887562
H	3.031666040	-1.305944324	7.405534744
C	3.360445499	1.964699388	5.468416691
C	4.324165821	1.682792306	6.435300827
H	4.931731701	0.235563904	7.921451569
H	3.378882408	2.876563787	4.877337933
H	5.133515358	2.381020308	6.625078678
H	-2.829968214	0.071480803	7.223778725
H	-3.290898800	-0.372120678	5.573936939
H	-2.438672304	1.673902869	4.649064541
C	-3.228704453	2.678396225	6.393045902
H	-4.279509544	2.357286692	6.436364174
H	-3.188987494	3.567564487	5.754160881
C	-2.690890551	3.014319897	7.788973331
H	-3.114862919	3.949905157	8.168313980
H	-2.938964605	2.216179132	8.496544838
C	-1.171755433	3.082045078	7.610414982
H	-0.874682724	3.989221573	7.069661617
H	-0.650661409	3.066051722	8.577105522
O	-0.811731696	1.902763009	6.927692413
H	-0.839677393	2.948600769	2.770888805

O	0.095358513	1.722589016	2.890382290
C	1.202247858	2.258708954	2.363207340
O	1.906397820	3.084936619	2.922887802
C	1.468154311	1.783342361	0.945276499
H	0.564774454	1.888580799	0.336771876
H	1.728434324	0.721591651	0.958228767
H	2.285843611	2.358395576	0.508559167
O	-0.295382798	3.983054399	4.827760220
C	-0.789456129	4.540552616	3.831624031
O	-1.212219357	3.907567978	2.768901348
C	-0.930997491	6.034352779	3.776930332
H	-0.910813332	6.454465866	4.782254696
H	-1.846810460	6.311962605	3.250882626
H	-0.082099289	6.430076122	3.208399773

===============================

B7"

===============================

C	-2.288535833	1.477793217	5.460558891
C	-2.254429579	0.483493507	6.610611439
C	-1.070346832	-0.495866835	6.517447472
O	-1.009526134	-1.520892501	7.190683842
N	-0.136874035	-0.103894301	5.596895218
Pd	-0.260630637	1.851808906	5.161129951
C	1.175469756	-0.757604718	5.552395821
H	1.313304305	-1.268864989	6.513001919
C	1.253700614	-1.862358928	4.420202732
C	1.270292640	-1.233139277	3.017471313
H	1.322959185	-2.019627094	2.255515814
H	0.363550186	-0.650229573	2.853368521
H	2.135150194	-0.573736370	2.877995491
C	2.519149065	-2.724777222	4.602295876
H	2.489554167	-3.572947025	3.909515858
H	3.436004639	-2.167782307	4.389005184
H	2.587076187	-3.130371332	5.618639469
C	0.023673370	-2.783107996	4.539765835
H	-0.059704453	-3.209243059	5.542128086
H	-0.897749543	-2.230535269	4.339302540
H	0.098546565	-3.596555710	3.808854818
C	2.282415628	0.282466769	5.433976650
C	3.596572399	0.008426364	5.831726551
N	1.970181584	1.499364734	4.947212219
C	4.573140144	0.988150239	5.703109741
H	3.831869125	-0.949625075	6.277709007
C	2.902665615	2.456412554	4.842039585
C	4.228857994	2.241145134	5.190171242
H	5.591910362	0.785024524	6.021090508
H	2.545161247	3.419278860	4.486424446
H	4.960148811	3.035903454	5.088280678
H	-2.182720423	1.003965259	7.570619583
H	-3.182361603	-0.104038157	6.630494118
H	-2.469687939	0.962980926	4.515452862
C	-3.140324831	2.735514641	5.600580692
H	-3.887824059	2.541211843	6.382798672
H	-3.696154356	2.881977081	4.665264606
C	-2.436980963	4.069257259	5.938337803
H	-3.217782736	4.826478004	6.087574482
H	-1.875424147	3.979640245	6.870456696
C	-1.502043009	4.576787472	4.831583500
H	-2.063975573	4.586163521	3.876702070
H	-1.242158651	5.622896671	5.058359623
O	-0.291986108	3.861814022	4.695230484
H	0.071083553	3.626470566	3.171175957
O	-0.601470828	1.362881303	3.083632708
C	-0.214152381	2.055143356	2.118866682
O	0.194525942	3.285429716	2.205724001

C	-0.171975151	1.444551587	0.746194899
H	-1.051808357	0.815757275	0.598096132
H	0.710035026	0.797073007	0.693517745
H	-0.105724379	2.213406086	-0.023611072
O	-0.179663926	2.432720900	7.079196453
C	0.646279871	1.960124612	7.994853973
O	1.442801714	1.044563532	7.883892536
C	0.466929317	2.737210989	9.298356056
H	-0.551055908	2.596177101	9.674057007
H	0.610083163	3.806754827	9.121452332
H	1.186012626	2.374646425	10.033752441

===============================

B7"-TS

===============================

C	-2.464296818	1.922169805	5.239749908
C	-2.027040482	0.849768579	6.221298218
C	-0.974243760	-0.269601017	5.988737583
O	-1.235669374	-1.384762764	6.446713924
N	0.186627701	0.069168411	5.378893852
Pd	0.088029914	1.966625452	4.781134129
C	1.359736800	-0.783456564	5.550162315
H	1.104007363	-1.473544717	6.362871647
C	1.680067420	-1.687152982	4.302287102
C	2.276551723	-0.906456649	3.117785215
H	2.394667864	-1.583333254	2.262134790
H	1.621421456	-0.086749047	2.818757296
H	3.257796288	-0.487729698	3.353760719
C	2.682881594	-2.767839670	4.752469540
H	2.930212259	-3.430493593	3.915549994
H	3.617108822	-2.324455023	5.114224911
H	2.267580748	-3.386567831	5.556974888
C	0.374612927	-2.365259171	3.844918013
H	-0.129672691	-2.862276316	4.678051472
H	-0.314892858	-1.621633768	3.434351921
H	0.589663386	-3.104784966	3.064371586
C	2.542259693	0.052618962	6.015185356
C	3.247148037	-0.298761755	7.173052311
N	2.866164684	1.134619117	5.288528442
C	4.317910194	0.489758492	7.584194660
H	2.940645933	-1.167585492	7.746281147
C	3.888098240	1.893947244	5.696052551
C	4.650308609	1.616601586	6.831100464
H	4.873745918	0.237910867	8.483627319
H	4.104988575	2.768647432	5.084808350
H	5.472974300	2.266238213	7.114749432
H	-1.741613507	1.295746207	7.179527283
H	-2.977161884	0.312558234	6.397650719
H	-2.688247204	1.574570060	4.235322475
C	-3.367454052	2.972805738	5.830590248
H	-3.971774578	2.514147282	6.622374058
H	-4.038873672	3.376548529	5.065769196
C	-2.504326582	4.105973721	6.419364452
H	-3.126090288	4.893134117	6.857148170
H	-1.848843217	3.708794117	7.200155735
C	-1.658635259	4.610190868	5.242263794
H	-2.208801508	5.367710114	4.666123390
H	-0.708162129	5.026043415	5.593974113
O	-1.413531780	3.492619991	4.402883530
H	-1.537254572	3.266738176	2.937046528
O	-0.370113909	1.178957582	2.819037914
C	-0.952591062	1.750960112	1.879737139
O	-1.575961113	2.897032499	1.974587321
C	-0.947538257	1.128736496	0.510703564
H	-1.015485287	0.043470766	0.604603291
H	0.009620442	1.364472628	0.032114994

H	-1.757572412	1.522998333	-0.104046620
O	0.649617672	2.943583250	6.438500404
C	0.809345722	2.447837114	7.650759697
O	0.418782771	1.353658557	8.059944153
C	1.588678718	3.410940886	8.543549538
H	1.349050045	4.455381870	8.316531181
H	2.658262491	3.258108377	8.349415779
H	1.381175876	3.184059620	9.591659546

================================

B7'''

================================

C	-2.305130005	1.685122728	5.531011581
C	-2.397460222	0.306762934	6.213287830
C	-1.064224005	-0.414383620	6.443294048
O	-0.813266039	-1.075704455	7.422032356
N	-0.122683525	-0.189601734	5.384020805
Pd	-0.257383674	1.959527612	5.224410057
C	1.225181460	-0.814977050	5.477030277
H	1.325612068	-1.130213380	6.518593311
C	1.310919285	-2.101544619	4.563738823
C	1.483155847	-1.737006783	3.076598406
H	1.576595664	-2.651802778	2.480566740
H	0.624564052	-1.181720734	2.694179773
H	2.386969328	-1.140291333	2.915999413
C	2.494964361	-2.992938757	4.997152805
H	2.415857553	-3.967445850	4.504281044
H	3.459112406	-2.571664810	4.705955029
H	2.499593973	-3.166446209	6.079268932
C	0.020097919	-2.932065010	4.749785900
H	-0.172048703	-3.134297132	5.808019161
H	-0.856919944	-2.435234547	4.325129986
H	0.123592444	-3.890323877	4.230766773
C	2.318377256	0.230046630	5.230437279
C	3.665945053	-0.054264337	5.476984501
N	1.957755804	1.453763604	4.811765194
C	4.628333569	0.919101417	5.233431816
H	3.952749014	-1.007672787	5.897736073
C	2.877243996	2.400087595	4.585832119
C	4.236409664	2.171838284	4.760862350
H	5.675467014	0.705530345	5.428131104
H	2.485251188	3.366048813	4.278121948
H	4.956506252	2.958939314	4.564281940
H	-2.856993198	0.386556059	7.201001167
H	-3.019413948	-0.363644272	5.605939388
H	-2.645864487	1.654201388	4.496464252
C	-2.871100187	2.846919298	6.311923504
H	-2.356767893	2.886875629	7.275052547
H	-3.924549341	2.607774734	6.530441284
C	-2.800634623	4.222959042	5.606472969
H	-3.721270800	4.406825066	5.036377907
H	-2.751611948	4.994677067	6.382535458
C	-1.611558318	4.387533188	4.634570122
H	-1.848690987	3.913288116	3.667523146
H	-1.478604317	5.461193085	4.423764706
O	-0.382687181	3.933488369	5.127588272
H	-0.601630688	-0.307120383	4.454103947
O	-0.515449166	1.978639245	3.172072411
C	-1.049116135	0.998183131	2.525492668
O	-1.418837190	-0.088059783	3.021453142
C	-1.185716629	1.225094676	1.031415224
H	-1.154391646	2.286401033	0.783600986
H	-2.113227367	0.768374264	0.679239094
H	-0.352879047	0.719161570	0.530651152
O	-0.226144165	2.161882162	7.254240036
C	0.722838223	1.713842154	8.047217369

O	1.717306137	1.081086397	7.720729351
C	0.413303643	2.043406963	9.503291130
H	-0.406450748	1.399533510	9.838739395
H	0.090788454	3.082218170	9.605714798
H	1.296249509	1.852200031	10.114515305

===============================

B7'''-TS

===============================

C	-2.529272318	1.890697956	5.488229275
C	-2.175136805	0.700693488	6.395110607
C	-1.100988030	-0.382062405	6.203466892
O	-1.163850069	-1.382490993	6.875680447
N	-0.035670452	-0.152338326	5.249808311
Pd	-0.209845811	2.004239321	4.821192265
C	1.250620723	-0.824046433	5.570075512
H	1.131594181	-1.269162774	6.559063435
C	1.588923931	-2.001160860	4.582507610
C	1.864962578	-1.502959490	3.151451349
H	2.100313187	-2.357559204	2.507146835
H	0.993600786	-1.000718474	2.725529432
H	2.709839582	-0.810263276	3.116431475
C	2.833531380	-2.745620489	5.108744144
H	3.040373802	-3.613859892	4.474210262
H	3.726343870	-2.113448143	5.102569103
H	2.678898811	-3.110014200	6.131293774
C	0.404525131	-2.990411997	4.562466621
H	0.137325749	-3.309138298	5.574113369
H	-0.483910769	-2.552177191	4.099792480
H	0.673930466	-3.876201153	3.976532698
C	2.363305569	0.211649939	5.641305923
C	3.382236958	0.099130251	6.593891144
N	2.318469524	1.230584979	4.771607876
C	4.389900684	1.058002949	6.625323772
H	3.373148680	-0.727795124	7.295516491
C	3.289647579	2.153236151	4.806923866
C	4.349955082	2.109930515	5.707963943
H	5.187714100	0.992290497	7.359869480
H	3.198996067	2.963447809	4.087406158
H	5.110703468	2.883934975	5.699291706
H	-1.950247884	1.075563908	7.398280144
H	-3.127768517	0.154958516	6.496180534
H	-2.842873573	1.641368389	4.480216503
C	-3.344925165	2.915348053	6.245649338
H	-3.906406403	2.415654421	7.044060707
H	-4.052278519	3.397152185	5.563674450
C	-2.428658724	3.991879225	6.858122826
H	-3.017090321	4.776314259	7.345198154
H	-1.764319777	3.541653156	7.602079868
C	-1.588318706	4.562315941	5.694786072
H	-2.110401154	5.414062500	5.233730316
H	-0.593856514	4.866223335	6.039411545
O	-1.510737896	3.596200228	4.669744968
H	-0.446191758	-0.409495056	4.318299770
O	-0.720360577	1.722998142	2.867533207
C	-1.193472028	0.641975105	2.344915390
O	-1.402430773	-0.427954257	2.951367855
C	-1.455913544	0.739269137	0.851930201
H	-1.788008213	1.741813898	0.576560080
H	-2.189354420	-0.012418329	0.555772483
H	-0.516848862	0.536120415	0.324829936
O	0.454982817	2.779387951	6.573934078
C	0.753454626	2.111027241	7.655769348
O	0.477960259	0.932902992	7.879843712
C	1.537313581	2.948987007	8.656357765
H	1.145296574	3.967425108	8.710990906

H 2.574471235 3.007742643 8.309370041

================================

DG of A7

================================

C	-2.003298044	1.733590007	5.658583641
C	-2.082743168	0.537379503	6.603325844
C	-1.132419348	-0.605732203	6.287368298
O	-1.217699528	-1.718702674	6.729433060
N	-0.067795075	-0.231654331	5.328826904
C	1.187108397	-1.046050310	5.409080505
H	1.057275772	-1.705595374	6.268937588
C	1.379370332	-1.948555589	4.136713028
C	1.741281748	-1.111371160	2.895477057
H	1.776550531	-1.760641098	2.013725758
H	1.020103097	-0.310499549	2.707242966
H	2.723052740	-0.642273486	3.007597208
C	2.506985426	-2.963103771	4.415449619
H	2.613593340	-3.637798786	3.559735298
H	3.470988512	-2.470277309	4.566015720
H	2.289839506	-3.575375557	5.298816204
C	0.080760323	-2.743698359	3.879588842
H	-0.259439796	-3.256942987	4.783556938
H	-0.738032997	-2.106566191	3.526029110
H	0.254844755	-3.489200592	3.097326756
C	2.369470835	-0.136092395	5.684048176
C	3.465821266	-0.594228566	6.419640541
N	2.329123497	1.108847618	5.181966782
C	4.556160450	0.250821829	6.603229046
H	3.447472334	-1.587321520	6.854370594
C	3.376320124	1.927985072	5.363536358
C	4.518718243	1.532675505	6.054747105
H	5.416659832	-0.083173610	7.176235676
H	3.252838373	2.921575069	4.947531700
H	1.514323950	2.471723318	9.636958122
H	5.346424580	2.223972082	6.175170898
H	-1.817813873	0.829068720	7.622876644
H	-3.099176645	0.125537053	6.631376266
C	-2.870291233	1.614654064	4.418304443
H	-3.903799295	1.769015670	4.767639160
H	-2.829074860	0.592023849	4.021880627
C	-2.549407005	2.626377583	3.313450098
H	-3.383454561	2.682637691	2.604788065
H	-2.430223942	3.615048409	3.764827967
C	-1.247769475	2.233288765	2.565388918
H	-1.507869244	1.670562267	1.654581547
H	-0.708724916	3.138456106	2.251086950
O	-0.411910772	1.372675180	3.297996759
H	-0.468513906	-0.279834867	4.379312992
H	-2.121820688	2.691059113	6.165179253

================================

MF of A7

================================

Pd	0.017698042	1.873292089	5.180344105
O	0.510912180	2.437598705	7.108030319
C	0.812105238	1.570464373	8.029578209
O	0.623475432	0.350105375	7.971038342
C	1.459309936	2.210163832	9.251514435
H	0.848811567	3.043146372	9.611198425
H	2.433451891	2.618214846	8.964273453
H	1.587067246	1.465916634	10.038647652
O	-0.308241785	3.859951496	4.928243160
C	0.688645065	4.570507050	4.455729961
O	1.769475460	4.131087303	4.079079151
C	0.366747051	6.059205055	4.437094212
H	0.474637181	6.454195976	5.452873230

H	-0.663416088	6.234983444	4.118486881
H	1.065936804	6.574484825	3.776837111

===============================

DG of A7-TS

===============================

C	-2.340305567	1.348458767	5.317164898
C	-2.290280104	-0.146181583	5.388811111
C	-0.973711014	-0.703738153	5.943057060
O	-0.895380318	-1.369969249	6.947628975
N	0.165532663	-0.337191522	5.167659760
C	1.434020758	-1.080704331	5.406471729
H	1.332446337	-1.526552200	6.398359299
C	1.600737929	-2.252139091	4.372354031
C	1.790992260	-1.723391771	2.937767267
H	1.886886597	-2.566583157	2.245053053
H	0.943200052	-1.118222952	2.595396280
H	2.685639620	-1.103106618	2.851083755
C	2.831774950	-3.091442347	4.770133495
H	2.922643423	-3.956716299	4.105384827
H	3.757805824	-2.515359640	4.697599411
H	2.743061543	-3.468090534	5.795874119
C	0.356747091	-3.162407875	4.425655365
H	0.149969980	-3.498614788	5.446154594
H	-0.539485276	-2.655048609	4.051615715
H	0.510473371	-4.043806076	3.794773102
C	2.604966402	-0.114842154	5.415998459
C	3.699116230	-0.306665480	6.261374474
N	2.514740705	0.923491240	4.576952457
C	4.741527557	0.617491663	6.218870163
H	3.728618622	-1.155382156	6.937580109
C	3.506185293	1.823529601	4.555791855
C	4.644735813	1.705740929	5.352827549
H	5.606891155	0.497793317	6.864958763
H	3.345690966	2.675855637	3.902729273
H	5.425705910	2.457991123	5.304360867
H	-3.063148260	-0.474668324	6.096073627
H	-2.500846148	-0.583820999	4.407246590
C	-3.351322651	2.065809727	4.494914055
H	-4.100543022	2.476241112	5.188184261
H	-3.853742123	1.383310795	3.799547911
C	-2.680699110	3.226228714	3.736930847
H	-3.409472942	3.771929741	3.128543615
H	-2.230509758	3.920340300	4.451608658
C	-1.579634428	2.565206528	2.901648521
H	-2.016229153	2.109035492	1.999670386
H	-0.818427801	3.294510126	2.597060442
O	-0.974321425	1.534590602	3.651282787
H	-0.056689292	-0.245235920	4.175175190
H	-2.010679722	1.846936822	6.231816292

===============================

MF of A7-TS

===============================

Pd	0.169411793	1.892289639	5.303278923
O	1.156883717	1.948812127	7.066642761
C	0.369044095	1.809748888	8.098751068
O	-0.857961595	1.669922829	8.054452896
C	1.125179887	1.838995218	9.418681145
H	1.450142622	2.864558220	9.622675896
H	2.021668673	1.216655612	9.354005814
H	0.477921963	1.495635629	10.226613045
O	-0.032202262	3.911193371	5.379256248
C	0.740564585	4.564840794	4.545978069
O	1.464103818	4.060463905	3.688109636
C	0.662408471	6.073802948	4.744875908

H	1.180289268	6.338318348	5.672589779
H	-0.376224279	6.399799347	4.844866753
H	1.142194748	6.580231190	3.905877590

================================

DG of B7

================================

C	-2.359063148	1.893539429	5.524950504
C	-2.644064188	0.430946469	5.859982014
C	-1.549705863	-0.590525925	5.596333504
O	-1.731233716	-1.770971060	5.463703156
N	-0.215327337	-0.008259121	5.710235119
C	1.024040699	-0.852984965	5.521387577
H	0.865802348	-1.739779830	6.145720005
C	1.408965945	-1.387033820	4.084675789
C	1.994619370	-0.292918056	3.168680191
H	2.145102262	-0.714377463	2.168548107
H	1.345797181	0.576916218	3.083458185
H	2.966329098	0.059335846	3.525334597
C	2.506012440	-2.456581116	4.311582088
H	2.805799961	-2.874432325	3.345292568
H	3.402956247	-2.041671991	4.780584812
H	2.141981602	-3.285182476	4.930972099
C	0.235625699	-2.085224152	3.371746778
H	-0.170155451	-2.900745153	3.973577738
H	-0.577653289	-1.398990989	3.138691664
H	0.603875339	-2.506201506	2.428906441
C	2.143018246	-0.000967022	6.102022171
C	3.118151188	-0.522531569	6.952069283
N	2.141193867	1.288661599	5.722744465
C	4.140880108	0.317767620	7.392975330
H	3.076455355	-1.563957334	7.254367352
C	3.116565228	2.095971584	6.150791645
C	4.146627426	1.647502780	6.979259968
H	4.916510105	-0.062250905	8.052008629
H	3.041829586	3.123425484	5.811354637
H	4.924361706	2.333846092	7.298436165
H	-2.894485474	0.319433033	6.922387600
H	-3.508051395	0.091764227	5.281999588
H	-2.641595125	2.130021811	4.503919601
C	-2.874444485	2.911640167	6.518133640
H	-3.967856884	2.765378475	6.540647984
H	-2.699059963	3.913437128	6.118251801
C	-2.328644276	2.821677685	7.951228619
H	-2.833987236	3.571220160	8.573091507
H	-2.552029371	1.842599988	8.394639015
C	-0.814411223	3.049517393	8.000218391
H	-0.581188202	4.064434052	7.644437313
H	-0.468626112	2.977747679	9.043572426
O	-0.093204275	2.085918665	7.278419971
H	-0.181670099	0.325896144	6.696766376

================================

MF of B7

================================

Pd	-0.274936020	2.082752228	5.276018620
O	-0.323448867	2.070064545	3.224555731
C	-1.102117777	1.260589242	2.564043283
O	-1.876623392	0.433943063	3.059050560
C	-0.945621908	1.400430441	1.057056189
H	0.044982649	1.032797694	0.769965589
H	-1.001942158	2.452237368	0.764785945
H	-1.714298129	0.818235397	0.547374785
O	-0.560334682	4.077784061	5.135666847
C	0.509410560	4.757473469	4.782209396
O	1.632804990	4.288756847	4.643684864

C	0.200759023	6.225615501	4.527922153
H	-0.581698000	6.590027332	5.197021008
H	-0.155042902	6.332395077	3.497440100
H	1.113030553	6.813016891	4.645432949

===============================

DG of B7-TS

===============================

C	-2.475529671	1.520079017	6.008864403
C	-2.476075649	0.133477539	6.578542233
C	-1.354684949	-0.789656878	6.076694012
O	-1.536235809	-1.975035071	5.900682449
N	-0.109879680	-0.146084487	5.879243374
C	1.102280855	-0.975092292	5.631474495
H	1.017017722	-1.864409447	6.266383171
C	1.331431866	-1.488888502	4.142292500
C	2.198288202	-0.516379714	3.313309193
H	2.309753656	-0.920742631	2.300767660
H	1.750541568	0.474676847	3.238747597
H	3.204027653	-0.408414096	3.731183052
C	2.086663485	-2.830027103	4.256618023
H	2.290446281	-3.229938030	3.257897377
H	3.051867008	-2.710593224	4.761380672
H	1.499099135	-3.575461864	4.804406643
C	0.009589764	-1.736466885	3.392026424
H	-0.574365616	-2.530572653	3.857589483
H	-0.612682343	-0.840123713	3.339382172
H	0.239666894	-2.038533926	2.364188910
C	2.274466038	-0.128753662	6.104320526
C	3.414584160	-0.686292887	6.685972691
N	2.161699295	1.188282013	5.873182297
C	4.482230186	0.155926362	6.998383522
H	3.462750196	-1.752886772	6.881617069
C	3.180041790	1.998271704	6.181454182
C	4.369267941	1.520898223	6.736071587
H	5.387220860	-0.249761626	7.442494392
H	3.011392355	3.049828529	5.964792252
H	5.179528713	2.206227541	6.963499069
H	-2.452268362	0.145919859	7.672932148
H	-3.408541441	-0.356584579	6.268995762
H	-2.397974968	1.534618855	4.915944576
C	-3.291981459	2.629264355	6.587787628
H	-4.336943150	2.285539865	6.614696503
H	-3.236840725	3.482510805	5.904815197
C	-2.794955254	3.032371521	7.976435661
H	-3.282406807	3.948150396	8.325181007
H	-3.005380869	2.242744207	8.707159996
C	-1.284105062	3.216565847	7.805387020
H	-1.067400336	4.150580883	7.276344299
H	-0.773887753	3.229882240	8.777896881
O	-0.792834163	2.106791496	7.079638004
H	0.045122407	0.446265906	6.700873852

===============================

MF of B7

===============================

Pd	-0.184486315	2.165839672	5.124601364
O	0.228232473	2.069036484	3.162687540
C	-0.770572007	1.632364631	2.443306446
O	-1.875119328	1.288371563	2.881694555
C	-0.424493521	1.511836767	0.968712747
H	0.210517913	0.629121244	0.834821641
H	0.143344060	2.381935358	0.631497741
H	-1.334996104	1.391229987	0.380370557
O	-0.706238508	4.107283592	4.870131016
C	0.338745356	4.907473087	4.885312080

O	1.491287470	4.577185154	5.147939682	H	-0.057227504	6.417289257	3.424003363
C	-0.022404766	6.339569092	4.515931129	H	0.743646383	7.018710136	4.893305302
H	-1.007120371	6.613196373	4.901521206				

Table S7. Vibrational Frequencies (in cm^{-1}) of the Optimized Geometries

==============================

PhI(Oac)2

==============================

32.36 46.84 55.56 57.06 81.06 84.71

100.39 105.07 126.26 145.05 181.19 191.95

215.11 230.76 242.12 263.81 264.75 420.50

470.91 491.54 498.52 611.92 613.78 619.33

653.27 661.81 673.76 694.78 762.44 861.85

929.27 935.92 958.48 984.30 1003.43 1014.85

1016.74 1018.08 1035.11 1065.43 1065.51 1078.92

1115.10 1199.78 1227.54 1284.23 1303.17 1340.15

1365.91 1394.64 1394.78 1480.96 1481.16 1484.83

1487.90 1488.03 1516.23 1623.38 1637.54 1753.75

1768.87 3064.03 3064.11 3135.24 3135.34 3173.41

3173.47 3187.88 3199.29 3210.57 3233.24 3233.43

==============================

Acetate

==============================

51.94 420.50 603.36 611.16 859.89 986.28

1020.69 1316.96 1374.64 1479.64 1493.17 1759.99

2995.08 3059.88 3080.92

==============================

Acetic

==============================

87.64 422.64 541.90 583.91 678.09 866.27

1003.24 1069.97 1218.46 1354.74 1421.72 1482.49

1488.56 1854.17 3066.12 3133.02 3185.40 3755.03

==============================

Iodobenzene

==============================

154.66 227.69 257.17 413.83 463.50 624.13

658.31 702.64 750.86 849.92 921.71 973.77

1001.94 1002.90 1036.86 1084.12 1107.96 1197.12

1220.11 1329.02 1353.22 1479.41 1515.64 1631.53

1634.95 3182.50 3191.39 3204.52 3212.97 3216.02

==============================

A1

==============================

31.66 38.39 40.92 46.55 52.08 55.71

64.15 66.62 72.42 73.15 86.31 95.31

101.03 112.69 118.27 129.04 132.13 138.43

148.25 156.16 175.78 184.49 201.40 205.09

211.83 233.95 247.19 254.20 261.60 278.58

287.47 300.22 302.57 314.69 320.94 332.32

335.61 358.31 363.47 416.89 421.64 435.45

444.28 449.46 478.18 500.27 517.60 534.76

548.40 598.16 602.77 612.95 617.05 620.40

669.55 676.21 681.38 692.57 716.59 746.00

775.48 779.14 804.59 836.68 859.67 873.61

901.14 912.33 931.43 937.00 943.01 948.49

951.85 972.50 984.53 985.37 992.23 1016.22

1017.59 1034.02 1037.52 1039.54 1053.97 1061.54

1064.11 1066.22 1074.02 1080.61 1083.71 1088.65

1094.76 1100.66 1135.03 1145.89 1165.04 1181.33

1198.98 1220.60 1242.86 1245.37 1261.31 1262.53

1268.37 1305.56 1315.03 1325.53 1328.09 1345.42

1354.57 1364.84 1376.67 1384.98 1403.37 1404.34

1407.30 1413.46 1422.97 1426.63 1428.76 1433.87

1459.12 1469.68 1477.36 1477.73 1480.37 1485.99

1488.39 1489.66 1497.50 1498.25 1501.45 1507.88

1510.02 1510.72 1519.04 1523.66 1525.51 1529.88

1543.14 1560.27 1625.38 1651.66 1664.85 1701.12

1812.22 2851.61 2976.96 3013.37 3027.89 3034.98

3036.84 3040.10 3044.86 3045.55 3047.65 3056.30

3060.09 3062.08 3072.34 3094.87 3099.37 3105.25

3108.45 3111.99 3113.38 3125.78 3131.16 3132.17

3133.75 3144.41 3166.26 3170.12 3201.16 3225.88

3229.72 3266.40 3698.29

==============================

A1-TS

==============================

-413.63 33.55 37.91 47.88 65.07 65.90

67.42 74.96 81.23 87.97 90.03 95.17

97.89 106.88 118.86 137.83 149.10 157.37

172.13 187.24 196.32 209.42 225.64 230.56

237.36 242.15 246.42 268.75 270.58 287.83

290.71 304.81 311.78 329.33 337.84 345.28

348.82 368.87 389.81 420.25 424.59 442.34

448.98 472.14 489.87 525.91 540.51 551.61

599.44 612.80 614.22 614.46 652.08 671.39

677.61 683.71 693.54 701.42 741.74 744.29

772.33 783.47 799.01 813.96 831.51 862.79

887.94 914.53 915.56 929.55 943.08 943.84

947.19 951.08 967.17 979.66 990.16 1004.82

1020.28 1032.25 1037.30 1040.56 1054.85 1056.09

1065.19 1066.40 1090.16 1097.71 1117.06 1119.56

1128.89 1145.70 1155.51 1157.45 1182.07 1199.09

1209.81 1219.57 1243.70 1245.75 1258.01 1266.15

1272.31 1303.20 1305.12 1321.65 1328.94 1345.67

1373.29 1373.72 1381.46 1396.66 1400.26 1407.37

1413.99 1418.63 1420.23 1423.13 1428.50 1448.43

1462.51 1475.50 1479.41 1483.49 1484.44 1489.88

1490.03 1490.94 1498.22 1500.20 1505.12 1508.03

1510.31 1511.02 1522.24 1525.74 1528.82 1530.05

1539.73 1547.44 1613.36 1622.72 1663.11 1674.44

1713.55 1761.25 2580.21 2707.35 2770.37 2984.66

3017.84 3024.43 3030.76 3035.93 3036.28 3041.11

3046.48 3049.33 3059.47 3066.96 3074.63 3080.41

3095.91 3103.30 3103.92 3105.33 3110.05 3121.14

3130.82 3133.94 3158.63 3166.97 3198.54 3221.07

3230.91 3236.45 3522.73

==============================

A2

==============================

24.11 30.22 38.34 43.62 45.26 54.28

55.46 66.83 68.96 73.39 81.80 95.04

97.31 106.32 113.96 131.03 138.27 159.40

167.32 169.86 182.89 194.94 196.93 213.55

220.29 227.34 235.13 245.50 253.24 274.78

279.38 301.03 302.81 311.76 316.86 332.02

344.12 358.55 366.43 390.14 420.30 423.65

441.76 470.23 484.92 501.71 517.67 521.90

563.60 577.61 608.12 609.04 667.91 673.39

678.44 685.01 693.84 695.07 741.29 759.89

767.94 778.22 810.26 828.86 864.00 889.56

897.40 904.21 930.11 933.40 939.66 947.15

950.14 962.06 969.74 977.97 982.45 1012.89

1015.29 1031.73 1032.76 1039.70 1049.89 1060.11

1062.52 1064.66 1070.38 1071.83 1090.45 1091.42

1101.34 1116.14 1144.96 1160.24 1197.86 1219.04

1225.09 1234.93 1242.07 1252.81 1265.69 1267.31

1284.64 1299.13 1304.60 1317.37 1324.05 1348.25

1353.63 1359.81 1363.60 1385.23 1389.31 1393.20

1409.93 1416.65 1417.95 1421.98 1424.42 1428.29

1456.43 1473.10 1478.86 1482.35 1487.12 1488.45

1491.63 1497.15 1499.19 1499.35 1502.07 1505.07

1511.14 1517.01 1521.19 1524.37 1526.82 1528.49

1536.19 1543.45 1624.80 1661.00 1680.81 1688.45

1748.63 2274.52 3002.34 3017.23 3023.43 3035.18

3041.03 3048.09 3048.33 3050.90 3057.60 3062.54

3065.63 3067.21 3070.40 3070.55 3103.99 3105.29

3107.45 3111.87 3119.06 3128.26 3128.38 3129.06

3142.57 3153.42 3166.66 3182.97 3198.71 3225.91

3231.04 3267.17 3555.97

==============================

A3

==============================

19.36 27.39 37.09 42.32 48.15 53.59

68.09 79.25 85.88 93.24 109.47 116.51

125.13 147.52 159.60 176.13 198.11 218.14

237.20 240.84 259.53 271.84 279.68 288.70

306.43 315.44 320.72 335.99 340.44 347.15

359.62 376.91 393.18 411.88 418.14 437.09

444.29 479.14 495.09 503.54 540.86 558.35

561.28 606.17 654.50 675.90 685.39 689.42

712.85 734.46 759.90 777.32 805.30 817.88

852.08 881.38 899.20 903.16 915.46 947.91

951.54 957.19 965.71 977.14 979.07 998.02

1015.04 1020.07 1021.60 1047.39 1049.88 1057.94

1061.84 1068.30 1076.55 1084.52 1093.33 1106.84

1112.27 1144.44 1178.12 1195.54 1209.16 1214.87

1238.49 1241.68 1246.58 1255.77 1286.49 1302.14

1319.74 1321.74 1338.54 1358.27 1363.03 1365.55

1384.53 1387.64 1389.57 1412.86 1416.56 1420.04

1421.43 1437.25 1451.15 1461.46 1480.04 1482.01

1490.52 1491.38 1498.43 1498.86 1506.24 1508.82

1513.14 1519.29 1523.04 1524.33 1525.64 1529.95

1540.29 1559.65 1623.48 1663.90 1693.72 2981.37

3021.01 3033.28 3036.92 3045.86 3047.19 3047.50

3061.62 3062.16 3067.35 3069.75 3076.99 3081.93

3092.85 3102.83 3105.66 3111.90 3114.33 3120.96

3125.90 3135.46 3146.89 3170.87 3199.90 3213.29

3228.82 3233.46 3780.18

==============================

A3-TS

==============================

-1275.39 28.32 30.08 39.69 48.04 55.69

64.28 67.57 82.72 94.77 100.94 111.96

122.16 139.53 165.86 185.03 194.87 207.92

225.53 232.73 243.63 245.28 259.23 266.45

285.92 288.99 293.19 308.71 310.81 324.70

331.69 350.77 372.30 402.69 409.44 421.02

440.46 463.16 484.93 507.92 532.20 554.65

567.13 591.39 619.04 636.67 669.10 674.20

697.13 732.89 741.22 781.92 782.16 806.80

863.38 878.05 896.64 915.80 917.70 922.78

947.37 950.84 953.00 975.42 995.76 997.89

1006.66 1017.63 1044.57 1047.28 1053.18 1056.63

1061.59 1070.49 1072.84 1084.49 1085.64 1103.25

1106.67 1129.58 1146.33 1173.35 1190.39 1195.37

1211.70 1237.59 1245.17 1246.07 1254.27 1259.52

1297.93 1300.19 1321.29 1326.67 1331.19 1348.10

1353.03 1360.68 1373.87 1393.14 1408.84 1412.24

1418.52 1420.63 1450.77 1459.75 1460.96 1477.82

1480.03 1485.59 1488.40 1491.68 1494.60 1500.82

1505.81 1510.85 1518.80 1522.17 1525.77 1536.07

1538.68 1548.19 1621.56 1660.87 1674.04 1733.03

2962.96 2993.19 2996.68 3032.33 3035.67 3038.41

3040.77 3044.63 3048.44 3058.52 3065.46 3074.81

3083.55 3101.33 3103.46 3105.55 3113.64 3123.64

3127.47 3136.27 3147.46 3173.40 3196.72 3214.71

3227.07 3230.72 3831.79

==============================

A3-TS'

==============================

-1393.72 24.65 30.27 41.98 54.25 58.48

66.03 70.30 83.07 103.51 103.65 109.93

131.22 141.44 166.38 175.09 197.48 213.06

231.92 239.36 246.86 252.94 264.34 271.42

287.43 289.43 301.96 309.54 317.80 325.64

333.61 359.08 371.19 405.70 411.92 415.82

419.16 440.95 460.36 486.23 526.45 548.45

557.48 576.65 619.16 629.54 666.62 676.23

700.19 729.50 737.42 781.25 792.27 806.52

866.12 897.55 905.55 917.83 922.98 940.27

947.65 951.58 959.58 976.34 993.48 1004.89

1016.35 1017.30 1044.43 1046.32 1052.31 1057.10

1061.75 1068.49 1070.89 1084.00 1085.60 1105.35

1122.97 1138.49 1146.03 1171.78 1195.29 1213.06

1219.44 1237.36 1247.14 1254.74 1258.07 1264.24

1295.33 1299.47 1301.86 1325.19 1333.15 1344.58

1347.77 1360.66 1377.41 1393.11 1393.65 1415.39

1417.08 1419.44 1449.58 1453.99 1461.41 1475.85

1479.38 1479.84 1489.64 1490.06 1491.65 1501.29

1507.63 1514.46 1518.85 1521.39 1526.07 1528.98

1537.79 1539.65 1620.96 1659.91 1666.32 1726.54

2962.37 2990.65 3030.97 3032.04 3036.01 3036.93

3039.44 3040.53 3045.09 3048.39 3063.40 3068.60

3076.64 3097.44 3101.16 3103.26 3112.13 3120.62

3124.32 3137.22 3143.89 3175.06 3196.04 3211.41

3224.63 3230.83 3831.41

==============================

A4

==============================

38.01 42.56 53.95 58.05 71.94 78.94

94.55 105.73 108.95 113.79 116.05 128.73

139.98 153.40 163.65 179.71 186.72 201.68

211.95 238.52 249.89 251.52 268.25 283.88

291.15 294.29 308.60 313.57 319.25 330.25

354.18 356.44 398.46 408.76 411.37 428.45

442.78 464.38 474.39 512.03 546.58 558.76

573.49 596.87 630.86 639.33 656.18 676.40

738.28 742.04 755.29 773.73 788.67 810.74

860.49 870.04 888.90 896.68 898.51 917.51

948.20 951.05 970.19 976.14 978.17 995.87

1014.47 1015.13 1028.24 1028.71 1040.49 1051.16

1058.33 1063.10 1072.97 1078.67 1085.88 1101.34

1120.90 1126.79 1146.85 1194.65 1202.09 1212.23

1227.79	1236.76	1248.02	1249.79	1256.12	1270.25
1285.64	1293.19	1312.15	1322.76	1327.15	1330.80
1353.44	1361.56	1367.70	1374.96	1389.09	1405.21
1412.49	1419.37	1429.06	1447.85	1457.37	1473.54
1476.15	1485.83	1489.51	1497.06	1498.10	1502.57
1506.17	1514.18	1515.78	1519.55	1525.90	1537.82
1540.12	1619.82	1656.94	1732.48	1755.86	2981.70
2989.74	3016.06	3024.77	3030.57	3031.17	3035.28
3037.51	3044.51	3045.66	3054.05	3069.84	3074.10
3096.19	3098.14	3101.52	3109.78	3120.33	3127.03
3144.30	3149.75	3182.05	3189.85	3196.43	3218.04
3223.57	3498.24	3816.97			

================================

A5

================================

35.18	51.21	54.69	78.94	92.74	96.79
106.91	129.74	147.94	165.44	186.78	223.37
241.62	255.29	270.81	293.02	294.96	299.87
313.53	317.58	320.96	335.29	356.76	364.81
400.90	408.70	413.81	434.72	451.46	463.32
493.67	521.80	547.60	558.42	562.98	624.02
648.43	676.58	735.96	744.28	772.55	809.28
818.94	867.16	880.32	893.32	911.52	914.49
924.44	947.79	950.71	964.26	966.70	976.93
1009.42	1011.82	1024.58	1035.47	1050.80	1058.09
1063.05	1072.54	1082.96	1102.04	1116.78	1136.37
1147.86	1183.16	1192.82	1211.30	1231.20	1236.44
1247.35	1255.25	1255.91	1283.89	1292.92	1302.01
1317.35	1324.80	1348.15	1353.19	1365.44	1390.16
1397.60	1404.30	1415.50	1418.58	1448.42	1451.24
1471.45	1476.20	1483.26	1489.60	1491.54	1501.80
1506.62	1514.33	1520.17	1520.80	1525.50	1538.77
1619.87	1655.90	1729.14	2977.08	3005.34	3021.19
3022.48	3029.62	3030.60	3033.40	3035.91	3039.65
3044.55	3071.47	3072.80	3081.66	3098.51	3101.48
3109.71	3118.28	3121.93	3146.97	3170.33	3192.97
3214.82	3221.62	3820.74			

================================

A6

================================

29.72	38.01	43.28	50.31	61.43	64.92
70.55	75.07	83.92	89.13	92.35	106.98
118.07	126.58	129.70	139.54	156.60	171.25
184.13	189.62	210.44	227.28	233.29	255.05
262.17	271.40	275.37	285.20	294.85	302.83
312.03	323.34	332.48	339.67	351.76	360.59
372.95	395.82	408.04	418.33	426.21	431.13
461.48	496.20	522.01	530.98	541.94	548.59
555.58	587.04	606.65	619.00	627.33	654.57
674.50	679.24	689.71	729.13	745.23	772.63
804.31	808.00	857.24	884.25	893.47	911.63
918.12	932.95	934.75	940.91	949.54	953.87
972.64	978.34	979.60	990.15	1013.15	1022.66
1028.68	1033.04	1039.11	1054.08	1059.74	1062.29
1065.11	1068.75	1070.44	1082.72	1096.21	1109.44
1134.14	1141.57	1194.73	1195.00	1202.58	1224.50
1239.94	1244.51	1253.93	1255.64	1263.36	1286.98
1301.44	1304.95	1318.98	1324.74	1328.97	1341.85
1352.64	1383.27	1384.43	1386.12	1389.04	1413.10
1416.00	1421.18	1422.65	1451.12	1453.84	1469.90
1474.51	1478.79	1479.92	1481.72	1486.60	1488.32
1493.38	1496.92	1505.72	1511.13	1514.68	1520.34

1523.60 1529.28 1545.44 1558.13 1615.08 1625.27

1660.33 1744.77 1768.80 2479.16 3000.19 3036.54

3038.87 3039.72 3046.67 3048.04 3052.70 3060.70

3061.46 3061.52 3069.65 3086.14 3099.73 3101.29

3104.76 3113.94 3120.15 3122.87 3131.02 3132.95

3134.61 3146.59 3158.21 3169.74 3169.92 3191.33

3205.96 3220.91 3231.80

==============================

A6'

==============================

37.34 44.76 51.22 57.00 63.26 70.11

74.17 85.02 97.00 102.45 113.85 115.12

127.93 137.72 143.18 154.87 168.53 174.89

182.67 189.05 206.43 213.29 237.66 254.60

264.11 271.12 275.77 284.10 297.77 305.52

314.38 318.37 321.41 330.29 338.95 354.32

368.42 370.53 406.66 419.77 433.98 441.83

460.51 483.25 496.20 522.58 529.76 534.34

558.44 562.00 604.87 615.08 633.77 652.77

674.38 689.85 690.36 739.43 745.83 774.42

808.62 821.66 866.39 868.54 892.00 901.75

914.09 926.92 931.63 934.75 951.87 952.59

976.40 981.49 984.13 993.90 998.55 1012.12

1019.69 1028.70 1036.86 1053.41 1056.33 1059.55

1065.38 1069.52 1074.74 1079.79 1085.98 1098.49

1122.28 1139.53 1143.03 1192.46 1195.47 1210.86

1237.43 1249.44 1251.89 1255.36 1259.52 1291.51

1297.68 1307.19 1313.70 1328.11 1345.82 1350.06

1358.72 1366.14 1376.64 1388.84 1396.76 1408.84

1414.55 1419.02 1423.00 1426.56 1452.63 1467.33

1474.81 1477.72 1479.09 1481.76 1486.52 1488.63

1490.14 1492.56 1507.35 1508.08 1514.02 1519.55

1524.21 1524.97 1530.36 1543.11 1628.30 1651.60

1657.43 1743.85 1761.55 2991.09 3033.79 3034.85

3040.38 3042.24 3044.27 3046.18 3049.07 3059.46

3062.31 3069.37 3080.87 3092.00 3095.68 3101.10

3105.29 3120.20 3122.25 3130.82 3131.13 3133.67

3135.29 3145.70 3148.03 3165.84 3170.65 3191.00

3215.39 3220.52 3236.71

==============================

B4

==============================

26.48 35.06 49.57 54.72 59.75 75.22

90.45 98.26 106.43 118.40 127.85 128.82

139.59 156.23 171.37 178.66 203.44 218.62

226.47 241.47 251.08 256.37 267.51 275.40

283.54 295.83 310.18 315.77 322.55 339.77

353.13 364.70 403.72 409.39 414.23 431.16

454.80 466.01 474.49 490.42 530.31 556.29

572.89 605.63 632.45 642.80 663.43 678.43

736.60 742.08 775.95 781.34 804.90 810.24

869.02 871.78 895.96 905.81 918.40 921.73

947.64 951.74 976.56 980.55 992.00 1008.20

1013.37 1028.35 1031.71 1035.16 1050.01 1055.62

1057.98 1062.58 1077.86 1080.40 1088.38 1093.64

1118.02 1142.02 1144.52 1192.50 1203.44 1211.39

1217.23 1236.42 1247.51 1255.21 1259.60 1282.38

1290.32 1297.05 1312.62 1321.12 1324.32 1328.51

1351.13 1357.67 1366.29 1396.50 1404.03 1411.43

1416.53 1419.61 1441.56 1449.54 1462.32 1474.07

1477.16 1488.32 1489.77 1500.60 1503.56 1506.95

1507.85 1514.71 1519.85 1525.41 1529.47 1538.83

1544.20 1621.44 1656.11 1731.54 1740.31 2969.28

2973.95 3012.79 3018.00 3022.80 3030.71 3032.13

3035.62 3036.77 3039.61 3046.59 3058.33 3066.46

3100.03 3103.70 3110.70 3118.13 3122.53 3130.74

3133.24 3145.11 3180.26 3185.92 3194.04 3215.80

3222.57 3342.19 3820.31

===============================

B5

===============================

21.06 51.22 55.21 58.55 73.70 95.65

101.26 119.32 136.50 147.56 193.19 224.72

243.14 244.06 256.31 259.58 270.30 290.43

306.61 307.73 311.32 323.66 349.24 389.28

390.15 404.73 408.07 426.44 433.56 473.55

486.47 518.63 550.87 557.24 574.26 633.86

650.26 677.95 732.58 742.13 775.41 810.34

811.60 856.19 867.67 898.25 903.62 917.98

921.22 948.83 952.15 969.49 974.93 977.66

1003.52 1010.85 1012.83 1028.27 1036.83 1056.72

1062.62 1078.74 1081.75 1099.98 1106.88 1124.68

1148.52 1173.03 1192.93 1212.96 1221.73 1236.49

1249.34 1256.39 1261.71 1283.75 1297.77 1305.48

1320.28 1325.31 1353.25 1361.44 1362.68 1371.82

1396.33 1402.38 1415.50 1418.86 1431.67 1448.61

1476.06 1478.41 1484.06 1489.69 1493.78 1501.35

1507.04 1513.88 1518.64 1519.05 1525.71 1539.14

1620.92 1655.48 1728.00 2994.10 3018.26 3025.18

3029.09 3031.57 3032.07 3034.78 3038.86 3044.98

3045.11 3061.95 3074.69 3089.49 3097.40 3100.18

3110.25 3118.22 3121.78 3142.57 3183.11 3192.25

3214.70 3222.70 3853.60

===============================

B6

===============================

41.09 43.74 47.29 52.01 55.50 66.38

69.30 83.04 86.40 104.98 108.02 114.41

124.97 129.61 152.00 160.38 165.87 176.67

186.97 203.26 214.51 228.80 230.80 252.55

263.57 280.08 282.28 291.03 296.13 300.14

305.81 319.55 321.01 324.45 330.19 358.95

373.02 396.43 406.38 417.49 440.11 453.84

463.80 488.45 518.79 534.40 541.57 546.88

560.88 589.03 609.42 611.04 642.22 652.91

674.14 689.17 700.93 738.82 754.28 775.70

801.98 809.31 863.72 874.04 891.11 908.11

914.27 916.34 930.44 939.84 951.07 951.21

976.97 978.80 981.37 992.36 1011.89 1015.87

1017.14 1029.74 1041.45 1051.73 1060.53 1063.21

1065.79 1068.95 1072.44 1081.74 1109.18 1114.39

1126.23 1140.14 1176.87 1191.80 1207.80 1215.95

1235.63 1243.61 1248.03 1255.49 1257.06 1284.87

1300.56 1306.76 1310.40 1326.41 1343.57 1355.73

1367.71 1373.44 1380.59 1388.97 1396.67 1409.47

1419.40 1421.67 1424.02 1448.27 1454.15 1470.75

1476.18 1479.04 1481.48 1484.48 1486.95 1488.66

1489.84 1503.64 1506.11 1507.75 1517.11 1519.57

1524.34 1530.58 1542.58 1590.29 1617.55 1628.98

1654.94 1737.21 1757.51 2508.21 2999.48 3028.02

3033.29 3040.39 3044.00 3049.93 3053.67 3056.99

3059.90 3060.25 3078.51 3082.32 3101.15 3102.37

3107.42 3112.25 3120.37 3122.78 3133.36 3134.05

3146.64 3152.19 3154.77 3166.95 3170.43 3190.85

3214.41 3219.94 3248.61

==============================

B6'

==============================

28.21 44.64 52.32 53.55 66.44 74.39

83.37 85.93 89.35 97.97 101.43 110.25

125.45 139.10 149.93 152.66 163.05 185.65

188.31 202.26 212.82 223.03 239.88 255.59

266.88 271.21 277.28 286.52 295.35 308.49

310.61 316.32 320.14 337.44 346.36 360.25

393.83 405.98 410.91 424.22 427.63 433.77

473.97 495.30 513.67 537.83 550.18 551.11

552.35 573.78 604.15 615.14 631.99 655.44

674.49 677.27 697.15 721.15 742.51 772.95

804.05 813.84 857.36 864.70 897.49 911.11

914.69 925.04 933.75 936.01 951.84 954.79

973.21 980.83 988.28 991.00 1013.89 1024.66

1028.15 1035.22 1045.48 1056.16 1059.21 1064.88

1068.20 1070.74 1078.59 1088.23 1111.27 1117.45

1132.29 1140.96 1163.51 1195.62 1203.60 1211.00

1238.44 1245.25 1254.57 1256.59 1273.95 1286.67

1294.19 1304.01 1317.29 1327.37 1340.16 1346.57

1372.14 1378.68 1382.90 1386.83 1395.31 1411.89

1416.75 1418.50 1420.79 1424.80 1452.58 1465.94

1477.31 1478.02 1481.78 1488.46 1489.22 1491.74

1492.38 1496.51 1508.29 1515.08 1516.99 1519.92

1523.83 1530.63 1533.34 1547.41 1623.21 1641.37

1660.68 1744.66 1768.43 2648.68 3007.64 3009.84

3016.93 3023.42 3039.57 3041.38 3046.72 3049.88

3055.12 3057.47 3062.29 3065.03 3084.39 3089.21

3097.85 3102.40 3110.22 3121.40 3128.23 3130.37

3136.03 3138.42 3149.34 3156.70 3168.03 3190.10

3195.59 3218.77 3234.83

==============================

A6-TS

==============================

-1315.52 31.83 37.06 46.70 53.92 63.23

77.83 84.38 89.07 90.15 100.63 104.62

110.03 116.39 118.77 125.55 140.28 166.00

171.86 179.21 200.23 211.78 219.26 230.62

243.84 258.23 267.03 270.69 285.69 294.07

307.73 314.99 320.30 325.87 331.25 345.06

367.25 388.59 407.83 421.51 432.51 442.41

454.78 490.30 508.40 520.22 536.12 550.84

555.61 568.11 607.50 610.02 622.45 639.47

647.98 678.28 682.31 695.05 721.85 753.55

779.78 812.98 818.80 852.09 886.59 908.08

919.89 924.23 934.92 937.45 939.68 950.29

951.47 961.66 982.84 989.00 1007.64 1011.60

1024.42 1025.01 1028.66 1029.03 1043.35 1061.19

1062.25 1064.13 1067.46 1073.30 1082.55 1091.36

1111.46 1137.17 1138.96 1192.83 1198.59 1206.60

1218.58 1239.09 1244.64 1256.80 1258.01 1261.68

1294.75 1307.98 1314.60 1318.46 1325.94 1328.78

1332.29 1349.23 1383.77 1389.70 1391.87 1396.63

1403.32 1418.88 1421.51 1426.41 1456.00 1470.63

1475.62 1480.17 1481.44 1483.10 1488.92 1489.38

1495.81 1499.55 1502.92 1506.24 1509.38 1515.13

1516.29 1529.10 1532.49 1543.21 1626.92 1653.26

1729.86 1739.21 1750.05 1817.35 3008.60 3014.40

3038.44 3042.58 3045.98 3048.05 3054.32 3055.24

3058.58 3060.57 3082.26 3102.50 3107.33 3107.52

3110.95 3125.54 3126.37 3127.55 3128.48 3133.48

3136.32 3151.30 3159.88 3161.35 3166.58 3190.06

3211.52 3223.45 3228.62

===============================

A7

===============================

31.33 38.58 42.95 50.91 60.27 69.24

77.68 81.75 87.47 99.85 106.89 111.30

113.03 121.11 127.75 142.48 160.91 163.21

181.07 191.61 205.06 208.70 232.14 242.33

248.36 253.76 272.48 278.74 281.10 290.81

304.01 311.52 316.56 330.82 347.90 365.86

374.70 407.28 415.47 421.27 433.79 459.13

487.51 508.68 522.76 523.94 547.64 560.00

571.35 602.99 614.65 620.79 633.15 645.99

674.74 681.99 690.43 711.77 749.27 779.70

804.95 815.02 820.05 875.75 887.61 903.70

917.12 927.08 935.01 939.15 942.12 950.99

954.89 965.74 986.20 1001.61 1007.18 1023.38

1025.20 1031.26 1032.57 1033.12 1043.98 1059.71

1064.46 1069.91 1071.76 1076.53 1082.12 1088.96

1108.59 1135.88 1137.34 1164.65 1192.26 1207.87

1221.49 1233.31 1244.56 1259.50 1261.90 1266.07

1282.91 1309.14 1319.93 1324.76 1330.89 1332.39

1345.41 1356.21 1377.41 1387.04 1392.11 1399.17

1401.55 1415.73 1422.09 1433.13 1453.63 1460.07

1464.98 1476.91 1479.73 1481.27 1481.39 1489.29

1490.00 1497.10 1498.95 1504.49 1509.08 1512.84

1517.86 1529.76 1531.85 1541.67 1628.42 1652.17

1701.74 1747.93 1865.94 2982.66 2998.55 3027.21

3037.38 3040.26 3045.69 3053.67 3055.11 3057.98

3058.72 3060.45 3102.61 3107.59 3116.31 3117.38

3119.53 3126.88 3129.39 3130.13 3131.19 3131.25

3133.45 3151.99 3165.23 3165.61 3189.11 3209.89

3218.05 3231.32 3307.60

===============================

A7-TS

===============================

-298.11 21.01 32.94 39.89 44.85 52.33

55.87 58.51 65.21 71.12 72.54 84.25

92.91 101.64 110.28 123.56 131.54 132.20

156.33 160.04 174.21 185.94 207.27 211.47

238.69 240.04 258.09 270.21 281.69 287.99

291.81 301.25 303.34 316.84 327.90 344.27

363.28 378.71 408.93 419.56 425.62 438.30

460.29 464.16 503.01 517.08 526.03 557.03

565.09 582.07 608.83 612.94 627.92 643.34

672.89 683.66 696.98 737.32 750.21 770.60

812.65 847.46 868.78 877.30 901.55 905.03

910.57 930.39 933.94 937.70 940.48 945.94

948.96 953.35 979.78 990.06 993.42 1007.85

1009.99 1014.25 1022.60 1025.25 1027.98 1045.91

1060.18 1062.88 1063.19 1074.19 1078.02 1088.70

1110.34 1133.78 1148.81 1163.04 1188.52 1196.33

1212.81 1225.09 1244.77 1255.02 1257.85 1261.92

1276.11 1300.04 1315.16 1328.61 1331.46 1341.71

1348.06 1350.27 1370.50 1385.18 1393.99 1395.08

1398.94 1423.02 1428.36 1432.61 1445.15 1459.66

1475.15 1480.92 1481.47 1482.36 1489.45 1489.78

1492.85 1494.08 1499.29 1508.25 1509.41 1519.43

1523.66 1525.76 1527.91 1538.43 1633.04 1647.42

1704.79 1725.15 1839.70 2995.44 3019.38 3038.37

3038.85 3043.43 3045.23 3056.96 3057.67 3067.35

3074.18 3080.01 3098.72 3099.24 3104.93 3108.81

3113.28 3115.77 3127.18 3128.08 3128.13 3131.54

3134.66 3142.67 3161.55 3166.04 3183.57 3199.80

3206.93 3219.28 3474.06

==============================

A8

==============================

23.78 32.71 34.45 44.51 49.61 55.67

63.56 74.78 76.23 78.36 87.99 97.09

100.73 115.02 124.27 137.96 152.23 159.96

167.58 180.95 187.40 192.81 193.62 216.22

224.57 233.15 239.55 254.26 268.16 288.70

296.12 301.01 310.27 320.33 332.15 337.14

347.12 380.27 409.88 423.12 457.36 462.34

484.02 516.46 527.24 534.42 549.24 569.87

574.52 607.34 611.19 646.56 651.57 664.97

680.78 687.88 703.67 752.81 776.66 780.69

787.94 818.76 846.80 877.29 899.58 900.43

927.47 930.45 934.02 935.67 944.59 953.29

958.04 967.72 972.09 978.04 993.90 1015.10

1023.09 1027.00 1034.07 1044.47 1047.68 1055.65

1060.09 1061.40 1063.38 1075.12 1094.06 1109.98

1135.86 1139.41 1181.85 1198.81 1211.52 1217.60

1221.61 1231.66 1248.81 1270.82 1279.27 1283.11

1304.89 1310.89 1321.63 1325.12 1338.20 1346.84

1355.80 1366.34 1386.00 1390.95 1400.01 1402.33

1407.59 1417.27 1422.26 1426.04 1459.55 1478.52

1481.25 1481.81 1486.13 1488.87 1489.00 1490.79

1499.92 1502.43 1503.34 1503.64 1513.48 1520.56

1523.63 1527.34 1529.30 1535.18 1628.26 1657.10

1708.96 1736.17 1791.17 3033.29 3041.97 3044.32

3047.20 3050.29 3053.12 3056.24 3057.53 3060.17

3084.44 3095.31 3107.50 3114.21 3116.25 3123.23

3124.84 3127.00 3127.05 3128.27 3130.68 3142.43

3147.12 3161.19 3164.36 3170.16 3197.13 3223.64

3254.12 3278.33 3622.77

==============================

A6-TS'

==============================

-210.66 8.10 37.38 52.26 56.13 56.29

60.36 69.03 78.85 82.09 85.36 94.51

106.76 117.22 129.05 129.26 141.46 153.33

170.75 184.12 204.59 212.09 233.59 236.73

257.74 261.76 272.39 278.02 297.99 301.72

313.89 318.71 322.23 342.67 357.89 374.20

379.83 406.83 421.30 429.73 430.53 436.84

464.07 487.57 503.36 521.84 550.53 553.16

556.73 584.13 601.73 607.92 628.27 637.04

655.79 675.33 684.33 729.39 741.67 747.29

773.44 804.89 810.05 856.64 888.72 899.34

912.12 918.55 935.32 946.17 949.93 953.22

953.98 963.60 971.39 980.64 995.54 1014.72

1025.36 1025.94 1033.49 1047.07 1053.59 1060.60

1064.92 1070.07 1070.49 1074.22 1080.57 1103.99

1108.23 1138.02 1141.53 1195.58 1202.55 1205.41

1238.94 1240.92 1255.77 1256.02 1258.38 1297.53

1301.19 1302.99 1319.10 1326.77 1326.86 1341.51

1349.38 1377.68 1387.72 1390.14 1391.54 1405.37

1416.12 1419.66 1422.03 1423.21 1451.92 1472.73

1474.16 1474.88 1478.68 1480.60 1482.39 1488.94

1491.01 1496.24 1501.34 1507.23 1511.56 1514.51

1520.10 1523.20 1530.01 1545.39 1622.19 1656.51
1660.39 1735.47 1749.16 1764.78 2998.47 3013.74
3032.73 3036.33 3039.98 3045.93 3052.01 3056.64
3057.67 3059.27 3064.56 3074.80 3089.71 3095.83
3102.23 3112.71 3118.04 3118.69 3127.97 3130.18
3134.72 3143.67 3155.61 3165.77 3174.08 3185.76
3191.63 3213.94 3225.53

==============================

A7'

==============================

24.52 35.99 49.50 51.28 56.66 60.73
67.33 74.07 80.72 85.96 93.52 104.50
113.09 126.32 128.03 135.31 140.83 154.22
170.46 184.01 212.15 226.04 232.57 236.27
257.93 260.61 275.00 279.63 300.17 311.77
315.01 322.86 327.14 342.78 366.65 378.87
383.78 405.84 411.86 424.45 431.04 436.55
463.83 484.89 499.01 522.80 540.06 549.83
554.89 583.58 606.20 607.61 632.39 653.83
659.35 675.82 681.87 726.32 745.94 770.39
805.15 808.57 855.96 886.37 897.11 909.47
916.41 934.56 936.30 947.69 952.32 953.99
962.46 968.53 979.76 992.03 1009.46 1012.26
1026.09 1028.50 1032.91 1052.06 1053.39 1059.44
1065.27 1069.73 1070.77 1079.00 1085.77 1103.64
1133.94 1140.53 1151.38 1195.15 1202.57 1203.76
1239.24 1240.22 1253.44 1255.23 1257.55 1297.69
1299.66 1305.69 1318.30 1325.21 1327.41 1341.14
1348.01 1374.90 1385.20 1387.02 1388.50 1395.82
1415.63 1421.63 1422.47 1424.49 1451.95 1468.93
1474.30 1475.39 1478.19 1480.44 1482.84 1489.36
1491.48 1495.72 1496.20 1505.81 1512.99 1515.60
1519.50 1522.64 1529.81 1545.81 1624.14 1660.59
1685.04 1740.09 1748.14 1763.99 3000.97 3001.76
3034.78 3035.79 3037.82 3046.71 3048.18 3054.84
3058.03 3058.98 3062.02 3069.50 3086.65 3096.65
3102.99 3119.13 3120.32 3122.15 3130.92 3132.27
3136.27 3143.40 3155.05 3167.99 3179.46 3187.28
3194.23 3218.04 3233.80

==============================

A7'-TS

==============================

-141.63 6.24 30.30 38.89 54.81 64.60
70.89 77.13 85.54 93.20 101.32 107.30
114.12 115.68 117.24 123.53 131.46 147.76
156.78 164.27 181.23 197.05 206.10 219.89
235.34 241.96 256.64 267.69 272.58 285.18
296.74 313.37 316.15 320.70 330.87 337.85
365.44 396.96 407.60 424.07 429.64 432.48
465.18 472.95 492.38 508.01 515.77 541.65
558.52 568.84 593.17 604.43 632.47 653.14
656.45 678.22 690.80 719.57 748.82 771.50
802.18 816.18 829.62 851.67 891.95 896.96
915.40 916.44 923.78 927.39 940.71 945.94
954.53 969.72 972.65 977.17 998.16 1009.29
1015.31 1030.95 1036.62 1039.49 1046.43 1054.70
1060.02 1064.02 1070.58 1075.55 1087.53 1120.00
1124.15 1132.33 1137.23 1180.62 1185.56 1189.96
1206.67 1240.37 1241.39 1249.09 1257.17 1265.84
1281.83 1295.47 1298.28 1312.66 1326.85 1338.70
1364.67 1367.55 1380.49 1387.67 1398.57 1401.25
1416.12 1420.49 1435.83 1438.07 1449.57 1456.07

1473.88	1474.75	1479.03	1482.52	1483.79	1486.34
1493.80	1493.96	1494.90	1502.95	1512.25	1515.92
1520.43	1525.13	1541.61	1562.88	1626.46	1646.39
1709.83	1741.62	1782.24	2448.16	2976.33	2978.77
3021.78	3033.94	3044.22	3048.45	3050.67	3056.63
3061.18	3064.67	3071.91	3087.20	3103.37	3106.16
3108.19	3114.15	3123.22	3127.47	3131.37	3148.26
3150.89	3153.51	3169.33	3173.41	3186.09	3186.61
3206.65	3207.65	3213.56			

================================

B6-TS

================================

-1256.56	36.74	40.68	50.57	53.67	60.64
72.03	77.94	82.25	88.79	93.33	111.01
114.27	122.22	127.97	138.87	151.07	159.71
171.01	182.07	194.01	210.89	230.00	237.38
250.94	263.70	280.07	282.95	289.81	299.64
315.19	325.16	330.52	333.97	343.57	378.81
392.55	393.64	415.14	427.18	431.45	438.23
445.50	478.96	518.24	520.19	524.79	538.89
560.93	564.67	584.29	602.77	610.02	643.07
655.43	671.95	682.64	687.18	738.82	755.48
779.64	816.26	818.34	854.57	874.47	895.19
898.71	919.60	925.25	937.17	939.13	952.57
955.40	968.32	984.17	985.92	1006.73	1015.35
1021.97	1022.63	1032.36	1033.60	1042.45	1060.57
1063.43	1067.95	1070.14	1077.26	1083.02	1092.47
1125.44	1130.18	1135.56	1190.19	1202.51	1219.18
1232.94	1239.58	1246.38	1251.52	1261.79	1266.81
1301.26	1306.72	1318.86	1322.69	1330.72	1333.15
1351.61	1356.94	1374.30	1382.13	1391.26	1391.76

1404.95	1416.23	1421.34	1429.31	1442.52	1459.28
1477.82	1479.27	1480.89	1481.40	1487.34	1488.13
1497.03	1503.33	1503.99	1507.48	1512.93	1521.36
1530.52	1533.63	1538.15	1553.81	1628.96	1652.18
1688.92	1733.42	1751.79	1810.54	2993.88	3019.11
3035.50	3036.45	3046.94	3053.98	3057.67	3057.87
3060.38	3066.72	3067.30	3082.70	3106.90	3108.12
3108.45	3116.20	3117.06	3123.24	3128.92	3134.79
3156.52	3165.02	3170.12	3176.21	3188.04	3188.11
3207.22	3213.36	3230.16			

================================

B7

================================

37.93	45.42	52.09	58.11	63.43	69.64
72.02	79.35	86.69	93.64	102.70	112.86
119.75	126.94	143.81	147.27	169.49	179.56
185.86	195.18	210.25	224.41	231.71	246.81
253.88	262.75	274.51	284.65	285.38	310.19
319.94	333.15	335.49	337.98	369.78	381.55
390.22	397.91	417.46	430.42	437.58	460.24
475.01	496.09	509.58	523.39	528.21	543.89
557.93	568.56	602.09	618.41	637.19	645.87
663.92	684.13	696.04	709.47	753.28	777.76
814.61	830.18	843.19	864.85	885.04	891.16
916.45	921.75	938.32	940.48	942.28	949.96
958.96	977.31	985.74	1003.24	1006.01	1016.89
1021.72	1023.44	1030.13	1033.47	1059.59	1064.54
1067.07	1070.85	1074.82	1078.30	1084.10	1115.41
1128.43	1132.10	1144.77	1187.00	1189.39	1198.91
1228.61	1237.02	1238.75	1242.95	1260.24	1265.94
1297.03	1300.32	1309.98	1321.46	1328.90	1351.18

1358.51 1362.07 1377.07 1388.61 1392.78 1399.00

1404.17 1411.83 1423.30 1434.34 1443.35 1463.73

1472.46 1479.03 1479.92 1481.39 1488.50 1489.70

1492.36 1498.51 1499.01 1507.22 1508.56 1511.96

1517.90 1527.91 1534.59 1553.09 1630.49 1650.26

1699.15 1749.60 1865.21 2986.87 2988.79 3022.84

3036.07 3039.00 3047.09 3051.49 3054.51 3057.94

3058.88 3061.45 3075.26 3107.55 3114.70 3118.42

3119.35 3123.81 3130.02 3131.06 3132.50 3133.89

3164.02 3167.71 3180.57 3187.63 3189.99 3199.29

3205.10 3212.03 3228.88

================================

B7-TS

================================

-347.21 20.88 35.15 43.67 48.59 58.36

65.89 71.83 74.77 81.33 81.79 88.04

89.65 101.46 108.25 111.96 120.57 138.92

152.04 170.65 185.57 194.28 208.75 211.81

248.47 254.00 273.23 279.57 285.78 287.84

303.45 312.40 326.97 333.15 341.67 345.06

358.50 373.26 399.56 420.49 424.74 432.13

458.53 474.40 515.80 526.74 548.36 557.15

566.82 580.61 605.29 616.92 637.89 639.10

664.85 685.26 700.39 715.26 749.71 776.41

815.49 836.57 861.00 878.58 888.35 896.38

905.84 911.28 921.22 928.42 937.07 950.40

955.84 966.56 982.59 1005.06 1013.33 1017.07

1019.34 1022.96 1029.97 1032.81 1040.86 1056.28

1059.03 1065.72 1067.85 1069.52 1077.07 1098.71

1100.99 1133.79 1147.72 1187.59 1192.20 1204.00

1211.01 1230.07 1238.47 1247.51 1259.63 1269.58

1276.16 1300.85 1308.72 1311.57 1330.94 1346.92

1350.69 1354.29 1390.76 1394.36 1399.96 1401.30

1402.70 1419.94 1429.12 1438.87 1458.96 1465.21

1472.16 1472.79 1479.57 1480.11 1481.97 1489.39

1489.76 1501.89 1502.32 1506.92 1512.40 1512.89

1519.08 1529.98 1533.79 1551.95 1628.95 1646.20

1696.47 1739.16 1825.94 3024.61 3028.38 3035.40

3043.71 3047.46 3053.04 3053.81 3054.09 3056.51

3057.16 3057.39 3090.35 3098.51 3104.14 3104.31

3109.08 3113.08 3115.89 3129.91 3130.00 3132.84

3162.72 3167.82 3168.34 3172.19 3182.89 3199.52

3204.64 3213.78 3375.01

================================

B8

================================

24.59 32.27 39.55 43.03 46.41 59.18

59.76 70.63 84.58 87.48 89.30 93.97

101.52 115.79 123.05 125.98 137.67 152.12

165.15 171.20 196.37 198.52 204.80 225.58

234.96 250.10 252.50 264.87 277.67 290.04

305.68 308.37 312.79 326.68 335.71 346.60

362.03 368.62 416.05 420.15 438.41 457.39

478.07 523.07 534.86 549.91 555.15 604.49

608.40 613.83 622.19 655.37 665.24 680.86

692.85 696.68 711.49 729.16 742.15 776.54

806.69 817.56 850.29 863.52 878.71 887.42

906.51 924.69 931.47 932.88 943.16 949.74

956.97 975.98 981.89 988.07 995.75 1008.52

1018.07 1022.66 1025.89 1030.20 1037.32 1059.36

1059.58 1060.74 1065.68 1070.82 1095.09 1125.47

1141.87 1149.22 1201.36 1204.58 1212.36 1218.69

1228.74 1235.88 1243.74 1257.21 1274.27 1288.61

1296.05 1310.60 1318.65 1325.16 1335.47 1337.52

1354.06 1366.83 1369.67 1389.30 1392.41 1394.54

1396.82 1419.24 1419.37 1425.98 1456.08 1469.95

1476.09 1481.04 1482.00 1488.60 1488.81 1489.66

1500.28 1500.77 1511.45 1515.70 1520.40 1522.59

1532.91 1543.23 1548.03 1597.36 1620.59 1661.72

1725.85 1755.67 1793.28 3026.60 3039.73 3043.62

3052.15 3056.42 3057.66 3058.76 3066.45 3075.35

3076.57 3093.40 3107.41 3119.29 3121.90 3124.61

3126.65 3128.02 3132.59 3133.64 3148.16 3148.95

3155.81 3159.59 3163.37 3166.60 3197.18 3216.29

3221.74 3242.94 3601.70

==============================

B6-TS'

==============================

-197.96 37.28 39.60 45.45 53.62 61.46

67.99 79.00 86.42 92.58 100.25 104.32

113.96 121.83 124.02 151.52 158.89 164.05

173.45 188.00 207.39 212.12 223.51 239.81

255.90 278.44 279.91 284.20 298.40 301.16

316.79 323.54 332.05 348.85 363.12 381.41

383.74 409.01 414.07 419.48 439.52 451.52

458.06 474.49 491.83 529.92 546.39 548.59

557.72 576.88 602.06 609.95 640.67 649.44

655.36 675.43 694.53 728.72 747.14 773.20

806.07 808.47 861.60 875.31 882.09 899.56

908.83 913.02 930.56 933.87 949.51 950.19

956.92 971.40 984.11 985.43 995.19 1009.96

1020.54 1023.22 1027.36 1050.93 1057.05 1062.38

1065.48 1072.24 1072.77 1079.62 1097.16 1116.72

1122.52 1138.98 1141.46 1190.17 1201.21 1208.88

1234.58 1241.12 1248.60 1251.24 1253.55 1300.46

1303.47 1306.80 1309.68 1327.48 1346.52 1357.05

1366.84 1375.59 1388.59 1391.02 1403.60 1413.98

1417.93 1420.83 1422.17 1437.58 1452.08 1468.86

1472.51 1480.99 1481.54 1481.70 1484.60 1487.41

1501.17 1501.87 1502.58 1506.56 1517.09 1519.39

1525.94 1527.23 1528.78 1552.11 1628.05 1642.99

1656.31 1715.50 1731.09 1742.65 2964.71 2998.93

3020.58 3027.00 3033.65 3039.31 3052.00 3056.29

3057.38 3058.74 3062.25 3073.71 3088.27 3094.45

3096.59 3100.88 3104.96 3107.51 3129.80 3134.42

3142.71 3145.55 3147.31 3164.94 3172.83 3189.23

3209.73 3214.92 3230.36

==============================

B7'

==============================

33.65 43.34 45.27 51.64 54.08 61.78

67.63 75.25 85.56 108.50 108.63 115.62

124.48 130.76 150.62 160.14 169.85 171.37

177.14 194.72 211.83 218.47 224.23 242.24

254.40 263.11 279.78 283.36 293.37 298.48

304.77 313.33 320.49 328.82 357.16 371.36

378.75 405.94 418.84 428.29 438.04 456.32

467.56 487.23 491.33 530.66 545.78 551.21

562.59 596.03 602.02 609.13 643.67 653.24

665.34 674.90 696.70 741.26 757.01 775.96

806.41 810.57 865.76 876.48 891.10 909.67

915.94 923.62 933.43 937.23 950.81 951.27

978.46 980.82 986.54 992.69 1011.61 1018.10

1021.53 1027.31 1048.01 1048.82 1060.02 1061.54

1065.04	1070.65	1073.02	1079.89	1110.60	1116.01
1122.81	1139.04	1191.02	1205.41	1208.90	1235.12
1238.27	1249.06	1250.18	1254.86	1287.08	1301.29
1305.53	1311.30	1316.60	1326.25	1344.00	1355.16
1366.79	1374.27	1386.54	1390.20	1390.84	1409.27
1418.67	1422.90	1423.30	1436.28	1453.07	1469.86
1472.85	1478.75	1480.66	1480.88	1485.21	1488.32
1490.86	1496.46	1505.28	1506.53	1506.91	1516.30
1523.93	1525.11	1529.95	1542.05	1627.96	1654.35
1657.10	1732.21	1738.82	1778.60	2960.47	2998.99
3026.61	3034.71	3042.60	3044.71	3050.78	3053.86
3059.80	3060.95	3063.75	3075.20	3100.75	3102.61
3111.36	3114.57	3120.44	3124.18	3135.06	3139.68
3144.26	3146.63	3149.99	3169.15	3178.75	3189.40
3211.53	3218.06	3234.97			

===============================

B7'-TS

===============================

-368.40	40.41	47.54	66.61	69.23	81.92
86.81	89.66	103.03	104.76	113.93	127.37
128.91	149.96	152.89	160.20	171.61	180.37
191.77	202.80	208.19	211.65	229.88	234.28
243.78	250.00	267.98	288.96	293.83	308.37
314.79	317.15	333.26	338.68	351.69	368.67
376.55	390.26	420.36	428.28	432.25	450.91
471.63	490.84	502.33	508.82	540.81	564.50
567.64	588.37	618.82	620.25	635.43	641.93
657.12	660.00	683.27	715.80	756.26	784.39
823.82	843.29	853.63	872.74	891.08	895.06
903.20	912.44	913.31	929.89	948.21	953.77
957.88	978.29	988.35	1002.47	1008.21	1012.44

1023.39	1029.99	1032.02	1034.15	1041.80	1049.67
1062.45	1068.40	1076.58	1077.18	1079.91	1087.38
1122.80	1147.87	1155.51	1177.09	1194.51	1211.36
1214.81	1233.63	1238.89	1244.73	1252.25	1259.73
1263.21	1269.03	1288.48	1307.53	1319.17	1350.80
1355.79	1383.12	1388.35	1389.92	1395.31	1400.76
1410.73	1418.53	1419.34	1427.28	1456.24	1463.81
1464.23	1474.28	1477.13	1479.07	1486.44	1488.97
1493.61	1495.25	1502.52	1504.64	1505.12	1508.91
1514.47	1521.90	1526.91	1544.05	1591.60	1610.80
1636.60	1653.27	1687.56	2613.80	2972.37	2976.20
2979.50	2982.64	2986.52	2994.62	3003.23	3009.90
3014.21	3014.89	3016.80	3021.25	3037.08	3046.41
3050.80	3052.44	3053.45	3059.64	3074.40	3075.72
3077.64	3078.61	3096.97	3102.33	3119.70	3123.15
3133.61	3146.76	3152.70			

===============================

B7''

===============================

31.98	41.70	44.08	50.21	62.97	66.13
77.49	79.36	82.30	88.62	92.70	98.43
108.64	126.10	134.58	146.21	148.35	170.83
180.14	188.67	210.28	221.10	230.60	235.44
252.88	260.26	268.06	281.92	286.06	297.11
301.37	316.54	322.28	328.68	352.57	360.75
396.20	405.42	410.80	424.38	431.28	442.94
470.54	481.70	495.31	535.53	541.95	549.64
549.97	571.09	602.47	603.44	635.75	655.40
660.54	674.49	688.06	723.29	740.49	773.50
805.76	813.72	854.87	865.27	899.72	913.32
918.02	928.95	933.61	934.71	951.44	953.41

974.08 980.03 983.03 993.74 1013.91 1024.06

1029.95 1034.44 1049.87 1054.66 1058.74 1066.05

1071.70 1078.11 1086.09 1091.25 1109.19 1120.82

1132.13 1140.93 1196.22 1199.08 1206.21 1217.73

1237.97 1243.87 1254.78 1257.83 1270.25 1284.01

1285.24 1303.08 1313.08 1328.86 1342.66 1346.08

1367.81 1381.22 1383.03 1385.22 1386.51 1404.32

1412.69 1417.77 1419.13 1422.07 1450.05 1464.13

1471.38 1477.28 1482.82 1485.37 1488.25 1491.78

1492.63 1493.41 1508.16 1510.33 1513.49 1520.17

1522.57 1529.92 1540.86 1545.62 1623.01 1660.01

1689.24 1738.73 1776.31 2559.84 2915.77 2996.01

3010.87 3032.91 3035.78 3037.11 3044.65 3045.76

3054.73 3055.12 3059.64 3067.12 3092.70 3101.43

3101.88 3118.07 3119.87 3124.31 3132.04 3138.34

3142.90 3147.75 3166.69 3169.01 3183.05 3186.84

3193.06 3217.35 3235.36

==============================

B7''-TS

==============================

-281.16 17.58 36.22 42.14 56.91 61.36

66.38 71.49 73.56 78.38 82.48 88.66

98.79 113.29 125.48 128.99 141.85 154.34

165.82 168.87 184.93 190.68 200.68 210.98

227.94 244.09 253.65 258.48 262.41 284.62

293.71 296.86 299.14 323.70 333.43 341.30

363.26 382.17 386.18 408.02 418.11 424.05

442.07 466.52 494.54 536.14 541.08 560.91

573.72 594.42 606.45 610.68 626.44 635.71

667.47 682.97 684.89 733.83 758.05 766.53

820.88 831.44 865.40 880.00 892.07 903.94

907.00 918.96 925.05 927.37 949.86 951.23

956.86 972.88 981.04 982.42 1003.51 1005.01

1013.46 1031.30 1040.85 1045.20 1050.61 1060.65

1064.17 1065.44 1069.77 1075.35 1078.17 1101.06

1107.88 1132.95 1147.07 1188.30 1196.33 1209.73

1211.10 1245.40 1246.59 1253.13 1266.77 1272.17

1289.19 1299.85 1301.81 1326.19 1340.06 1356.78

1367.29 1377.19 1378.50 1381.41 1387.69 1391.58

1397.98 1416.99 1419.47 1422.13 1430.37 1450.54

1473.41 1476.44 1485.45 1489.81 1491.76 1493.64

1499.07 1501.15 1502.84 1508.12 1513.99 1514.78

1521.96 1525.37 1539.36 1553.87 1624.70 1646.36

1698.35 1703.36 1712.45 2586.28 2965.43 3012.30

3028.92 3035.02 3036.72 3041.36 3050.67 3050.86

3063.81 3073.09 3086.79 3099.37 3103.26 3105.31

3105.49 3107.83 3110.72 3116.96 3129.56 3136.29

3139.83 3148.49 3156.91 3163.65 3179.43 3181.48

3198.73 3201.18 3210.09

==============================

B7'''

==============================

20.44 39.04 43.88 57.23 59.23 60.02

66.65 69.66 78.62 83.41 87.10 109.30

115.19 137.59 141.31 142.67 155.82 167.23

182.41 197.62 204.56 214.75 225.10 242.56

253.01 258.50 266.38 271.50 278.21 288.85

293.25 308.64 319.17 337.98 348.44 357.73

376.48 408.49 415.03 427.48 436.47 471.12

489.74 516.83 525.66 530.40 534.23 543.07

559.08 600.55 608.55 620.51 628.52 654.67

670.97 684.96 693.11 720.17 745.29 771.35

776.12 803.29 837.67 857.39 898.80 908.24

918.62 931.30 936.13 939.00 944.67 950.72

955.47 983.18 984.71 992.72 1012.26 1027.78

1032.97 1034.17 1040.42 1041.81 1060.29 1061.69

1064.81 1068.96 1073.36 1081.35 1092.32 1114.02

1137.03 1140.90 1170.08 1197.71 1200.66 1211.16

1231.28 1236.79 1248.17 1258.73 1262.33 1278.76

1289.75 1309.64 1312.34 1314.14 1332.49 1337.68

1342.40 1374.24 1380.74 1386.81 1393.48 1402.26

1418.57 1420.96 1421.42 1427.18 1457.43 1460.45

1477.09 1478.56 1482.19 1482.92 1489.81 1491.70

1493.05 1500.58 1503.42 1505.71 1514.17 1522.11

1527.02 1534.34 1548.81 1568.07 1629.47 1646.50

1657.95 1760.87 1841.67 2894.79 2972.10 2992.13

3001.94 3032.95 3042.29 3045.27 3045.64 3051.10

3058.52 3059.25 3080.27 3105.91 3108.84 3110.06

3114.74 3118.95 3126.14 3131.58 3133.39 3133.68

3137.68 3148.90 3156.65 3167.88 3173.45 3189.15

3194.94 3218.69 3254.16

==============================

B7'''-TS

==============================

-265.36 11.66 33.97 55.06 61.86 66.40

73.87 77.03 80.06 84.60 90.81 97.30

105.29 111.17 121.99 129.50 138.62 157.04

164.77 169.41 185.49 196.05 220.31 230.08

240.97 248.93 260.23 270.35 273.71 278.26

285.74 304.17 313.01 318.92 325.83 345.68

360.89 411.53 412.93 418.48 428.20 441.03

459.61 475.96 525.31 526.58 539.59 553.90

567.23 583.17 606.81 610.62 628.94 638.92

669.74 685.28 688.23 700.75 752.06 770.67

817.15 821.22 852.89 872.72 882.53 893.99

906.26 916.26 925.96 937.93 947.69 951.77

954.31 957.00 978.17 986.82 999.18 1007.75

1009.22 1013.44 1033.53 1037.04 1040.35 1052.00

1062.17 1062.78 1067.12 1068.28 1071.64 1084.21

1123.31 1131.75 1151.90 1190.38 1202.93 1209.65

1211.77 1226.25 1246.97 1259.75 1262.42 1273.68

1278.21 1303.88 1308.36 1323.80 1339.66 1341.59

1352.23 1359.57 1369.80 1378.64 1388.18 1395.23

1409.40 1420.87 1423.65 1430.09 1431.38 1459.91

1469.50 1475.55 1479.82 1481.39 1487.33 1490.42

1491.74 1495.80 1498.27 1500.97 1507.47 1510.55

1517.67 1529.31 1530.07 1541.88 1626.76 1648.75

1661.53 1729.13 1822.62 2942.27 2997.85 3000.47

3035.86 3046.18 3048.71 3053.39 3057.76 3057.82

3066.31 3080.10 3104.39 3104.77 3107.78 3117.39

3118.42 3121.37 3122.80 3124.29 3131.15 3133.49

3139.17 3154.69 3166.55 3170.87 3177.03 3188.88

3210.00 3216.84 3221.32

VI. Reference

(1) D. Morton, D. Pearson, R. A. Field, R. A. Stockman, *Synlett* **2003**, *13*, 1985.

(2) (a) S. D. Kuduk, R. M. DiPardo, R. K. Chang, C. Ng, M. G. Bock, *Tetrahedron Lett.* **2004**, *45*, 6641; (b) K. R. Prasad, O. Revu, *Tetrahedron* **2013**, *69*, 8422.

(3) B. Li, M. Driess, J. F. Hartwig, *J. Am. Chem. Soc.* **2014**, *136*, 6586.

(4) P. A. Evans, J. D. Roseman, L. T. Garber, *Synth. Commun.* **1996**, *26*, 4685.

(5) S. Lee, P. S. J. Kaib, B. List, *J. Am. Chem. Soc.* **2017**, *139*, 2156.

(6) Parr, R. G.; Yang, W., Density Functional Theory of Atoms and Molecules. Oxford University Press: New York, **1989**.

(7) Bochevarov, A. D.; Harder, E.; Hughes, T. F.; Greenwood, J. R.; Braden, D. A.; Philipp, D. M.; Rinaldo, D.; Halls, M. D.; Zhang, J.; Friesner, R. A., *Int. J. Quantum Chem.* **2013**, *113*, 2110.

(8) (a) Marten, B.; Kim, K.; Cortis, C.; Friesner, R. A.; Murphy, R. B.; Ringnalda, M. N.; Sitkoff, D.; Honig, B., *J. Phys. Chem.* **1996**, *100*, 11775. (b) Edinger, S. R.; Cortis, C.; Shenkin, P. S.; Friesner, R. A., *J. Phys. Chem. B* **1997**, *101*, 1190. (c) Friedrichs, M.; Zhou, R. H.; Edinger, S. R.; Friesner, R. A., *J. Phys. Chem. B* **1999**, *103*, 3057.

(9) Grimme, S.; Antony, J.; Ehrlich, S. & Krieg, H. *J. Chem. Phys.* **2010**, 132, 154104.

(10) Ditchfield, R.; Hehre, W. J.; Pople, J. A., *J. Chem. Phys.* **1971**, *54*, 724.

(11) (a) Hay, P. J.; Wadt, W. R., *J. Chem. Phys*. **1985**, 82, 270. (b) Wadt, W. R.; Hay, P. J., *J. Chem. Phys*. **1985**, 82, 284. (c) Hay, P. J.; Wadt, W. R., *J. Chem. Phys*. **1985**, *82*, 299.

(12) Dunning, T. H., Jr., *J. Chem. Phys.* **1989**, *90*, 1007.

(13) (a) Marten, B.; Kim, K.; Cortis, C.; Friesner, R. A.; Murphy, R. B.; Ringnalda, M. N.; Sitkoff, D.; Honig, B., *J. Phys. Chem.* **1996**, *100*, 11775. (b) Edinger, S. R.; Cortis, C.; Shenkin, P. S.; Friesner, R. A., *J. Phys. Chem. B* **1997**, *101*, 1190. (c) Friedrichs, M.; Zhou, R. H.; Edinger, S. R.; Friesner, R. A., *J. Phys. Chem. B* **1999**, *103*, 3057.

Appendix I

Spectral Copies of ^{1}H, ^{13}C NMR Data Obtained in this Study

***N*-((*S*)-2,2-dimethyl-1-(pyridin-2-yl)propyl)-2-((*S*)-tetrahydrofuran-2-yl)acetamide (2a).**

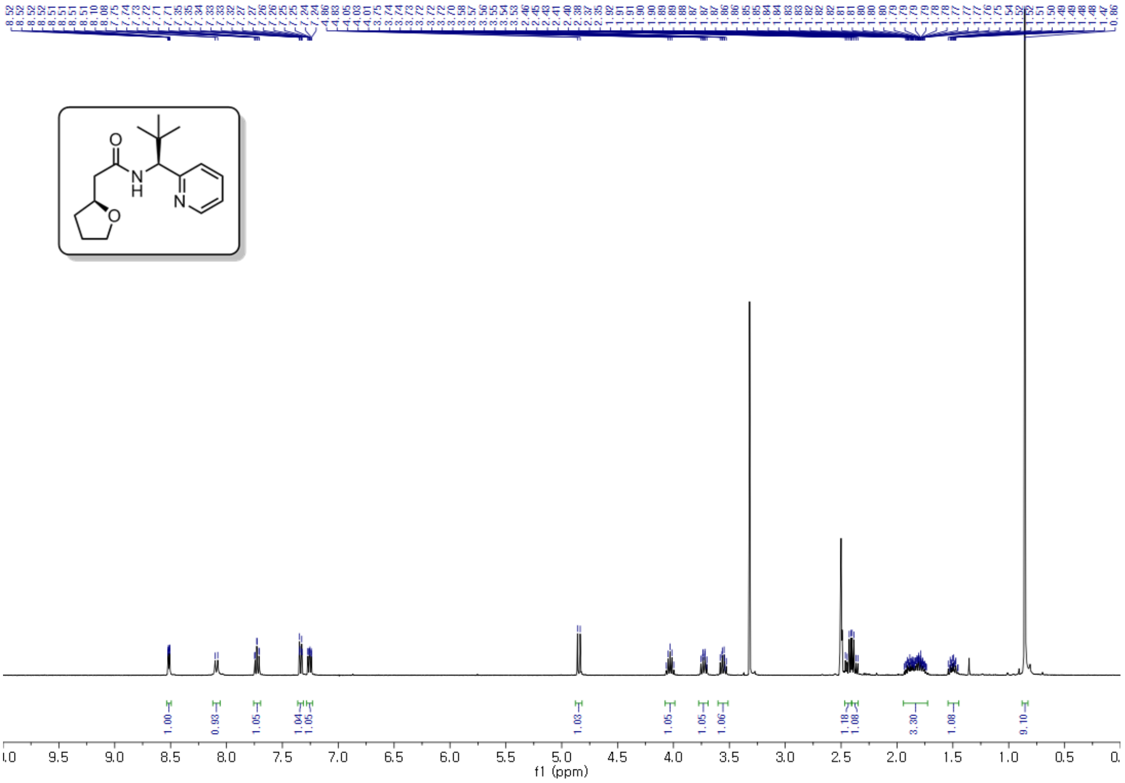

400 MHz, ^{1}H NMR in DMSO-d_6

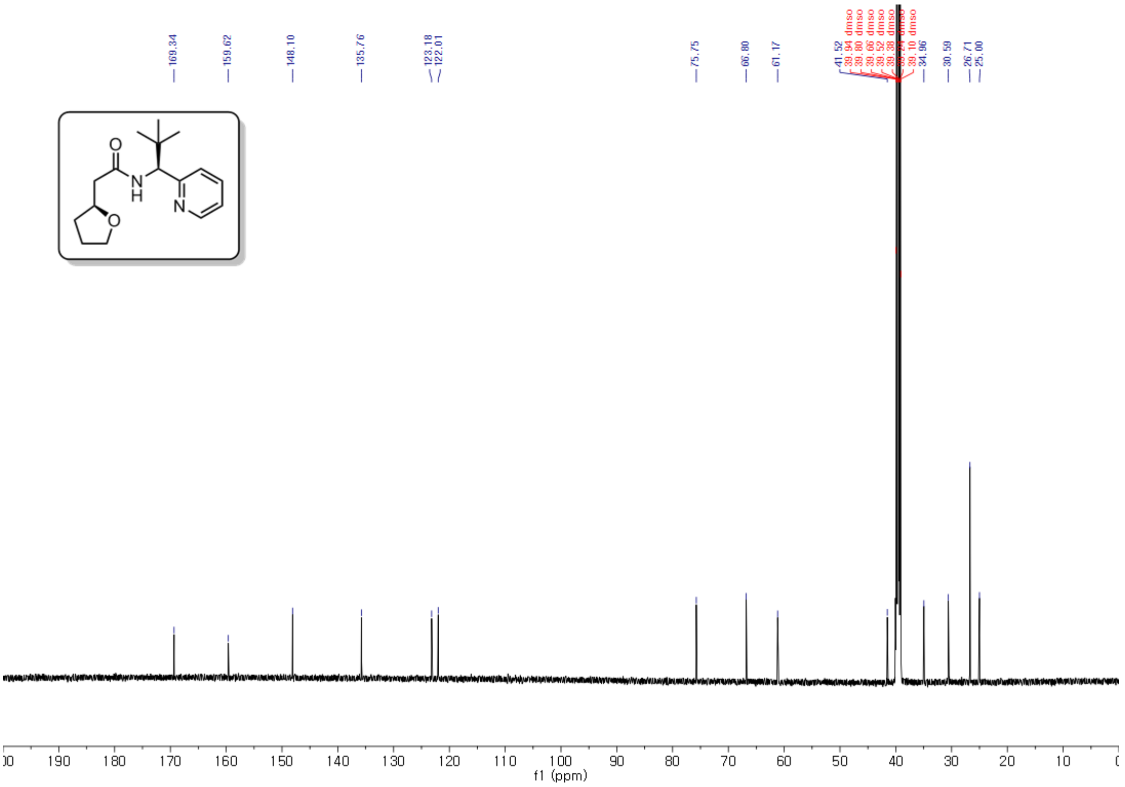

151 MHz, ^{13}C NMR in DMSO-d_6

***N*-((*S*)-2,2-dimethyl-1-(pyridin-2-yl)propyl)-2-((*S*)-5,5-dimethyltetrahydrofuran-2-yl)acetamide (2b).**

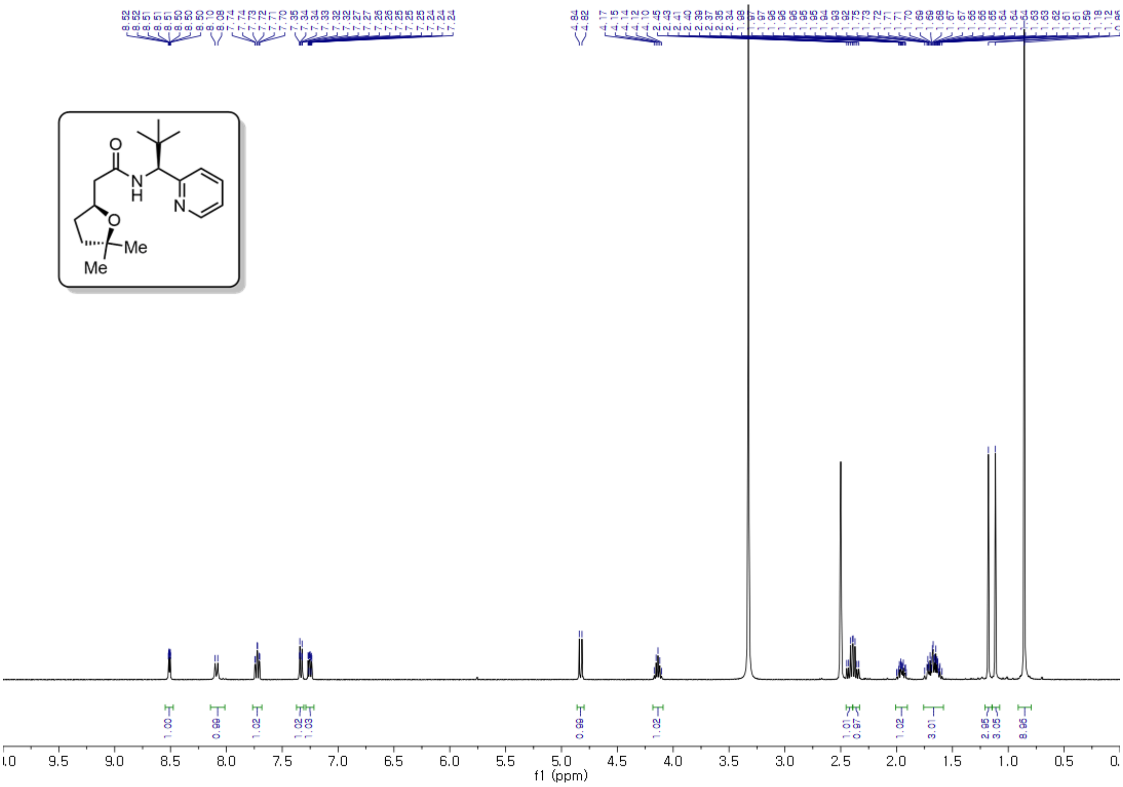

400 MHz, ^{1}H NMR in DMSO-d_6

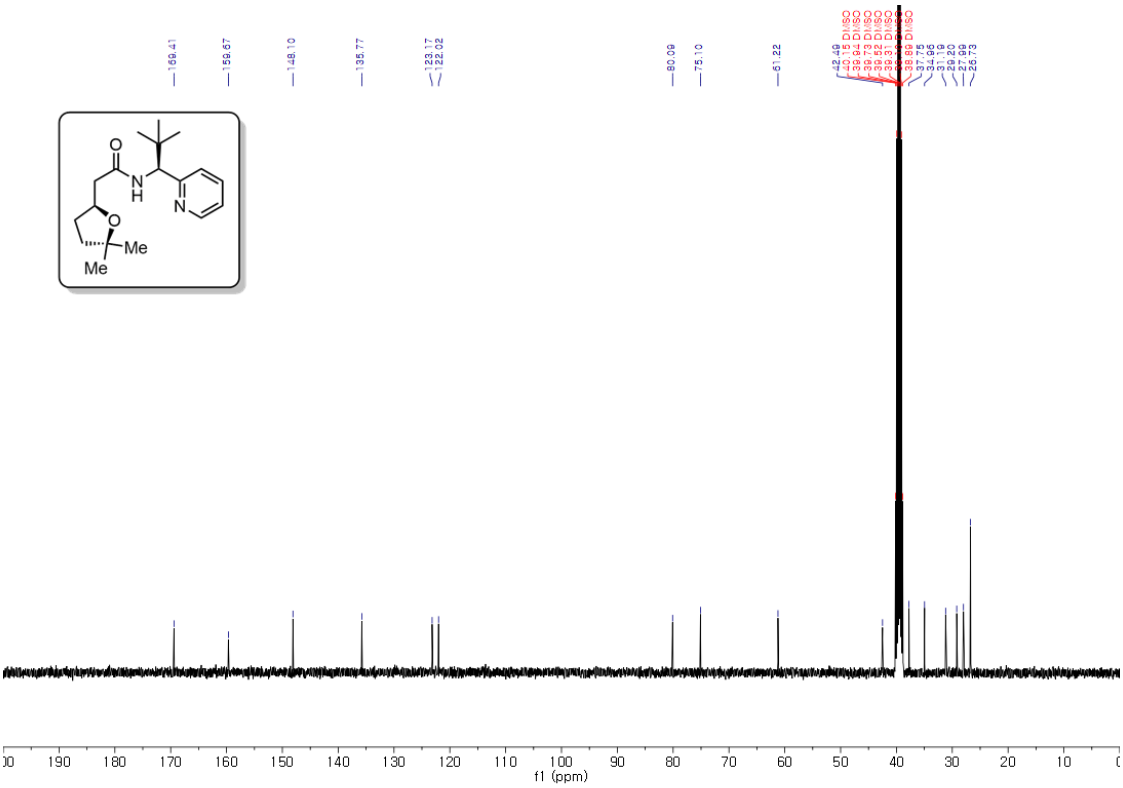

151 MHz, ^{13}C NMR in DMSO-d_6

N-((S)-2,2-dimethyl-1-(pyridin-2-yl)propyl)-2-((S)-5-oxaspiro[3.4]octan-6-yl)acetamide (2c).

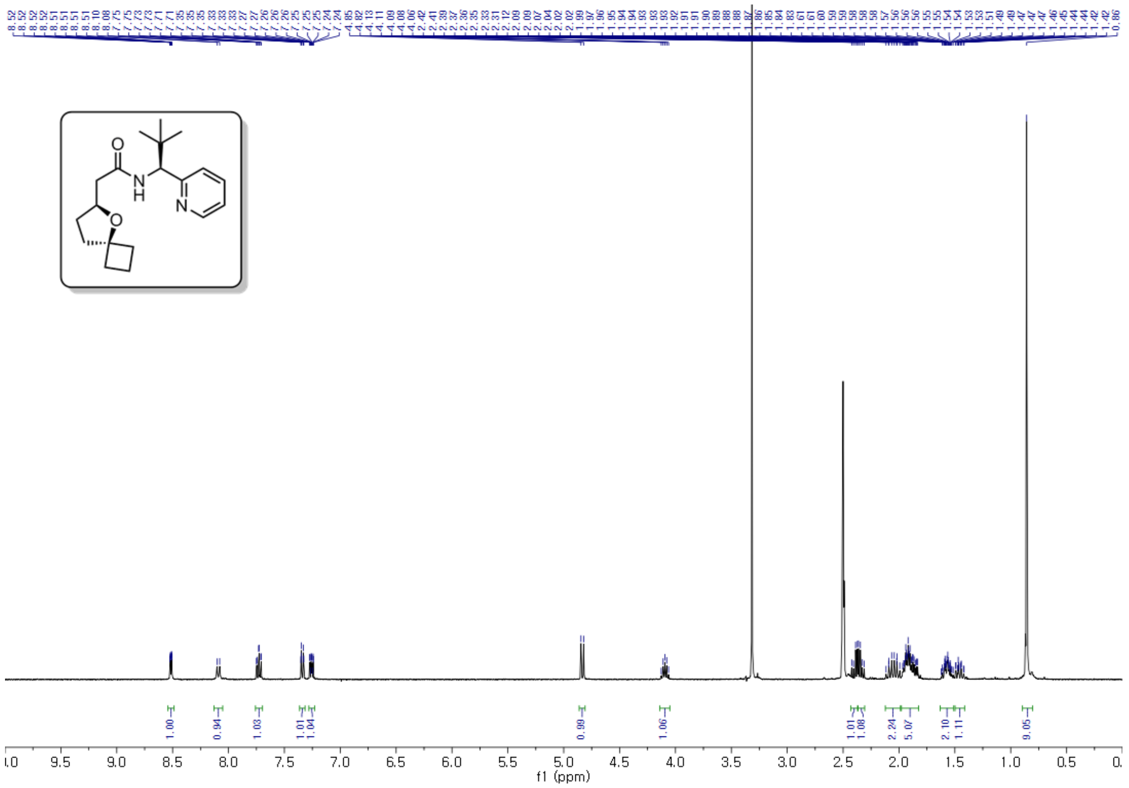

400 MHz, ^{1}H NMR in DMSO-d_6

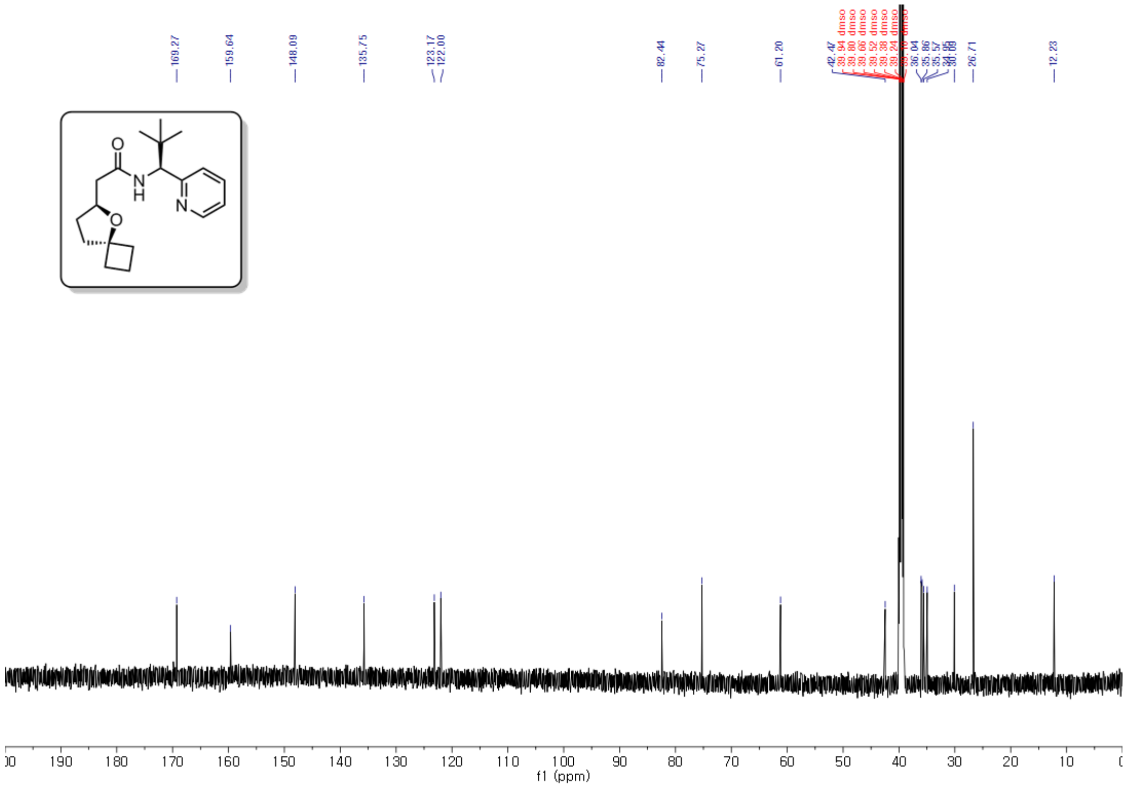

151 MHz, ^{13}C NMR in DMSO-d_6

***N*-((*S*)-2,2-dimethyl-1-(pyridin-2-yl)propyl)-2-((2*S*,5*S*)-5-methyltetrahydrofuran-2-yl)acetamide (2d).**

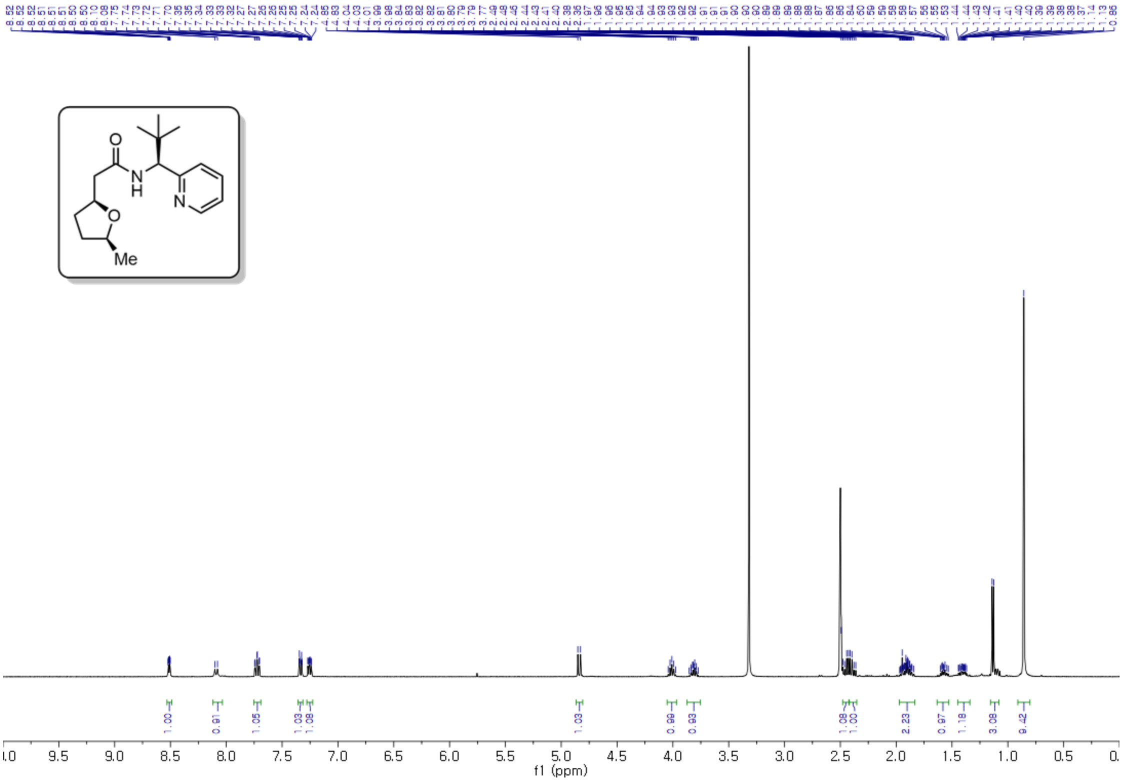

400 MHz, ^{1}H NMR in DMSO-d_6

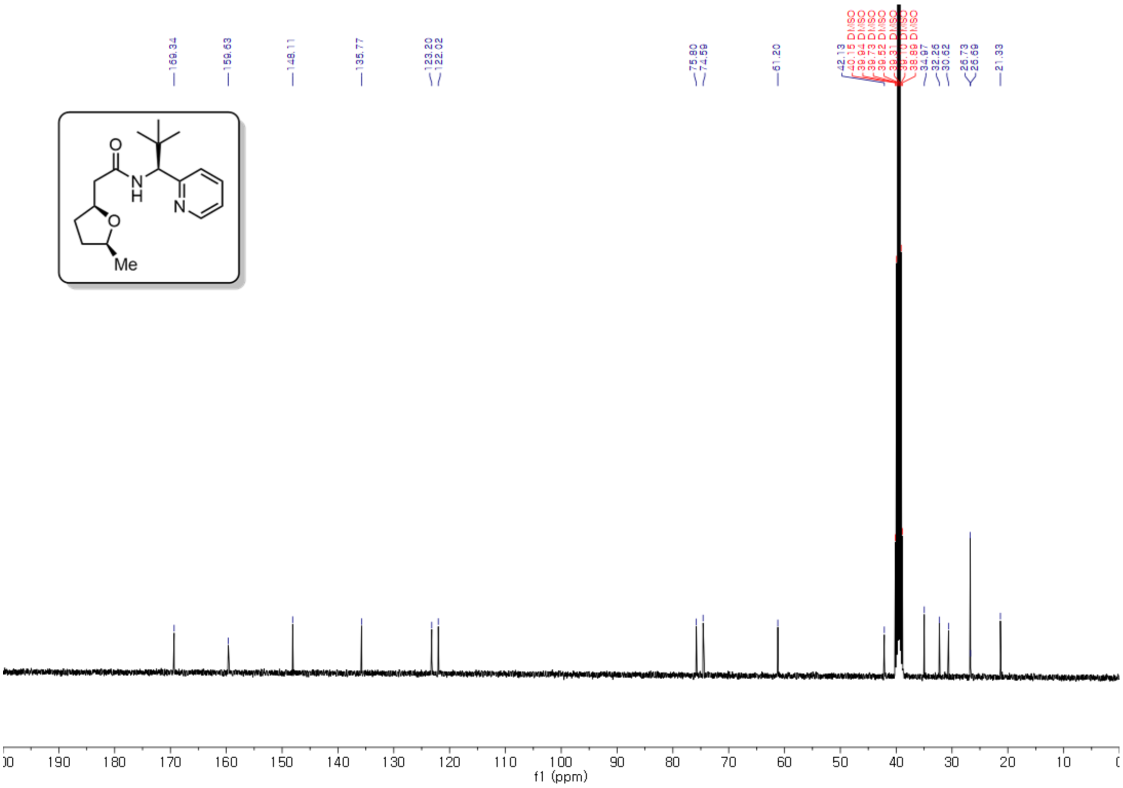

151 MHz, ^{13}C NMR in DMSO-d_6

N-((*S*)-2,2-dimethyl-1-(pyridin-2-yl)propyl)-2-((2*S*,5*R*)-5-methyltetrahydrofuran-2-yl)acetamide **(2e).**

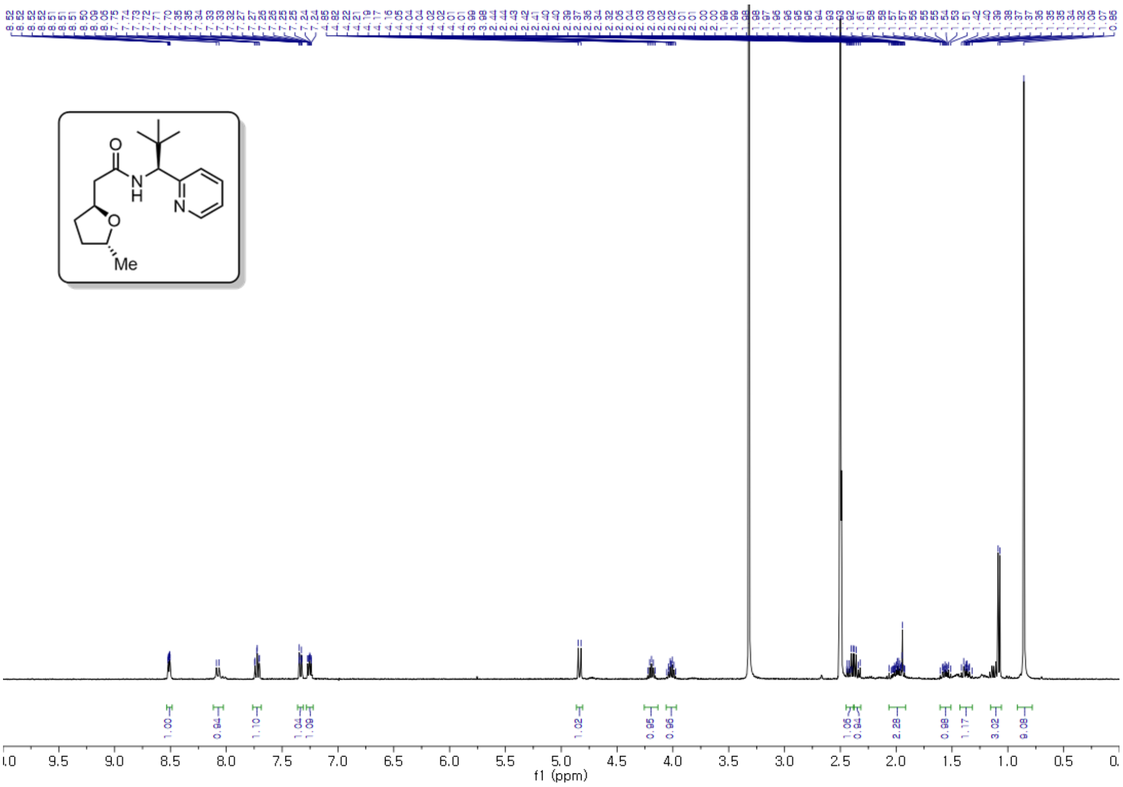

400 MHz, ^{1}H NMR in DMSO-d_6

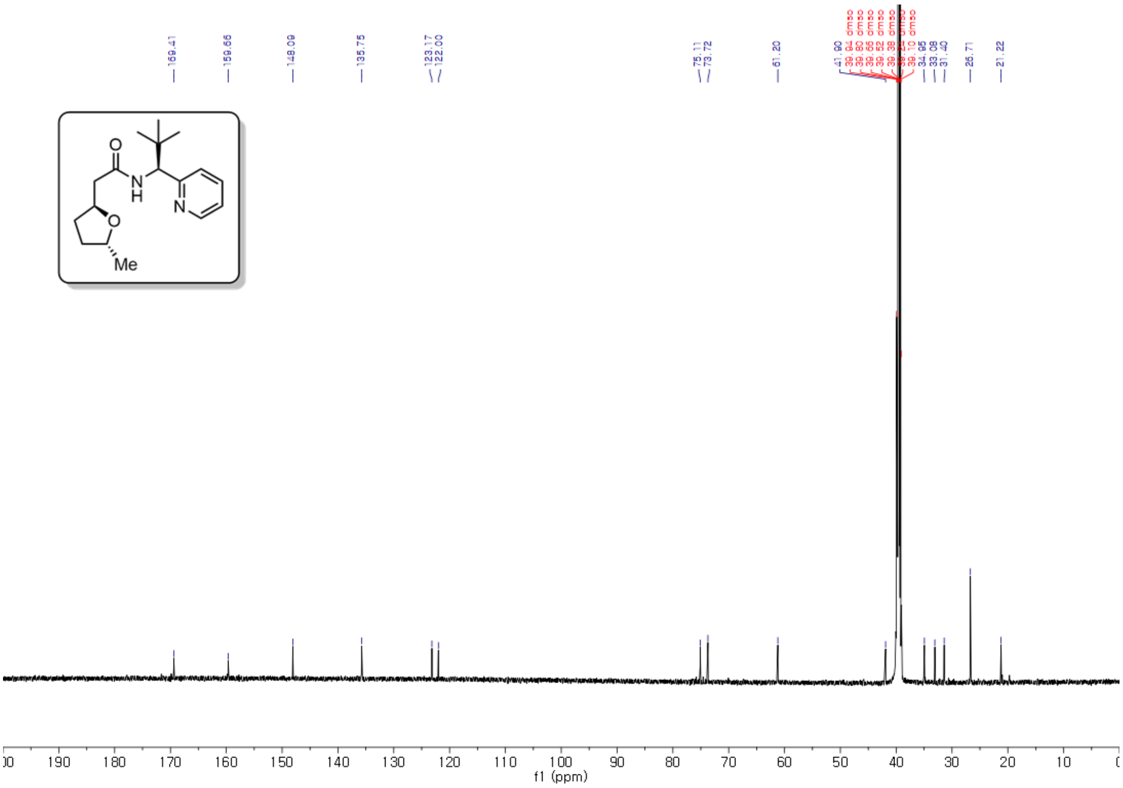

151 MHz, ^{13}C NMR in DMSO-d_6

***tert*-butyl (*S*)-6-(2-(((*S*)-2,2-dimethyl-1-(pyridin-2-yl)propyl)amino)-2-oxoethyl)-5-oxa-2-azaspiro[3.4]octane-2-carboxylate (2f).**

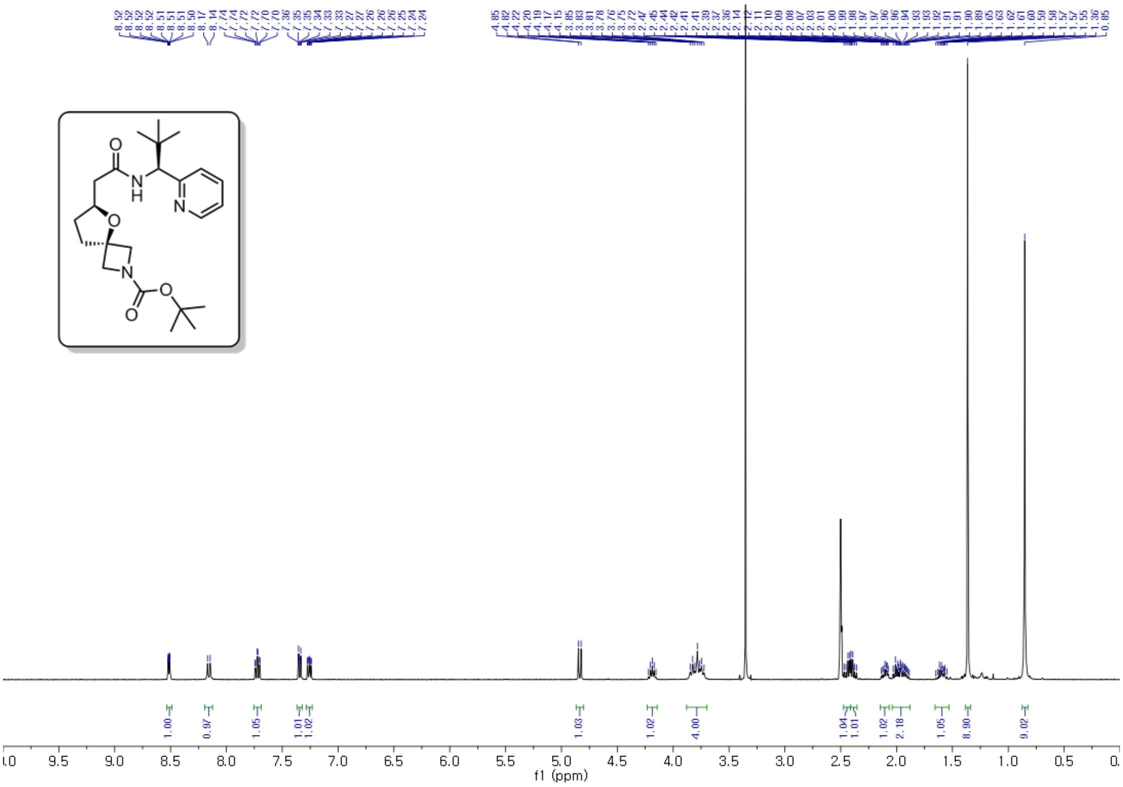

400 MHz, ^{1}H NMR in DMSO-d_6

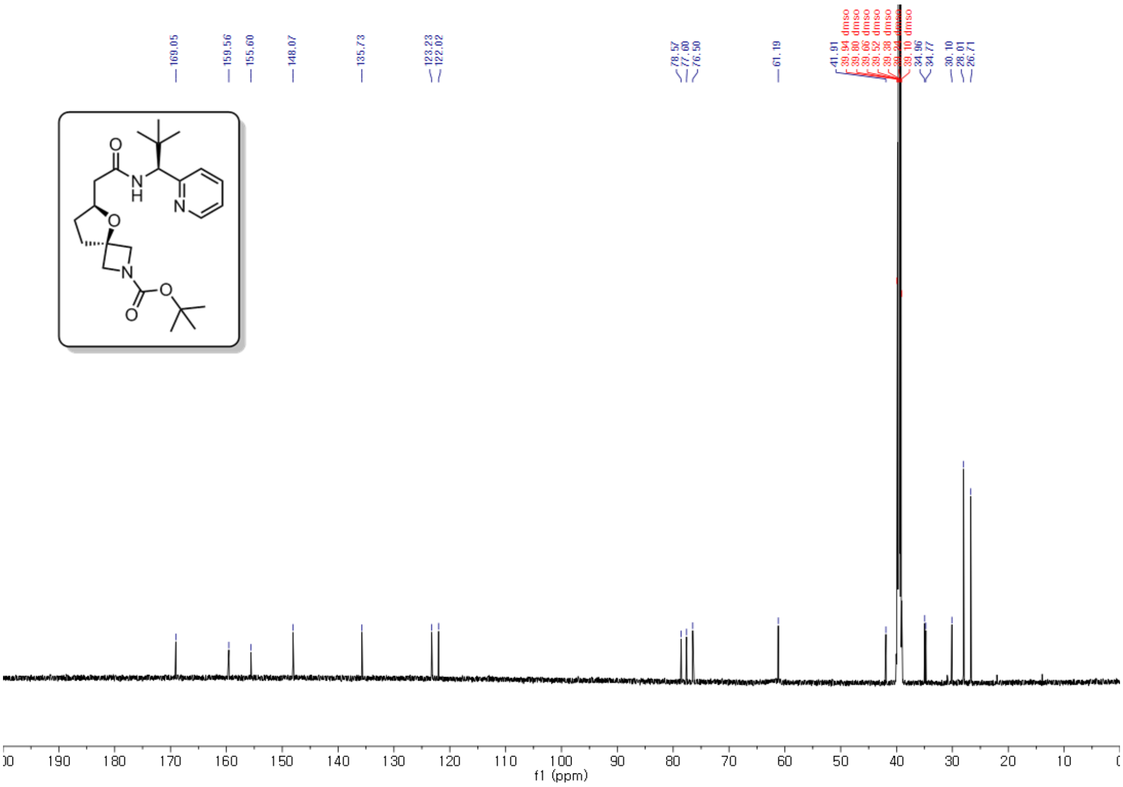

151 MHz, ^{13}C NMR in DMSO-d_6

2-((*S*)-2-acetyl-5-oxa-2-azaspiro[3.4]octan-6-yl)-*N*-((*S*)-2,2-dimethyl-1-(pyridin-2-yl)propyl)acetamide (2g).

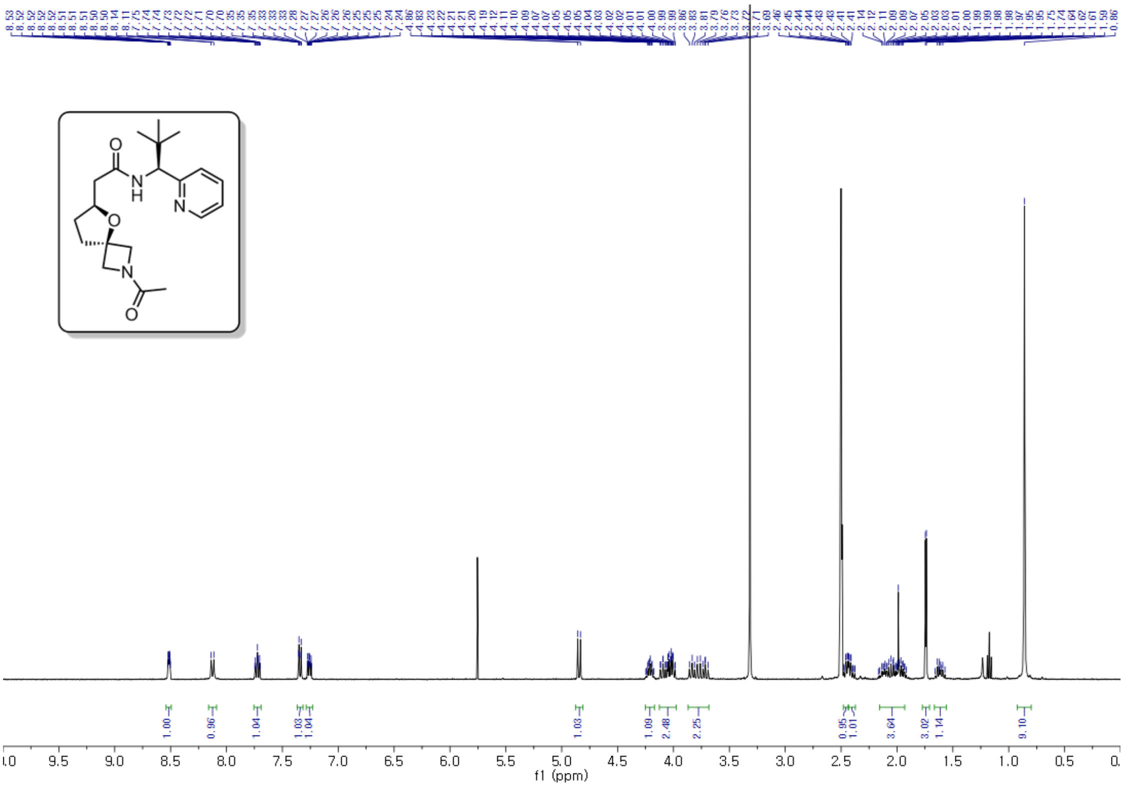

400 MHz, ^{1}H NMR in DMSO-d_6

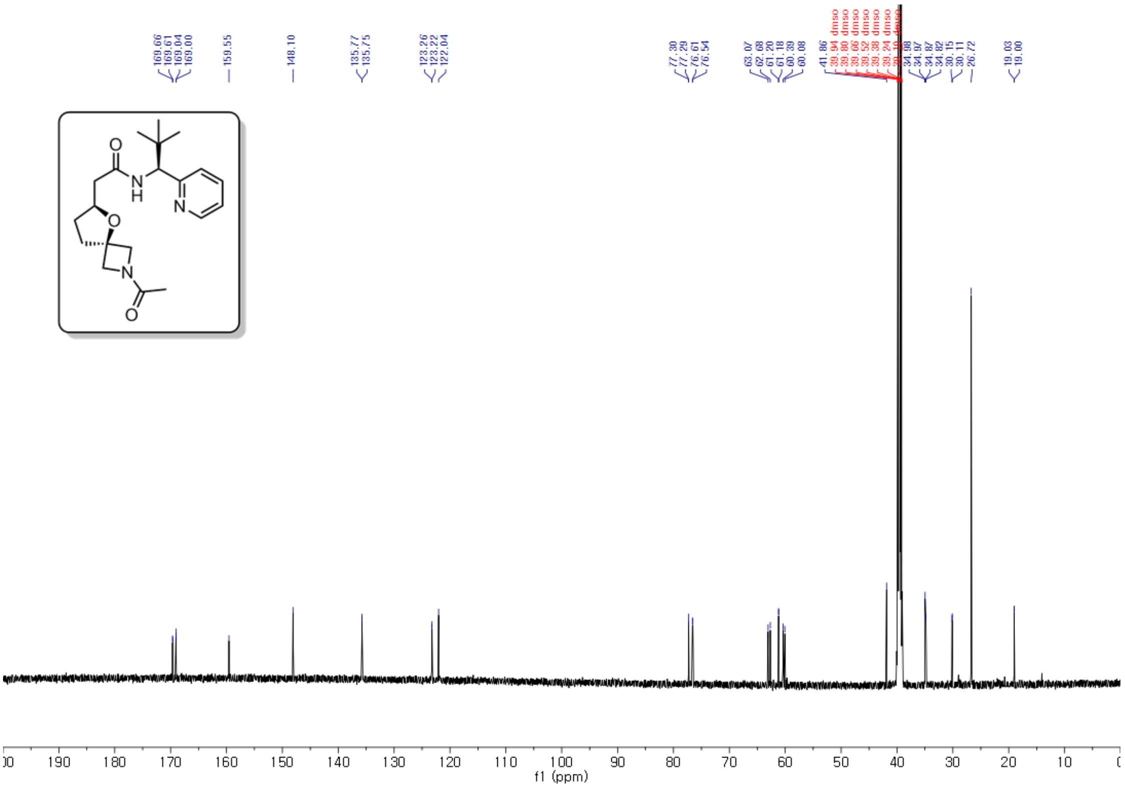

151 MHz, ^{13}C NMR in DMSO-d_6

***N*-((*S*)-2,2-dimethyl-1-(pyridin-2-yl)propyl)-2-((*S*)-2-(methylsulfonyl)-5-oxa-2-azaspiro[3.4]octan-6-yl)acetamide (2h).**

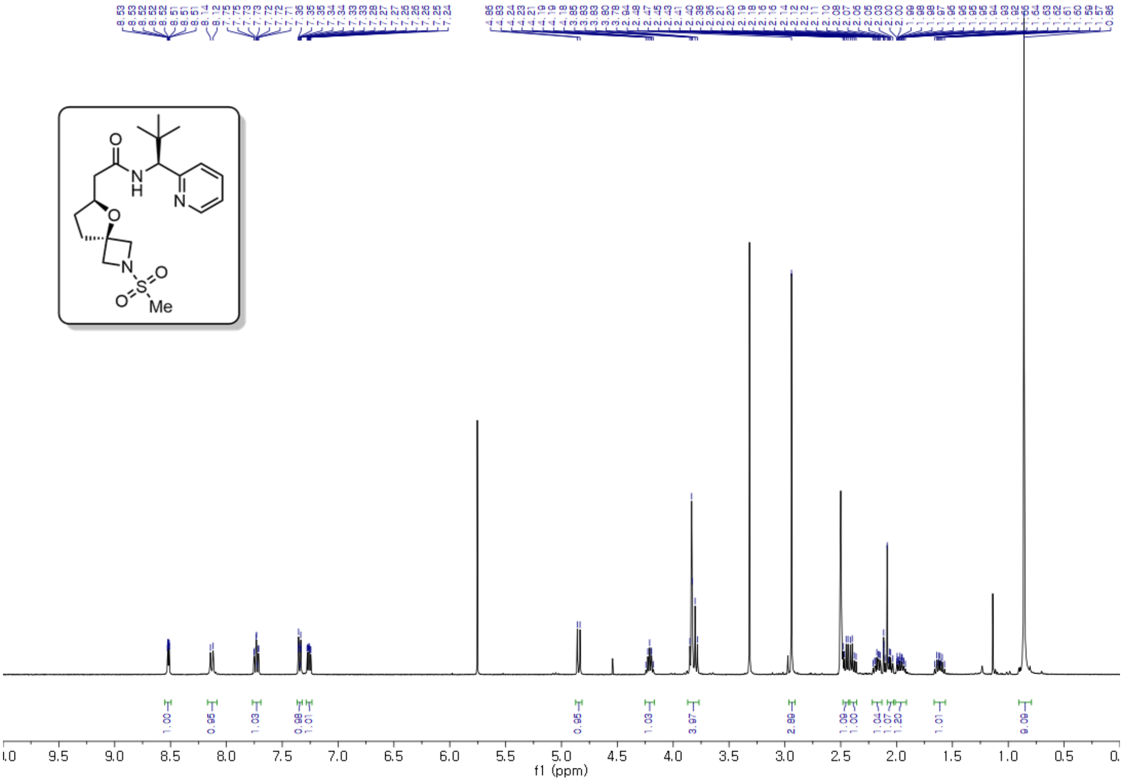

400 MHz, ^{1}H NMR in DMSO-d_6

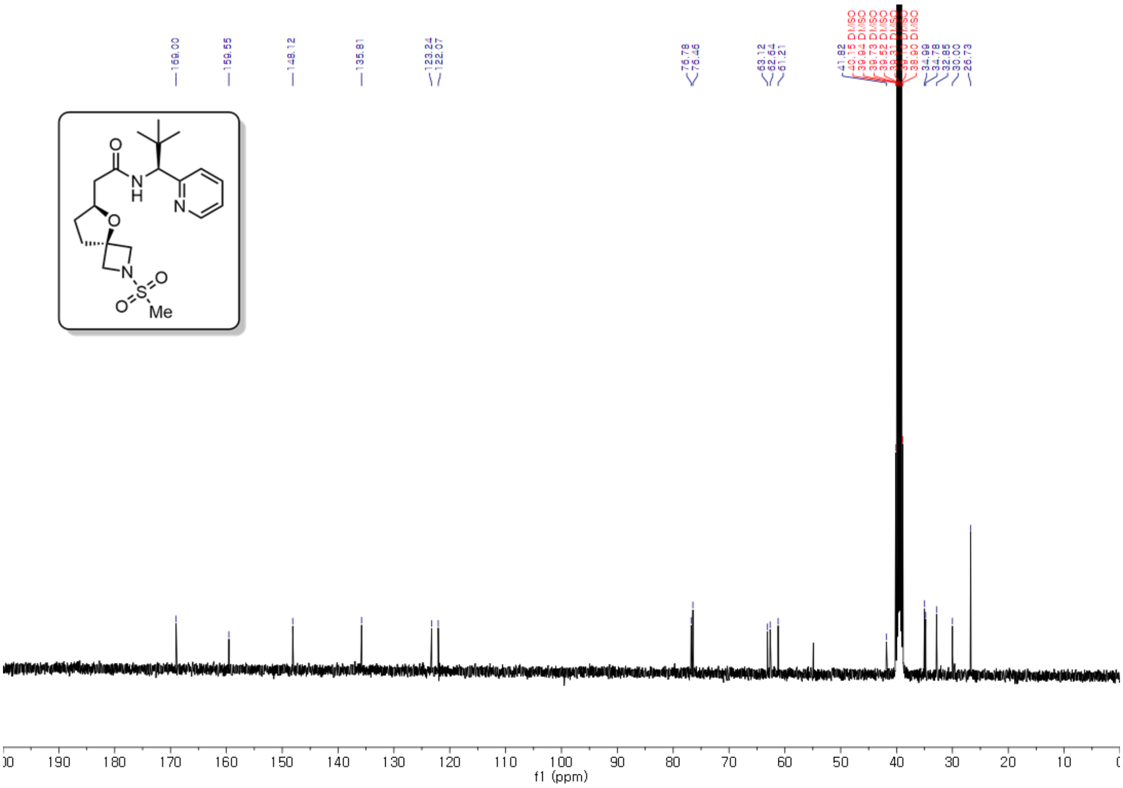

151 MHz, ^{13}C NMR in DMSO-d_6

***N*-((*S*)-2,2-dimethyl-1-(pyridin-2-yl)propyl)-2-((*S*)-2-tosyl-5-oxa-2-azaspiro[3.4]octan-6-yl)acetamide (2i).**

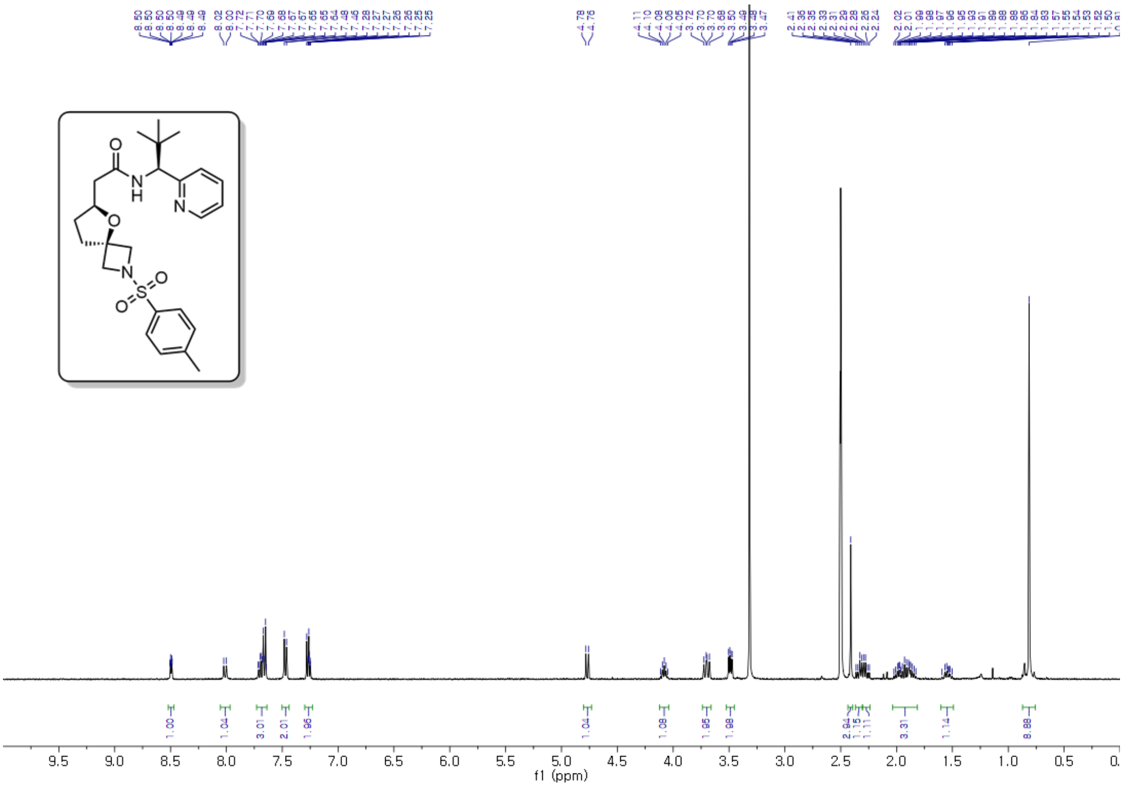

400 MHz, ^{1}H NMR in DMSO-d_6

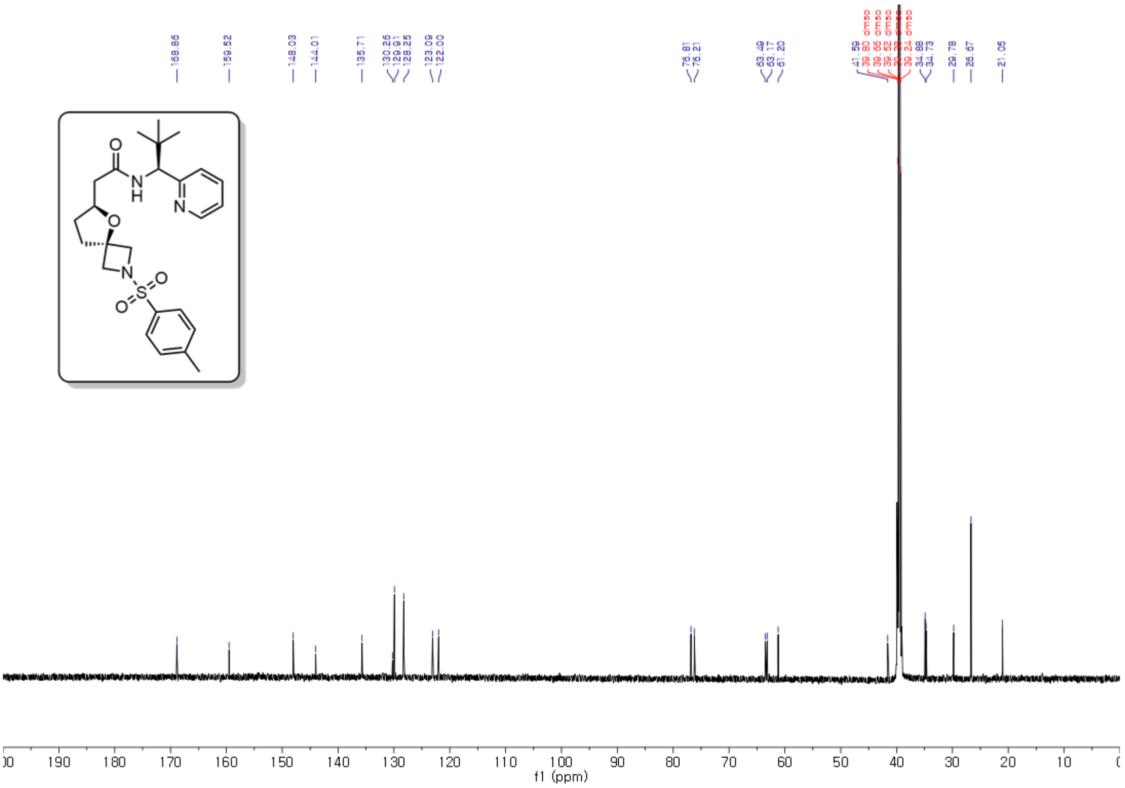

151 MHz, ^{13}C NMR in DMSO-d_6

_N_-((_S_)-2,2-dimethyl-1-(pyridin-2-yl)propyl)-2-((_S_)-1-oxaspiro[4.4]nonan-2-yl)acetamide (2j).

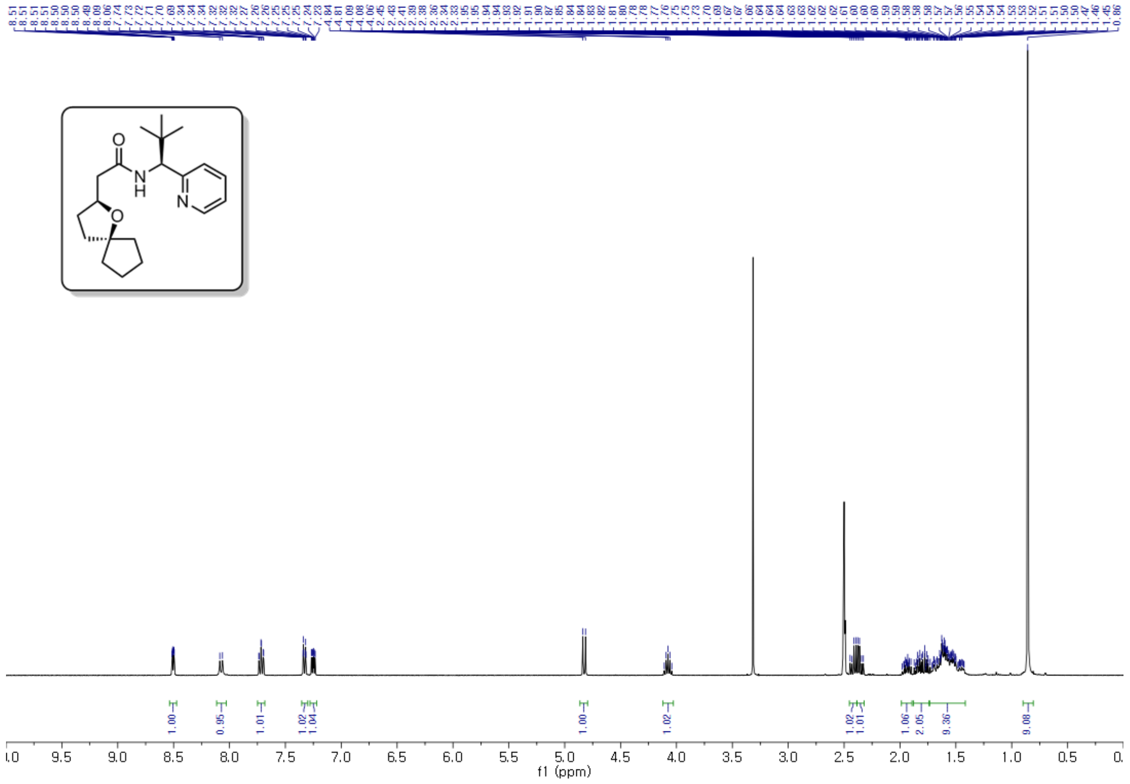

400 MHz, ^{1}H NMR in DMSO-d_6

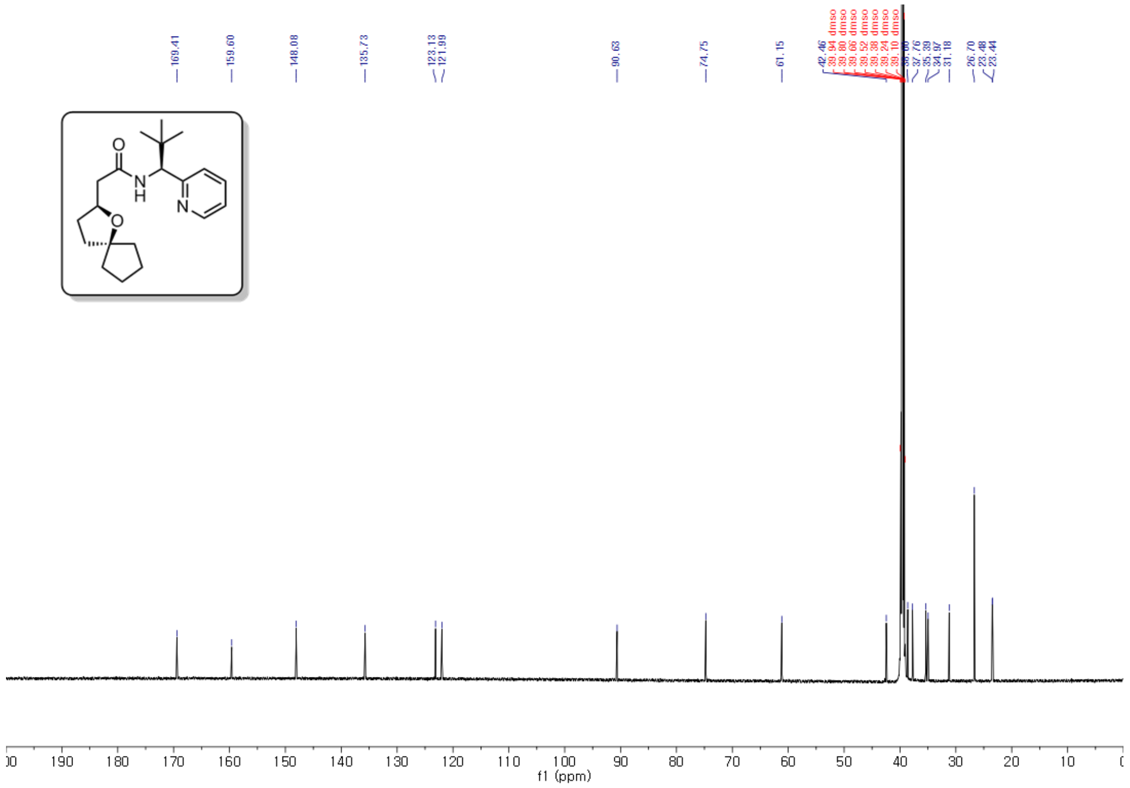

151 MHz, ^{13}C NMR in DMSO-d_6

***N*-((*S*)-2,2-dimethyl-1-(pyridin-2-yl)propyl)-2-((*S*)-1-oxaspiro[4.5]decan-2-yl)acetamide (2k).**

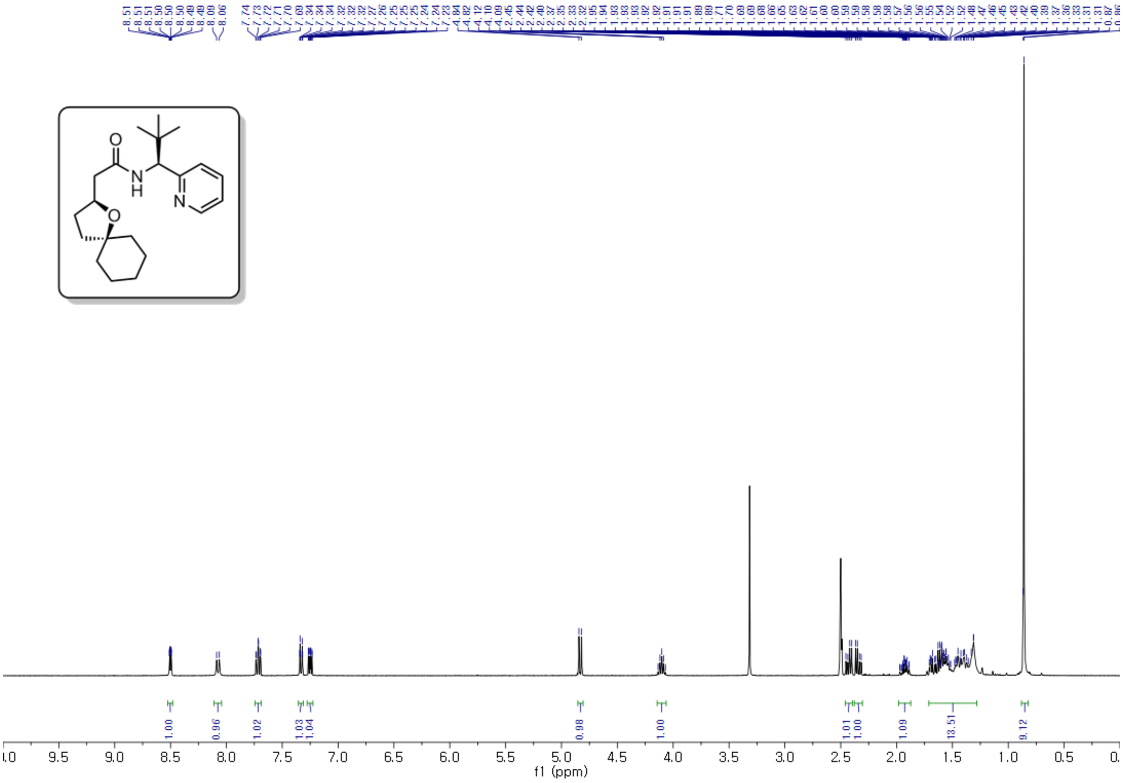

400 MHz, ^{1}H NMR in DMSO-d_6

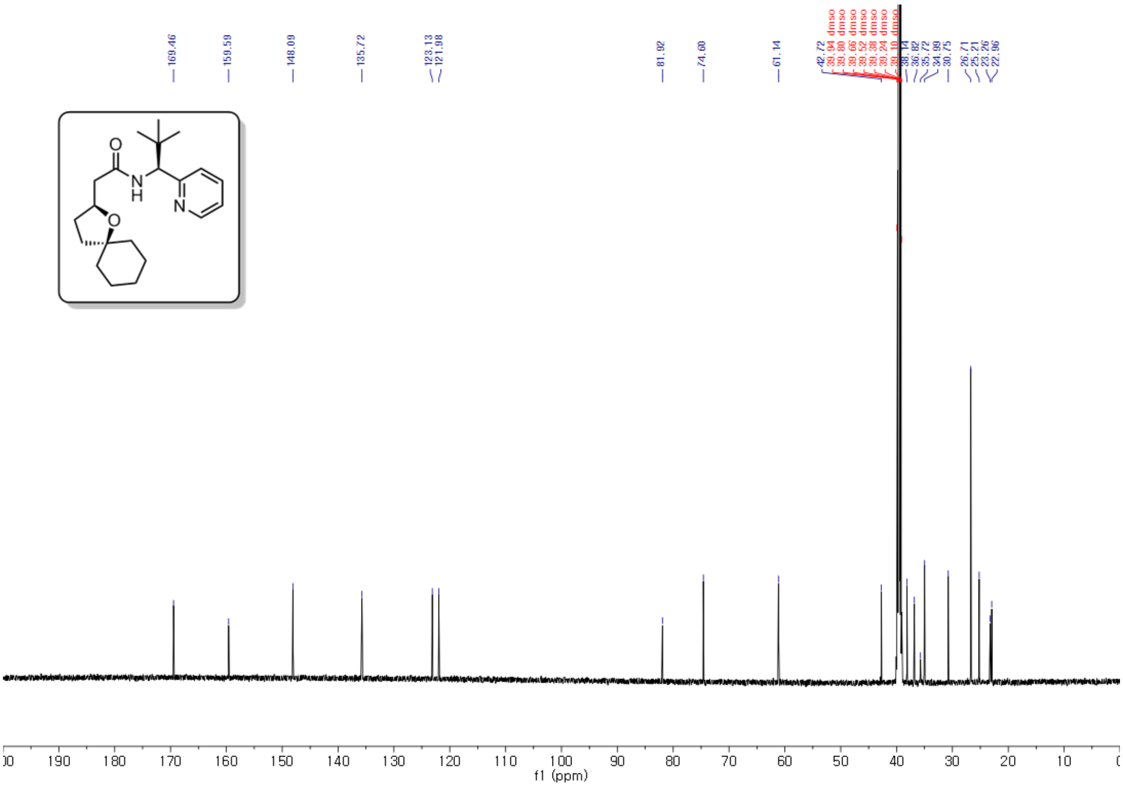

151 MHz, ^{13}C NMR in DMSO-d_6

***N*-((*S*)-2,2-dimethyl-1-(pyridin-2-yl)propyl)-2-((*S*)-8,8-dimethyl-1-oxaspiro[4.5]decan-2-yl)acetamide (2l).**

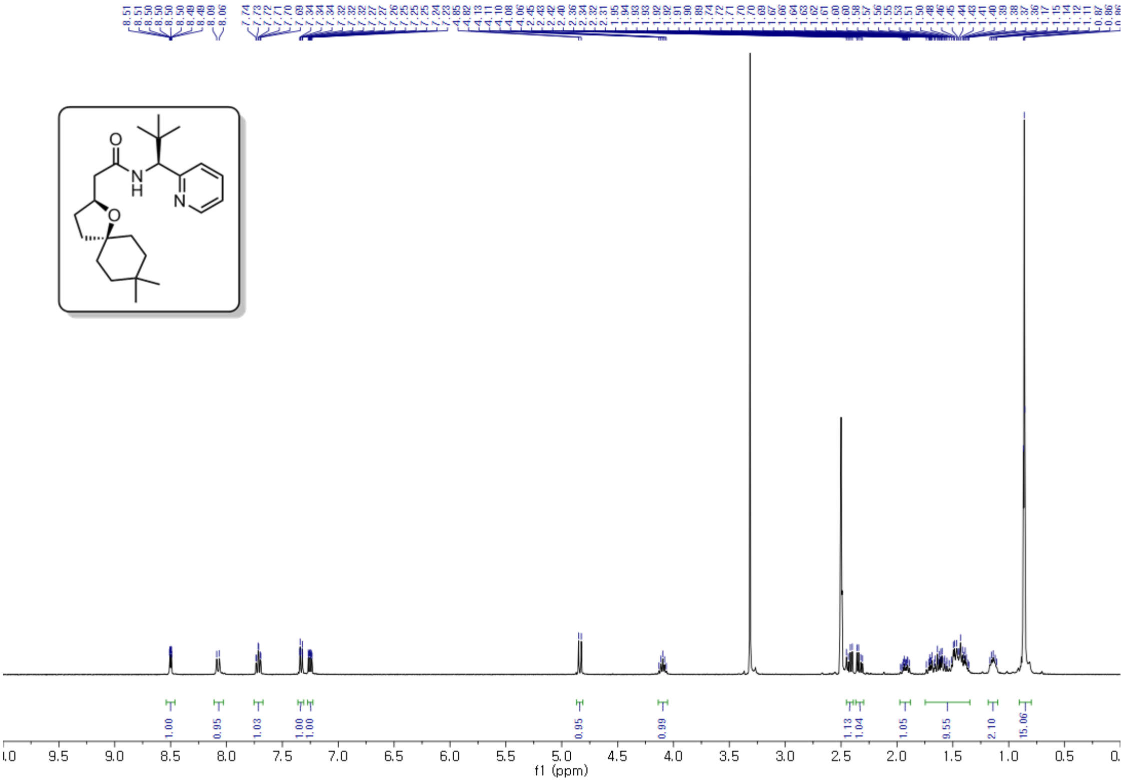

400 MHz, ^{1}H NMR in DMSO-d_6

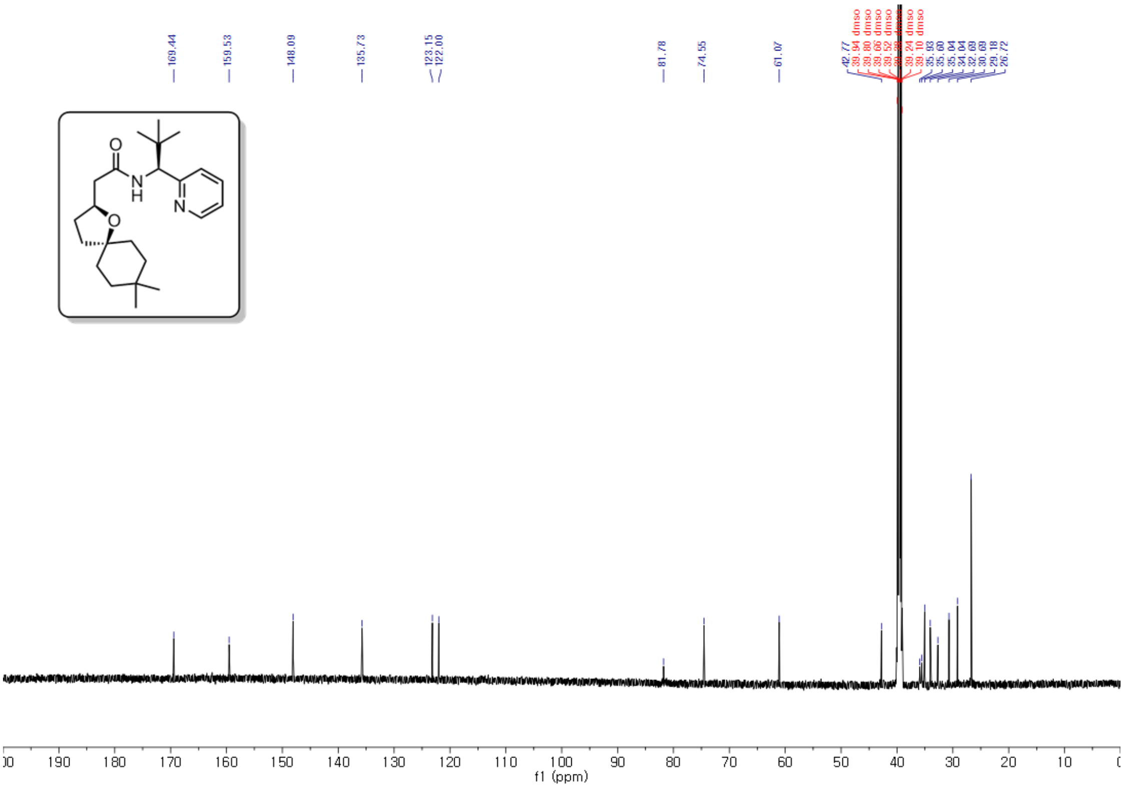

151 MHz, ^{13}C NMR in DMSO-d_6

***N*-((*S*)-2,2-dimethyl-1-(pyridin-2-yl)propyl)-2-((*S*)-7,7,9,9-tetramethyl-1-oxaspiro[4.5]decan-2-yl)acetamide (2m).**

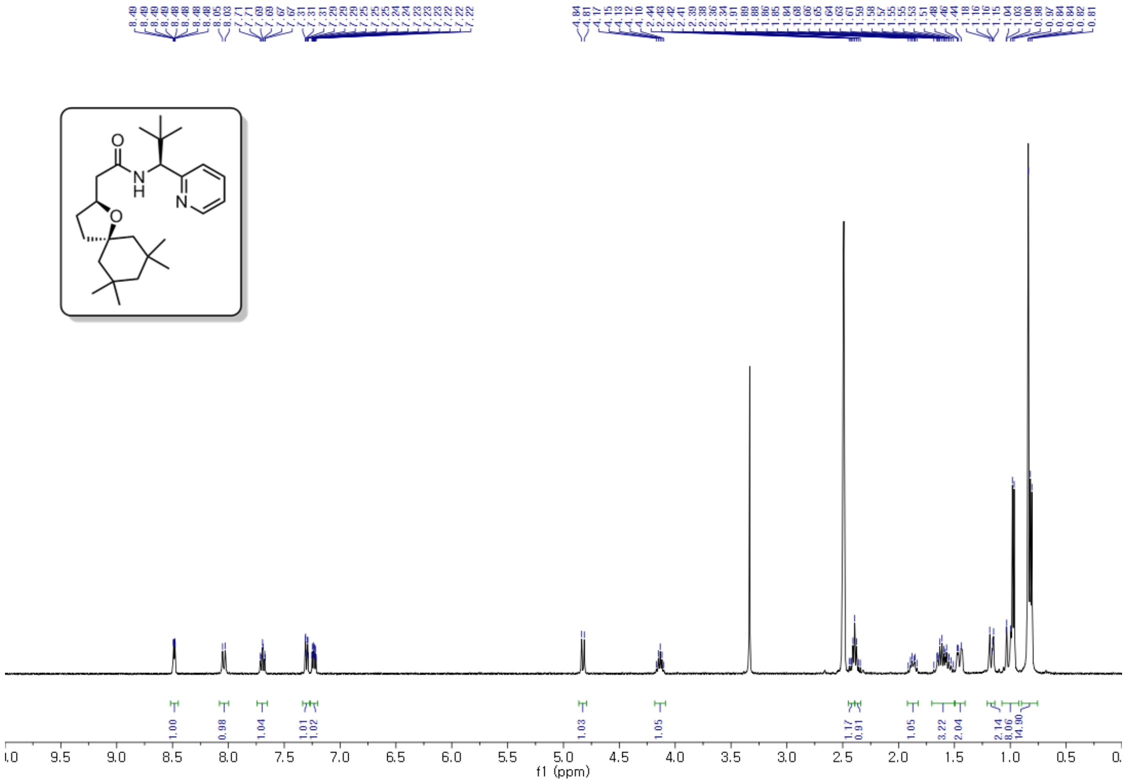

400 MHz, ^{1}H NMR in DMSO-d_6

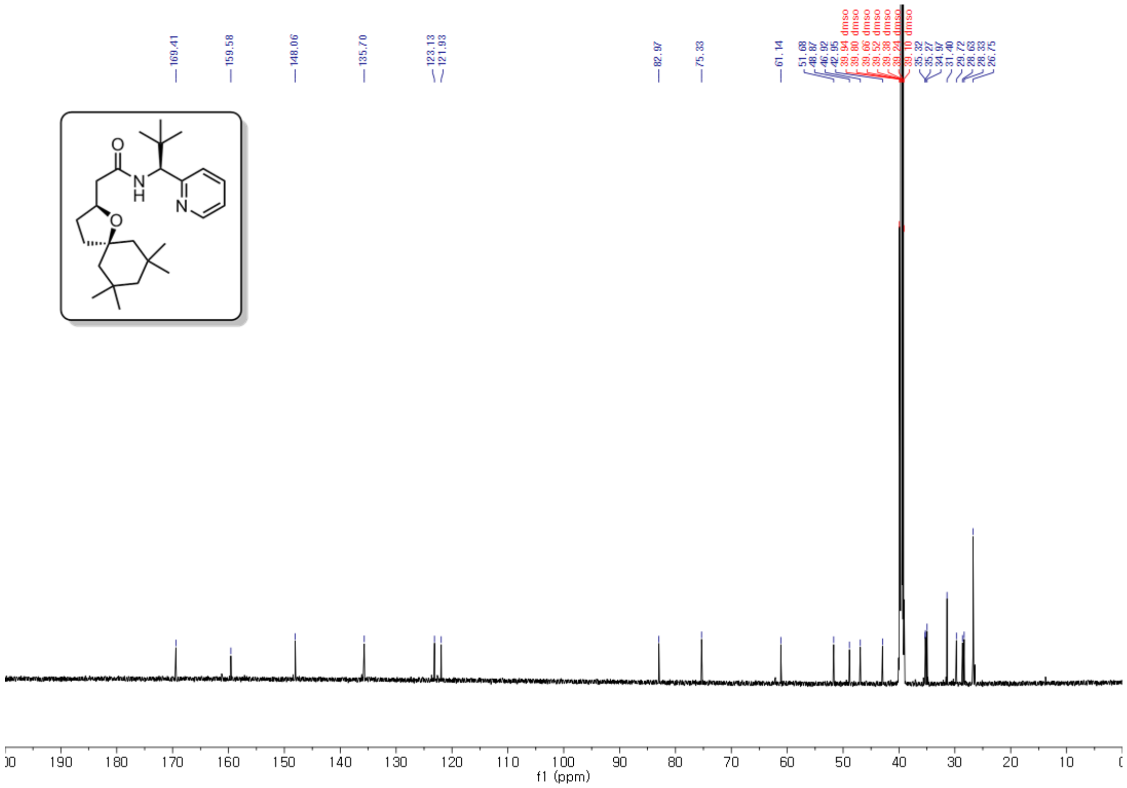

151 MHz, ^{13}C NMR in DMSO-d_6

***N*-((*S*)-2,2-dimethyl-1-(pyridin-2-yl)propyl)-2-((*S*)-1,8-dioxaspiro[4.5]decan-2-yl)acetamide (2n).**

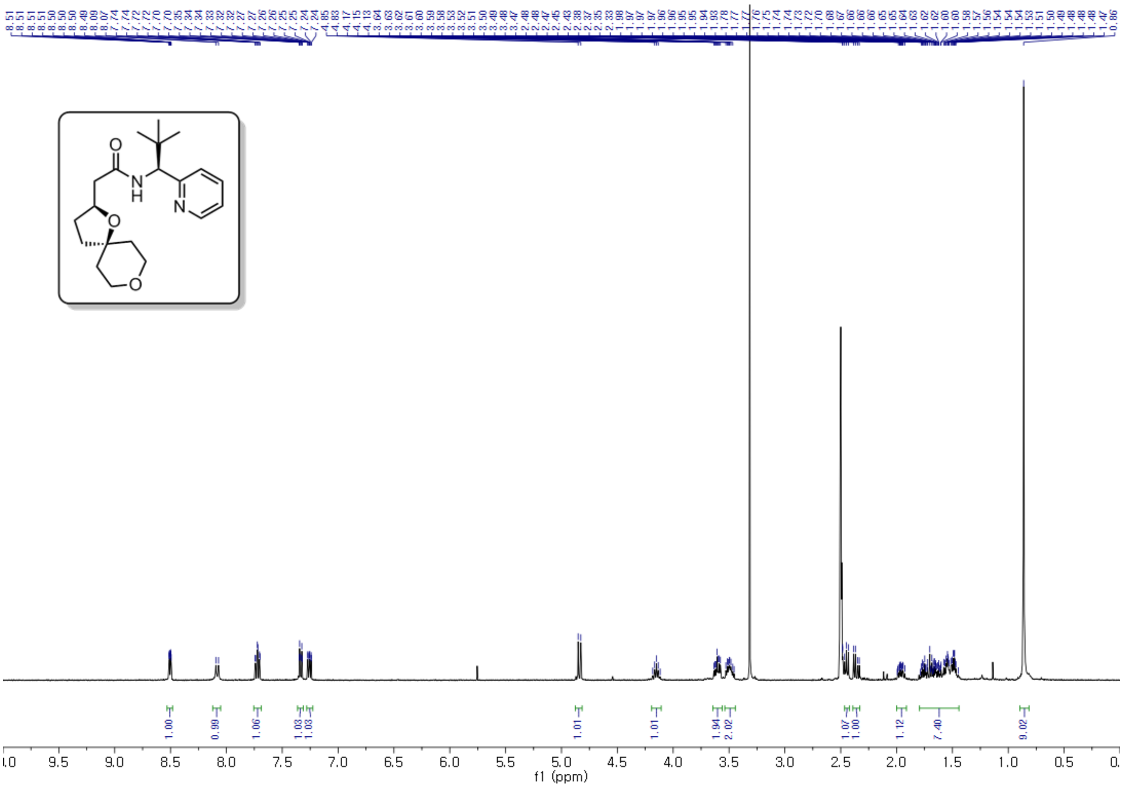

400 MHz, ^{1}H NMR in DMSO-d_6

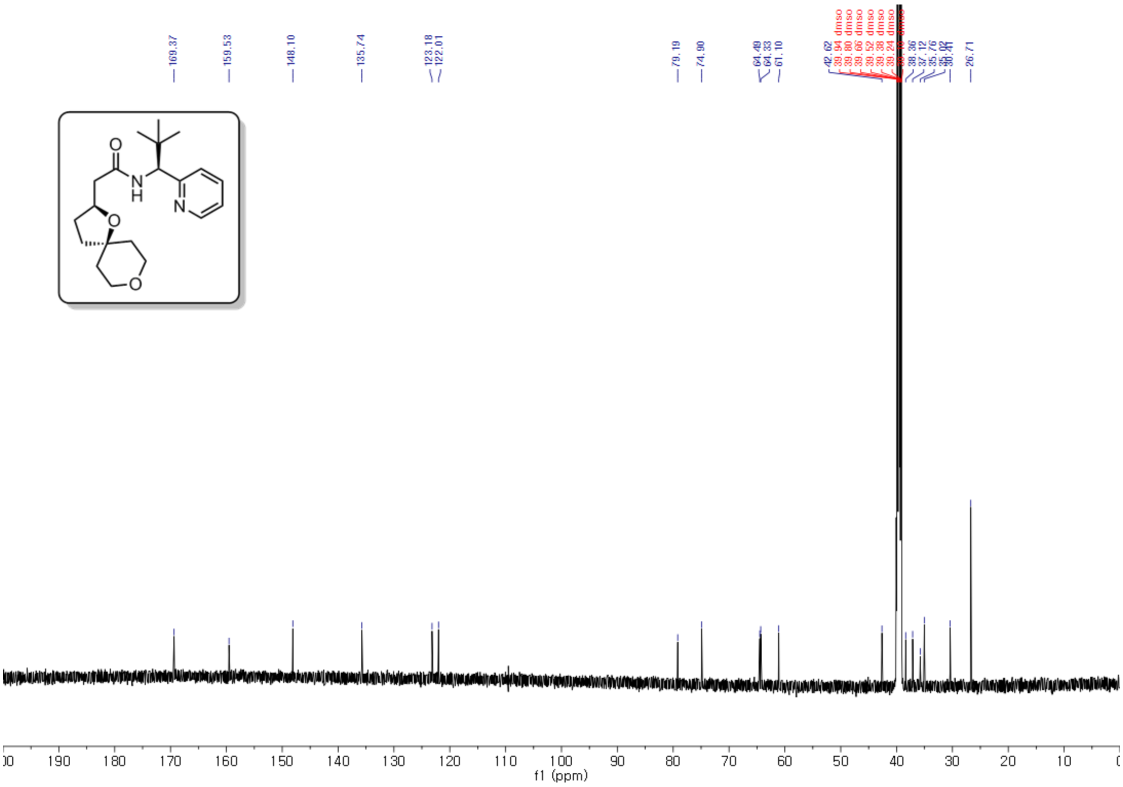

151 MHz, ^{13}C NMR in DMSO-d_6

***tert*-butyl (*S*)-2-(2-(((*S*)-2,2-dimethyl-1-(pyridin-2-yl)propyl)amino)-2-oxoethyl)-1-oxa-8-azaspiro[4.5]decane-8-carboxylate (2o).**

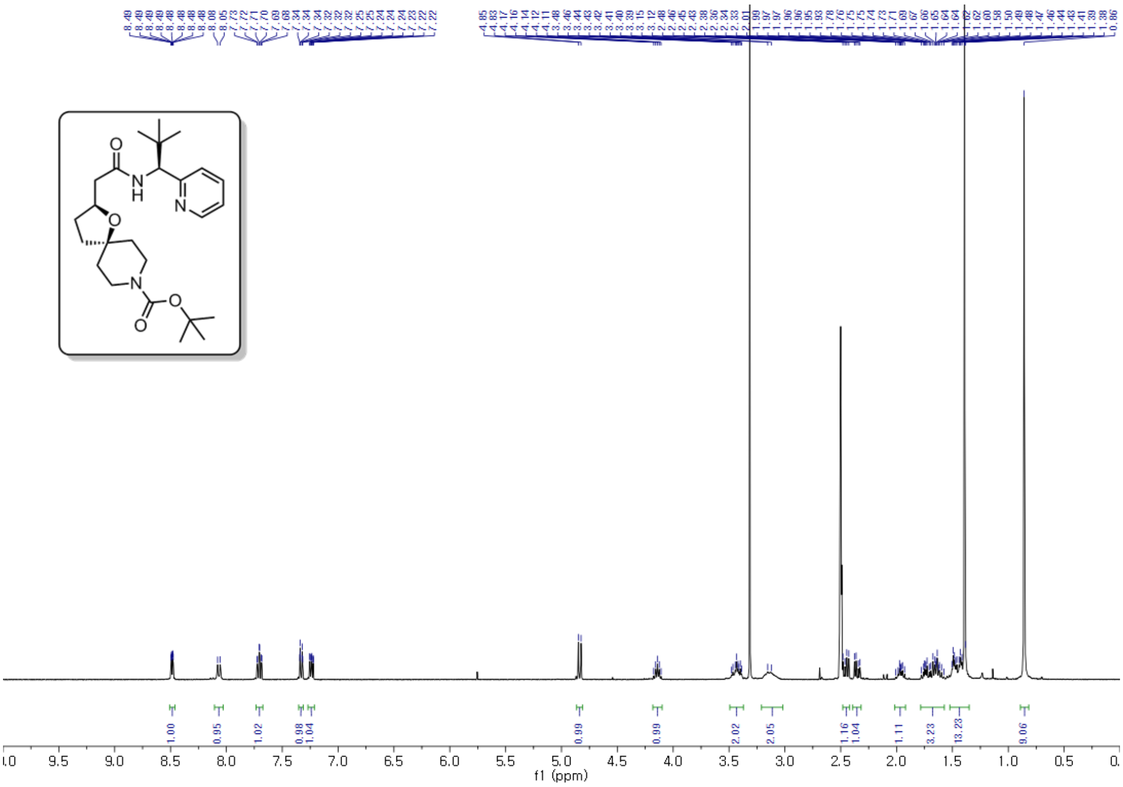

400 MHz, ^{1}H NMR in DMSO-d_6

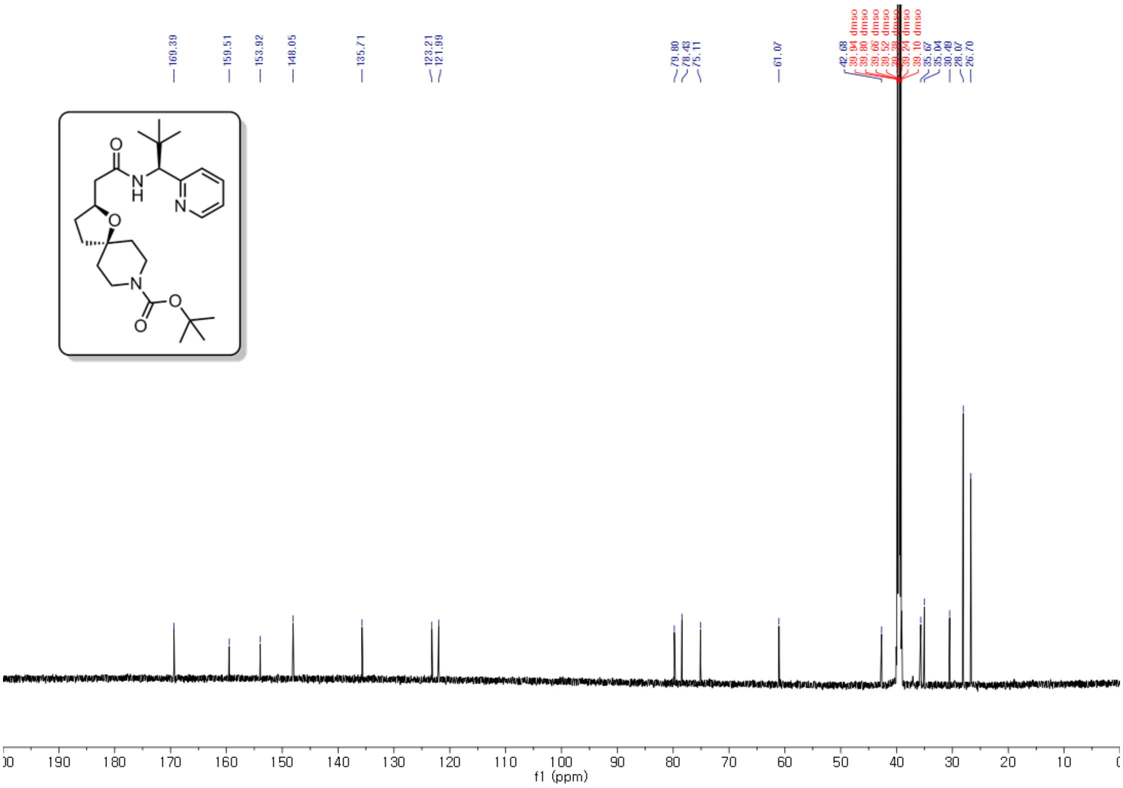

151 MHz, ^{13}C NMR in DMSO-d_6

2-((*S*)-8-acetyl-1-oxa-8-azaspiro[4.5]decan-2-yl)-*N*-((*S*)-2,2-dimethyl-1-(pyridin-2-yl)propyl)acetamide (2p).

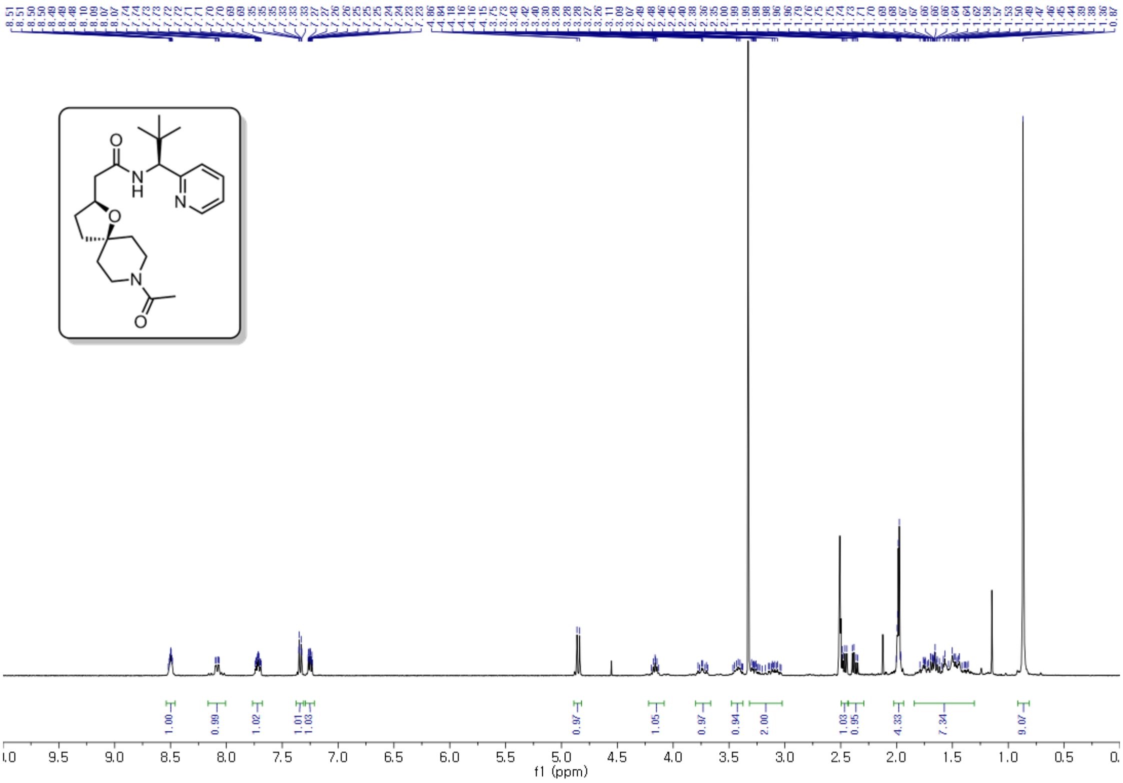

400 MHz, ^{1}H NMR in DMSO-d_6

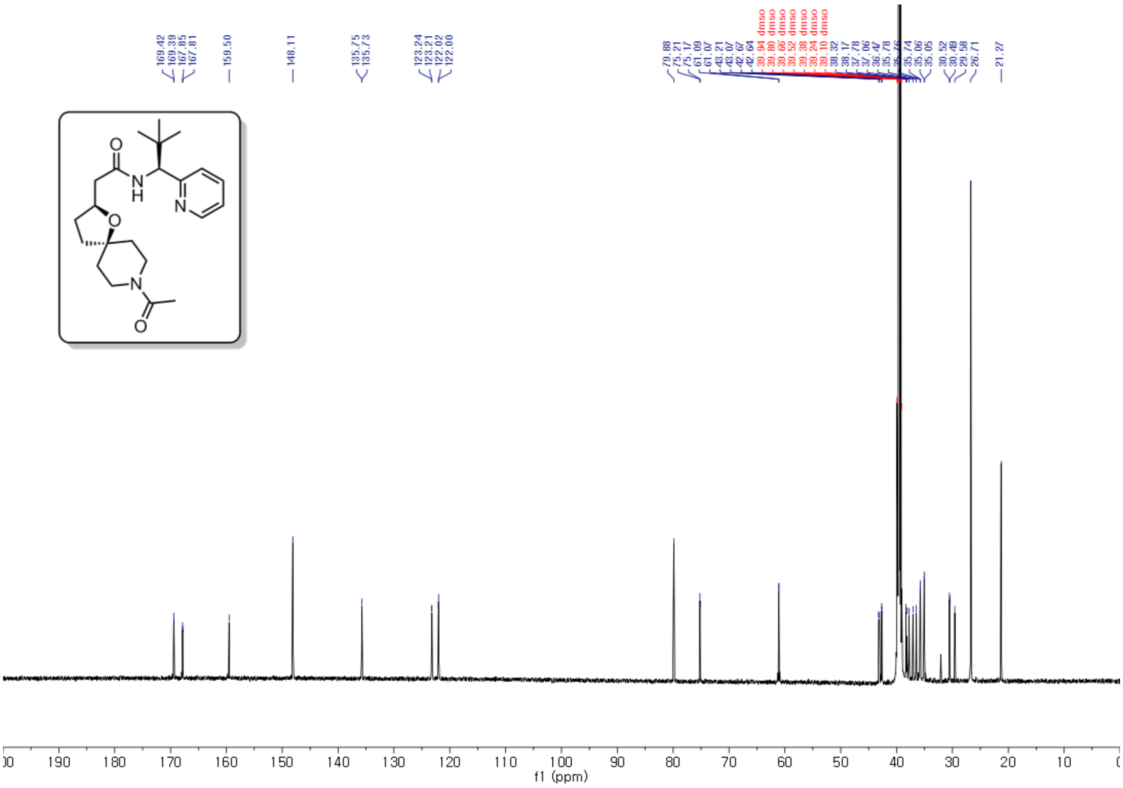

151 MHz, ^{13}C NMR in DMSO-d_6

***N*-((*S*)-2,2-dimethyl-1-(pyridin-2-yl)propyl)-2-((*S*)-1-oxaspiro[4.6]undecan-2-yl)acetamide (2q).**

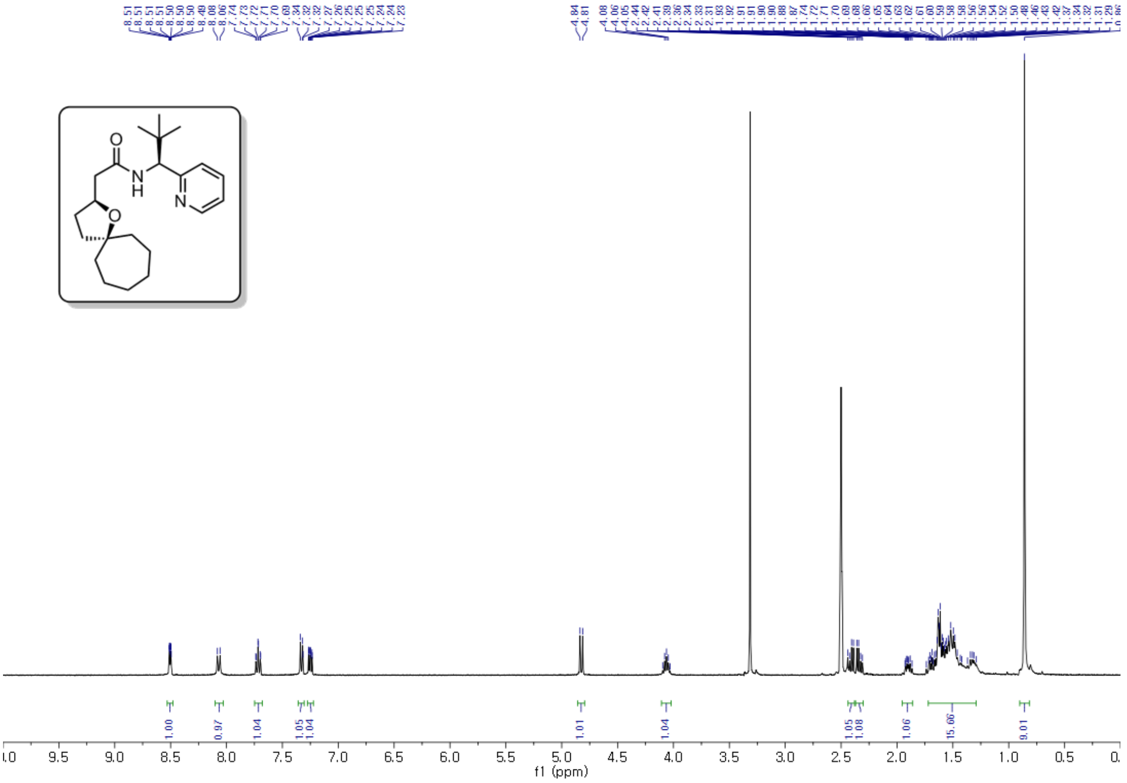

400 MHz, ^{1}H NMR in DMSO-d_6

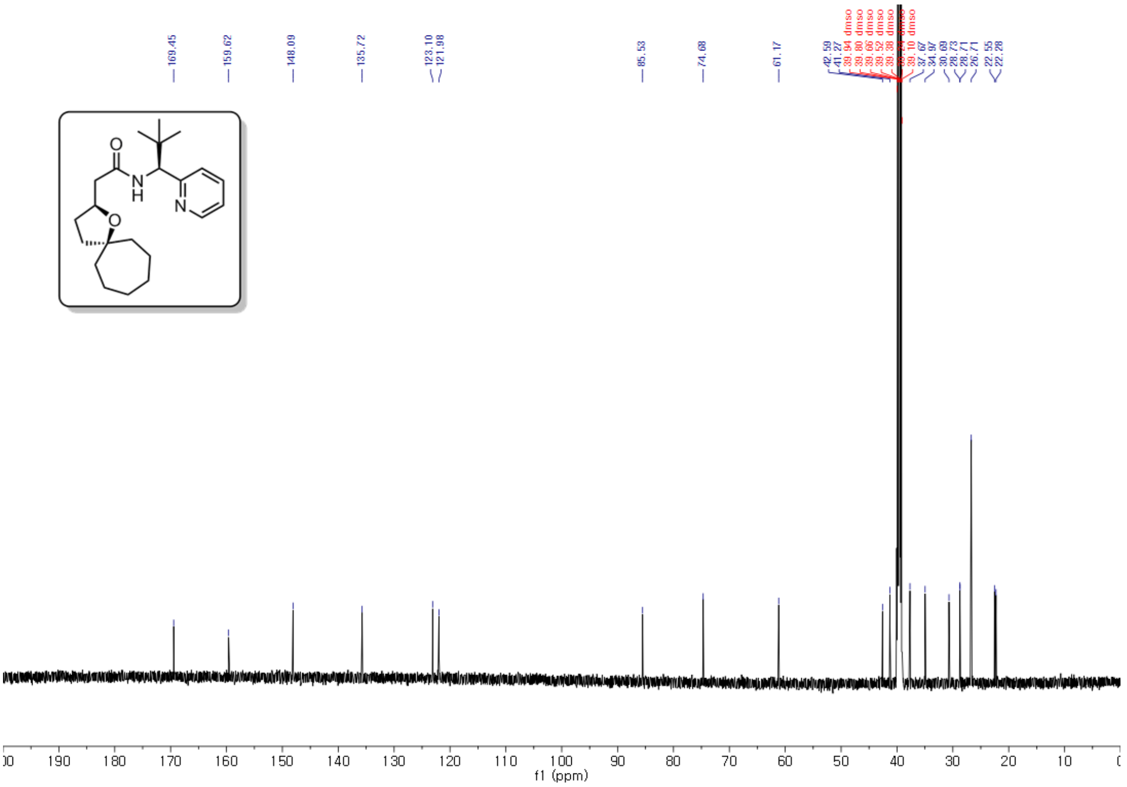

151 MHz, ^{13}C NMR in DMSO-d_6

N-((S)-2,2-dimethyl-1-(pyridin-2-yl)propyl)-2-((S)-1-oxaspiro[4.7]dodecan-2-yl)acetamide (2r).

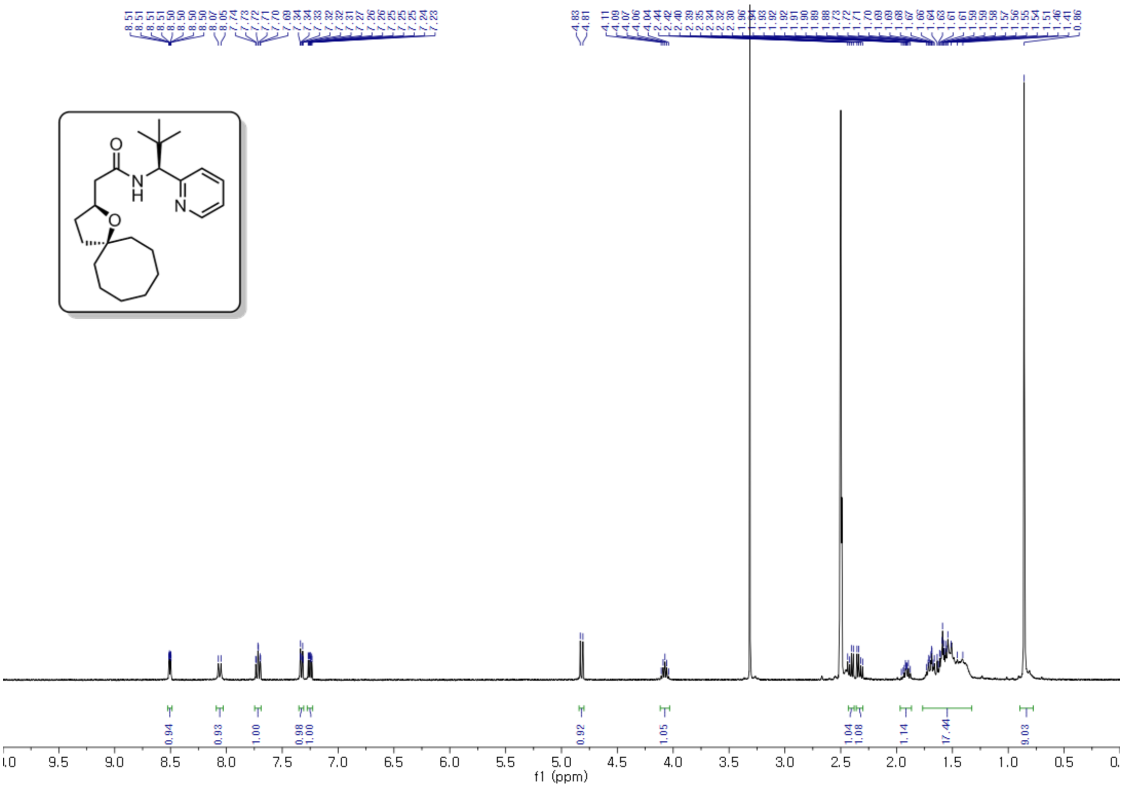

400 MHz, ^{1}H NMR in DMSO-d_6

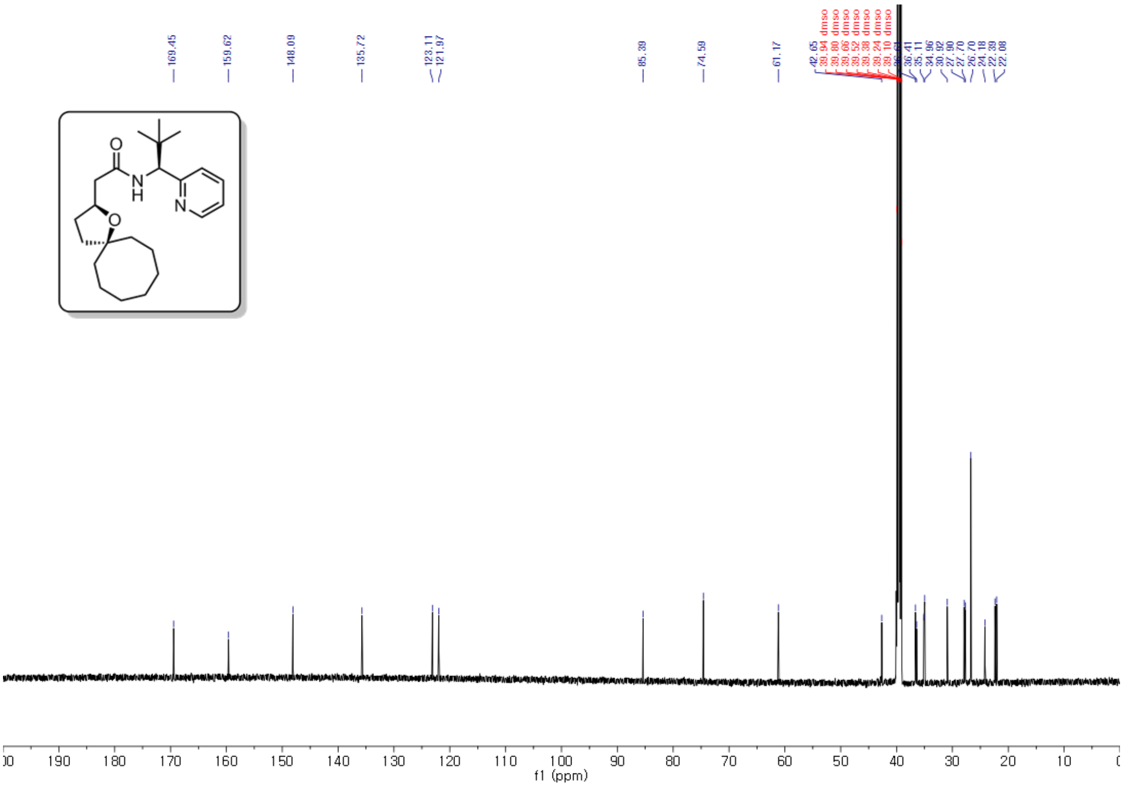

151 MHz, ^{13}C NMR in DMSO-d_6

2-((1*R*,5*S*,5'*S*)-dihydro-3'H-spiro[bicyclo[3.3.1]nonane-9,2'-furan]-5'-yl)-*N*-((*S*)-2,2-dimethyl-1-(pyridin-2-yl)propyl)acetamide (2s).

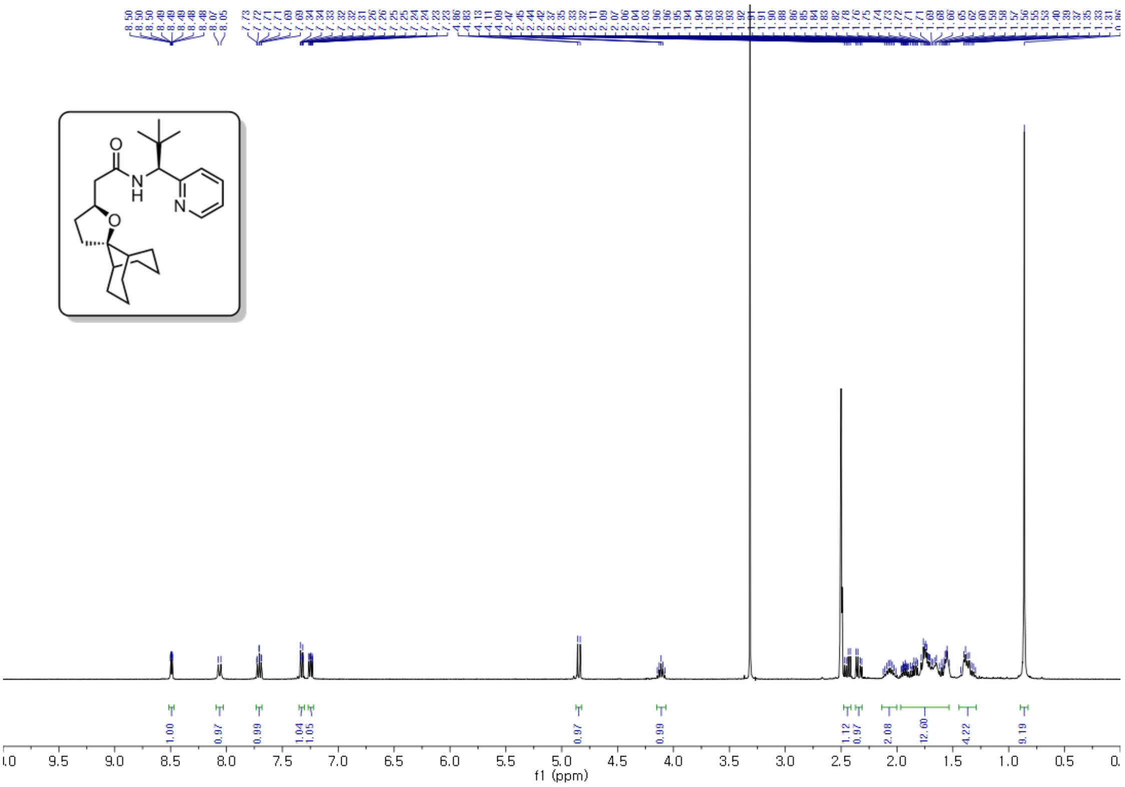

400 MHz, ^{1}H NMR in DMSO-d_6

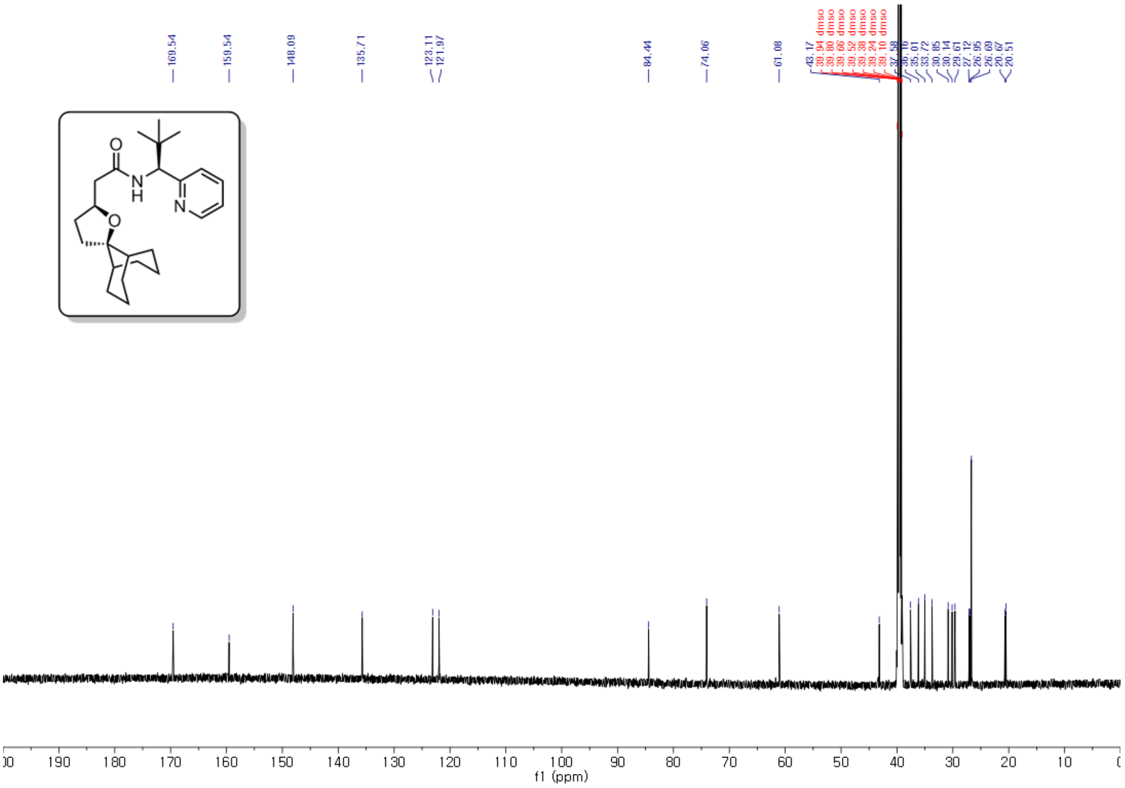

151 MHz, ^{13}C NMR in DMSO-d_6

2-((1S,3S,5S,5'S,7S)-dihydro-3'H-spiro[adamantane-2,2'-furan]-5'-yl)-N-((S)-2,2-dimethyl-1-(pyridin-2-yl)propyl)acetamide (2t).

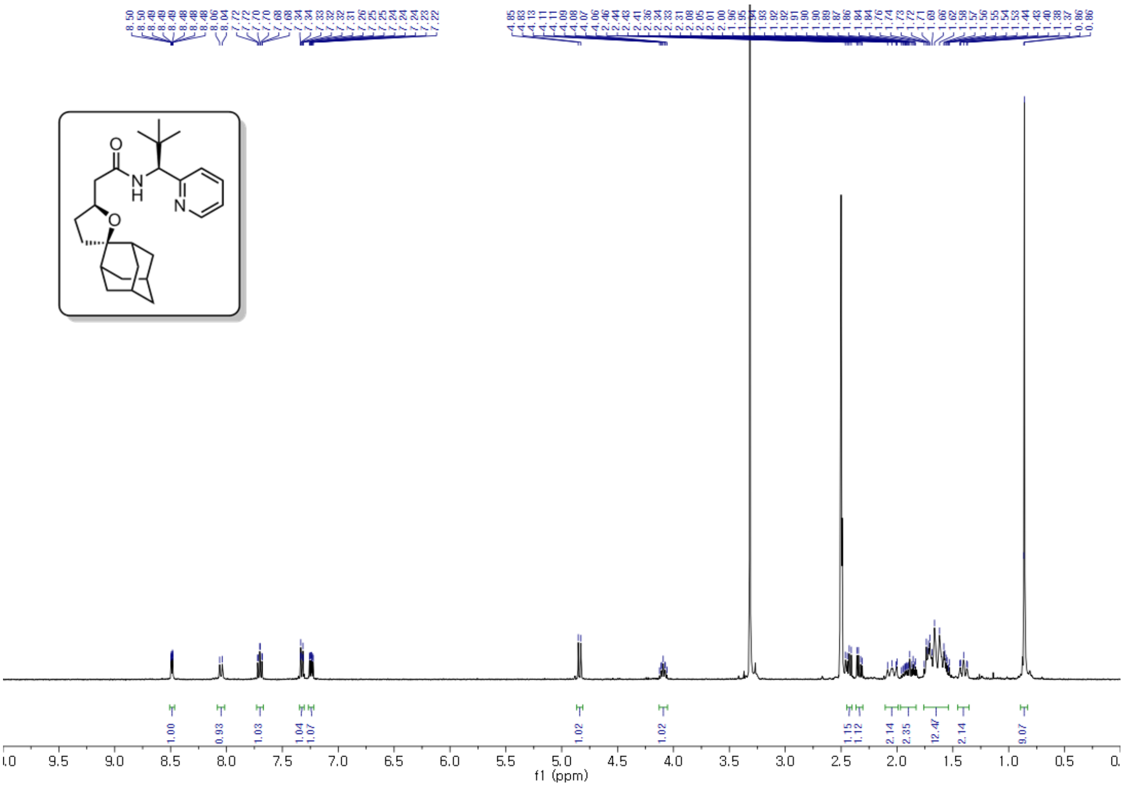

400 MHz, ^{1}H NMR in DMSO-d_6

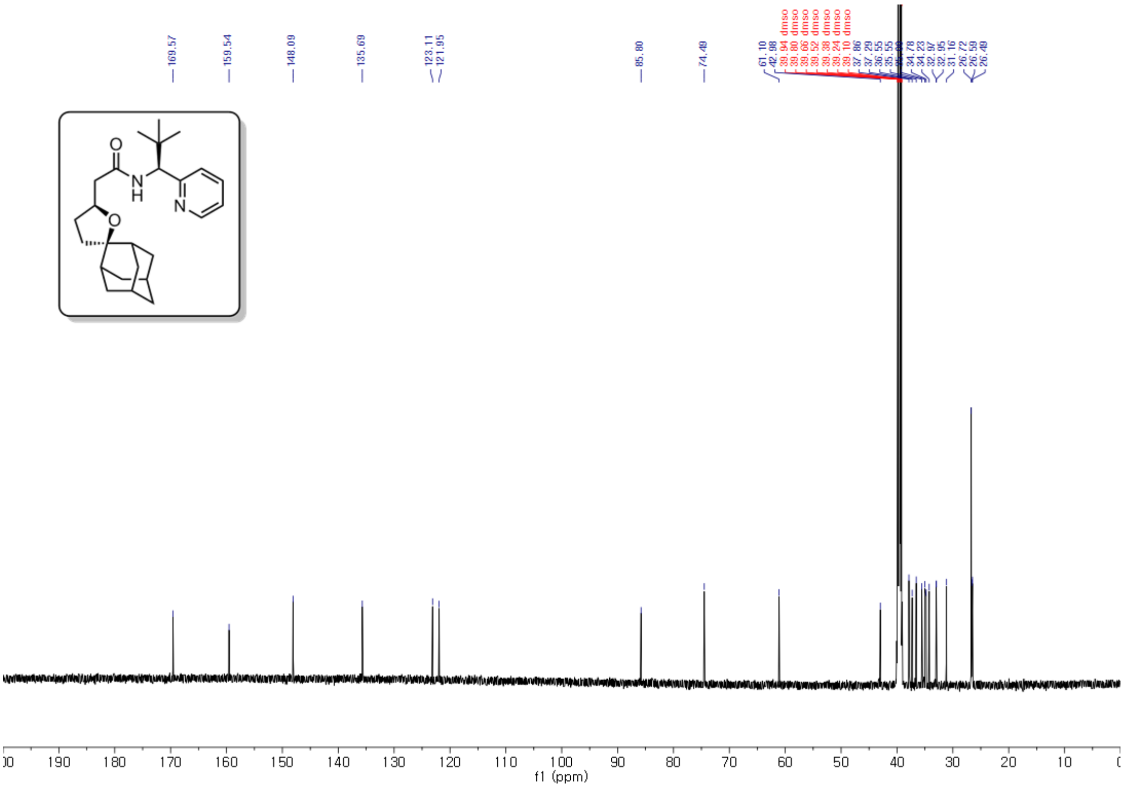

151 MHz, ^{13}C NMR in DMSO-d_6

N-((S)-2,2-dimethyl-1-(pyridin-2-yl)propyl)-2-((S)-1-oxadispiro[4.2.5⁸.2⁵]pentadecan-2-yl)acetamide (2u).

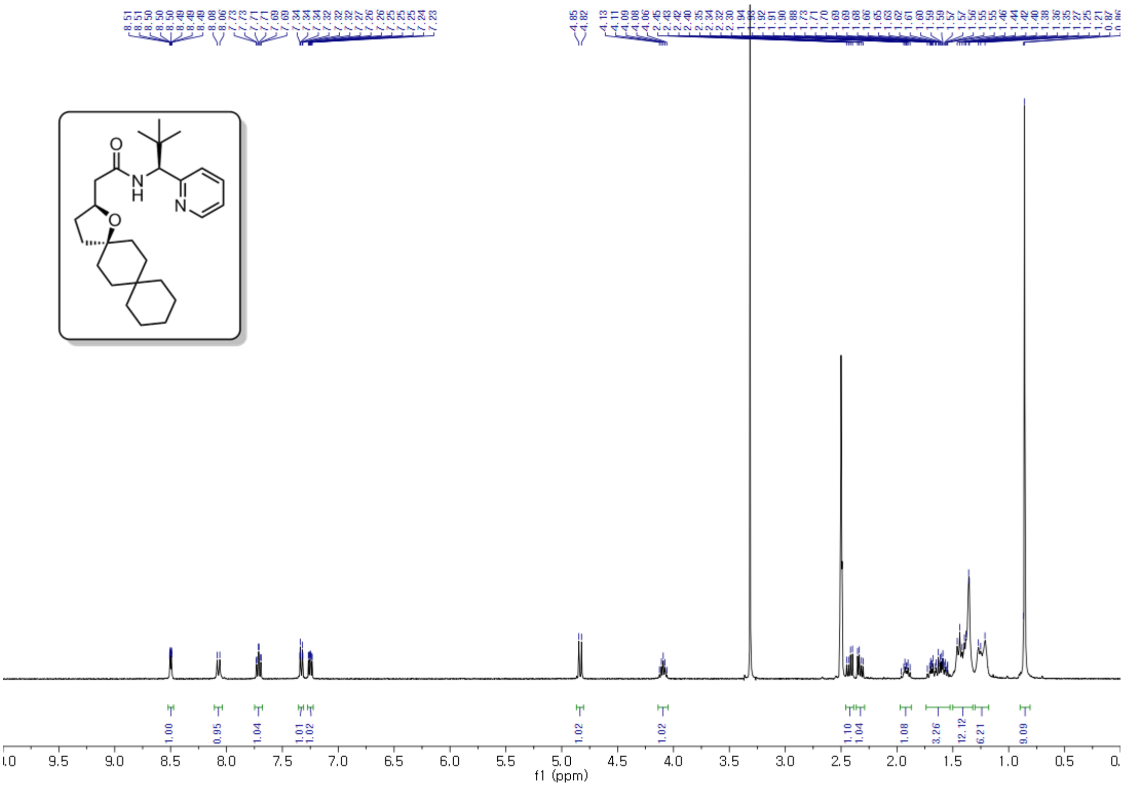

400 MHz, ^{1}H NMR in DMSO-d_6

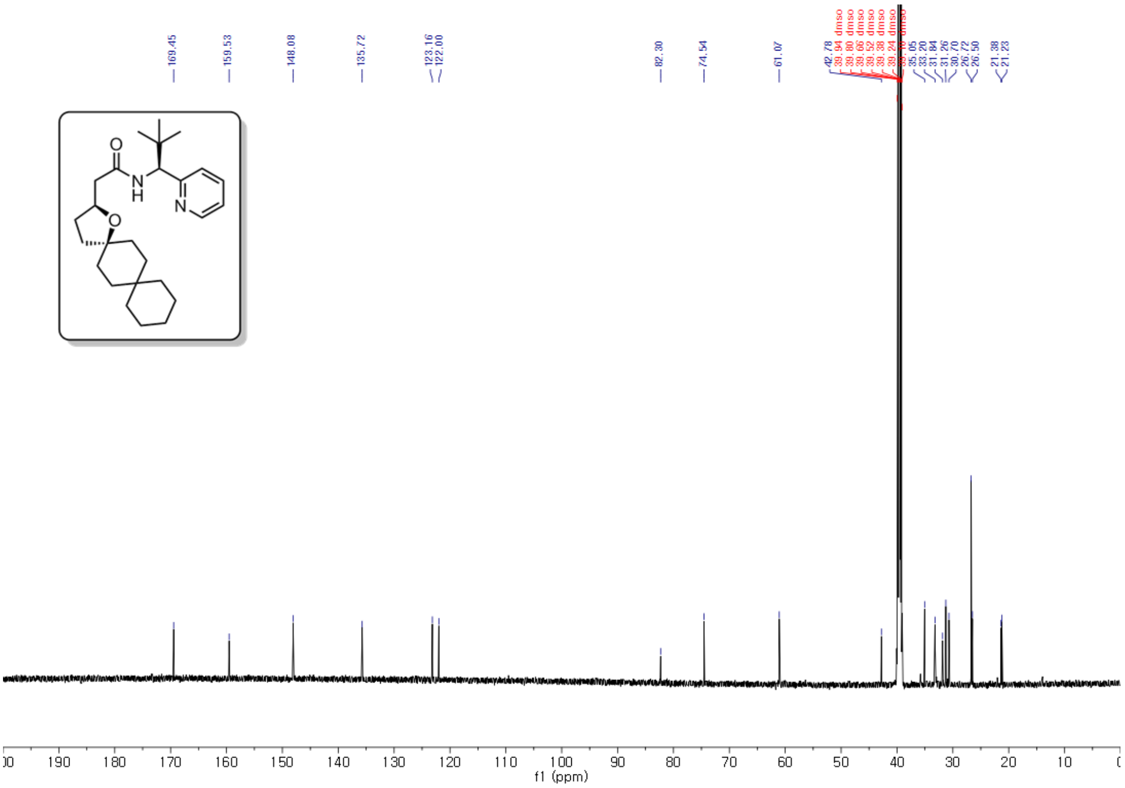

151 MHz, ^{13}C NMR in DMSO-d_6

***tert*-butyl (*S*)-2-(2-(((*S*)-2,2-dimethyl-1-(pyridin-2-yl)propyl)amino)-2-oxoethyl)-1-oxa-11-azadispiro[4.2.5^8.2^5]pentadecane-11-carboxylate (2v)**

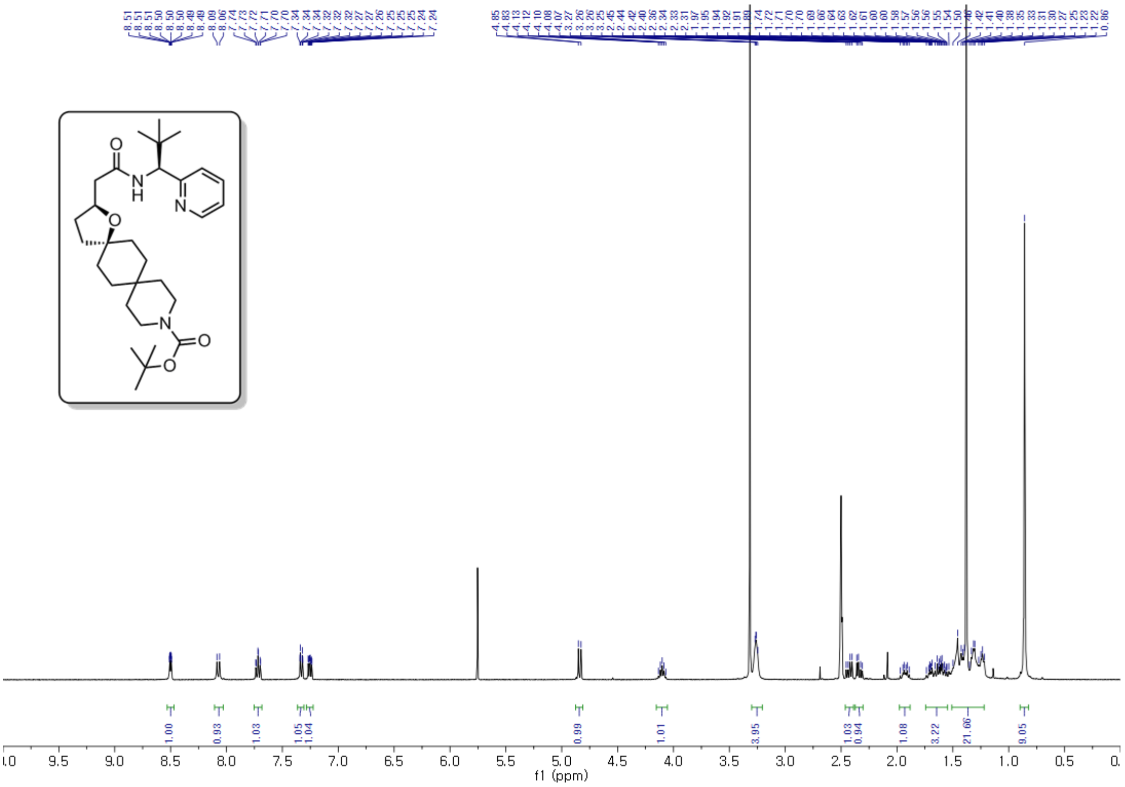

400 MHz, ^{1}H NMR in DMSO-d_6

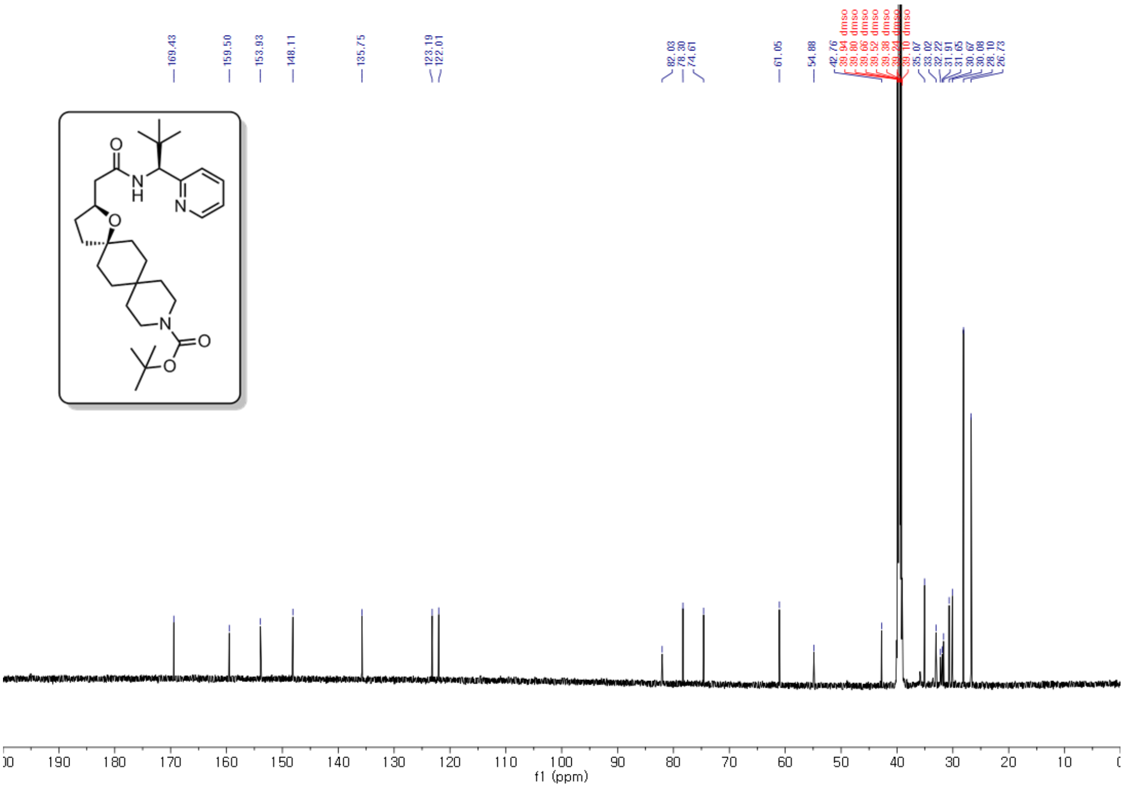

151 MHz, ^{13}C NMR in DMSO-d_6

HPLC Traces

***N*-((*S*)-2,2-dimethyl-1-(pyridin-2-yl)propyl)-2-((*S*)-tetrahydrofuran-2-yl)acetamide (2a).**

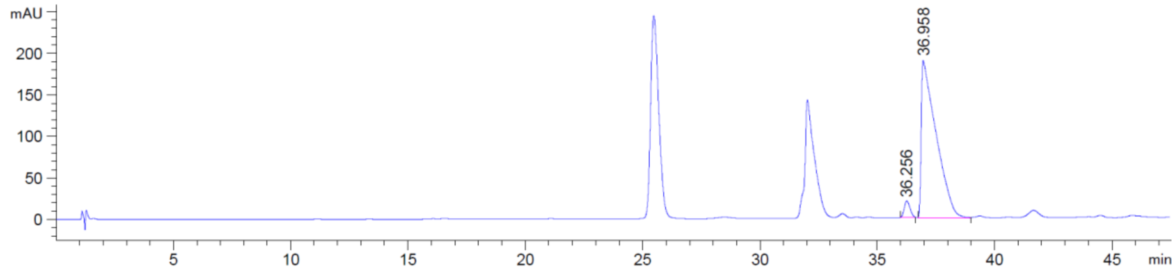

***N*-((*S*)-2,2-dimethyl-1-(pyridin-2-yl)propyl)-2-((*S*)-5-oxaspiro[3.4]octan-6-yl)acetamide (2c).**

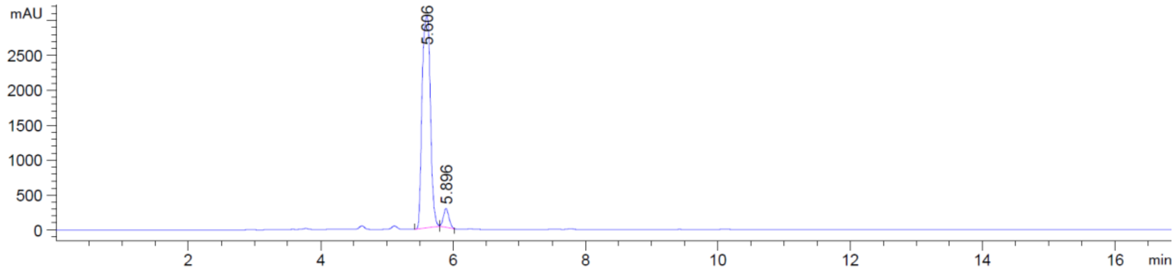

***tert*-butyl (*S*)-6-(2-(((*S*)-2,2-dimethyl-1-(pyridin-2-yl)propyl)amino)-2-oxoethyl)-5-oxa-2-azaspiro[3.4]octane-2-carboxylate (2f).**

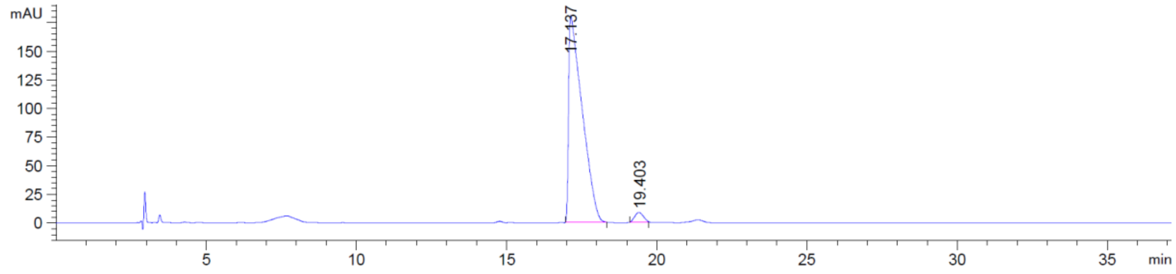

2-((*S*)-2-acetyl-5-oxa-2-azaspiro[3.4]octan-6-yl)-*N*-((S)-2,2-dimethyl-1-(pyridin-2-yl)propyl)acetamide (2g).

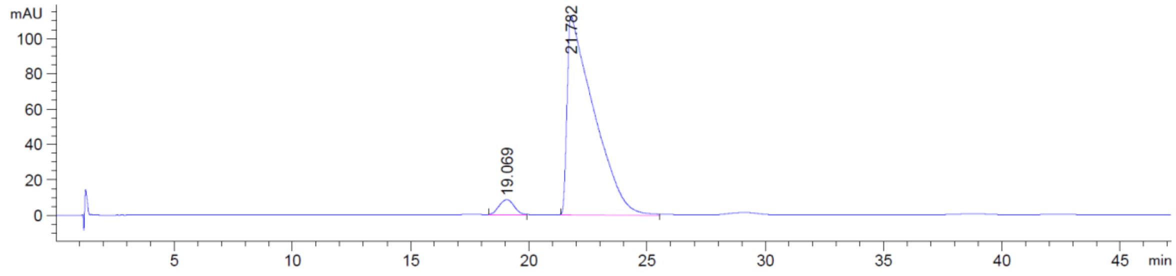

***N*-((*S*)-2,2-dimethyl-1-(pyridin-2-yl)propyl)-2-((*S*)-2-(methylsulfonyl)-5-oxa-2-azaspiro[3.4]octan-6-yl)acetamide (2h).**

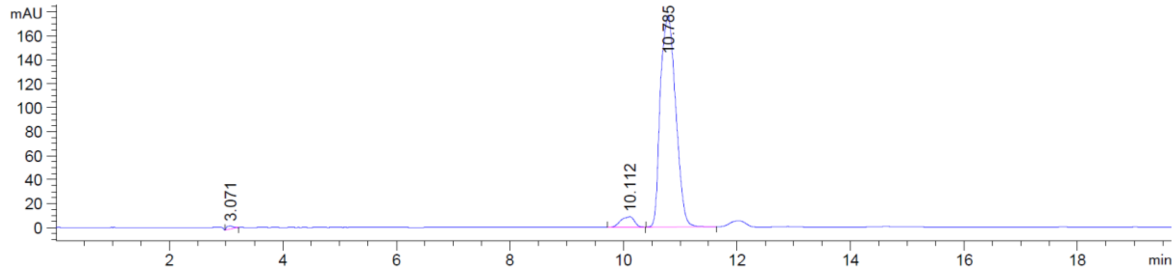

***N*-((*S*)-2,2-dimethyl-1-(pyridin-2-yl)propyl)-2-((*S*)-2-tosyl-5-oxa-2-azaspiro[3.4]octan-6-yl)acetamide (2i).**

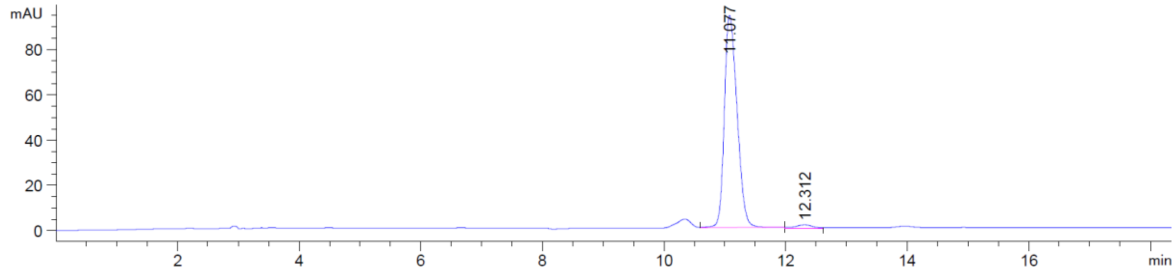

***N*-((*S*)-2,2-dimethyl-1-(pyridin-2-yl)propyl)-2-((*S*)-1-oxaspiro[4.4]nonan-2-yl)acetamide (2j).**

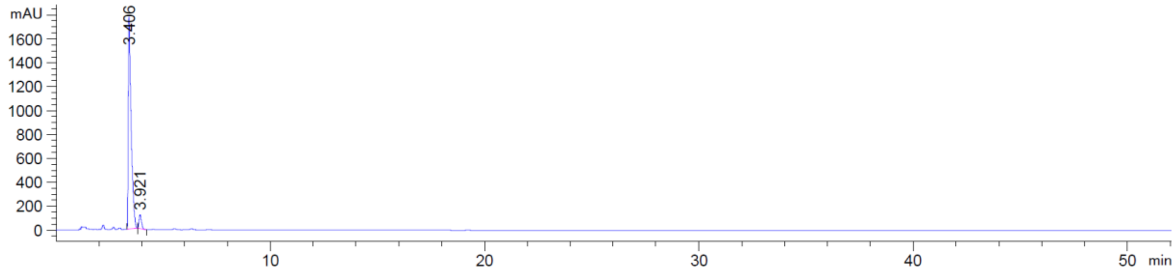

***N*-((*S*)-2,2-dimethyl-1-(pyridin-2-yl)propyl)-2-((*S*)-1-oxaspiro[4.5]decan-2-yl)acetamide (2k).**

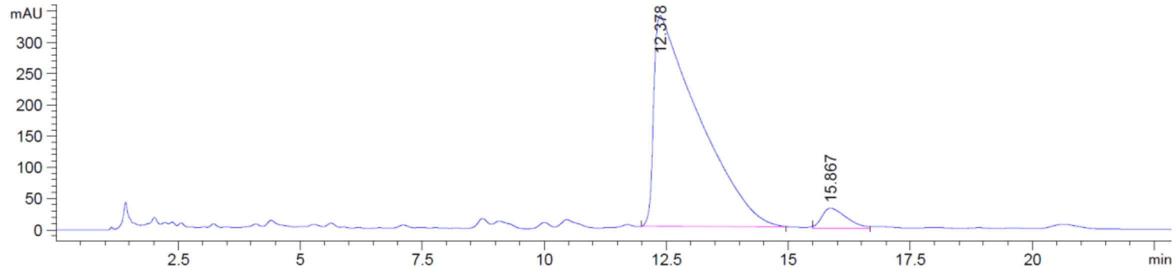

***N*-((*S*)-2,2-dimethyl-1-(pyridin-2-yl)propyl)-2-((*S*)-8,8-dimethyl-1-oxaspiro[4.5]decan-2-yl)acetamide (2l).**

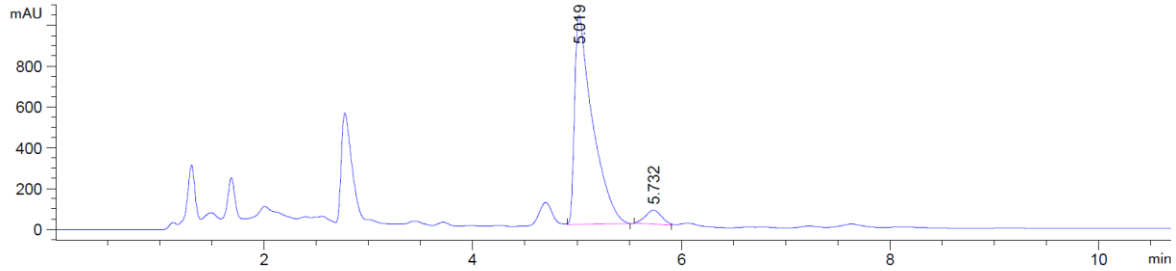

***N*-((*S*)-2,2-dimethyl-1-(pyridin-2-yl)propyl)-2-((*S*)-7,7,9,9-tetramethyl-1-oxaspiro[4.5]decan-2-yl)acetamide (2m).**

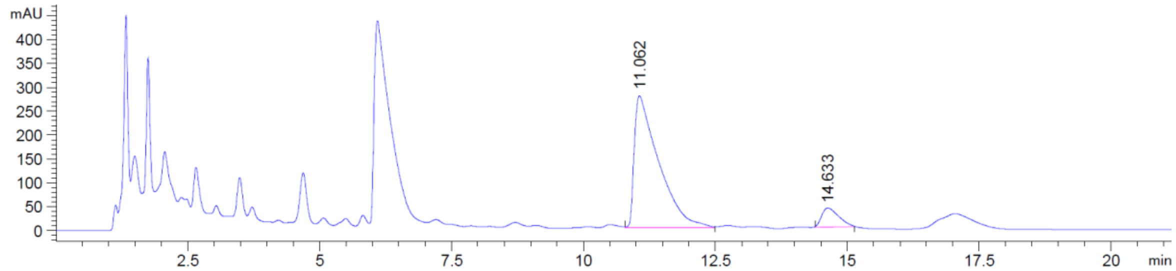

***N*-((*S*)-2,2-dimethyl-1-(pyridin-2-yl)propyl)-2-((*S*)-1,8-dioxaspiro[4.5]decan-2-yl)acetamide (2n).**

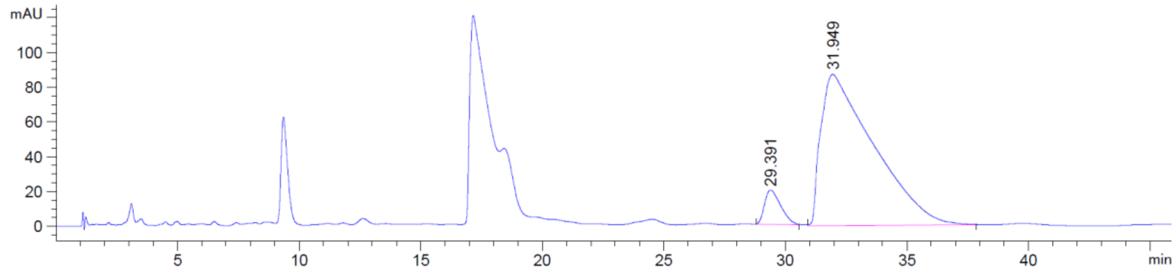

***tert*-butyl (*S*)-2-(2-(((*S*)-2,2-dimethyl-1-(pyridin-2-yl)propyl)amino)-2-oxoethyl)-1-oxa-8-azaspiro[4.5]decane-8-carboxylate (2o).**

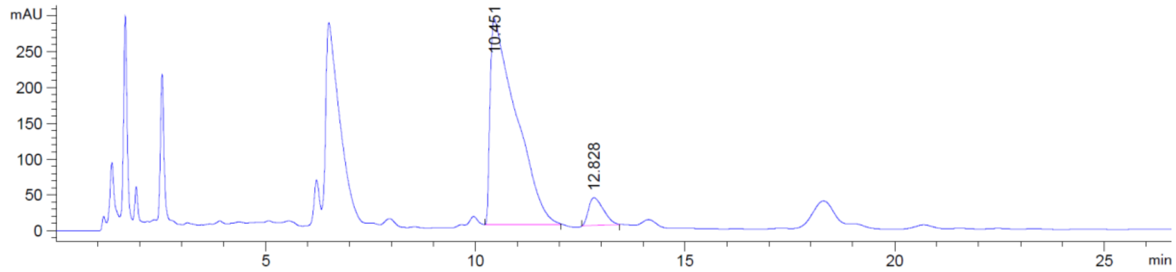

2-((*S*)-8-acetyl-1-oxa-8-azaspiro[4.5]decan-2-yl)-*N*-((*S*)-2,2-dimethyl-1-(pyridin-2-yl)propyl)acetamide (2p).

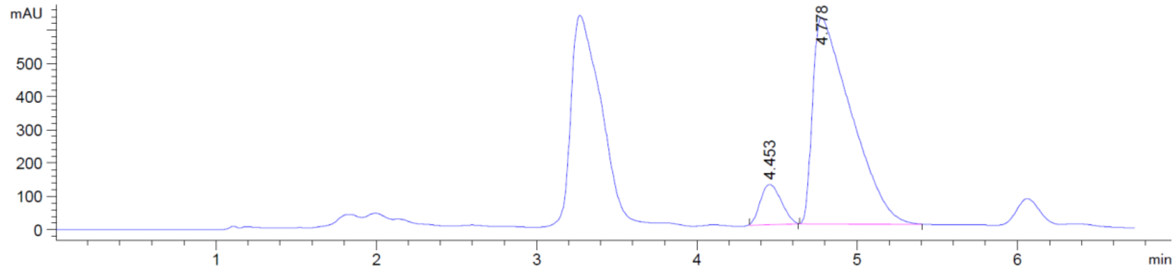

***N*-((*S*)-2,2-dimethyl-1-(pyridin-2-yl)propyl)-2-((*S*)-1-oxaspiro[4.6]undecan-2-yl)acetamide (2q).**

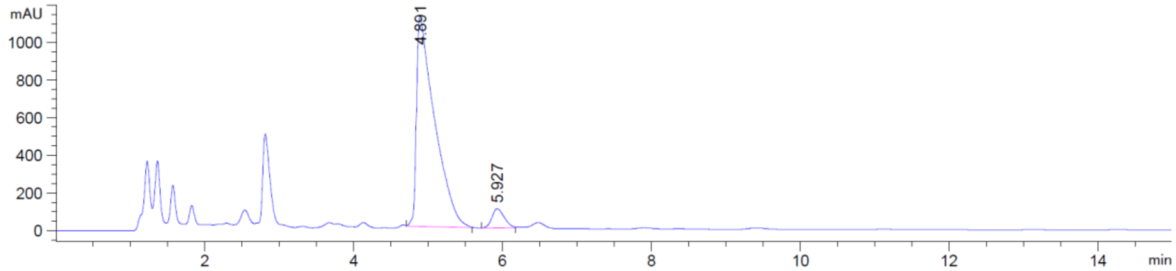

***N*-((*S*)-2,2-dimethyl-1-(pyridin-2-yl)propyl)-2-((*S*)-1-oxaspiro[4.7]dodecan-2-yl)acetamide (2r).**

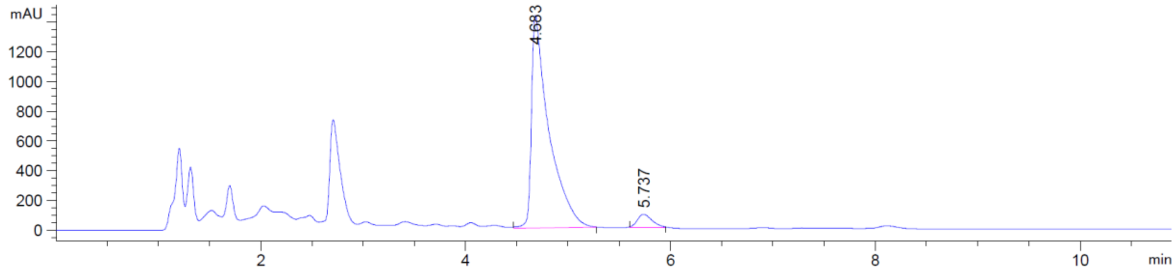

2-((1*R*,5*S*,5'*S*)-dihydro-3'H-spiro[bicyclo[3.3.1]nonane-9,2'-furan]-5'-yl)-*N*-((*S*)-2,2-dimethyl-1-(pyridin-2-yl)propyl)acetamide (2s).

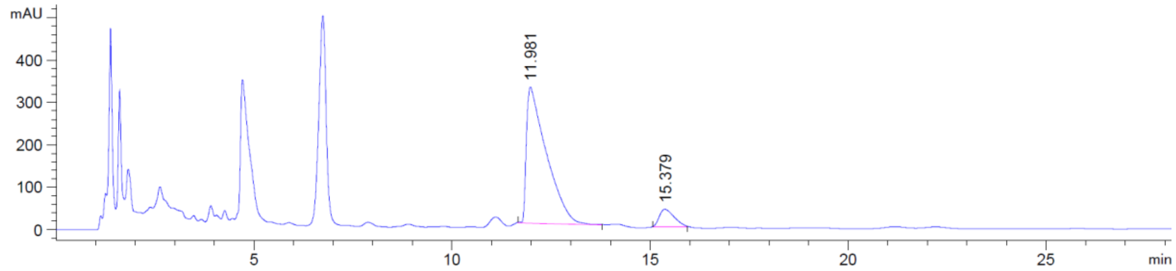

2-((1*S*,3*S*,5*S*,5'*S*,7*S*)-dihydro-3'H-spiro[adamantane-2,2'-furan]-5'-yl)-*N*-((*S*)-2,2-dimethyl-1-(pyridin-2-yl)propyl)acetamide (2t).

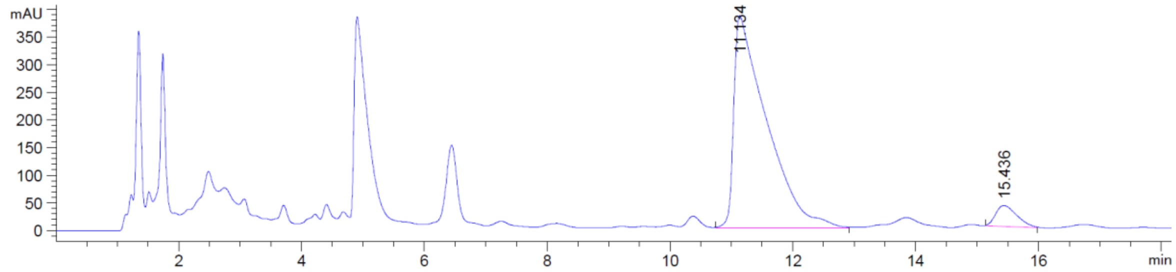

***N*-((*S*)-2,2-dimethyl-1-(pyridin-2-yl)propyl)-2-((*S*)-1-oxadispiro[4.2.5^8.2^5]pentadecan-2-yl)acetamide (2u).**

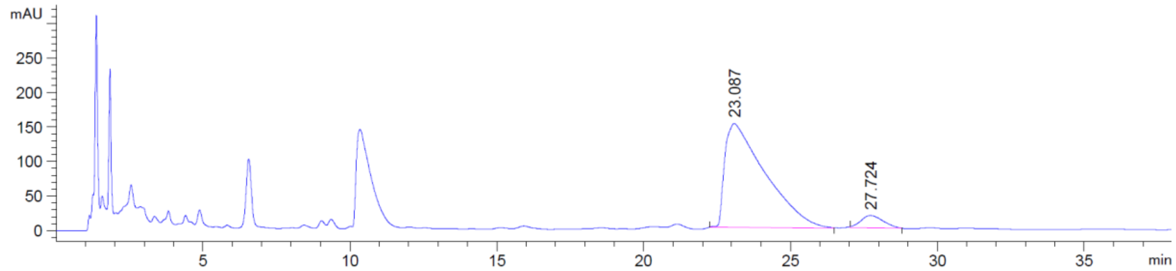

***tert*-butyl (*S*)-2-(2-(((*S*)-2,2-dimethyl-1-(pyridin-2-yl)propyl)amino)-2-oxoethyl)-1-oxa-11-azadispiro[4.2.5^8.2^5]pentadecane-11-carboxylate (2v).**

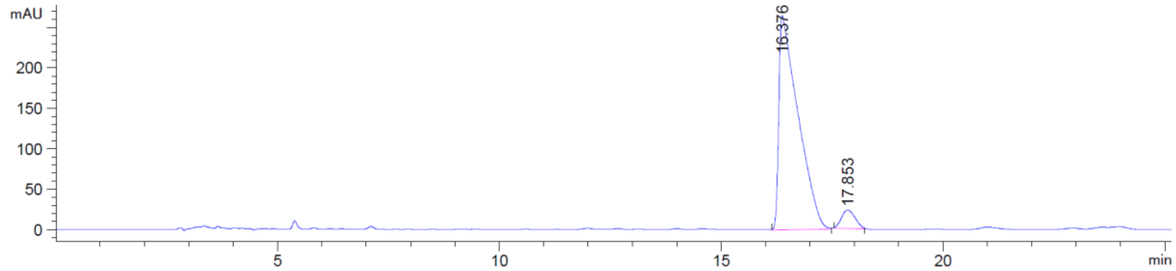

Appendix II

Crystallographic Data for 2a

Crystallographic Data for 2a(nos. CCDC 1581871)

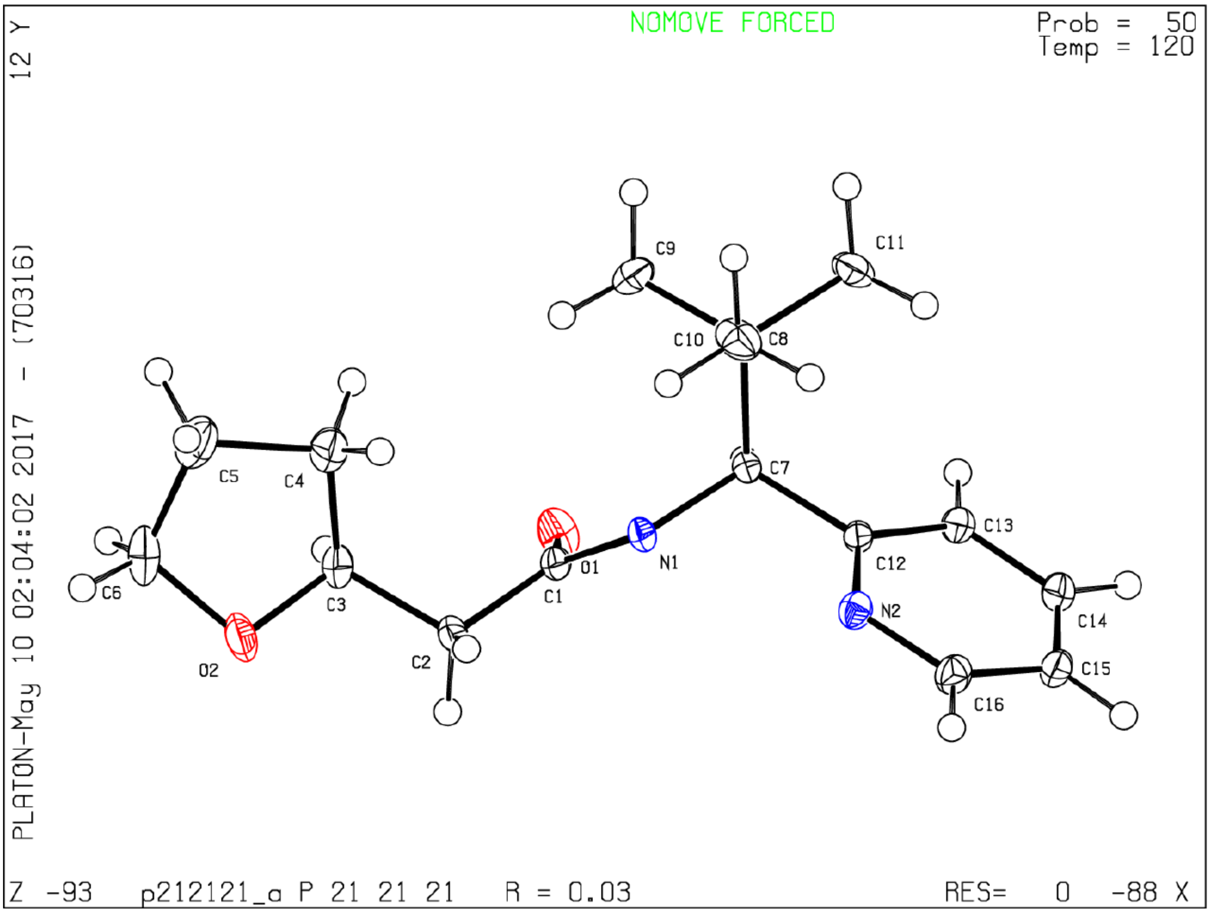

Table S8. Crystal data and structure refinement for **2a**.

Identification code	p212121_a	
Empirical formula	C16 H23 N2 O2	
Formula weight	275.36	
Temperature	120(2) K	
Wavelength	0.71073 Å	
Crystal system	Orthorhombic	
Space group	$P2_12_12_1$	
Unit cell dimensions	a = 6.8237(4) Å	α= 90°.
	b = 9.6779(6) Å	β= 90°.
	c = 23.2090(13) Å	γ = 90°.
Volume	1532.70(16) Å^3	
Z	4	

Density (calculated)	1.193 Mg/m^3
Absorption coefficient	0.079 mm^{-1}
F(000)	596
Crystal size	0.380 x 0.350 x 0.240 mm^3
Theta range for data collection	3.371 to 33.198°.
Index ranges	-10<=h<=10, -14<=k<=14, -35<=l<=35
Reflections collected	90932
Independent reflections	5861 [R(int) = 0.0253]
Completeness to theta = 25.242°	99.7 %
Absorption correction	Semi-empirical from equivalents
Max. and min. transmission	0.7456 and 0.6249
Refinement method	Full-matrix least-squares on F^2
Data / restraints / parameters	5861 / 0 / 184
Goodness-of-fit on F^2	1.084
Final R indices [I>2sigma(I)]	R1 = 0.0338, wR2 = 0.0981
R indices (all data)	R1 = 0.0349, wR2 = 0.0995
Absolute structure parameter	-0.09(9)
Extinction coefficient	n/a
Largest diff. peak and hole	0.607 and -0.252 e.$Å^{-3}$

Table S9. Atomic coordinates (x 10^4) and equivalent isotropic displacement parameters (Å^{2}x 10^3) for **2a**. U(eq) is defined as one third of the trace of the orthogonalized U^{ij} tensor.

	x	y	z	U(eq)
C(1)	4563(1)	5289(1)	2451(1)	14(1)
C(2)	3672(2)	6064(1)	1947(1)	17(1)
C(3)	4796(2)	5746(1)	1399(1)	19(1)
C(4)	6918(2)	6250(2)	1389(1)	31(1)
C(5)	7262(2)	6668(2)	754(1)	31(1)
C(6)	5297(3)	6388(2)	469(1)	31(1)
C(7)	6110(1)	5480(1)	3403(1)	13(1)
C(8)	8369(1)	5749(1)	3421(1)	18(1)
C(9)	9292(2)	5066(2)	2890(1)	33(1)
C(10)	8828(2)	7300(1)	3419(1)	26(1)
C(11)	9254(2)	5076(1)	3959(1)	24(1)
C(12)	5008(1)	5961(1)	3936(1)	13(1)
C(13)	4870(2)	5092(1)	4411(1)	18(1)
C(14)	3813(2)	5518(1)	4888(1)	23(1)
C(15)	2909(2)	6804(1)	4880(1)	22(1)
C(16)	3117(2)	7610(1)	4391(1)	20(1)
N(1)	5222(1)	6076(1)	2888(1)	13(1)
N(2)	4152(1)	7211(1)	3924(1)	17(1)
O(1)	4671(2)	4012(1)	2450(1)	24(1)
O(2)	3872(2)	6445(1)	923(1)	26(1)

Table S10. Bond lengths [Å] and angles [°] for **2a**.

C(1)-O(1)	1.2374(12)
C(1)-N(1)	1.3464(12)
C(1)-C(2)	1.5170(14)
C(2)-C(3)	1.5166(14)
C(2)-H(2A)	0.9900
C(2)-H(2B)	0.9900
C(3)-O(2)	1.4400(14)
C(3)-C(4)	1.5278(19)
C(3)-H(3)	1.0000
C(4)-C(5)	1.5468(18)
C(4)-H(4A)	0.9900
C(4)-H(4B)	0.9900
C(5)-C(6)	1.519(2)
C(5)-H(5A)	0.9900
C(5)-H(5B)	0.9900
C(6)-O(2)	1.4351(17)
C(6)-H(6A)	0.9900
C(6)-H(6B)	0.9900
C(7)-N(1)	1.4589(12)
C(7)-C(12)	1.5201(13)
C(7)-C(8)	1.5638(14)
C(7)-H(7)	1.0000
C(8)-C(9)	1.5331(16)
C(8)-C(10)	1.5333(17)
C(8)-C(11)	1.5337(15)
C(9)-H(9A)	0.9800
C(9)-H(9B)	0.9800
C(9)-H(9C)	0.9800
C(10)-H(10A)	0.9800
C(10)-H(10B)	0.9800
C(10)-H(10C)	0.9800
C(11)-H(11A)	0.9800
C(11)-H(11B)	0.9800
C(11)-H(11C)	0.9800
C(12)-N(2)	1.3437(13)
C(12)-C(13)	1.3902(13)

C(13)-C(14)	1.3850(15)
C(13)-H(13)	0.9500
C(14)-C(15)	1.3893(18)
C(14)-H(14)	0.9500
C(15)-C(16)	1.3852(17)
C(15)-H(15)	0.9500
C(16)-N(2)	1.3488(13)
C(16)-H(16)	0.9500

O(1)-C(1)-N(1)	123.06(9)
O(1)-C(1)-C(2)	121.13(9)
N(1)-C(1)-C(2)	115.81(8)
C(3)-C(2)-C(1)	110.13(8)
C(3)-C(2)-H(2A)	109.6
C(1)-C(2)-H(2A)	109.6
C(3)-C(2)-H(2B)	109.6
C(1)-C(2)-H(2B)	109.6
H(2A)-C(2)-H(2B)	108.1
O(2)-C(3)-C(2)	109.05(9)
O(2)-C(3)-C(4)	104.71(9)
C(2)-C(3)-C(4)	115.29(9)
O(2)-C(3)-H(3)	109.2
C(2)-C(3)-H(3)	109.2
C(4)-C(3)-H(3)	109.2
C(3)-C(4)-C(5)	103.98(11)
C(3)-C(4)-H(4A)	111.0
C(5)-C(4)-H(4A)	111.0
C(3)-C(4)-H(4B)	111.0
C(5)-C(4)-H(4B)	111.0
H(4A)-C(4)-H(4B)	109.0
C(6)-C(5)-C(4)	103.52(11)
C(6)-C(5)-H(5A)	111.1
C(4)-C(5)-H(5A)	111.1
C(6)-C(5)-H(5B)	111.1
C(4)-C(5)-H(5B)	111.1
H(5A)-C(5)-H(5B)	109.0
O(2)-C(6)-C(5)	105.79(10)
O(2)-C(6)-H(6A)	110.6

C(5)-C(6)-H(6A)	110.6
O(2)-C(6)-H(6B)	110.6
C(5)-C(6)-H(6B)	110.6
H(6A)-C(6)-H(6B)	108.7
N(1)-C(7)-C(12)	109.86(7)
N(1)-C(7)-C(8)	111.39(8)
C(12)-C(7)-C(8)	114.55(8)
N(1)-C(7)-H(7)	106.9
C(12)-C(7)-H(7)	106.9
C(8)-C(7)-H(7)	106.9
C(9)-C(8)-C(10)	109.62(11)
C(9)-C(8)-C(11)	108.05(10)
C(10)-C(8)-C(11)	109.72(9)
C(9)-C(8)-C(7)	108.21(9)
C(10)-C(8)-C(7)	111.37(9)
C(11)-C(8)-C(7)	109.80(8)
C(8)-C(9)-H(9A)	109.5
C(8)-C(9)-H(9B)	109.5
H(9A)-C(9)-H(9B)	109.5
C(8)-C(9)-H(9C)	109.5
H(9A)-C(9)-H(9C)	109.5
H(9B)-C(9)-H(9C)	109.5
C(8)-C(10)-H(10A)	109.5
C(8)-C(10)-H(10B)	109.5
H(10A)-C(10)-H(10B)	109.5
C(8)-C(10)-H(10C)	109.5
H(10A)-C(10)-H(10C)	109.5
H(10B)-C(10)-H(10C)	109.5
C(8)-C(11)-H(11A)	109.5
C(8)-C(11)-H(11B)	109.5
H(11A)-C(11)-H(11B)	109.5
C(8)-C(11)-H(11C)	109.5
H(11A)-C(11)-H(11C)	109.5
H(11B)-C(11)-H(11C)	109.5
N(2)-C(12)-C(13)	122.11(9)
N(2)-C(12)-C(7)	118.34(8)
C(13)-C(12)-C(7)	119.52(9)
C(14)-C(13)-C(12)	119.29(10)

C(14)-C(13)-H(13)	120.4
C(12)-C(13)-H(13)	120.4
C(13)-C(14)-C(15)	119.18(10)
C(13)-C(14)-H(14)	120.4
C(15)-C(14)-H(14)	120.4
C(16)-C(15)-C(14)	118.01(10)
C(16)-C(15)-H(15)	121.0
C(14)-C(15)-H(15)	121.0
N(2)-C(16)-C(15)	123.45(11)
N(2)-C(16)-H(16)	118.3
C(15)-C(16)-H(16)	118.3
C(1)-N(1)-C(7)	122.17(8)
C(12)-N(2)-C(16)	117.96(9)
C(6)-O(2)-C(3)	104.36(10)

Symmetry transformations used to generate equivalent atoms:

Table S11. Anisotropic displacement parameters (Å^{2}x 10^3) for **2a**. The anisotropic displacement factor exponent takes the form: $-2\pi^2$[h^2 $a^{*2}U^{11}$ + ... + 2 h k a* b* U^{12}]

	U^{11}	U^{22}	U^{33}	U^{23}	U^{13}	U^{12}
C(1)	15(1)	16(1)	12(1)	-2(1)	-1(1)	0(1)
C(2)	19(1)	21(1)	12(1)	-2(1)	-2(1)	2(1)
C(3)	25(1)	20(1)	12(1)	-2(1)	0(1)	0(1)
C(4)	24(1)	50(1)	19(1)	2(1)	2(1)	-4(1)
C(5)	35(1)	34(1)	25(1)	6(1)	9(1)	-2(1)
C(6)	47(1)	33(1)	13(1)	2(1)	2(1)	-2(1)
C(7)	14(1)	14(1)	11(1)	0(1)	-1(1)	1(1)
C(8)	13(1)	26(1)	14(1)	1(1)	-1(1)	0(1)
C(9)	18(1)	57(1)	22(1)	-9(1)	4(1)	6(1)
C(10)	20(1)	30(1)	28(1)	9(1)	-5(1)	-9(1)
C(11)	17(1)	31(1)	24(1)	6(1)	-5(1)	2(1)
C(12)	13(1)	16(1)	11(1)	0(1)	0(1)	-2(1)
C(13)	17(1)	22(1)	16(1)	4(1)	2(1)	0(1)
C(14)	19(1)	35(1)	15(1)	5(1)	2(1)	-1(1)
C(15)	17(1)	34(1)	14(1)	-4(1)	2(1)	-2(1)
C(16)	20(1)	22(1)	18(1)	-5(1)	1(1)	-1(1)
N(1)	16(1)	13(1)	11(1)	-1(1)	-2(1)	0(1)
N(2)	19(1)	17(1)	15(1)	-2(1)	1(1)	-1(1)
O(1)	36(1)	14(1)	22(1)	-4(1)	-7(1)	0(1)
O(2)	33(1)	33(1)	13(1)	1(1)	-6(1)	1(1)

Table S12. Hydrogen coordinates (x 10^4) and isotropic displacement parameters ($Å^2$x 10^{3}) for **2a**.

	x	y	z	U(eq)
H(2A)	2284	5789	1899	20
H(2B)	3713	7070	2024	20
H(3)	4770	4727	1328	23
H(4A)	7827	5504	1506	37
H(4B)	7098	7050	1649	37
H(5A)	7621	7656	723	38
H(5B)	8311	6101	577	38
H(6A)	5013	7095	172	37
H(6B)	5293	5466	284	37
H(7)	5926	4457	3377	16
H(9A)	10722	5150	2911	49
H(9B)	8816	5524	2541	49
H(9C)	8929	4086	2879	49
H(10A)	8268	7729	3764	39
H(10B)	8259	7726	3074	39
H(10C)	10251	7435	3417	39
H(11A)	8901	4094	3969	36
H(11B)	8742	5535	4304	36
H(11C)	10683	5168	3949	36
H(13)	5494	4214	4408	22
H(14)	3707	4938	5217	28
H(15)	2171	7122	5201	26
H(16)	2499	8489	4383	24

Table S13. Torsion angles [°] for **2a**.

O(1)-C(1)-C(2)-C(3)	-59.22(13)
N(1)-C(1)-C(2)-C(3)	120.41(10)
C(1)-C(2)-C(3)-O(2)	177.49(8)
C(1)-C(2)-C(3)-C(4)	-65.10(13)
O(2)-C(3)-C(4)-C(5)	-25.32(13)
C(2)-C(3)-C(4)-C(5)	-145.14(11)
C(3)-C(4)-C(5)-C(6)	1.04(15)
C(4)-C(5)-C(6)-O(2)	23.76(15)
N(1)-C(7)-C(8)-C(9)	-60.85(12)
C(12)-C(7)-C(8)-C(9)	173.70(10)
N(1)-C(7)-C(8)-C(10)	59.71(11)
C(12)-C(7)-C(8)-C(10)	-65.74(11)
N(1)-C(7)-C(8)-C(11)	-178.56(8)
C(12)-C(7)-C(8)-C(11)	55.99(11)
N(1)-C(7)-C(12)-N(2)	-30.38(11)
C(8)-C(7)-C(12)-N(2)	95.86(10)
N(1)-C(7)-C(12)-C(13)	147.94(9)
C(8)-C(7)-C(12)-C(13)	-85.81(11)
N(2)-C(12)-C(13)-C(14)	0.25(16)
C(7)-C(12)-C(13)-C(14)	-178.02(9)
C(12)-C(13)-C(14)-C(15)	0.22(16)
C(13)-C(14)-C(15)-C(16)	-0.25(17)
C(14)-C(15)-C(16)-N(2)	-0.17(17)
O(1)-C(1)-N(1)-C(7)	-0.57(15)
C(2)-C(1)-N(1)-C(7)	179.81(8)
C(12)-C(7)-N(1)-C(1)	-123.56(9)
C(8)-C(7)-N(1)-C(1)	108.43(10)
C(13)-C(12)-N(2)-C(16)	-0.65(15)
C(7)-C(12)-N(2)-C(16)	177.63(9)
C(15)-C(16)-N(2)-C(12)	0.62(16)
C(5)-C(6)-O(2)-C(3)	-40.99(13)
C(2)-C(3)-O(2)-C(6)	165.21(10)
C(4)-C(3)-O(2)-C(6)	41.30(12)

Symmetry transformations used to generate equivalent atoms:
